# Light-Mediated Binaphthyl Enhanced [2 + 2] Dearomatization of Heterocycles via an Energy-Transfer Process

**DOI:** 10.1021/acs.orglett.5c02570

**Published:** 2025-08-04

**Authors:** Maurizio Chiminelli, Carlotta Galbardi, Raimondo Maggi, Franca Bigi, Luca Capaldo, Nicola Della Ca’, Rosanna Viscardi, Luciano Marchiò, Giovanni Maestri, Matteo Lanzi

**Affiliations:** † Department of Chemistry, Life Sciences and Environmental Sustainability, 9370 Università di Parma, Parco Area delle Scienze, 17/A, 43124 Parma, Italy; ‡ ENEA, Casaccia Research Center, 00123 Santa Maria di Galeria, Rome, Italy

## Abstract

Three-dimensional heterocycles are crucial in medicinal chemistry, yet efficient methods for their preparation remain limited. We report a light-mediated [2 + 2] dearomatization strategy for simple and benzo-fused heteroaromatics via energy-transfer activation. A binaphthyl cocatalyst combined with an Ir-photocatalyst enables high yields and selectivity under mild conditions. Mechanistic studies reveal the fundamental role of the cocatalyst in stabilizing reactive intermediates via dispersion interactions. The versatility of the products was demonstrated through synthetic applications that smoothly increased the molecular complexity.

## Graphical abstract

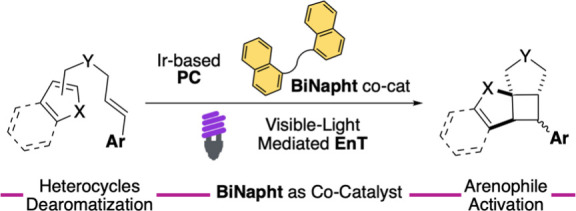

Three-dimensional heterocyclic frameworks hold a pivotal position in medicinal chemistry, as they play a distinctive role in shaping the properties of therapeutic agents.^1^ As a consequence, the efficient construction of these architectures remains of continuous interest in organic chemistry.^2^ Among others, strategies that enable the transformation of simple and readily available starting materials like aromatic units into complex three-dimensional molecules are particularly promising.^3^

Light-mediated dearomative methodologies can allow access to otherwise inaccessible reactions via energy transfer (EnT) (Figure 1a).^4^ These strategies can bypass the steep thermodynamic barriers of thermal approaches by accessing a photoexcited triplet state, which can yield, in turn, sophisticated 3D-frameworks.^5^

**Figure 1:**
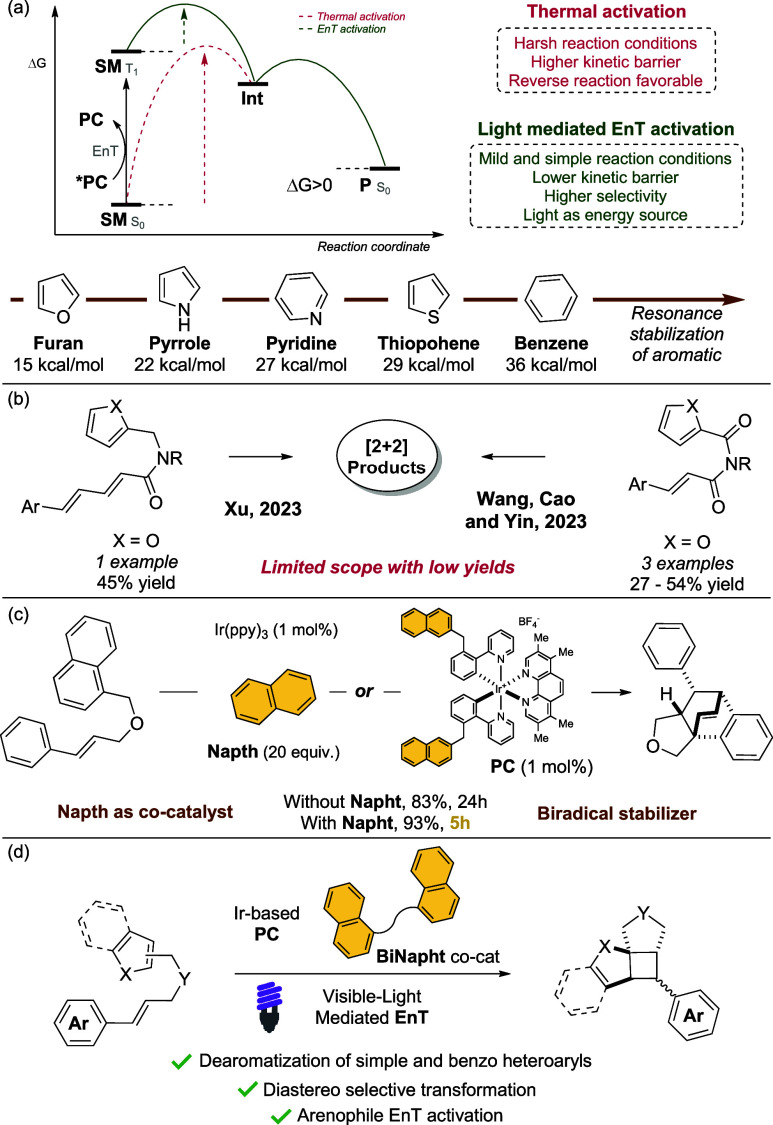
(a) Dearomative reactions and resonance stabilization of aromatics; (b) visible-light-mediated [2 + 2] dearomatization of furan; (c) our previous works on the [4 + 2] dearomatization of arenes with alkenes; (d) the present [2 + 2] dearomatization of heteroarenes with alkenes.

The [2 + 2] dearomative cycloaddition of indole has been investigated, but the activation of this heteroarene requires the presence of a conjugated auxochrome, such as a carbonyl or phenyl unit.^6^ Analogous transformations on benzofurans and benzothiophenes remain limited.^7^ The [2 + 2] dearomatization of simple aromatics such as furan has been reported using UV light by Schreiber.^8^

The use of styryl dienes amides was reported by Xu in 2023, and a single example on furan was disclosed (Figure 1b).^9^ Wang, Cao, and Yin used acyl-heterocycles conjugated with aryl-acrylamides,^10^ and the desired products were obtained in low yields. Baik and Meggers reported the first asymmetric [2 + 2] dearomatization of benzothiophenes and benzofurans using a chiral rhodium photocatalyst. Activation of the substrate still required a conjugated acyl group on the 2-position of the heterocycle.^11^

Our research group has recently validated a new class of cocatalysts, namely, naphthalene and binaphthyl derivatives (Figure 1c).^12^ These have been demonstrated to promote otherwise silent reactions by stabilizing the triplet intermediates of EnT sequences via dispersion interaction, including the [4 + 2] *para*-cycloadditions on arenes and naphthalenes.^13^

The [2 + 2] dearomative cycloaddition of simple heteroarenes via selective activation of an arenophile partner remains underexplored.^14^ Therefore, we targeted a similar reactivity because light-mediated dearomative cycloadditions enable access to remarkable three-dimensional heterocyclic architectures.

We hypothesized that a simple cinnamyl arenophile could enable the dearomative cycloaddition of simple heteroaromatics under light-mediated EnT conditions (Figure 1d). We began our investigation by reacting substrate **1a** in the presence of **PC1** under blue LED irradiation. A small amount of product **2a** was isolated (14%), along with the *Z*-isomer of **1a** (46%) and trace amounts of **2a′** (Table 1, entry 1). Performing the reaction in the presence of naphthalene (20 equiv), **2a** was obtained in 63% yield (Table 1, entry 2).^12a,12b,15^

**Table 1:**
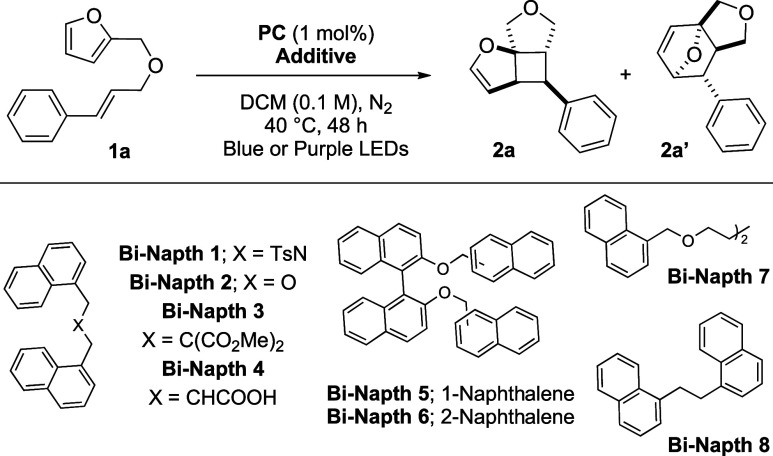
Optimization of the [2 + 2] Dearomatization of Furans

			Yields	
Entry^a^	PC	Co-catalyst	**2a**	**2a**′ (*Z*-**1a**)
**1** ^b^	**PC1**	-	14%	Traces (46%)
**2** ^b^	**PC1**	Napht (20 equiv)	63%	-
**3** ^b^	**PC1**	BiNapht 1 (30 mol %)	38%	9% (17%)
**4** ^b^	**PC1**	BiNapht 2 (30 mol %)	43%	10% (10%)
**5** ^b^	**PC1**	BiNapht 3 (30 mol %)	41%	10% (7%)
**6** ^b^	**PC1**	BiNapht 4 (30 mol %)	14%	4% (49%)
**7** ^b^	**PC1**	BiNapht 6 (30 mol %)	49%	11% (13%)
**8** ^b^	**PC1**	BiNapht 8 (30 mol %)	45%	14% (12%)
**9** ^[^ ^c^	**PC1**	BiNapht 8 (30 mol %)	40%	- (23%)
**10** ^c^	**PC2**	BiNapht 8 (30 mol %)	82%	-
**11** ^c^	**PC3**	BiNapht 8 (30 mol %)	65%	9%(9%)
**12** ^c^ ^,^ ^d^	**TXT**	BiNapht 8 (30 mol %)	77%	-
**13** ^c^	**PC2**	BiNapht 8 (10 mol %)	85%	-
**14** ^c^	**PC2**	BiNapht 8 (50 mol %)	74%	-
**15** ^c^	**PC2**	-	50%	12%

a Conditions: unless otherwise specified **1a** (0.15 mmol, 1 equiv), **PC** (1 mol %) in DCM (0.1 M) in 5 mm NMR tube under N_2_; yields were determined using 1,3,5-trimethoxybenzene as standard.

b Irradiated with blue LEDs (λ = 456 nm) for 48 h;

c Irradiated with purple LEDs (λ = 390 nm) for 48 h;

d TXT was used in 10 mol %; **PC1** = [Ir­(ppy)_3_]; **PC2** = [Ir­(dF­(CF_3_)­ppy)_2_(dtbpy))­PF_6_], **PC3** = [Ir­(dF­(CF_3_)­ppy)_2_(bpy))­PF_6_], **TXT** = thioxantone; see page S18 of the Supporting Information for further details.

The molar excess of the cocatalyst may affect the sustainability of the method. Thus, a library of polynaphthyls (**BiNapth1–8**) was prepared adopting a multivalent strategy. A catalytic amount of **BiNapth**s (30 mol %) (Table 1, entries 2–7) allowed the formation of the desired product **2a** in up to 49% yield. **BiNapht8** was selected for further screening due to its simpler synthesis and cleaner reaction. Light influenced the reaction outcome. On irradiating with purple LEDs instead of blue ones, a lower yield was obtained using **PC1**. **PC2** improved the outcome (Table 1, entries 8, 9), while **PC3** and **PC4** produced **2a** with reduced efficiency (Table 1, entry 10). The use of organic photosensitizers or further modification of the cocatalyst loading has not improved the reaction outcome (Table 1, entries 12–15).

Having successfully established the optimal reaction conditions (Table 1, entry 9), namely, the use of **PC2** (1 mol %), **Bi-Napth8** (30 mol %), and purple light irradiation, we proceeded to assess the robustness of the method (Scheme 1). The structure of compound **2a** was initially elucidated through an X-ray crystallographic analysis, confirming the relative configuration of the four newly generated stereocenters. Notably, a single stereoisomer was consistently observed in mostcases. Substitutions on the alkyl chain were well tolerated; either the methyl or phenyl group provided the desired compounds **2b** and **2c**, respectively, in 55% and 70% yield. Both products were obtained as mixtures of two diastereoisomers from the corresponding racemic reagents.

**Scheme 1:**
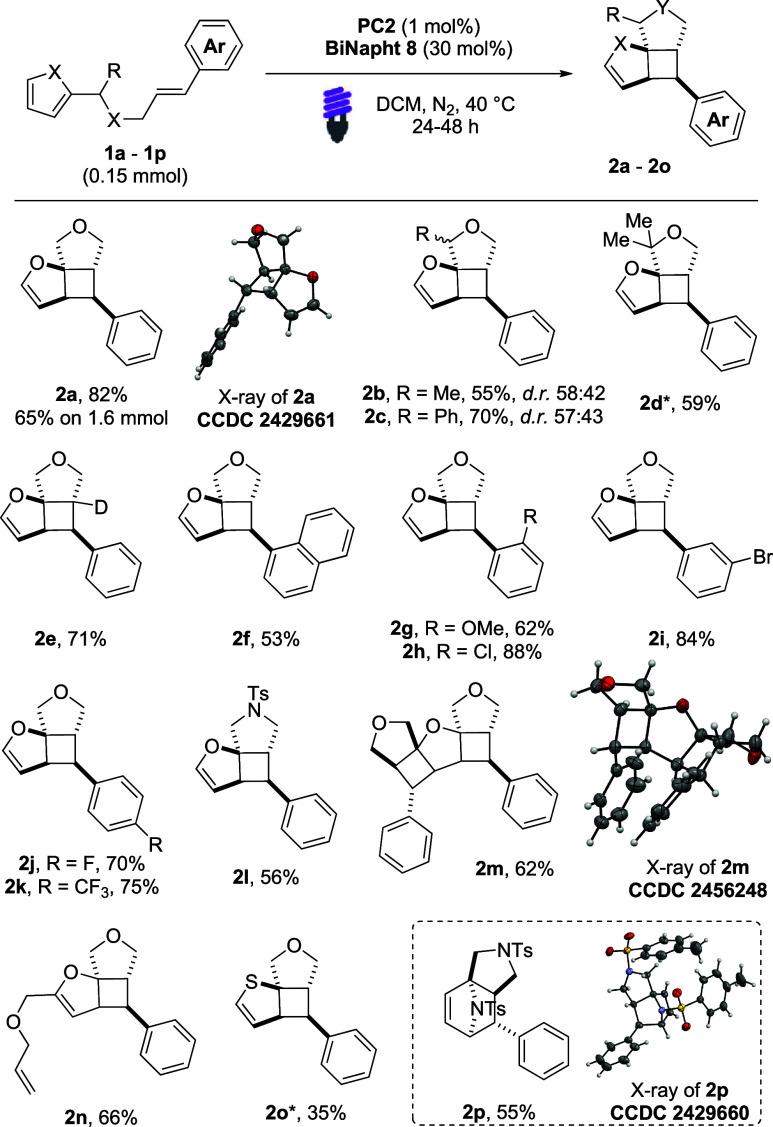
Reaction Scope of [2 + 2] Dearomatization of Heteroaromatics^a^

a Unless otherwise specified reactions were conducted on a 0.15 mmol scale following the conditions of entry 12 of Table 1. *Conditions from entry 1 Table 1 were used. Thermal ellipsoids are drawn at the 50% probability level.

The Thorpe–Ingold effect was not observable, while steric hindrance may have contributed to the moderate yield of **2d** (59%). Given that deuterium labeling can enhance pharmacokinetic properties, a deuterated alkene (**1e**) was subjected to the reaction conditions. The product **2e** was obtained with no impact on the stereochemical outcome and in good yield.^16^ Substituents on the aromatic fragments were then taken into consideration. Extended aromatic units, such as the 1-naphthyl units, might affect the reaction by presenting a steric effect, and **2f** was recovered in a 53% yield. Electron-rich substituents reduced the reaction productivity, and **2g** was obtained in a moderate yield. Conversely, a series of electron-withdrawing atoms and groups afforded the highest yields. Functional tricyclic products bearing 2-chloride, **2h**, and 3-bromide, **2i**, were synthesized in 88% and 84% yields, respectively. Fluorine-containing moieties were also well tolerated, and **2j** and **2k** were isolated in 70% and 75% yield, respectively. Tethering the reaction partners with a protected nitrogen atom allowed us to generate a dihydropyrrole ring, in synthetically useful yield (**2l**, 56%). Alkyl fragments were strategically incorporated at the 5-position of the furan ring. Incorporation of an additional cinnamyl unit enabled the selective formation of pentacyclic **2m** in a 62% yield as a single diastereoisomer. The product **2m** presents four new C–C bonds that were formed with the concomitant establishment of eight contiguous stereocenters. It is hypothesized that a [2 + 2] dearomative cycloaddition begins the cascade, yielding a dihydrofuran intermediate. The sequential activation of the second cinnamyl arm triggers a second [2 + 2] cycloaddition, whose diastereocontrol is ensured by the rigidity of the scaffold established in the initial dearomative process. Compound **2n** was obtained in 66% yield, and it retains an allyl group that is amenable to further synthetic elaboration.

To further explore the dearomatization of simple heteroaromatics, we applied our optimized reaction conditions to thiophene **1o** and protected pyrrole **1p**. While the target product **2o** was obtained in 35% yield, an intriguing contrast was noted with product **2p**, where the [4 + 2]-cycloadduct was recovered (for details on regiochemisty studies see SI p S56).

The protocol was extended to include benzoheterocycles (Scheme 2). Benzofuran derivatives **3a** and **3b** smoothly underwent the [2 + 2] dearomative cycloaddition, leading to products **4b** and **4a** in 92% and 93% yield, respectively. Compared to the previous series of substrates (Scheme 1), the dearomatization of benzoheterocycles proceeds smoothly even without the cocatalyst. This difference can be attributed to the lower aromatic stabilization energy of benzoheterocyclic systems.^4b,17^ Benzoheterocyclic products were obtained as a separable mixture of two diastereoisomers. This phenomenon can be explained by the stability of an intermediate triplet that has a benzyl radical character, which allows its rotation prior to the final bond-forming step. Benzothiophenes **3c**–**3f** also reacted efficiently, yielding products with high yields and consistent diastereoselectivity. The structure and the stereochemistry of the products were confirmed by X-ray analysis of compounds **4c** and **4c′**. Indoles were also successfully investigated. Both **4g** and **4h** were obtained in excellent yields. These results showed that the method allows the functionalization of indoles that do not present any additional auxochrome.^7b,7c^

**Scheme 2:**
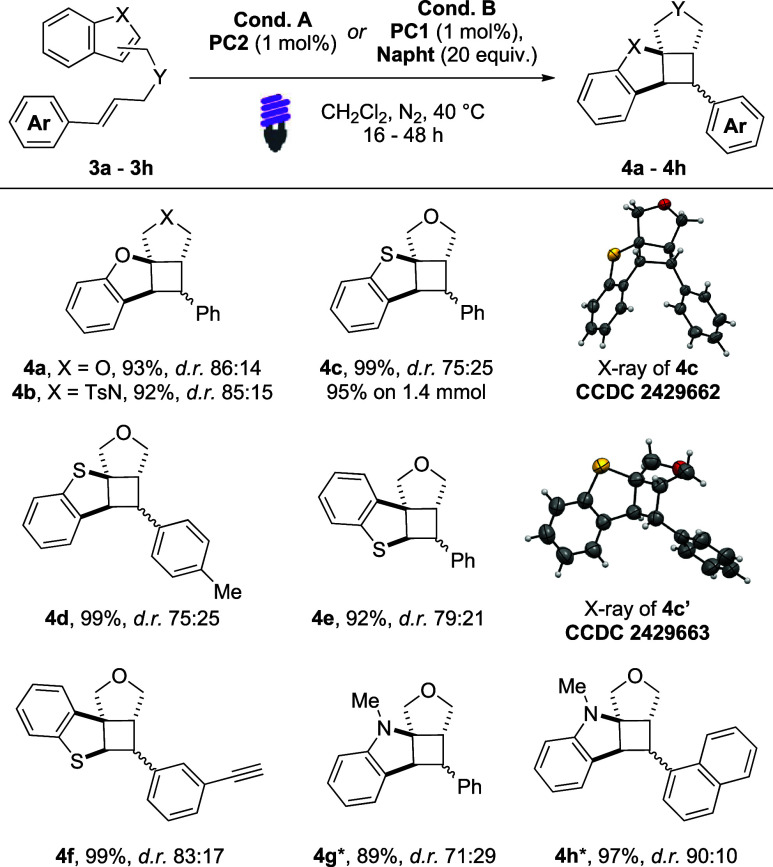
Reaction Scope of [2 + 2] Dearomatization of Benzoheteroaromatics^a^

a Unless otherwise specified, reaction were conducted on a 0.15 mmol scale following the conditions of entry 1 of Table 1;. *Conditions from entry 2 Table 1 were used. Thermal ellipsoids are drawn at the 50% probability level.

In order to assess the scalability of the method, **2a** was prepared in a 1.6 mmol scale (62% yield). We then explored the potential application of this product to further increase the molecular complexity (Scheme 3). The compound **2a** was subjected to oxidative conditions using *m*CPBA, delivering the 1,2-dihydroxylation product **5** in excellent yield. A CAN-mediated cycloaddition with 1,3-cyclohexanedione led to the formation of pentacyclic product **6**. This step underscores the potential of our methodology to access structurally complex frameworks through mild and selective transformations.

**Scheme 3:**
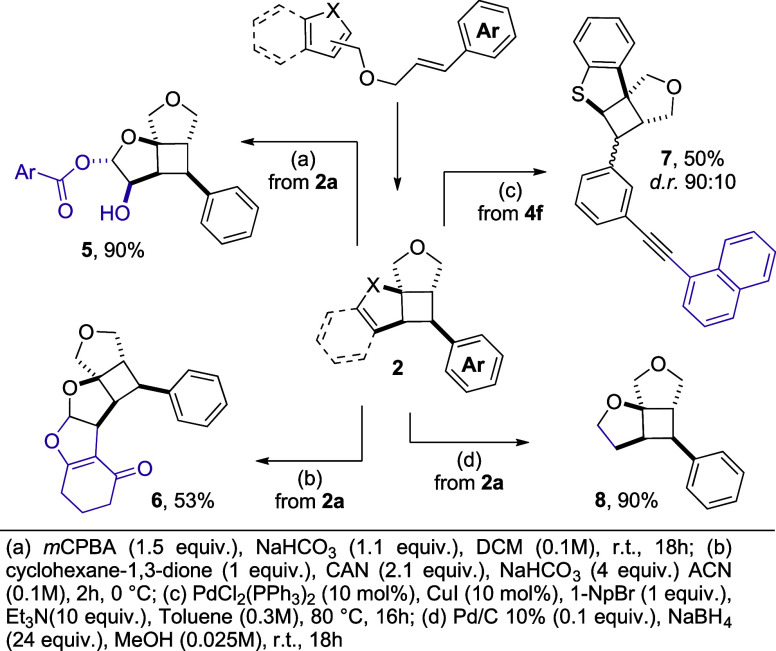
Valorization of the Products

Further diversification was achieved by the Sonogashira cross-coupling reaction, which afforded **7** in a moderate, yet synthetically useful yield. Finally, the reduction of the vinyl ether moiety yielded the three-dimensional sp^3^-rich tricyclic structure **8** in quantitative yield.

Mechanistic surveys to elucidate the reaction pathway and assess the influence of the cocatalyst **BiNapht8** were conducted experimentally and via DFT calculations.^12b,13a,13b^

To investigate the potential involvement of the SET mechanism, cyclic voltammetry studies were conducted on **1a**, **3c**, and **BiNapht8**. The absence of oxidation peaks suggests that SET pathways are unlikely to contribute under the optimized reaction conditions. (See the Supporting Information.)

Stern–Volmer quenching studies of **PC2** were performed using **1a**, **1q**, **3c**, and **BiNapht8** as quencher (Figure 2). The reagent **1q** had the lowest quenching efficiency (*K*_SV_ = 2.5 M^–1^), while **1a** and **3c** were better quenchers (*K*_SV_ = 766.4 and 647.6 M^–1^, respectively). These findings suggest that the cinnamyl fragment is essential for the reaction outcome, as further supported by the lack of reactivity observed using **1q**. Finally, **BiNapht8** exhibited the highest quenching constant of the series (*K*_SV_ = 1905.0 M^–1^).

**Figure 2:**
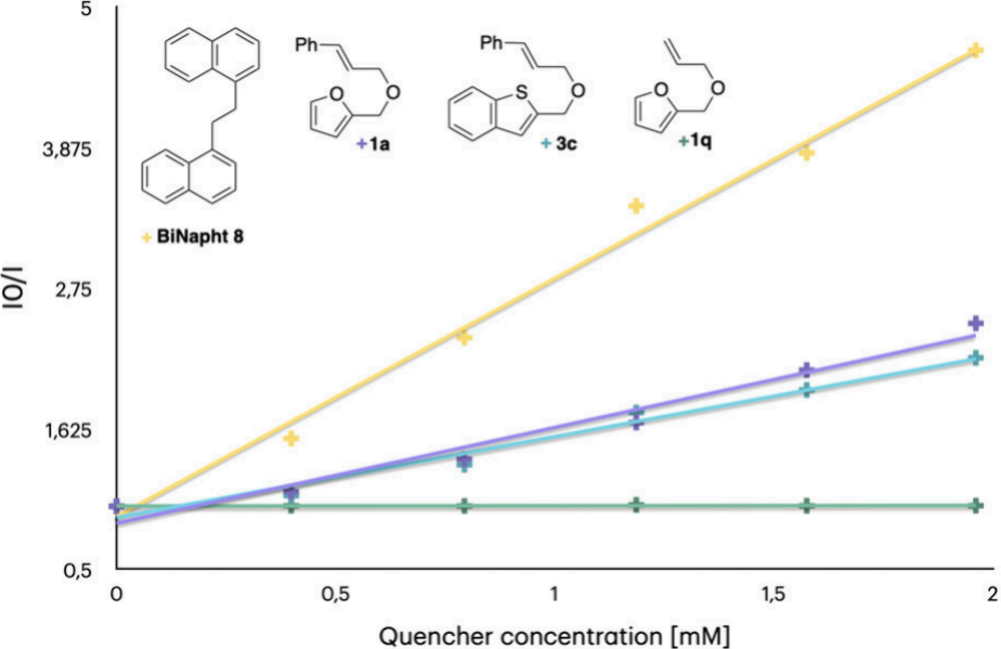
Stern–Volmer quenching studies of **PC2**.

DFT calculations were performed using DCM as an implicit solvent at the M06/Def2-TZVP level, which proved to be reliable to describe related EnT processes.^18^ The modeling was performed using substrate **1a** and comparing the energy profile of the reactions with and without the **BiNapht8** (Scheme 4). The calculated triplet energies (*E*_T_) of **1a** and **BiNapht8** (49.1 and 55.1 kcal/mol, respectively) are compatible with their direct sensitized from **PC2** (*E*_T_ = 60.1 kcal/mol).^19^ In the ground state, **BiNapht8** and **1a** can reversibly form the [**BiNapht8**:**1a**] complex (Δ*G* = +0.8 kcal/mol). The sensitization of the [**BiNapht8**:**1a**] adduct is more energetically favorable (*E*_T_ = 48.0 kcal/mol) than that of free substrate **1a**. This result suggests that the activation of the substrate is more favorable in the presence of the cocatalyst. This might arise from stronger dispersion interactions in the triplet-state adduct ^**3**^ **[1a:BiNapht8]**.^13a^ Interestingly, the subsequent 5-*exo*-trig cyclization is essentially barrierless, modeling the reaction of both ^**3**^ **Ia** and ^**3**^ **[Ia:BiNapht8]** (details in the SI). The product **2a**, which is likely formed via sequential intersystem crossing (ISC) and radical recombination, is less stable than **1a** (Δ*G* = +7.7 kcal/mol), showing the endergonic character of the reaction that reflects the loss of the aromatic stabilization.^4b,10,15^

**Scheme 4:**
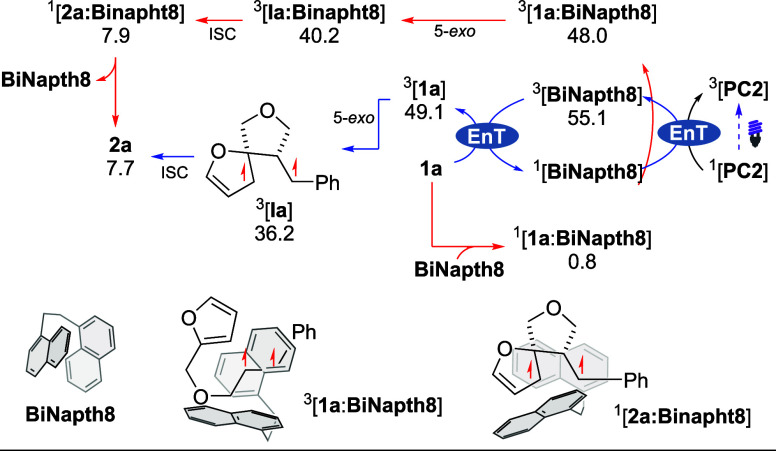
Proposed Mechanism

Experimental and computational evidence suggests that two complementary reaction pathways, both involving the cocatalyst **BiNapht8**, are capable of delivering **2a**. One involves a relay-type activation of **1a** by ^3^[**BiNapht8**] (Scheme 4, blue line), while a second proceeds via direct photoexcitation of a preformed^1^ **1a**:**BiNapht8**] complex (Scheme 4, red line).

In conclusion, we developed an efficient, broad [2 + 2] dearomative cycloaddition of simple and benzo heteroaromatics via EnT. High yields and selectivity were achieved under mild conditions combining an Ir photocatalyst with a binaphthyl cocatalyst. Computational studies revealed that the cocatalyst stabilizes intermediates through dispersion interactions. The synthetic utility of the products was demonstrated through diverse postfunctionalization, underscoring the value of this platform for building molecular complexity. By leveraging the design of chiral binphthyl cocatalyst, this approach also holds promise for future development of enantioselective dearomative cycloadditions, opening a potential avenue in asymmetric synthesis.

## Supplementary Material



## Data Availability

The data underlying this study are available in the published article and its Supporting Information.

## Notes

Detailed procedures, copy of the spectra, and detailed mechanistic studies (PDF)
