# Supplementary material for: Light-Mediated Binaphthyl Enhanced [2 + 2] Dearomatization of Heterocycles via an Energy-Transfer Process
Source: Org Lett. 2025 Aug 4;27(32):8909–14. doi: 10.1021/acs.orglett.5c02570 (PMC12362593; doi:10.1021/acs.orglett.5c02570)
Supplement: Supplementary file 1 [file ol5c02570_si_001.pdf]

Light-Mediated BiNaphthyl Enhanced [2+2] Dearomatization of Heterocycles *via* Energy-Transfer Process

Maurizio Chiminelli,[a] Carlotta Galbardi,[a] Raimondo Maggi,[a] Franca Bigi,[a] Luca Capaldo,[a] Nicola Della Ca',[a] Rosanna Viscardi,[b] Luciano Marchiò,[a] Giovanni Maestri,[a*] Matteo Lanzi[a*]

[a] Department of Chemistry, Life Sciences and Environmental Sustainability, Università di Parma, Parco Area delle Scienze, 17/A, 43124 Parma, Italy

[b] ENEA, Casaccia Research Center, 00123 Santa Maria di Galeria, Rome, Italy

Table of Contents

General remarks	3
Setup adopted for the catalytic reactions.	4
Synthesis of substrates	7
Synthesis of 1-methyl-1H-indole-2-carbaldehyde	7
Synthesis of alcohols	7
Synthesis of primary Ts-amine	8
Synthesis of cinnamic-2-d acid	8
Synthesis of esters	8
Reduction of esters with DIBAL	9
Synthesis of bromo derivatives of substituted cinnamyl alcohols	9
General procedure for the synthesis of reagents,	10
Characterization of substrates	11
Optimization experiments	17
General procedures for the [2+2] dearomatization	20
GP1-P	20
GP2-P	20
GP3-P	21
Products Characterization	22
Valorization of the products	31
Mechanistic Investigation	35
Absorption Spectra	35
Emission Spectra	37
Cyclic Voltammetry Experiments	39
Stern-Volmer quenching studies	42
Computational Data	46
Screening of functionals for the calculation of ^{3}[1a] energy	47
Summary of Energies	48
Computational study on Transition States (TS)	49
Comprehensive energy profiles	52
Intermolecular Non-Covalent Interaction for [1a:BiNapht8]	54
Conformational study of ^{3}[1a:BiNapht8] complex	55
Investigation on the stereo and regio selectivity of the reaction	56
Cartesian Coordinates of Optimized Structures	58
Crystallographic data	69
Copy of NMR spectra	79
References	140

General remarks

All chemicals those synthesis is not reported hereafter were purchased from commercial sources and used as received. Substrate **1q** was obtained following the previously reported procedure.[1]

Solvents were dried passing through alumina columns using an Inert® system and were stored under nitrogen.

Present visible-light promoted reactions did not require the use of dry solvents but the presence of molecular oxygen exerts a negative effect on their rate. All the reaction herein reported have been carried out in standard 5mm NMR tubes.

Chromatographic purifications were performed under gradient using a Combiflash® system and prepacked disposable silica cartridges or through isocratic flash chromatography using commercial 60 Å silica gel.

All reactions that required heating were performed with the use of high-vacuum grade silicon oil.

Reactions promoted by visible light were performed into standard 5 mm NMR tubes, surrounded by a commercial strip of 300 household leds (12V, 17W). These were put at a distance of ca 10 cm and irradiated with blue light or purple light (chip SMD5630-300 ip65).

^{1}H and ^{13}C NMR spectra were recorded at 300 K on a Bruker 400 MHz or a Jeol 600 MHz spectrometers using residual non-deuterated solvents as internal standards (7.26 ppm for ^{1}H-NMR and 77.00 ppm for ^{13}C-NMR for $CDCl_3$, 2.05 ppm for ^{1}H NMR and 29.84 ppm for ^{13}C NMR for acetone-d6). ^{19}F-NMR spectra were recorded in $CDCl_3$ at 298 K on a Jeol 600 spectrometer fitted with a BBFO probehead at 564 MHz. The terms *m*, *s*, *d*, *t*, *q* and quint represent multiplet, singlet, doublet, triplet, quadruplet and quintuplet respectively, and the term brs means a broad signal. Reported assignments were based on decoupling, COSY, NOESY, HSQC and HMBC correlation experiments.

Mass analyses were recorded on an Infusion Water Acquity Ultra Performance LC HO6UPS-823M instrument equipped with a SQ detector (Electrospray source); high-resolution mass analyses were recorded on a LTQ ORBITRAP XL Thermo Mass Spectrometer (Electrospray source).

Single crystal Data were collected with a Bruker D8 diffractometer equipped with PhotonII area detector, using a CuKα or a MoKα microfocus 4 radiation source. The data collection strategy covered the sphere of reciprocal space. Absorption corrections were applied using the program SADABS. The structure was solved with the SHELXT code. Fourier analysis and refinement were performed by the full-matrix least-squares methods based on F2 using SHELXL-2014 as implemented in Olex2. All the nonH atoms were refined with anisotropic displacement parameters. **CCDC** numbers 2429660, 2429661, 2429662, 2429663 and 2456248 contains respectively the X-Ray data for **2p**, **2a**, **4c**, **4c’** and **2m**. All the crystals used for the single crystal diffraction were obtained through slow evaporation of a mixture of hexane and ethyl acetate.

Setup adopted for the catalytic reactions.

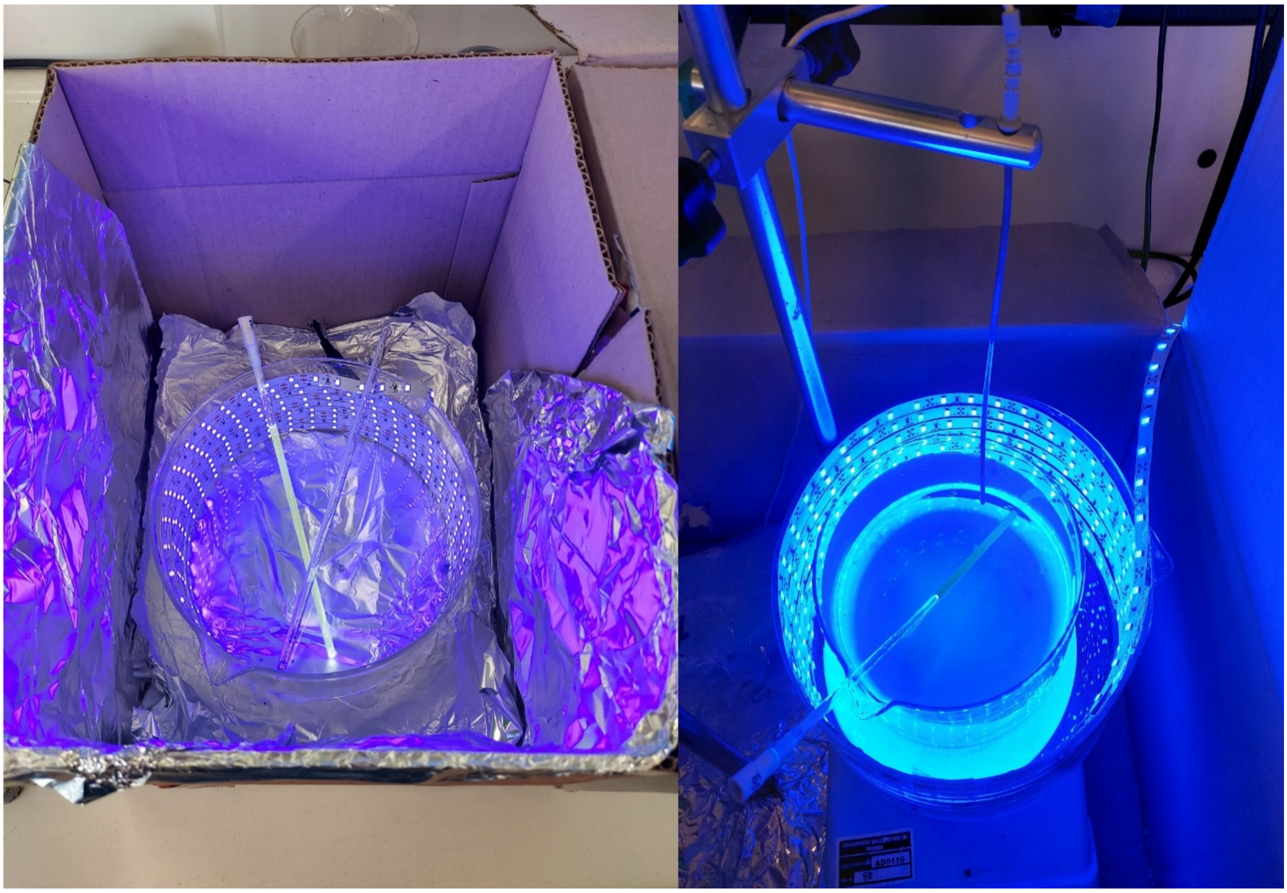

Left: setup using Violet LEDs; Right: setup using Blue LEDs

Emission spectra of the LEDs used in this work

Emission spectra of light sources were measured with a Jeti Spectroradiometer specbos 1211UV

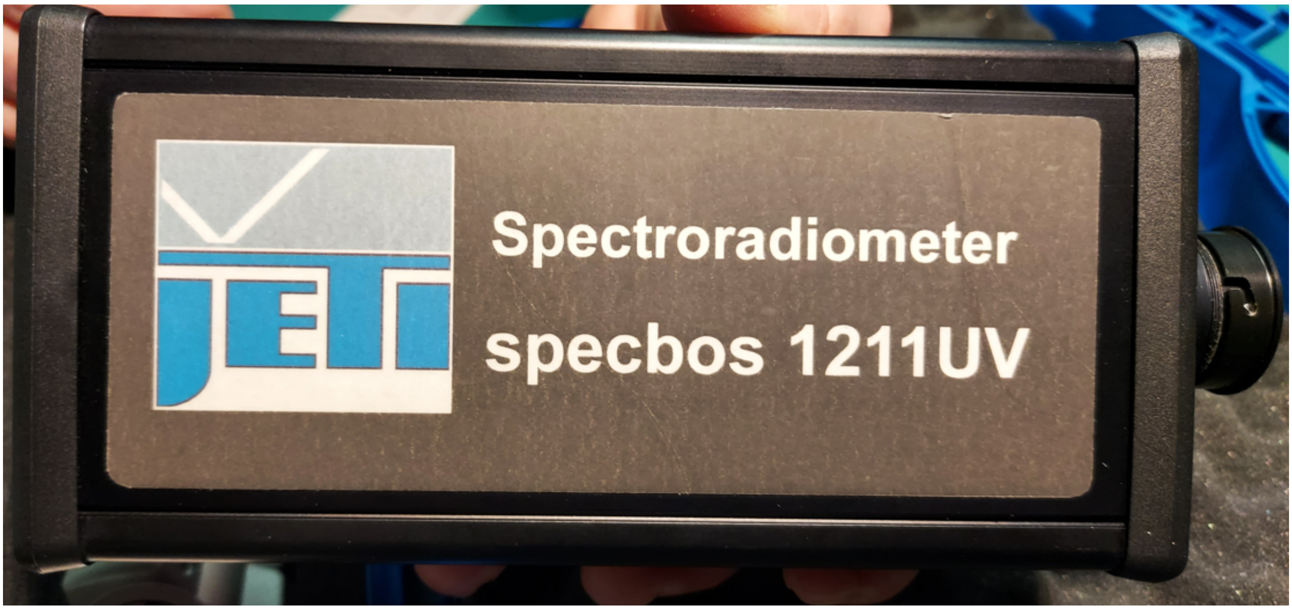

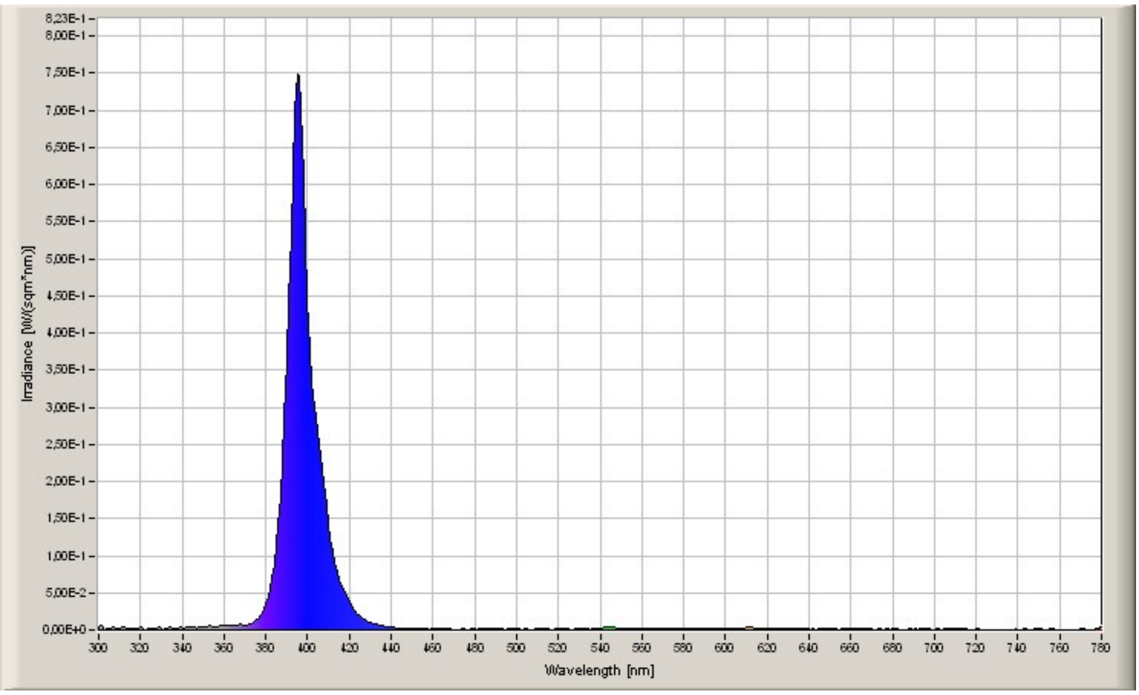

Emission spectra of the Violet LEDs light

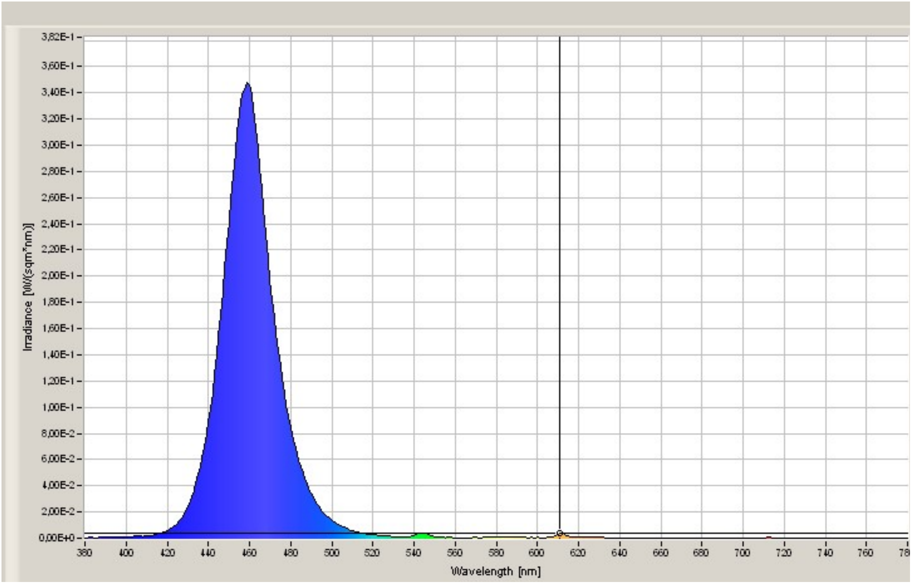

Emission spectra of the Blue LEDs light

Synthesis of substrates

Synthesis of 1-methyl-1H-indole-2-carbaldehyde

To a solution of 1-methyl-1H-indole (1 equiv.) in Et_2O (0.7 M) at 0 °C, *n*BuLi (2.5 M in hexane, 1.1 equiv.) was slowly added, and the mixture was refluxed for 3 hours. The reaction was cooled down to room temperature, then DMF (1.5 equiv.) was added, and the solution was refluxed for 5 hours. After complete conversion as monitored by TLC, the mixture was quenched with a saturated NH_4Cl solution and extracted with EtOAc (3 times). The combined organic layers were dried over Na_2SO_4 and concentrated under reduced pressure. The crude was purified by chromatography on silica gel (*n*Hex/EtOAc gradient) to afford the desired product (Yield = 63%).[2]

Synthesis of alcohols

A solution of aldehyde/ketone (1 equiv.) in MeOH (0.2 M) was cooled to 0 °C and $NaBH_4$ (1.5 equiv.) was then added in three portions. The mixture was stirred at room temperature until completion, monitoring the process by TLC. The solution was then quenched with saturated NH_4Cl solution and solvent was evaporated. Then, the product was extracted with EtOAc. The organic phase was washed with brine, dried over Na_2SO_4 and concentrated under reduced pressure. The resulting crude was used without further purification for the next step.

Synthesis of furan-2-yl(phenyl)methanol

PhMgBr (1.2 M in THF, 1.25 equiv.) was slowly added to a solution of furfural (1 equiv.) in THF (0.5 M) at 0 °C. The mixture was stirred at room temperature for 18 hours. After complete conversion as monitored by TLC, the solution was quenched with saturated NH_4Cl solution and extracted with EtOAc. The combined organic phase was then washed with brine, dried over Na_2SO_4 and concentrated under reduced pressure. The crude was finally purified by chromatography on silica gel (*n*Hex/EtOAc gradient) to afford the desired product. (Yield = 95%).[3]

Synthesis of 2-(furan-2-yl)propan-2-ol

*n*BuLi (2.5 M in hexane, 1.2 equiv.) was slowly added to a solution of furan (1 equiv.) in THF (1M) at -10 °C. The mixture was stirred at the same temperature for 2 hours, before adding dry acetone (1.2 equiv.). After complete conversion as monitored by TLC, the solution was quenched with saturated NH_4Cl solution and extracted with EtOAc. The combined organic phase was then washed with brine, dried over Na_2SO_4 and concentrated under reduced pressure. The crude was finally purified by chromatography on silica gel (*n*Hex/EtOAc gradient) to afford the desired product. (Yield = 56%).[4]

Synthesis of primary Ts-amine

Via reductive amination: To a solution of aldehyde (1 equiv.), $TsNH_2$ (1 equiv.) and TEA (5 equiv.) in DCM (0.04 M) was slowly added $TiCl_4$ (1M in DCM, 0.5 equiv.) at 0 °C and the mixture was then stirred at room temperature for 18 hours. The reaction was then quenched with water and extracted with DCM. The combined organic phase was washed with brine, dried over Na_2SO_4, filtered and concentrated under reduced pressure. The crude was later dissolved in MeOH (0.1 M) and $NaBH_4$ (1.25 equiv.) was added portion wise at 0 °C. After 2 hours of stirring at room temperature, the solvent was evaporated and then diluted with EtOAc. The combined organic phase was then washed with brine, dried over Na_2SO_4 and concentrated under reduced pressure. The crude was finally purified by chromatography on silica gel (nHex/EtOAc gradient) to afford the desired product. Yields between 63-90%.

Tosylation of primary amines: To a solution of primary amine (1.05 equiv.) and TEA (1.05 equiv.) in DCM (0.4 M) was slowly added TsCl (1 equiv.) at 0 °C, and the reaction was stirred at room temperature for 18 hours. After complete conversion as monitored by TLC, the solution was quenched with water, extracted with DCM and the organic phase was washed with water and brine. Then, the organic phase was dried over with Na_2SO_4, filtered and concentrated under reduced pressure. The crude was finally purified by chromatography on silica gel (nHex/EtOAc gradient) to afford the desired product. Yields between 85-95%.

Synthesis of cinnamic-2-*d* acid

In a round bottom flask equipped with a magnetic stirring bar and a condenser a solution of malonic acid (1 equiv.), piperidine (0.35 equiv.) and D_2O (14 equiv.) in pyridine (1.5 M) were refluxed for 2 hours; benzaldehyde (1 equiv.) was then added, and the mixture was stirred for an additional 3 hours. The resulting mixture was poured in a HCl solution (10% m/V), the precipitate was filtered, washed with water and dried under vacuo affording the corresponding carboxylic acid (Yield = 53%; deuterium incorporation: 84%).

Synthesis of esters

Via Fisher esterification: In a schlenk equipped with a magnetic stirring bar, were dissolved cinnamyl acid (1 equiv.) and sulfuric acid (cat.) in methanol (0.5M). The reaction was refluxed for 18 hours. After complete conversion as monitored by TLC, the solution was quenched with water, extracted with EtOAc and the organic phase was washed with water and brine. Finally, the organic phase was dried over with Na_2SO_4, filtered and concentrated under reduced pressure. The resulting crude was used without further purification for the next step.

Via Heck coupling: In a Schlenk tube equipped with a magnetic stirring bar under nitrogen atmosphere, were added $Pd(OAc)_2$ (20 mol%), P(*o*-tol)$_3$ (40 mol%), TEA (1.5 equiv.), acrylate (1.3 equiv.) and the aryl halide (1 equiv.) in DMF (1 M). The resulting mixture was stirred at 120 °C for 18 h. After complete conversion as monitored by TLC, the mixture was diluted with EtOAc, washed twice with water and a saturated LiCl solution, dried with Na_2SO_4 and concentrated under reduced pressure. The crude was purified by chromatography on silica gel (*n*Hex/EtOAc gradient). Yields between 40-92%.

Via Wittig olefination: To a solution of phosphonium ylide (1.08 equiv.) in MeCN (0.12 M) the desired aldehyde (1 equiv.) was added. The resulting mixture was refluxed for 45 min under stirring. After complete conversion monitored by TLC, the mixture was concentrated under reduced pressure and purified by chromatography on silica gel (*n*Hex/EtOAc gradient). Yields between 70-96%.

Reduction of esters with DIBAL

To a solution of ester (1 equiv.) in THF (0.25M) at -78 °C was slowly added DIBAL (1M in toluene, 2.3 equiv.) and the mixture was stirred at -78° C for 2 hours. The reaction was then warmed to 0 °C before adding saturated NH_4Cl solution and Rochell's salt. The mixture was then extracted with EtOAc, and the organic phase was washed with brine, dried over Na_2SO_4 and concentrated under reduced pressure. The crude was purified by chromatography on silica gel (nHex/EtOAc gradient). Yields between 60-90%.

Synthesis of bromo derivatives of substituted cinnamyl alcohols

To a stirred solution of alcohol (1 equiv.) in dry Et_2O (0.8 M) under nitrogen was added PBr_3 (1.05 equiv.) at 0°C. The resulting mixture was stirred at 0 °C for 30 minutes. The solution was quenched with saturated $NaHCO_3$ solution and extracted with DCM. The combined organic phase was washed with water and brine, dried over Na_3SO_4, filtered and concentrated under reduced pressure. The resulting crude was used without further purification for the next step.

General procedure for the synthesis of reagents,

GP-1: In a Schlenk-type flask containing TBAI (5 mol%) and the desired alcohol (1 equiv.) in dry THF (0.5 M) was added NaH (60% wt, 1.15 equiv.) at 0 °C under nitrogen atmosphere. The reaction was stirred for 30 minutes at 0 °C, and bromide (1.15 equiv.) was slowly added. The resulting mixture was stirred for 18 hours at room temperature. After complete conversion monitored by TLC, the mixture was quenched with saturated NH_4Cl solution and extracted with EtOAc. The combined organic phase was washed with water and brine, dried over Na_2SO_4, filtered and concentrated under reduced pressure. The resulting crude was purified by chromatography on silica gel (*n*Hex/EtOAc gradient).

GP-2: A mixture of sulfonyl amide (1 equiv.), cinnamyl chloride (1.3 equiv.) and K_2CO_3 (1.5 equiv.) in acetone (0.33 M) was refluxed for 18 hours. After complete conversion monitored by TLC, the mixture was quenched with saturated NH_4Cl solution and filtered on Celite®. Acetone was evaporated, and the mixture was diluted with EtOAc. The combined organic phase was washed with water and brine, dried over Na_2SO_4, filtered and concentrated under reduced pressure. The resulting crude was purified by chromatography on silica gel (nHex/EtOAc gradient).[5]

GP-3: To a stirred suspension of NaH (60% wt, 1.5 equiv.) in THF (0.13 M) at 0 °C, (1-methyl-1H-indol-2-yl)methanol (1 equiv.) was slowly added, and the mixture was stirred at the same temperature. After 2 hours, bromide (2 equiv.) was added, and the reaction was stirred for 18 hours at room temperature. After complete conversion monitored by TLC, the mixture was quenched with saturated NH_4Cl solution and extracted with EtOAc. The combined organic phase was washed with water and brine, dried over Na_2SO_4, filtered and concentrated under reduced pressure. The resulting crude was purified by chromatography on silica gel (nHex/EtOAc gradient).

GP-4: To a stirred suspension of NaH (60% wt, 1.1 equiv.) in DMF (0.32 M) at 0 °C, the unbiased alcohol (1 equiv.) was slowly added, and the mixture was stirred at the same temperature. After 30 minutes, bromide (1.1 equiv.) was added, and the reaction was stirred for 18 hours at room temperature. After complete conversion monitored by TLC, the mixture was quenched with saturated NH_4Cl solution and extracted with EtOAc. The combined organic phase was washed with water and brine, dried over Na_2SO_4, filtered and concentrated under reduced pressure. The resulting crude was purified by chromatography on silica gel (nHex/EtOAc gradient).

Characterization of substrates

2-((cinnamyloxy)methyl)furan 1a

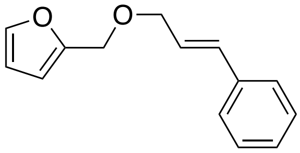

Following the **GP1** from the corresponding alcohol (197.0 mg, 2 mmol), **1a** was obtained in 77% as pale-yellow liquid (330.2 mg, 1.54 mmol). (hexane/ethyl acetate 8:2) ^{1}**H NMR** (400 MHz, $CDCl_3$) δ 7.48 – 7.40 (m, 3H), 7.38 – 7.32 (m, 2H), 7.31 – 7.25 (m, 1H), 6.67 (d, J = 15.9 Hz, 1H), 6.41 – 6.29 (m, 3H), 4.55 (s, 2H), 4.23 (dd, J = 6.2, 1.4 Hz, 2H). ^{13}**C NMR** (101 MHz, $CDCl_3$) δ 151.8, 142.9, 136.7, 133.0, 128.6, 127.8, 126.5, 125.7, 110.3, 109.4, 70.6, 63.9. **ESI-MS** calcd for $C_{14}H_{15}O_2$ $[M+H]^+$ 215.1067 found 215.1069.

2-(1-(cinnamyloxy)ethyl)furan 1b

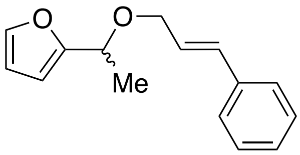

Following the **GP4** from the corresponding alcohol (134.2 mg, 1.2 mmol), **1b** was obtained in 55% as pale-yellow liquid (150.7 mg, 0.66mmol). (hexane/ethyl acetate 8:2) ^{1}**H NMR** (400 MHz, $CDCl_3$) δ 7.46 – 7.22 (m, 6H), 6.61 (d, J = 16.0 Hz, 1H), 6.40 – 6.24 (m, 3H), 4.61 (q, J = 6.6 Hz, 1H), 4.21 – 4.05 (m, 2H), 1.58 (d, J = 6.5 Hz, 3H). ^{13}**C NMR** (101 MHz, $CDCl_3$) δ 155.6, 142.2, 136.8, 132.4, 128.5, 127.6, 126.5, 126.2, 110.0, 107.0, 69.8, 69.0, 19.9. **ESI-HRMS** calcd for $C_{15}H_{17}O_2$ $[M+H]^+$ 229.1223 found 229.1224.

2-((cinnamyloxy)(phenyl)methyl)furan 1c

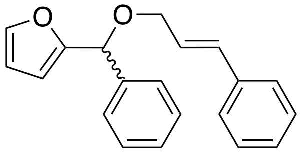

Following the **GP4** from the corresponding alcohol (209.04 mg, 1.2 mmol), **1c** was obtained in 78% as pale-yellow liquid. (271.8 mg, 0.94 mmol). (hexane/ethyl acetate 8:2) ^{1}**H NMR** (400 MHz, $CDCl_3$) δ 7.49 – 7.44 (m, 2H), 7.43 – 7.28 (m, 8H), 7.27 – 7.21 (m, 1H), 6.61 (d, J = 16.0 Hz, 1H), 6.38 – 6.28 (m, 2H), 6.16 – 6.13 (m, 1H), 5.52 (s, 1H), 4.24 – 4.17 (m, 2H). ^{13}**C NMR** (101 MHz, $CDCl_3$) δ 154.5, 142.8, 139.2, 136.7, 132.8, 128.6, 128.5, 128.1, 127.7, 127.3, 126.5, 125.8, 110.1, 108.6, 76.2, 69.5. **ESI-HRMS** calcd for $C_{20}H_{19}O_2$ $[M+H]^+$ 291.1380 found 291.1383.

2-(2-(cinnamyloxy)propan-2-yl)furan 1d

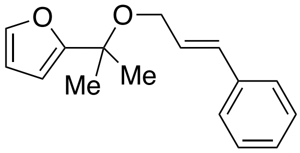

Following the **GP4** from the corresponding alcohol (126.2 mg, 1 mmol), **1d** was obtained in 52% as pale-yellow liquid (126.5 mg, 0.52 mmol). (hexane/ethyl acetate 8:2) ^{1}**H NMR** (400 MHz, $CDCl_3$) δ 7.45 – 7.18 (m, 6H), 6.55 (d, J = 15.9 Hz, 1H), 6.37 – 6.34 (m, 1H), 6.32 – 6.29 (m, 1H), 6.27 – 6.19 (m, 1H), 3.93 – 3.88 (m, 2H), 1.63 (s, 6H). ^{13}**C NMR** (101 MHz, CDCl3) δ 157.6, 142.0, 137.0, 131.5, 128.4, 127.4, 127.0, 126.5, 109.8, 106.9, 73.1, 64.3, 25.9. **ESI-HRMS** calcd for $C_{16}H_{19}O_2$ $[M+H]^+$ 243.3255 found 243.3253.

(E)-2-(((3-phenylallyl-2-d)oxy)methyl)furan 1e

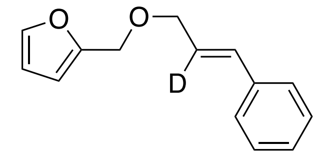

Following the **GP4** from the corresponding alcohol (132.2 mg, 1.35 mmol), **1e** was obtained in 51% as pale-yellow liquid (148.2 mg, 0.74 mmol). (hexane/ethyl acetate 8:2) **^{1}H NMR** (400 MHz, $CDCl_3$) δ 7.48 – 7.23 (m, 6H), 6.70 – 6.62 (m, 1H, 1H non-deuterated 1x), 6.41 – 6.28 (m, 2H, 1H non-deuterated 1x), 4.55 (s, 2H), 4.22 (s, 2H). **^{13}C NMR** (101 MHz, $CDCl_3$) δ 151.8, 142.9, 136.7, 128.6, 127.7, 126.5, 125.7 (non-deuterated 1x), 125.4 (t, J = 23.2 Hz), 110.3, 109.4, 70.6 (non-deuterated 1x), 70.5, 63.9. **ESI-HRMS** calcd for $C_{14}H_{14}DO_2$ $[M+H]^+$ 216.1129 found 216.1130.

(E)-2-(((3-(naphthalen-1-yl)allyl)oxy)methyl)furan 1f

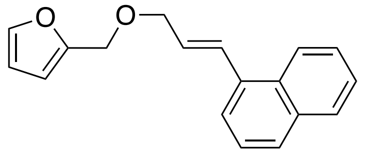

Following the **GP4** from the corresponding alcohol (66.8 mg, 0.68 mmol), **1f** was obtained in 64% as yellow viscous oil (114.8 mg, 0.43 mmol). (hexane/ethyl acetate 8:2) **^{1}H NMR** (400 MHz, $CDCl_3$) δ 8.16 – 8.11 (m, 1H), 7.89 – 7.84 (m, 1H), 7.79 (d, J = 8.2 Hz, 1H), 7.62 (d, J = 7.1 Hz, 1H), 7.56 – 7.37 (m, 5H), 6.42 – 6.30 (m, 3H), 4.60 (s, 2H), 4.32 (dd, J = 6.0, 1.5 Hz, 2H). **^{13}C NMR** (101 MHz, $CDCl_3$) δ 151.8, 142.9, 134.5, 133.6, 131.2, 130.0, 129.0, 128.6, 128.1, 126.1, 125.8, 125.6, 124.0, 123.8, 110.4, 109.5, 70.8, 64.0. **ESI-HRMS** calcd for $C_{18}H_{17}O_2$ $[M+H]^+$ 265.1223 found 265.1221.

(E)-2-(((3-(2-methoxyphenyl)allyl)oxy)methyl)furan 1g

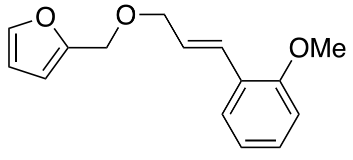

Following the **GP4** from the corresponding alcohol (66.8 mg, 0.68 mmol), **1g** was obtained in 57% as pale-yellow viscous oil (139.1 mg, 0.57 mmol). (hexane/ethyl acetate 8:2) **^{1}H NMR** (400 MHz, $CDCl_3$) δ 7.48 – 7.37 (m, 2H), 7.29 – 7.20 (m, 1H), 6.99 – 6.85 (m, 3H), 6.38 – 6.27 (m, 3H), 4.51 (s, 2H), 4.23 – 4.18 (m, 2H), 3.85 (s, 3H). **^{13}C NMR** (101 MHz, $CDCl_3$) δ 156.7, 151.8, 142.7, 128.8, 128.1, 127.0, 126.2, 125.6, 120.6, 110.8, 110.2, 109.3, 71.1, 63.6, 55.4. **ESI-HRMS** calcd for $C_{15}H_{17}O_3$ $[M+H]^+$ 245.1172 found 245.1171.

(E)-2-(((3-(2-chlorophenyl)allyl)oxy)methyl)furan 1h

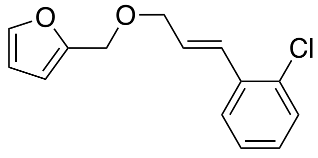

Following the **GP4** from the corresponding alcohol (84.4 mg, 0.86 mmol), **1h** was obtained in 78% as pale orange oil (166.8 mg, 0.67 mmol). (hexane/ethyl acetate 8:2) **^{1}H NMR** (400 MHz, $CDCl_3$) δ 7.54 (dd, J = 7.5, 1.9 Hz, 1H), 7.45 – 7.41 (m, 1H), 7.35 (dd, J = 7.7, 1.6 Hz, 1H), 7.25 – 7.15 (m, 2H), 7.02 (d, J = 15.9 Hz, 1H), 6.38 – 6.34 (m, 2H), 6.28 (dt, J = 15.9, 6.1 Hz, 1H), 4.53 (s, 2H), 4.23 (dd, J = 6.1, 1.5 Hz, 2H). **^{13}C NMR** (101 MHz, $CDCl_3$) δ 151.6, 142.9, 134.8, 133.1, 129.7, 129.1, 128.7, 128.6, 127.0, 126.9, 110.3, 109.5, 70.5, 63.9. **ESI-HRMS** calcd for $C_{14}H_{14}ClO_2$ $[M+H]^+$ 249.0677 found 249.0678.

(E)-2-(((3-(3-bromophenyl)allyl)oxy)methyl)furan 1i

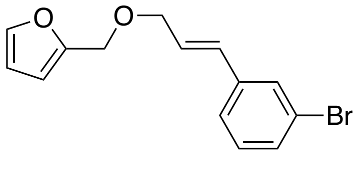

Following the **GP4** from the corresponding alcohol (98.1 mg, 1 mmol), **1i** was obtained in 66% as pale yellow oil (193.7 mg, 0.66 mmol). (hexane/ethyl acetate 8:2) **^{1}H NMR** (400 MHz, $CDCl_3$) δ 7.54 – 7.51 (m, 1H), 7.43 (s, 1H), 7.39 – 7.33 (m, 1H), 7.32 – 7.27 (m, 1H), 7.18 (t, J = 7.8 Hz, 1H), 6.56 (d, J = 16.0 Hz, 1H), 6.38 – 6.24 (m, 3H), 4.51 (s, 2H), 4.22 – 4.15 (m, 2H). **^{13}C NMR** (101 MHz, CDC13) δ 151.6, 142.9, 138.9, 131.1, 130.6, 130.1, 129.4, 127.4, 125.1, 122.8, 110.3, 109.5, 70.2, 64.1. **ESI-HRMS** calcd for $C_{14}H_{14}BrO_2$ $[M+H]^+$ 293.0172 found 293.0175.

(E)-2-(((3-(4-fluorophenyl)allyl)oxy)methyl)furan 1j

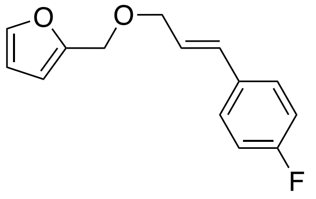

Following the **GP4** from the corresponding alcohol (91.7 mg, 0.93 mmol), **1j** was obtained in 70% as pale yellow oil (151.2 mg, 0.65 mmol). (hexane/ethyl acetate 8:2) **^{1}H NMR** (400 MHz, $CDCl_3$) δ 7.45 – 7.41 (m, 1H), 7.38 – 7.32 (m, 2H), 7.04 – 6.97 (m, 2H), 6.59 (d, J = 15.9 Hz, 1H), 6.38 – 6.33 (m, 2H), 6.21 (dt, J = 15.9, 6.1 Hz, 1H), 4.51 (s, 2H), 4.20 – 4.15 (m, 2H). **^{13}C NMR** (101 MHz, $CDCl_3$) δ 162.4 (d, J = 246.8 Hz), 151.7, 142.9, 132.8 (d, J = 3.4 Hz), 131.7, 128.0 (d, J = 8.0 Hz), 125.4 (d, J = 2.3 Hz), 115.5 (d, J = 21.7 Hz), 110.3, 109.4, 70.5, 64.0. **^{19}F NMR** (565 MHz, $CDCl_3$) δ -114.16 – -114.22 (m). **ESI-HRMS** calcd for $C_{14}H_{14}FO_2$ $[M+H]^+$ 233.0972 found 233.0976.

(E)-2-(((3-(4-(trifluoromethyl)phenyl)allyl)oxy)methyl)furan 1k

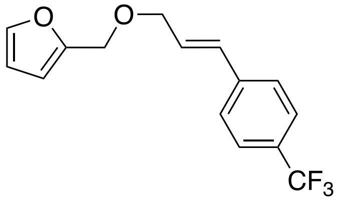

Following the **GP4** from the corresponding alcohol (128.9 mg, 1.3 mmol), **1k** was obtained in 42% as pale yellow oil (154.1 mg, 0.54 mmol). (hexane/ethyl acetate 8:2) **^{1}H NMR** (400 MHz, $CDCl_3$) δ 7.61 – 7.57 (m, 2H), 7.51 – 7.45 (m, 3H), 6.69 (d, J = 15.9 Hz, 1H), 6.45 – 6.37 (m, 3H), 4.55 (s, 2H), 4.24 (dd, J = 5.8, 1.6 Hz, 2H). **^{13}C NMR** (101 MHz, $CDCl_3$) δ 151.6, 143.0, 140.2, 131.0, 129.5 (q, J = 32.3 Hz), 128.6, 126.6, 125.5 (q, J = 3.9 Hz), 124.2 (q, J = 277.6 Hz), 110.3, 109.5, 70.2, 64.2. **^{19}F NMR** (565 MHz, $CDCl_3$) δ -62.4. **ESI-HRMS** calcd for $C_{15}H_{14}F_3O_2$ $[M+H]^+$ 283.0940 found 283.0943.

N-cinnamyl-N-(furan-2-ylmethyl)-4-methylbenzenesulfonamide 1l

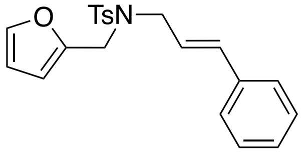

Following the **GP2** from the corresponding tosyl amide (251.3 mg, 1 mmol), **1l** was obtained in 83% as pale yellow oil (305.2 mg, 0.83 mmol). (hexane/ethyl acetate 8:2) **^{1}H NMR** (400 MHz, $CDCl_3$) δ 7.72 (d, J = 8.3 Hz, 2H), 7.36 – 7.24 (m, 8H), 6.46 (d, J = 15.9 Hz, 1H), 6.31 – 6.28 (m, 1H), 6.21 – 6.18 (m, 1H), 6.03 – 5.94 (m, 1H), 4.46 (s, 2H), 3.96 (d, J = 6.7 Hz, 2H), 2.44 (s, 3H). **^{13}C NMR** (101 MHz, $CDCl_3$) δ 149.8, 143.2, 142.5, 137.3, 136.3, 134.2, 129.6, 128.6, 127.9, 127.3, 126.5, 123.7, 110.4, 109.5, 49.3, 42.8, 21.5. **ESI-HRMS** calcd for $C_{21}H_{22}NaNO_3S$ $[M+Na]^+$ 390.1134 found 390.113.

2,5-bis((cinnamyloxy)methyl)furan 1m

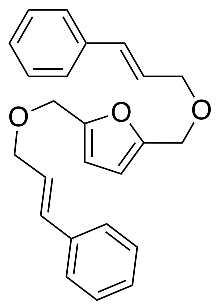

Following **GP4** from the corresponding alcohol (385.3mg, 3mmol), **1m** was obtained in 67% as a yellow solid (689mg, 2mmol). (hexane/ethyl acetate 8:2) **^{1}H NMR** (400 MHz, $CDCl_3$) δ = 7.44 – 7.22 (m, 10H), 6.65 (d, *J*=16.0, 2H), 6.40 – 6.21 (m, 4H), 4.53 (s, 4H), 4.23 (dd, *J*=6.1, 1.4, 4H). **^{13}C NMR** (101 MHz, $CDCl_3$) δ 152.1, 136.6, 133.0, 128.6, 127.8, 126.6, 125.7, 110.1, 70.7, 64.0. **ESI-HRMS** calcd for $C_{24}H_{24}O_3$ $[M+H]^+$ 360.1725 found 360.1724.

2-((allyloxy)methyl)-5-((cinnamyloxy)methyl)furan 1n

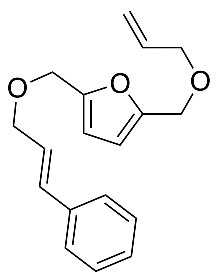

Following the **GP4** from the corresponding alchol (169.8 mg, 1.1mmol), **1n** was obtained in 50% as yellow oil (157mg, 0.55 mmol). (hexane/ethyl acetate 8:2) **^{1}H NMR** (600 MHz, $CDCl_3$) δ = 7.40 – 7.21 (m, 5H), 6.61 (d, J=15.9, 1H), 6.33 – 6.24 (m, 3H), 5.98 – 5.85 (m, 1H), 5.33 – 5.26 (m, 1H), 5.20 (d, J=10.4, 1H), 4.49 (s, 2H), 4.44 (s, 2H), 4.19 (d, J=6.2, 2H), 4.02 (d, J=5.8, 2H). **^{13}C NMR** (101 MHz, $CDCl_3$) δ = 152.2, 152.2, 136.8, 134.5, 133.0, 128.6, 127.8, 126.6, 125.8, 117.6, 110.2, 110.1, 71.2, 70.8, 64.1. **ESI-HRMS** calcd for $C_{18}H_{20}O_3$ $[M+H]^+$ 284.1412 found 284.1413.

2-((cinnamyloxy)methyl)thiophene 1o

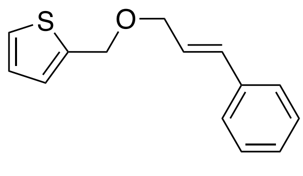

Following the **GP4** from the corresponding tosyl amide (114.2 mg, 1 mmol), **1o** was obtained in 72% as pale yellow oil (165.8 mg, 0.72 mmol). (hexane/ethyl acetate 8:2) **^{1}H NMR** (400 MHz, $CDCl_3$) δ 7.46 – 7.40 (m, 2H), 7.39 – 7.32 (m, 3H), 7.31 – 7.25 (m, 1H), 7.08 – 7.05 (m, 1H), 7.04 – 7.01 (m, 1H), 6.67 (d, J = 15.9 Hz, 1H), 6.35 (dt, J = 15.9, 6.1 Hz, 1H), 4.77 (d, J = 0.8 Hz, 2H), 4.25 (dd, J = 6.1, 1.5 Hz, 2H). **^{13}C NMR** (101 MHz, $CDCl_3$) δ 141.0, 136.7, 132.9, 128.6, 127.8, 126.7, 126.5, 125.9, 125.8, 70.4, 66.5. **ESI-HRMS** calcd for $C_{14}H_{15}OS$ $[M+H]^+$ 230.0765 found 230.0767.

N-cinnamyl-4-methyl-N-((1-tosyl-1H-pyrrol-2-yl)methyl)benzenesulfonamide 1p

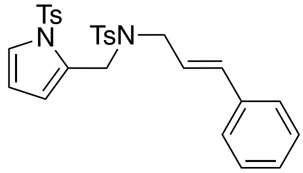

Following the **GP4** from the corresponding tosyl amide (635.5 mg, 1.57 mmol), **1p** was obtained in 70% as pale yellow oil (572.0 mg, 1.09 mmol). (hexane/ethyl acetate 8:2) **^{1}H NMR** (400 MHz, $CDCl_3$) δ 7.68 (d, J = 8.3 Hz, 2H), 7.60 (d, J = 8.4 Hz, 2H), 7.32 – 7.20 (m, 6H), 7.16 – 7.06 (m, 4H), 6.26 – 6.11 (m, 3H), 5.75 – 5.65 (m, 1H), 4.55 (s, 2H), 3.90 – 3.84 (m, 2H), 2.43 (s, 3H), 2.31 (s, 3H). **^{13}C NMR** (101 MHz, $CDCl_3$) δ 145.1, 143.5, 136.9, 136.3, 135.9, 134.0, 130.0, 129.8, 128.5, 127.9, 127.4, 126.7, 126.5, 123.40, 123.35, 115.0, 111.9, 50.0, 44.2, 21.6, 21.5. **ESI-HRMS** calcd for $C_{28}H_{29}N_2O_4S_2$ $[M+H]^+$ 521.1563 found 521.1561.

2-((cinnamyloxy)methyl)benzofuran 3a

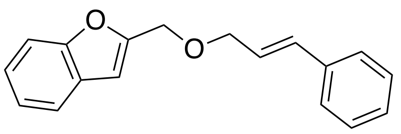

Following the **GP4** from the corresponding alcohol (133.3 mg, 0.9 mmol), **3a** was obtained in 95% as pale yellow solid (226.3 mg, 0.86 mmol). (hexane/ethyl acetate 8:2) **^{1}H NMR** (400 MHz, $CDCl_3$) δ 7.60 (d, J = 7.5 Hz, 1H), 7.53 (d, J = 8.1 Hz, 1H), 7.46 – 7.40 (m, 2H), 7.39 – 7.22 (m, 5H), 6.77 – 6.65 (m, 2H), 6.36 (dt, J = 15.9, 6.1 Hz, 1H), 4.71 (s, 2H), 4.33 – 4.27 (m, 2H). **^{13}C NMR** (101 MHz, $CDCl_3$) δ 155.3, 154.3, 136.6, 133.3, 128.6, 128.1, 127.8, 126.6, 125.5, 124.4, 122.8, 121.1, 111.4, 105.8, 71.0, 64.4. **ESI-HRMS** calcd for $C_{18}H_{17}O_2$ $[M+H]^+$ 265.1223, found 265.1221.

N-(benzofuran-2-ylmethyl)-N-cinnamyl-4-methylbenzenesulfonamide 3b

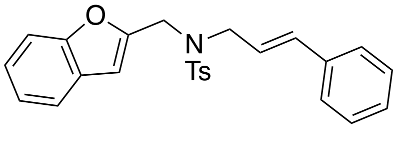

Following the **GP4** from the corresponding tosyl amide (473.4 mg, 1.57 mmol), **3b** was obtained in 65% as pale yellow solid (426.1 mg, 1.02 mmol). (hexane/ethyl acetate 8:2) **^{1}H NMR** (400 MHz, $CDCl_3$) δ 7.74 – 7.70 (m, 2H), 7.51 – 7.47 (m, 1H), 7.33 – 7.17 (m, 10H), 6.56 – 6.54 (m, 1H), 6.47 (d, J = 15.9 Hz, 1H), 6.03 (dt, J = 15.8, 6.7 Hz, 1H), 4.59 (s, 2H), 4.07 – 4.02 (m, 2H), 2.38 (s, 3H). **^{13}C NMR** (101 MHz, $CDCl_3$) δ 154.9, 152.5, 143.3, 137.2, 136.1, 134.6, 129.5, 128.6, 128.0, 127.9, 127.4, 126.5, 124.3, 123.6, 122.8, 121.0, 111.1, 106.2, 49.6, 43.3, 21.5. **ESI-HRMS** calcd for $C_{25}H_{24}NO_3S$ $[M+H]^+$ 418.1471, found 418.1472.

2-((cinnamyloxy)methyl)benzo[b]thiophene 3c

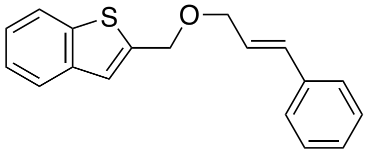

Following the **GP4** from the corresponding alcohol (410.55 mg, 2.5 mmol), **3c** was obtained in 90% as pale-yellow oil (630.9 mg, 2.25 mmol). (hexane/ethyl acetate 8:2) **^{1}H NMR** (400 MHz, $CDCl_3$) δ 7.89 – 7.83 (m, 1H), 7.80 – 7.74 (m, 1H), 7.47 – 7.24 (m, 8H), 6.68 (d, J = 15.9 Hz, 1H), 6.36 (dt, J = 15.9, 6.1 Hz, 1H), 4.86 (d, J = 1.0 Hz, 2H), 4.28 (dd, J = 6.1, 1.5 Hz, 2H). **^{13}C NMR** (101 MHz, $CDCl_3$) δ 142.2, 140.3, 139.5, 136.6, 133.1, 128.6, 127.8, 126.6, 125.6, 124.29, 124.27, 123.5, 122.6, 122.5, 70.6, 67.2. **ESI-HRMS** calcd for $C_{18}H_{16N}NaOS$ $[M+Na]^+$ 303.0814, found 303.0813.

(E)-2-(((3-(p-tolyl)allyl)oxy)methyl)benzo[b]thiophene 3d

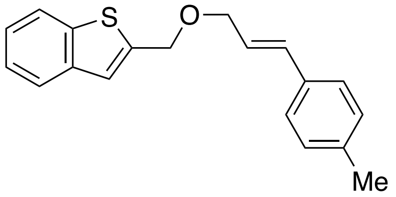

Following the **GP4** from the corresponding alcohol (121.5 mg, 0.74 mmol), **3d** was obtained in 67% as pale-yellow oil (146.0 mg, 0.47 mmol). (hexane/ethyl acetate 8:2) **^{1}H NMR** (400 MHz, $CDCl_3$) δ 7.86 – 7.82 (m, 1H), 7.77 – 7.72 (m, 1H), 7.38 – 7.28 (m, 4H), 7.24 (brs, 1H), 7.15 (d, J = 7.9 Hz, 2H), 6.63 (d, J = 16.0 Hz, 1H), 6.29 (dt, J = 15.9, 6.2 Hz, 1H), 4.83 (s, 2H), 4.25 (dd, J = 6.2, 1.4 Hz, 2H), 2.36 (s, 3H). **^{13}C NMR** (101 MHz, $CDCl_3$) δ 142.3, 140.3, 139.5, 137.7, 133.8, 133.1, 129.3, 126.5, 124.5, 124.3, 123.5, 122.6, 122.5, 70.7, 67.1, 21.3. **ESI-HRMS** calcd for $C_{19}H_{19}OS$ $[M+H]^+$ 295.1151, found 295.1153.

3-((cinnamyloxy)methyl)benzo[b]thiophene 3e

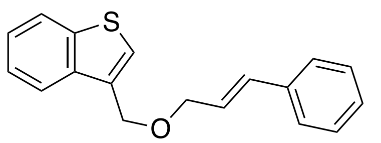

Following the **GP4** from the corresponding alcohol (328.4 mg, 2 mmol), **3e** was obtained in 78% as pale yellow oil (417.4 mg, 1.56 mmol). (hexane/ethyl acetate 8:2) **^{1}H NMR** (400 MHz, $CDCl_3$) δ 7.96 – 7.88 (m, 2H), 7.49 – 7.23 (m, 8H), 6.68 (d, J = 16.0 Hz, 1H), 6.43 – 6.32 (m, 1H), 4.86 (s, 2H), 4.31 – 4.25 (m, 2H). **^{13}C NMR** (101 MHz, $CDCl_3$) δ 140.7, 138.2, 136.7, 133.3, 132.8, 128.6, 127.8, 126.5, 125.9, 124.8, 124.5, 124.2, 122.8, 122.3, 70.8, 66.4. **ESI-HRMS** calcd for $C_{18}H_{17}OS$ $[M+H]^+$ 281.0995, found 281.0994.

(E)-3-(((3-(3-ethynylphenyl)allyl)oxy)methyl)benzo[b]thiophene 3f

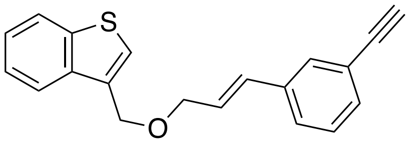

Following the **GP4** from the corresponding alcohol (228.3 mg, 1.39 mmol), **3f** was obtained in 69% as pale yellow oil (287.9 mg, 0.98 mmol). (hexane/ethyl acetate 8:2) **^{1}H NMR** (400 MHz, $CDCl_3$) δ 7.95 – 7.87 (m, 2H), 7.55 – 7.52 (m, 1H), 7.46 – 7.36 (m, 5H), 7.33 – 7.26 (m, 1H), 6.62 (d, J = 16.0 Hz, 1H), 6.37 (dt, J = 16.0, 5.9 Hz, 1H), 4.88 – 4.82 (m, 2H), 4.26 (dd, J = 5.9, 1.5 Hz, 2H), 3.10 (s, 1H). **^{13}C NMR** (101 MHz, $CDCl_3$) δ 140.7, 138.2, 136.9, 133.2, 131.5, 131.3, 130.2, 128.6, 127.1, 126.9, 124.8, 124.6, 124.2, 122.8, 122.4, 122.3, 83.5, 70.5, 66.6. **ESI-HRMS** calcd for $C_{20}H_{17}OS$ $[M+H]^+$ 305.0955, found 305.0954.

2-((cinnamyloxy)methyl)-1-methyl-1H-indole 3g

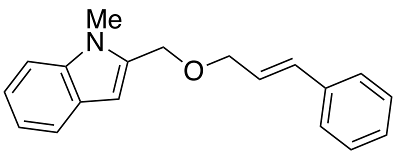

Following the **GP4** from the corresponding tosyl amide (161.2 mg, 1 mmol), **3g** was obtained in 75% as pale yellow solid (208.4 mg, 0.75 mmol). (hexane/ethyl acetate 8:2) **^{1}H NMR** (400 MHz, $CDCl_3$) δ 7.63 (d, J = 7.8 Hz, 1H), 7.43 – 7.23 (m, 7H), 7.17 – 7.10 (m, 1H), 6.65 (d, J = 15.9 Hz, 1H), 6.53 (s, 1H), 6.32 (dt, J = 16.0, 6.1 Hz, 1H), 4.76 (s, 2H), 4.19 (dd, J = 6.1, 1.4 Hz, 2H), 3.84 (s, 3H). **^{13}C NMR** (101 MHz, $CDCl_3$) δ 138.2, 136.7, 135.7, 132.9, 128.6, 127.8, 127.2, 126.5, 125.7, 121.9, 120.8, 119.5, 109.2, 103.1, 69.8, 64.0, 30.0. **ESI-HRMS** calcd for $C_{19}H_{20}NO$ $[M+H]^+$ 278.1539, found 278.1538.

(E)-1-methyl-2-(((3-(naphthalen-1-yl)allyl)oxy)methyl)-1H-indole 3h

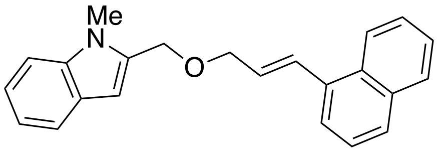

Following the **GP4** from the corresponding alcohol (178.6 mg, 1.11 mmol), **3h** was obtained in 72% as pale yellow solid (261.7 mg, 0.74 mmol). (hexane/ethyl acetate 8:2) **^{1}H NMR** (400 MHz, CDCl3) δ 8.14 – 8.08 (m, 1H), 7.89 – 7.84 (m, 1H), 7.80 (d, J = 8.2 Hz, 1H), 7.66 – 7.57 (m, 2H), 7.55 – 7.32 (m, 5H), 7.29 – 7.23 (m, 1H), 7.17 – 7.10 (m, 1H), 6.56 (s, 1H), 6.34 (dt, J = 15.6, 6.0 Hz, 1H), 4.81 (s, 2H), 4.28 (dd, J = 6.0, 1.6 Hz, 2H), 3.85 (s, 3H). **^{13}C NMR** (101 MHz, $CDCl_3$) δ 138.2, 135.8, 134.4, 133.6, 131.2, 130.0, 129.0, 128.6, 128.1, 127.2, 126.1, 125.8, 125.6, 124.0, 123.8, 121.9, 120.8, 119.5, 109.2, 103.1, 70.0, 64.1, 30.0. **ESI-HRMS** calcd for $C_{23}H_{21}NaNO$ $[M+Na]^+$ 350.1515 found 350.1516.

Optimization experiments

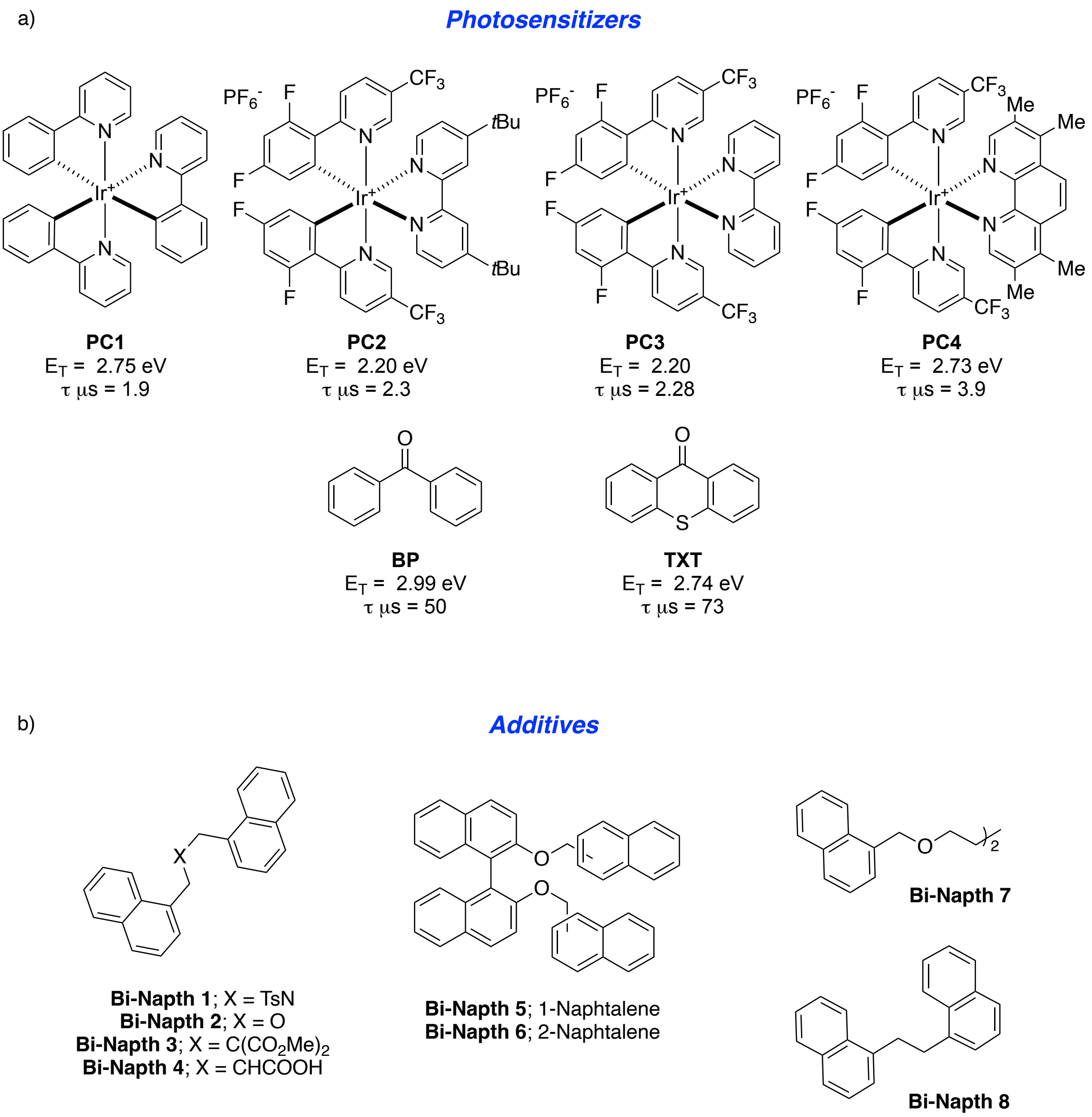

Figure S1 a) Structures and photophysical properties of photosensitizers. Data related to the photosensitizer can be found respectively at reference [6] for **PC1**, **PC2** and **PC3**, reference [7] for **PC4**, and reference [8],[9] for **TXT** and **PC**. b) Structure of the additive used in this study

Influence of the additive

Table S1

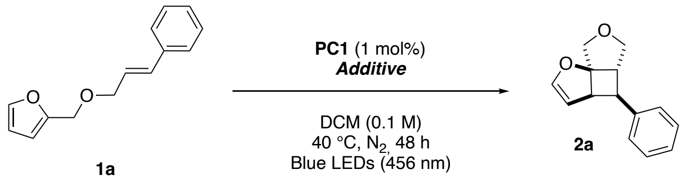

Entry[a]	Additive	Yields of 2+2	4+2	Z
**1[b]**	Napht (5 equiv.)	44%	14%	-
**2**	Napht (20 equiv.)	63%	-	-
**3**	-	14%	2%	46%
**4**	BiNapht 2 (1 equiv.)	33%	9%	16%
**5**	BiNapht 3 (1 equiv.)	31%	8%	8%
**6**	BiNapht 7 (1 equiv.)	32%	8%	23%
**7**	BiNapht 8 (1 equiv.)	50%	14%	3%
**8**	BiNapht 5 (1 equiv.)	16%	6%	52%
**9**	BiNapht 1 (30 mol%)	38%	9%	17%
**10**	BiNapht 2 (30 mol%)	43%	10%	10%
**11**	BiNapht 3(30 mol%)	41%	10%	7%
**12**	BiNapht 4 (30 mol%)	14%	4%	49%
**13**	BiNapht 6 (30 mol%)	49%	11%	13%
**14**	BiNapht 8 (30 mol%)	45%	14%	12%

[a]**Conditions**: unless otherwise specify **1a** (0.15 mmol, 1 equiv.), **PC1** (1 mol%) in DCM (0.1 M) in 5 mm-NMR tube under N_2, irradiated with Blue LEDs (λ = 456 nm) for 48 hours, yields were determined using 1,3,5 trimethoxybenzene as internal standard. [b] irradiated for 40 hours

Table S2

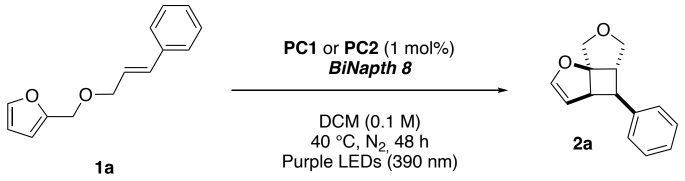

Entry[a]	PC	Additive BiNapht 8 amount	Yields of 2+2	4+2	Z
**15**	**PC1**	30 mol%	40%	-	23%
**16**	**PC2**	-	50%	12%	-
**17**	**PC2**	1 equiv.	66%	-	-
**18[b]**	**PC2**	1 equiv.	76%	-	-
**19**	**PC2**	10 mol%	81%	-	-
**20**	**PC2**	50 mol%	74%	-	-
**21[c]**	**PC2**	30 mol%	35%	12%	23%
**22**	**PC2**	30 mol%	85%	-	-

[a]**Conditions**: unless otherwise specify **1a** (0.15 mmol, 1 equiv.), **PC** (1 mol%) and **BiNapht 8** (amount from the table) in DCM (0.1 M) in 5 mm-NMR tube under N_2, irradiated with Purple LEDs (λ = 390 nm) for 48 hours, yields were determined using 1,3,5 trimethoxybenzene as internal standard; [b] 2 mol% of **PC2** was used; [c] irradiated for 24 hours

Influence of the Photocatalysts

Table S3

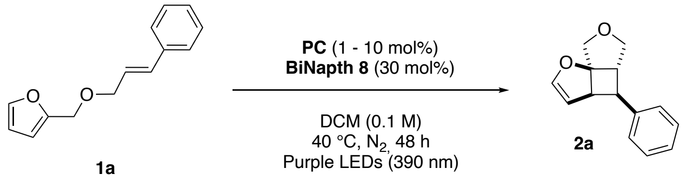

Entry[a]	PC	Yields of 2+2	Yields of 4+2	Yields of Z
**23**	**TXT** (10 mol%)	78%	-	-
**24**	**BP** (10 mol%)	-	-	56%
**25**	**PC1**	40%	-	23%
**26**	**PC2**	85%	-	-
**27**	**PC3**	65%	9%	9%
**28**	**PC4**	77%	-	-
**29**	w/o PC or light		SM	

[a]**Conditions**: unless otherwise specify **1a** (0.15 mmol, 1 equiv.), **PC** (1 mol%) in DCM (0.1 M), **BiNapth 8** (30 mol%) in 5 mm-NMR tube under N_2, irradiated with Purple LEDs (λ = 390 nm) for 48 hours, yields were determined using 1,3,5 trimethoxybenzene as internal standard. Note: TXT = Thioxanthone; BP = Benzophenone

Influence of the Solvent

Table S4

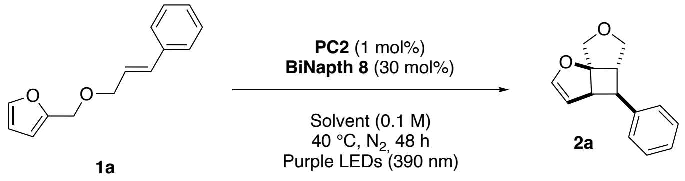

Entry[a]	Solvents	Yields of 2+2	Yields of 4+2	Yields of Z
**30**	MeCN/DCM 3:1	61%	-	-
**31**	Toluene	70%	3%	9%
**32**	EtOAc	83%	-	-
**33**	DCE	82%	-	-
**34**	DCM (0.05M)	80%	-	-
**35**	DCM (0.2M)	51%	-	26%

[a]**Conditions**: unless otherwise specify **1a** (0.15 mmol, 1 equiv.), **PC2** (1 mol%), **BiNapth 8** (30 mol%) in 5 mm-NMR tube under N_2, irradiated with Purple LEDs (λ = 390 nm) for 48 hours, yields were determined using 1,3,5 trimethoxybenzene as internal standard.

General procedures for the [2+2] dearomatization

GP1-P

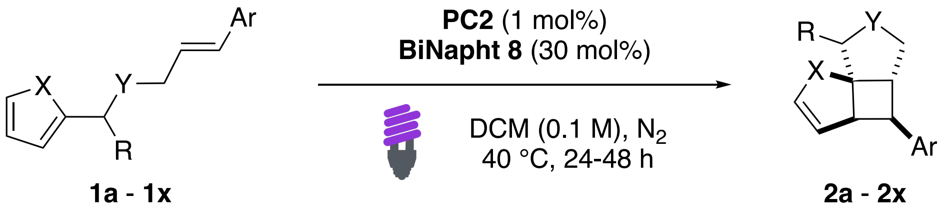

To a vial charged with the desired substrate **1a-1x** (0.15 mmol, 1 equiv.), **BiNapth 8** (13 mg, 0.3 mmol, 30 mol%) and **PC2** (1 mol%), dry and degassed DCM (0.1 M) was added through a syringe. The solution was transferred into an NMR tube capped with a rubber septum, degassed by freeze-pump-thaw (2 times) and the tube was irradiated with purple household LEDs strip for 24 – 48 hours. Conversion was monitored by TLC and the mixture was then concentrated in vacuo. The residue was purified by chromatography on silica gel (nHex /EtOAc, under gradient).

GP2-P

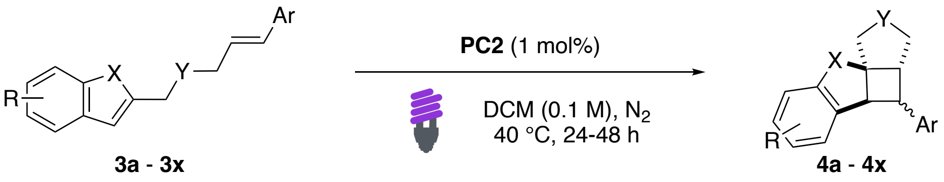

To a vial charged with substrate **3a** – **3x** (0.15 mmol, 1 equiv.) and **PC2** (1 mol%), dry and degassed DCM (0.1 M) was added through a syringe. The solution was transferred into an NMR tube capped with a rubber septum, degassed by freeze-pump-thaw (2 times) and the tube was irradiated with purple household LEDs strip for 16 hours. Conversion was monitored by TLC and the mixture was then concentrated in vacuo. The residue was purified by chromatography on silica gel (*n*Hex/EtOAc, under gradient).

GP3-P

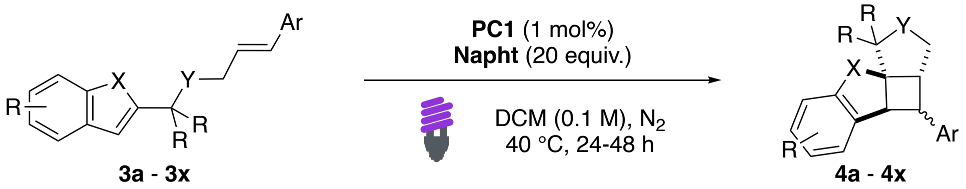

To a vial charged with substrate **4g** or **4h** (0.15 mmol, 1 equiv.), naphthalene (385.5 mg, 3 mmol, 20 equiv.) and Ir(ppy)$_3$ (1 mg, 1 mol%), dry and degassed DCM (0.1 M) was added through a syringe. The solution was transferred into an NMR tube capped with a rubber septum, degassed by freeze-pump-thaw (2 times) and the tube was irradiated with blue household LEDs strip for 24 – 48 hours. Conversion was monitored by TLC and the mixture was then concentrated in vacuo. The residue was purified by chromatography on silica gel (nHex/EtOAc, under gradient).

Products Characterization

4-phenyl-3a,4,4a,5-tetrahydro-7H-cyclobuta[1,2-b:1,4-c']difuran 2a

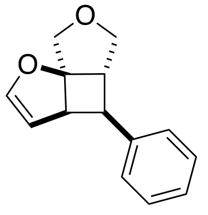

Following the **GP1-P** using **1a** (31.9 mg, 0.15 mmol, 342.8 mg, 1.6 mmol), **2a** was obtained in 82% yield as white solid (26.2 mg). The reaction performed on 1.6 mmol scale of **1a**, required 120 hours of irradiation to completely consume the starting material. **2a** was obtained in 65% (228.8 mg). (hexane/ethyl acetate 9:1) **^{1}H NMR** (400 MHz, $CDCl_3$) δ 7.41 – 7.21 (m, 5H), 6.42 – 6.36 (m, 1H), 4.91 – 4.85 (m, 1H), 4.25 (d, J = 9.9 Hz, 1H), 3.99 – 3.89 (m, 2H), 3.86 – 3.77 (m, 2H), 3.56 (t, J = 7.3 Hz, 1H), 3.41 – 3.35 (m, 1H). **^{13}C NMR** (101 MHz, $CDCl_3$) δ 147.3, 140.1, 128.2, 127.6, 126.4, 104.6, 91.6, 72.9, 71.3, 51.0, 51.0, 44.0. **ESI-HRMS** calcd for $C_{14}H_{15}O_2$ $[M+H]^+$ 215.1067 found 215.1070.

7-methyl-4-phenyl-3a,4,4a,5-tetrahydro-7H-cyclobuta[1,2-b:1,4-c']difuran 2b

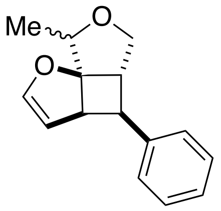

Following the **GP1-P** using **1b** (34.4 mg, 0.15 mmol), **2b** was obtained in 55% yield as white solid as a mixture of diastereomers (58:42) (18.9 mg). The reaction required 36 hours of irradiation to completely consume the starting material. (hexane/ethyl acetate 9:1) **^{1}H NMR** (400 MHz, $CDCl_3$) δ 7.44 – 7.21 (m, 6H Dia1, 6H Dia2), 6.39 (td, *J*=3.1, 1.6, 1H Dia1), 6.32 (d, *J*=2.8, 1H Dia2), 4.84 (m, 1H Dia1), 4.68 – 4.58 (m, 1H Dia2), 4.32 – 4.19 (m, 1H Dia2), 4.12 (dt, *J*=9.5, 4.5, 2H Dia2), 4.02 – 3.93 (m, 1H Dia1), 3.91 – 3.71 (m, 2H Dia1), 3.65 (dd, *J*=11.6, 4.6, 1H Dia2), 3.51 (dt, *J*=32.3, 7.3, 1H Dia1), 3.39 – 3.32 (m, 1H Dia1), 3.23 – 3.17 (m, 1H Dia2), 1.42 (d, *J*=6.3, 3H Dia2), 1.28 (d, *J*=5.1, 3H Dia1). **^{13}C NMR** (101 MHz, $CDCl_3$) δ 147.3, 147.3, 140.2, 140,2, 128.5, 128.2, 128.1, 127.6, 127.6, 126.3, 126.3,125.7, 104.3, 104.0, 93.9, 92.8, 77.6, 75.1, 71.5, 70, 51.1, 51.0, 50.9, 46.2, 44.8, 44.2. **ESI-HRMS** calcd for $C_{15}H_{17}O_2$ $[M+H]^+$ 229.1223 found 229.1225.

4,7-diphenyl-3a,4,4a,5-tetrahydro-7H-cyclobuta[1,2-b:1,4-c']difuran 2c

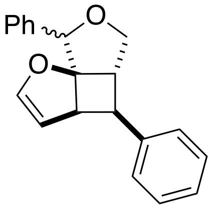

Following the **GP1-P** using **1c** (43.4 mg, 0.15 mmol), **2c** was obtained as a mixture of diastereomers (57:43) in a total yield of 70%. The reaction required 40 hours of irradiation to completely consume the starting material. **2c'** (17.3 mg, 40% yield); (hexane/ethyl acetate 9:1) **^{1}H NMR** (400 MHz, $CDCl_3$) δ 7.53 – 7.18 (m, 10H), 6.40 – 6.36 (m, 1H), 5.02 (s, 1H), 4.76 – 4.70 (m, 1H), 4.19 (dd, J = 9.7, 4.8 Hz, 1H), 4.07 (d, J = 9.7 Hz, 1H), 3.56 – 3.45 (m, 2H), 3.39 – 3.33 (m, 1H). **^{13}C NMR** (101 MHz, $CDCl_3$) δ 147.0, 140.1, 137.8, 128.4, 128.2, 127.5, 126.3, 125.7, 104.6, 94.4, 80.6, 71.7, 51.2, 47.4, 43.9. **ESI-HRMS** calcd for $C_{20}H_{19}O_2$ $[M+H]^+$ 291.1380 found 291.1378. **2c''** (12.7 mg, 30% yield): **^{1}H NMR** (400 MHz, $CDCl_3$) δ 7.43 – 7.24 (m, 10H), 6.33 (dd, *J*=2.9, 1.6, 1H), 5.28 (s, 1H), 4.86 (t, J = 2.8 Hz, 1H), 4.44 (dd, J = 9.5, 6.0 Hz, 1H), 4.05 – 4.00 (m, 1H), 3.95 (dd, J = 9.6, 1.9 Hz, 1H), 3.71 (t, J = 7.3 Hz, 1H), 3.59 – 3.52 (m, 1H). **^{13}C NMR** (101 MHz, $CDCl_3$) δ 147.6, 139.9, 137.7, 128.2, 128.1, 127.7, 127.6, 127.3, 126.5, 103.7, 92.7, 83.8, 72.3, 51.9, 51.6, 45.4. **ESI-HRMS** calcd for $C_{20}H_{19}O_2$ $[M+H]^+$ 291.1380 found 291.1381.

7,7-dimethyl-4-phenyl-3a,4,4a,5-tetrahydro-7H-cyclobuta[1,2-b:1,4-c']difuran 2d

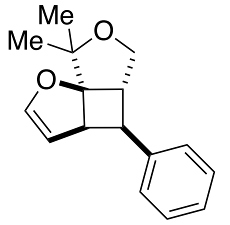

Following the **GP3-P** using **1d** (36.5 mg, 0.15 mmol), **2d** was obtained in 59% (21.6 mg). The reaction required 38 hours of irradiation to completely consume the starting material. (hexane/ethyl acetate 9:1) **^{1}H NMR** (400 MHz, $CDCl_3$) δ 7.37 – 7.31 (m, 2H), 7.29 – 7.21 (m, 3H), 6.39 – 6.35 (m, 1H), 4.79 (t, J = 2.9 Hz, 1H), 4.05 (dd, J = 9.7, 5.0 Hz, 1H), 3.94 – 3.88 (m, 1H), 3.70 (d, J = 9.7 Hz, 1H), 3.48 (t, J = 7.2 Hz, 1H), 3.33 (t, J = 5.8 Hz, 1H), 1.43 (s, 3H), 1.29 (s, 3H). **^{13}C NMR** (101 MHz, $CDCl_3$) δ 147.1, 140.3, 128.1, 127.7, 126.3, 103.9, 95.3, 78.6, 68.7, 50.9, 47.6, 44.8, 23.2, 21.1. ESI-HRMS calcd for $C_{16}H_{19}O_2$ $[M+H]^+$ 243.1380 found 243.1381.

4-phenyl-3a,4,4a,5-tetrahydro-7H-cyclobuta[1,2-b:1,4-c']difuran-4a-d 2e

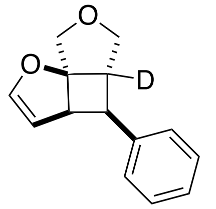

Following the **GP1-P** using **1e** (32.3 mg, 0.15 mmol), **2e** was obtained in 71% (22.8 mg) as transparent viscous oil. The reaction required 72 hours of irradiation to completely consume the starting material. (hexane/ethyl acetate 9:1) **^{1}H NMR** (400 MHz, $CDCl_3$) δ 7.39 – 7.22 (m, 5H), 6.41 – 6.36 (m, 1H), 4.90 – 4.85 (m, 1H), 4.24 (d, J = 10.0 Hz, 1H), 3.98 – 3.89 (m, 2H), 3.86 – 3.78 (m, 2H), 3.59 – 3.53 (m, 1H), 3.40 – 3.35 (m, 1H non-deuterated 2x). **^{13}C NMR** (101 MHz, $CDCl_3$) δ 147.3, 140.1, 128.2, 127.5, 126.4, 104.6, 91.6, 72.9, 71.3, 50.9, 51.0 – 50.4 (m), 43.9. **ESI-HRMS** calcd for $C_{14}H_{14}DO_2$ $[M+H]^+$ 216.1129 found 216.1131.

4-(naphthalen-1-yl)-3a,4,4a,5-tetrahydro-7H-cyclobuta[1,2-b:1,4-c']difuran 2f

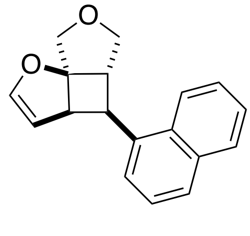

Following the **GP1-P** using **1f** (39.9 mg, 0.15 mmol), **2f** was obtained in 53% (21.0 mg) as white solid. The reaction required 24 hours of irradiation to completely consume the starting material. (hexane/ethyl acetate 9:1) **^{1}H NMR** (400 MHz, $CDCl_3$) δ 7.96 – 7.84 (m, 2H), 7.76 (d, J = 8.2 Hz, 1H), 7.54 – 7.44 (m, 3H), 7.40 (d, J = 7.0 Hz, 1H), 6.29 – 6.25 (m, 1H), 4.41 (brs, 1H), 4.31 (d, J = 9.9 Hz, 1H), 4.16 – 4.12 (m, 2H), 4.05 (dd, J = 9.6, 5.1 Hz, 1H), 3.93 (d, J = 9.6 Hz, 1H), 3.85 (d, J = 9.9 Hz, 1H), 3.68 – 3.62 (m, 1H). **^{13}C NMR** (101 MHz, $CDCl_3$) δ 147.4, 135.5, 133.8, 131.8, 128.9, 127.1, 126.1, 125.7, 125.2, 123.8, 123.7, 104.1, 91.7, 73.2, 71.4, 51.8, 48.7, 42.0. **ESI-HRMS** calcd for $C_{18}H_{17}O_2$ $[M+H]^+$ 265.1223 found 265.1220.

4-(2-methoxyphenyl)-3a,4,4a,5-tetrahydro-7H-cyclobuta[1,2-b:1,4-c']difuran 2g

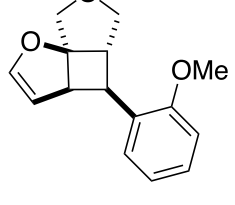

Following the **GP1-P** using **1g** (35.1 mg, 0.144 mmol), **2g** was obtained in 62% (21.9 mg) as colorless viscous oil. The reaction required 46 hours of irradiation to completely consume the starting material. (hexane/ethyl acetate 9:1) **^{1}H NMR** (400 MHz, $CDCl_3$) δ 7.29 – 7.21 (m, 2H), 7.00 – 6.94 (m, 1H), 6.87 (d, J = 7.4 Hz, 1H), 6.33 – 6.29 (m, 1H), 4.80 – 4.75 (m, 1H), 4.24 (d, J = 9.9 Hz, 1H), 3.98 (dd, J = 9.4, 5.0 Hz, 1H), 3.93 – 3.77 (m, 6H), 3.69 (t, J = 7.4 Hz, 1H), 3.39 – 3.35 (m, 1H). **^{13}C NMR** (101 MHz, $CDCl_3$) δ 157.5, 147.0, 128.4, 127.4, 127.3, 120.0, 109.9, 105.0, 91.9, 73.1, 71.4, 55.2, 50.5, 49.6, 39.6. **ESI-HRMS** calcd for $C_{15}H_{17}O_3$ [M+H]+ 245.1172 found 245.1170.

4-(2-chlorophenyl)-3a,4,4a,5-tetrahydro-7H-cyclobuta[1,2-b:1,4-c']difuran 2h

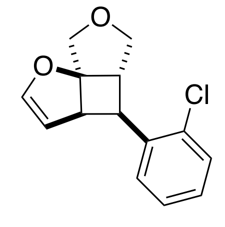

Following the **GP1-P** using **1h** (37.1 mg, 0.15 mmol), **2h** was obtained in 88% (32.7 mg) as white solid. The reaction required 24 hours of irradiation to completely consume the starting material. (hexane/ethyl acetate 9:1) **^{1}H NMR** (400 MHz, $CDCl_3$) δ 7.44 – 7.15 (m, 4H), 6.36 – 6.29 (m, 1H), 4.75 – 4.68 (m, 1H), 4.25 (d, J = 9.9 Hz, 1H), 4.05 – 3.95 (m, 2H), 3.90 (d, J = 9.6 Hz, 1H), 3.83 – 3.74 (m, 2H), 3.43 (t, J = 6.1 Hz, 1H). **^{13}C NMR** (101 MHz, $CDCl_3$) δ 147.5, 137.5, 134.1, 129.5, 128.0, 127.7, 126.4, 104.3, 91.3, 72.9, 71.2, 50.7, 48.8, 42.6. **ESI-HRMS** calcd for $C_{14}H_{14}ClO_2$ $[M+H]^+$ 249.0677 found 249.0673.

4-(3-bromophenyl)-3a,4,4a,5-tetrahydro-7H-cyclobuta[1,2-b:1,4-c']difuran 2i

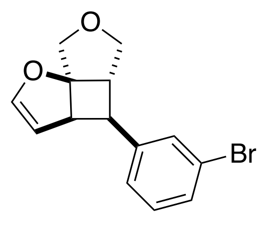

Following the **GP1-P** using **1i** (42.9 mg, 0.146 mmol), **2i** was obtained in 84% (37.1 mg) as white solid. The reaction required 48 hours of irradiation to completely consume the starting material. (hexane/ethyl acetate 9:1) **^{1}H NMR** (400 MHz, $CDCl_3$) δ 7.41 – 7.35 (m, 2H), 7.24 – 7.15 (m, 2H), 6.42 – 6.38 (m, 1H), 4.88 – 4.83 (m, 1H), 4.23 (d, J = 9.9 Hz, 1H), 3.96 – 3.87 (m, 2H), 3.84 – 3.76 (m, 2H), 3.51 (t, J = 7.3 Hz, 1H), 3.36 – 3.28 (m, 1H). **^{13}C NMR** (101 MHz, $CDCl_3$) δ 147.6, 142.6, 130.6, 129.8, 129.5, 126.2, 122.4, 104.1, 91.6, 72.8, 71.2, 43.7. **ESI-HRMS** calcd for $C_{14}H_{14}BrO_2$ $[M+H]^+$ 293.0172 found 293.0170.

4-(4-fluorophenyl)-3a,4,4a,5-tetrahydro-7H-cyclobuta[1,2-b:1,4-c']difuran 2j

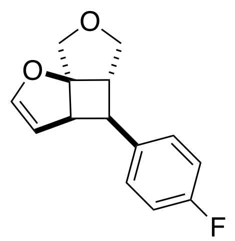

Following the **GP1-P** using **1j** (34.3 mg, 0.15 mmol), **2j** was obtained in 70% (24.0 mg) as pale yellow liquid. The reaction required 40 hours of irradiation to completely consume the starting material. (hexane/ethyl acetate 9:1) **^{1}H NMR** (400 MHz, $CDCl_3$) δ 7.23 – 7.15 (m, 2H), 7.04 – 6.96 (m, 2H), 6.40 – 6.35 (m, 1H), 4.83 – 4.78 (m, 1H), 4.20 (d, J = 9.9 Hz, 1H), 3.94 – 3.84 (m, 2H), 3.81 – 3.73 (m, 2H), 3.49 (t, J = 7.3 Hz, 1H), 3.32 – 3.25 (m, 1H). **^{13}C NMR** (101 MHz, $CDCl_3$) δ 161.6 (d, J = 244.5 Hz), 147.5, 135.8 (d, J = 3.3 Hz), 129.0 (d, J = 7.8 Hz), 115.0 (d, J = 21.2 Hz), 104.3, 91.5, 72.8, 71.2, 51.3, 51.1, 43.4. **^{19}F NMR** (565 MHz, $CDCl_3$) δ -116.57 – -116.68 (m). **ESI-HRMS** calcd for $C_{14}H_{14}FO_2$ $[M+H]^+$ 233.0972 found 233.0970.

4-(4-(trifluoromethyl)phenyl)-3a,4,4a,5-tetrahydro-7H-cyclobuta[1,2-b:1,4-c']difuran 2k

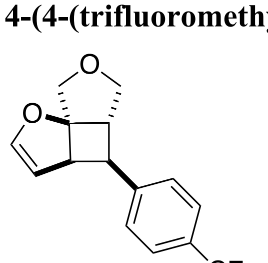

Following the **GP1-P** using **1k** (42.3 mg, 0.15 mmol), **2k** was obtained in 75% (31.7 mg) as pale yellow liquid. The reaction required 32 hours of irradiation to completely consume the starting material. (hexane/ethyl acetate 9:1) **^{1}H NMR** (400 MHz, $CDCl_3$) δ 7.57 (d, J = 8.1 Hz, 2H), 7.34 (d, J = 8.0 Hz, 2H), 6.40 – 6.35 (m, 1H), 4.83 – 4.77 (m, 1H), 4.22 (d, J = 10.0 Hz, 1H), 3.96 – 3.75 (m, 4H), 3.57 (t, J = 7.3 Hz, 1H), 3.37 – 3.31 (m, 1H). **^{13}C NMR** (101 MHz, $CDCl_3$) δ 147.7, 144.2 (d, J = 1.3 Hz), 128.6 (d, *J* = 32.3 Hz), 127.8, 125.1 (d, J = 4.0 Hz), 124.2 (q, *J* = 271.8 Hz), 104.0, 91.6, 72.7, 71.2, 50.9, 50.8, 43.9. **^{19}F NMR** (565 MHz, $CDCl_3$) δ -62.3. **ESI-HRMS** calcd for $C_{15}H_{14}F_3O_2$ $[M+H]^+$ 283.0940 found 283.0943.

4-phenyl-6-tosyl-3a,4,4a,5,6,7-hexahydrofuro[2',3':1,4]cyclobuta[1,2-c]pyrrole 2l

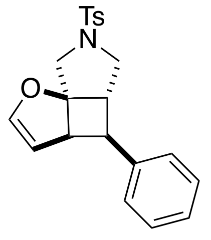

Following the **GP1-P** using **1l** (55.0 mg, 0.15 mmol), **2l** was obtained in 56% (30.8 mg) as white solid. The reaction required 60 hours of irradiation to completely consume the starting material. (hexane/ethyl acetate 9:1) **^{1}H NMR** (400 MHz, $CDCl_3$) δ 7.76 (d, J = 8.3 Hz, 2H), 7.42 – 7.19 (m, 7H), 6.30 – 6.26 (m, 1H), 4.84 (t, J = 2.9 Hz, 1H), 3.96 (d, J = 10.2 Hz, 1H), 3.92 – 3.87 (m, 1H), 3.67 (t, J = 7.4 Hz, 1H), 3.54 (d, J = 9.9 Hz, 1H), 3.26 – 3.20 (m, 1H), 2.98 – 2.91 (m, 2H), 2.48 (s, 3H). **^{13}C NMR** (101 MHz, $CDCl_3$) δ 146.7, 144.1, 139.4, 131.4, 129.8, 128.2, 128.1, 127.5, 126.5, 104.5, 89.9, 52.9, 52.8, 51.4, 49.6, 44.2, 21.6. **ESI-HRMS** calcd for $C_{21}H_{21}NNaO_3S$ $[M+Na]^+$ 390.1134 found 390.1133.

4,5-diphenyl decahydro-1H-furo[3''',4''':2'',3'']cyclobuta[1'',2'':4',5']furo[2',3':1,4] cyclobuta[1,2-c]furan 2m

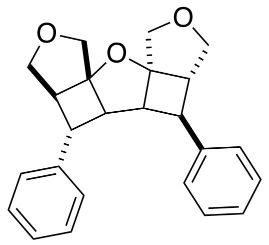

Following the **GP1-P** using **1m** (43.3 mg, 0.12 mmol), **2m** was obtained in 62% (26.8 mg) as withe solid. The reaction required 72 h of irradiation to fully consume the starting material. (hexane/ethyl acetate 9:1) **^{1}H NMR** (400 MHz, $CDCl_3$) δ 7.43 – 7.02 (m, 10H), 3.99 – 3.81 (m, 6H), 3.71 (d, *J*=9.6, 2H), 3.18 (t, *J*=7.8, 2H), 2.89 – 2.74 (m, 4H). **^{13}C NMR** (101 MHz, $CDCl_3$) δ 140.0, 128.6, 127.6, 126.5, 94.2, 72.8, 71.8, 47.9, 47.5, 38.1, 37.9. **ESI-HRMS** calcd for $C_{24}H_{24}O_3$ $[M+H]^+$ 360.1725 found 359.1721.

(4*S*)-2-((allyloxy)methyl)-4-phenyl-3a,4,4a,5-tetrahydro-7*H*-cyclobuta[1,2-*b*:1,4-*c'*]difuran 2n

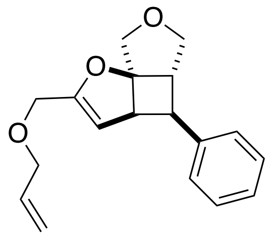

Following the **GP1-P** using **1n** (42.6 mg, 0.15 mmol), **2n** was obtained in 66% (28.2 mg) as yellow solid. The reaction required 48 h of irradiation to fully consume the starting material. (hexane/ethyl acetate 9:1) **^{1}H NMR** (600 MHz, $CDCl_3$) δ 7.30 (t, J=7.6, 2H), 7.21 (dt, J=7.8, 2.7, 3H), 5.93 – 5.81 (m, 1H), 5.24 – 5.14 (m, 1H), 4.79 (d, J=2.6, 1H), 4.20 (d, J=10.0, 1H), 4.05 – 3.77 (m, 8H), 3.51 (t, J=7.4, 1H), 3.36 (t, J=6.0, 1H). **^{13}C NMR** (101 MHz, $CDCl_3$) δ 156.5, 140.3, 134.4, 128.3, 127.6, 126.4, 117.8, 103.2, 92.3, 72.9, 71.5, 71.4, 64.6, 51.9, 51.2, 44.1 **ESI-HRMS** calcd for $C_{18}H_{20}O_3$ $[M+H]^+$ 284.1412 found 284.1415

4-phenyl-3a,4,4a,5-tetrahydro-7H-thieno[2',3':1,4]cyclobuta[1,2-c]furan 2o

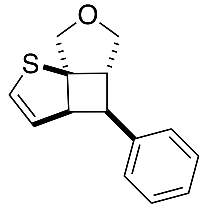

Following the **GP3-P** using **1o** (34.3 mg, 0.15 mmol), **2o** was obtained in 35% (11.5 mg) as white solid. The reaction required 48 hours of irradiation to completely consume the starting material. (hexane/ethyl acetate 9:1) **^{1}H NMR** (400 MHz, $CDCl_3$) δ 7.37 – 7.22 (m, 5H), 6.11 (dd, J = 6.1, 1.8 Hz, 1H), 5.09 – 5.04 (m, 1H), 4.31 (d, J = 10.0 Hz, 1H), 4.06 – 4.00 (m, 1H), 3.92 (d, J = 9.6 Hz, 1H), 3.86 – 3.77 (m, 2H), 3.67 (d, J = 10.0 Hz, 1H), 3.50 – 3.44 (m, 1H). **^{13}C NMR** (101 MHz, $CDCl_3$) δ 139.6, 128.2, 128.1, 127.1, 126.5, 123.9, 74.2, 73.7, 61.1, 58.5, 55.7, 49.2. **ESI-HRMS** calcd for $C_{14}H_{14}NaOS$ $[M+Na]^+$ 253.0658, found 253.0655.

7-phenyl-2,8-ditosyl-1,2,3,6,7,7a-hexahydro-3a,6-epiminoisoindole 2p

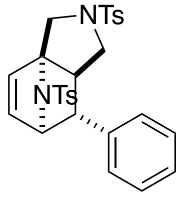

Following the **GP1-P** using **1p** (78.1 mg, 0.15 mmol), **2p** was obtained in 55% (42.9 mg) as white solid. The reaction required 36 hours of irradiation to completely consume the starting material. (hexane/ethyl acetate 9:1) **^{1}H NMR** (400 MHz, $CDCl_3$) δ 7.77 – 7.72 (m, 2H), 7.60 – 7.55 (m, 2H), 7.37 – 7.22 (m, 9H), 6.30 – 6.26 (m, 1H), 5.42 – 5.39 (m, 1H), 4.72 – 4.69 (m, 1H), 4.12 (d, J = 9.3 Hz, 1H), 3.71 (d, J = 9.4 Hz, 1H), 3.51 – 3.44 (m, 1H), 2.70 – 2.61 (m, 1H), 2.48 – 2.37 (m, 8H). **^{13}C NMR** (101 MHz, $CDCl_3$) δ 144.1, 143.9, 141.3, 139.3, 136.2, 134.8, 132.1, 130.0, 129.9, 128.9, 128.1, 127.2, 127.1, 127.0, 75.7, 75.2, 58.4, 48.4, 48.1, 47.9, 21.7, 21.6. **ESI-HRMS** calcd for $C_{28}H_{29}N_2O_4S_2$ $[M+H]^+$ 521.1563, found 521.1565.

4-phenyl-3a,4,4a,5-tetrahydro-1Hbenzo[4',5']cyclobuta[1,2-b:1,4-c']difuran 4a

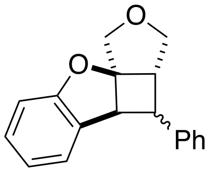

Following the **GP3-P** using **3a** (38.1 mg, 0.144 mmol), **4a** was obtained in 93% yield as a mixture of diastereomers (86:14) as yellow solid (35.2 mg). (hexane/ethyl acetate 9.5:0.5) **^{1}H NMR** (400 MHz, $CDCl_3$) δ 7.75 – 7.67 (m, 1H Dia2), 7.53 – 7.46 (m, 1H Dia2), 7.45 – 7.08 (m, 4H Dia1, 5H Dia2), 7.04 – 6.85 (m, 3H Dia1, 2H Dia2), 6.72 – 6.64 (m, 1H Dia1), 6.43 (d, J = 7.4 Hz, 1H Dia1), 4.34 (d, J = 10.0 Hz, 1H Dia1), 4.28 – 4.18 (m, 1H Dia1, 2H Dia2), 4.03 – 3.91 (m, 3H Dia1), 3.86 – 3.68 (m, 1H Dia1, 3H Dia2), 3.62 (d, J = 10.3 Hz, 1H Dia2), 3.46 – 3.39 (m, 1H Dia2), 3.38 – 3.31 (m, 1H Dia1). **^{13}C NMR** (101 MHz, CDCl3) δ 160.6 (Dia2), 160.5 (Dia1), 138.9 (Dia1), 138.4 (Dia2), 132.2 (Dia2), 132.1 (Dia2), 132.0 (Dia2), 131.4 (Dia2), 128.7 (Dia2), 128.5 (Dia1), 128.3 (Dia2), 128.1 (Dia1), 128.0 (Dia1), 127.8 (Dia1), 127.4 (Dia1), 126.8 (Dia2), 126.6 (Dia1), 125.0 (Dia2), 121.6 (Dia2), 120.9 (Dia1), 110.3 (Dia2), 110.2 (Dia1), 94.2 (Dia2), 92.6 (Dia1), 72.9 (Dia1), 71.7 (Dia2), 71.5 (Dia1), 69.2 (Dia2), 50.9 (Dia1), 50.5 (Dia1), 49.1 (Dia2), 48.8 (Dia2), 46.5 (Dia2), 44.0 (Dia1). **ESI-HRMS** calcd for $C_{18}H_{17}O_2$ $[M+H]^+$ 265.1223, found 265.1222.

4-phenyl-2-tosyl-1,2,3,3a,4,4a-hexahydrobenzofuro[2',3':1,4]cyclobuta[1,2-c]pyrrole 4b

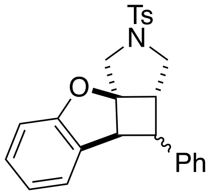

Following the **GP3-P** using **3b** (62.7 mg, 0.15 mmol), **4b** was obtained in 92% yield as a mixture of diastereomers (85:15) as white solid (57.7 mg). (hexane/ethyl acetate 9.5:0.5) **^{1}H NMR** (400 MHz, $CDCl_3$) δ 7.76 (d, J = 8.3 Hz, 2H Dia1), 7.64 (d, J = 8.3 Hz, 2H Dia2), 7.45 – 7.29 (m, 2H Dia1, 6H Dia2), 7.23 – 7.12 (m, 3H Dia1, 3H Dia2), 7.11 – 7.04 (m, 1H Dia1), 6.94 – 6.85 (m, 2H Dia1, 1H Dia2), 6.82 (d, J = 8.0 Hz, 1H Dia2), 6.76 (d, J = 8.0 Hz, 1H Dia1), 6.68 – 6.61 (m, 1H Dia1), 6.39 (d, J = 7.4 Hz, 1H Dia1), 4.36 – 4.27 (m, 1H Dia1, 1H Dia2), 4.04 (d, J = 10.3 Hz, 1H Dia1), 3.90 – 3.72 (m, 1H Dia1, 2H Dia2), 3.56 (d, J = 10.0 Hz, 1H Dia1), 3.30 – 3.23 (m, 1H Dia2), 3.22 – 3.15 (m, 1H Dia1), 3.10 (d, J = 10.5 Hz, 1H Dia2), 3.05 (d, J = 10.3 Hz, 1H Dia1), 2.99 – 2.91 (m, 1H Dia1, 1H Dia2), 2.80 – 2.74 (m, 1H Dia2), 2.52 – 2.43 (m, 3H Dia1, 3H Dia2). **^{13}C NMR** (101 MHz, $CDCl_3$) δ 160.3, 159.9, 144.2, 144.0, 138.2, 137.1, 131.2, 130.9, 129.9, 129.7, 128.72, 128.66, 128.4, 128.2, 128.14, 128.05, 127.8, 127.3, 126.84, 126.79, 124.9, 121.8, 121.2, 110.2, 110.0, 92.3, 90.9, 53.6, 53.0, 52.8, 51.3, 49.2, 49.1, 48.6, 47.8, 46.9, 44.1, 21.63, 21.59. **ESI-HRMS** calcd for $C_{25}H_{24}NO_3S$ $[M+H]^+$ 418.1471, found 418.1474.

4-phenyl-3,3a,4,4a-tetrahydro-1H-benzo[4',5']thieno[2',3':1,4]cyclobuta[1,2-c]furan 4c

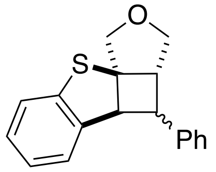

Following the **GP3-P** using **3c** (42.1 mg, 0.15 mmol), **4c** was obtained in 99% yield as a mixture of diastereomers (75:25). (41.6 mg) The scale-up reaction was performed on 1.5 mmol of **3c**, the reaction required 50 hours of irradiation to completely consume the starting material (95%, 392.5 mg, 1.4 mmol). The two diastereomers were then separated and crystallized to obtain their relative configurations. (hexane/ethyl acetate 9.5:0.5) **Dia 1** (white solid), **^{1}H NMR** (400 MHz, $CDCl_3$) δ 7.19 – 7.09 (m, 4H), 7.06 – 6.95 (m, 3H), 6.70 – 6.64 (m, 1H), 6.18 (d, J = 7.7 Hz, 1H), 4.41 (d, J = 9.2 Hz, 1H), 4.34 (d, J = 10.0 Hz, 1H), 3.99 (d, J = 9.7 Hz, 1H), 3.89 – 3.82 (m, 2H), 3.69 (d, J = 10.0 Hz, 1H), 3.42 – 3.36 (m, 1H). **^{13}C NMR** (101 MHz, $CDCl_3$) δ 142.2, 139.2, 138.5, 128.5, 127.9, 127.7, 126.9, 126.6, 124.1, 121.7, 74.7, 74.0, 60.4, 57.9, 56.0, 49.1. **ESI-HRMS** calcd for $C_{18}H_{16}NaOS$ $[M+Na]^+$ 303.0814 found 303.0816. **Dia 2,** (white solid): **^{1}H NMR** (400 MHz, $CDCl_3$) δ 7.47 – 7.14 (m, 7H), 7.06 – 7.00 (m, 2H), 4.44 (d, J = 7.6 Hz, 1H), 4.17 (d, J = 9.6 Hz, 1H), 3.92 (dd, J = 9.4, 7.6 Hz, 1H), 3.83 – 3.70 (m, 2H), 3.58 (d, J = 9.6 Hz, 1H), 3.47 – 3.40 (m, 1H). **^{13}C NMR** (101 MHz, $CDCl_3$) δ 142.4, 141.9, 138.3, 128.6, 128.04, 128.01, 126.8, 124.8, 124.2, 122.0, 75.1, 69.3, 60.4, 56.9, 53.0, 48.0. **ESI-HRMS** calcd for $C_{18}H_{16}NaOS$ $[M+Na]^+$ 303.0814, found 303.0817.

4-(p-tolyl)-3,3a,4,4a-tetrahydro-1H-benzo[4',5']thieno[2',3':1,4]cyclobuta[1,2-c]furan 4d

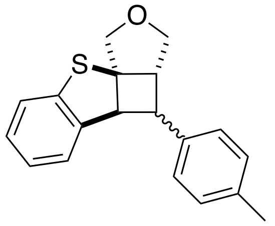

Following the **GP3-P** using **3d** (44.2 mg, 0.15 mmol), **4d** was obtained in 99% yield as a mixture of diastereomers (75:25) as pale yellow solid (43.7 mg). (hexane/ethyl acetate 9.5:0.5) **^{1}H NMR** (400 MHz, $CDCl_3$) δ 7.30 – 7.15 (m, 1H Dia1, 6H Dia2), 7.10 – 6.94 (m, 3H Dia1, 2H Dia2), 6.91 – 6.85 (m, 2H Dia1), 6.73 (t, J = 7.4 Hz, 1H Dia1), 6.24 (d, J = 7.6 Hz, 1H Dia1), 4.44 – 4.33 (m, 2H Dia1, 1H Dia2), 4.17 (d, J = 9.6 Hz, 1H Dia2), 4.00 (d, J = 9.7 Hz, 1H Dia1), 3.91 – 3.78 (m, 2H Dia1, 2H Dia2), 3.75 – 3.68 (m, 1H Dia1, 1H Dia2), 3.57 (d, J = 9.6 Hz, 1H Dia2), 3.44 – 3.35 (m, 1H Dia1, 1H Dia2), 2.40 (s, 3H Dia2), 2.28 (s, 3H Dia1). **^{13}C NMR** (101 MHz, $CDCl_3$) δ 142.4, 142.1, 142.0, 138.7, 136.4, 136.2, 136.1, 135.3, 129.3, 128.6, 128.4, 128.0, 127.9, 127.7, 127.0, 124.8, 124.1, 121.9, 121.7, 75.1, 74.7, 73.9, 69.3, 60.4, 57.9, 57.1, 56.4, 53.0, 48.8, 47.7, 21.1, 21.0. **ESI-HRMS** calcd for $C_{19}H_{19}OS$ $[M+H]^+$ 295.1151, found 295.1154.

4-phenyl-3,3a,4,4a-tetrahydro-1H-benzo[4',5']thieno[3',2':1,4]cyclobuta[1,2-c]furan 4e

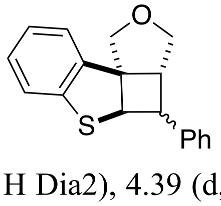

Following the **GP3-P** using **3e** (42.0 mg, 0.15 mmol), **4e** was obtained in 92% yield as a mixture of diastereomers (79:21) as white solid (38.8 mg). (hexane/ethyl acetate 9.5:0.5) **^{1}H NMR** (400 MHz, $CDCl_3$) δ 7.44 – 7.07 (m, 9H Dia1, 9H Dia2), 4.71 (d, J = 8.4 Hz, 1H Dia1), 4.47 (d, J = 7.8 Hz, 1H Dia2), 4.39 (d, J = 10.0 Hz, 1H Dia1), 4.26 (d, J = 9.6 Hz, 1H Dia2), 4.11 – 4.00 (m, 1H Dia1, 1H Dia2), 3.97 – 3.83 (m, 3H Dia1, 1H Dia2), 3.82 – 3.75 (m, 1H Dia2), 3.69 (d, J = 9.7 Hz, 1H Dia2), 3.51 – 3.45 (m, 1H Dia2), 3.36 (t, J = 5.5 Hz, 1H Dia1). **^{13}C NMR** (101 MHz, $CDCl_3$) δ 145.4, 144.3, 139.8, 139.2, 138.1, 138.0, 128.9, 128.6, 128.50, 128.47, 128.1, 127.9, 126.9, 126.8, 124.7, 124.4, 124.1, 123.1, 122.8, 121.9, 76.0, 75.4, 74.2, 69.1, 64.6, 63.6, 55.4, 55.1, 54.9, 52.2, 50.8, 48.1. **ESI-HRMS** calcd for $C_{18}H_{17}OS$ $[M+H]^+$ 281.0995, found 281.0993.

4-(3-ethynylphenyl)-3,3a,4,4a-tetrahydro-1H-benzo[4',5']thieno[3',2':1,4]cyclobuta[1,2-c]furan 4f

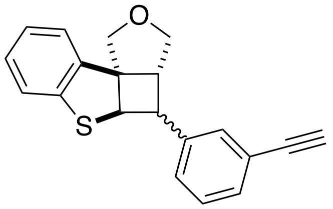

Following the **GP3-P** using **3f** (45.7 mg, 0.15 mmol), **4f** was obtained in 99% yield as a mixture of diastereomers (83:17) as white solid (45.3 mg,). (hexane/ethyl acetate 9.5:0.5) ^{1}**H NMR** (400 MHz, $CDCl_3$) δ 7.44 – 7.32 (m, 1H Dia1, 3H Dia2), 7.31 – 7.21 (m, 3H Dia1, 3H Dia2), 7.21 – 7.05 (m, 4H Dia1, 2H Dia2), 4.68 (d, J = 8.4 Hz, 1H Dia1), 4.43 (d, J = 7.8 Hz, 1H Dia2), 4.38 (d, J = 10.0 Hz, 1H Dia1), 4.25 (d, J = 9.7 Hz, 1H Dia2), 4.06 (d, J = 9.7 Hz, 1H Dia1), 4.02 – 3.96 (m, 1H Dia2), 3.95 – 3.88 (m, 2H Dia1), 3.86 – 3.75 (m, 1H Dia1, 2H Dia2), 3.67 (d, J = 9.7 Hz, 1H Dia2), 3.49 – 3.43 (m, 1H Dia2), 3.33 (t, J = 5.5 Hz, 1H Dia1), 3.11 (s, 1H Dia2), 3.06 (s, 1H Dia1). ^{13}**C NMR** (101 MHz, $CDCl_3$) δ 145.1, 144.2, 140.0, 139.0, 138.4, 137.8, 132.1, 131.5, 130.6, 130.5, 129.0, 128.9, 128.7, 128.5, 128.3, 128.1, 124.7, 124.5, 124.1, 123.1, 122.8, 122.4, 121.9, 121.8, 83.7, 77.3, 77.1, 75.9, 75.3, 74.1, 69.0, 64.6, 63.6, 55.2, 54.8, 54.7, 51.9, 50.3, 47.7. **ESI-HRMS** calcd for $C_{20}H_{17}OS$ $[M+H]^+$ 305.0955, found 305.0953.

9-methyl-4-phenyl-3a,4,4a,9-tetrahydro-1H,3H-furo[3',4':1,4]cyclobuta[1,2-b]indole 4g

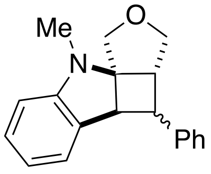

Following the **GP3-P** using **3g** (42.0 mg, 0.15 mmol), **4g** was obtained in 89% yield as a mixture of diastereomers (71:29) as pale yellow solid (37.4 mg). (hexane/ethyl acetate 9:1) ^{1}**H NMR** (400 MHz, $CDCl_3$) δ 7.92 – 7.86 (m, 1H Dia2), 7.55 – 7.49 (m, 1H Dia2), 7.45 – 6.90 (m, 6H Dia1, 5H Dia2), 6.72 – 6.66 (m, 1H Dia2), 6.59 – 6.50 (m, 1H Dia1, 1H Dia2), 6.49 – 6.42 (m, 1H Dia1), 6.20 (dt, J = 7.4, 1.3 Hz, 1H Dia1), 4.20 (d, J = 10.1 Hz, 1H Dia1), 4.11 – 4.05 (m, 1H Dia1, 1H Dia2), 4.04 – 3.98 (m, 2H Dia2), 3.94 (d, J = 9.6 Hz, 1H Dia1), 3.89 – 3.80 (m, 2H Dia1), 3.78 – 3.72 (m, 1H Dia1), 3.71 – 3.65 (m, 3H Dia2), 3.49 – 3.42 (m, 1H Dia2), 3.39 – 3.32 (m, 1H Dia1), 2.92 – 2.81 (m, 3H Dia1, 3H Dia2). ^{13}**C NMR** (101 MHz, $CDCl_3$) δ 154.3, 153.5, 139.3, 138.9, 133.4, 128.9, 128.8, 128.4, 128.3, 128.2, 128.0, 127.9, 127.8, 127.0, 126.5, 126.4, 125.9, 123.9, 118.5, 118.1, 108.3, 107.3, 75.5, 75.0, 74.0, 71.1, 71.0, 69.9, 50.2, 48.8, 47.3, 45.1, 44.2, 44.1, 30.5, 30.0. **ESI-HRMS** calcd for $C_{19}H_{20}NO$ $[M+H]^+$ 278.1539, found 278.1541.

9-methyl-4-(naphthalen-1-yl)-3a,4,4a,9-tetrahydro-1H,3H-furo[3',4':1,4]cyclobuta[1,2-b]indole 4h

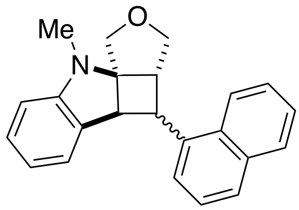

Following the **GP3-P** using **3h** (49.1 mg, 0.15 mmol), **4h** was obtained in 97% yield as a mixture of diastereomers (90:10) as yellow solid (47.8 mg). (hexane/ethyl acetate 9.5:0.5) **^{1}H NMR** (400 MHz, $CDCl_3$) δ 8.18 (d, J = 7.9 Hz, 1H Dia2), 7.95 – 7.87 (m, 1H Dia1, 1H Dia2), 7.85 – 7.76 (m, 2H Dia1), 7.74 – 7.55 (m, 2H Dia1, 3H Dia2), 7.54 – 7.42 (m, 2H Dia1, 2H Dia2), 7.31 – 7.25 (m, 1H Dia2), 7.24 – 7.16 (m, 1H Dia1), 7.05 (d, J = 7.2 Hz, 1H Dia1, 1H Dia2), 6.95 (t, J = 7.7 Hz, 1H Dia2), 6.75 – 6.67 (m, 1H Dia1), 6.59 (d, J = 7.9 Hz, 1H Dia1), 5.64 (d, J = 7.3 Hz, 1H Dia2), 4.47 – 4.38 (m, 1H Dia1, 2H Dia2), 4.37 – 4.29 (m, 1H Dia1, 1H Dia2), 4.03 – 3.96 (m, 1H Dia1, 2H Dia2), 3.78 – 3.65 (m, 2H Dia1, 2H Dia2), 3.60 – 3.53 (m, 1H Dia1), 3.17 (d, J = 10.2 Hz, 1H Dia1), 2.95 (s, 3H Dia1), 2.88 (s, 3H Dia2). **^{13}C NMR** (101 MHz, $CDCl_3$) δ 153.6, 134.1, 133.8, 133.7, 133.6, 131.9, 131.6, 129.0, 128.9, 128.3, 128.0, 127.2, 126.9, 126.2, 126.0, 125.9, 125.7, 125.58, 125.56, 125.2, 124.5, 124.1, 123.9, 123.7, 123.5, 118.4, 107.5, 75.5, 75.4, 74.1, 70.9, 70.1, 50.3, 46.5, 44.8, 44.2, 41.5, 30.3, 29.8. **ESI-HRMS** calcd for $C_{23}H_{21}NNaO$ $[M+Na]^+$ 350.1515, found 350.1511.

Valorization of the products

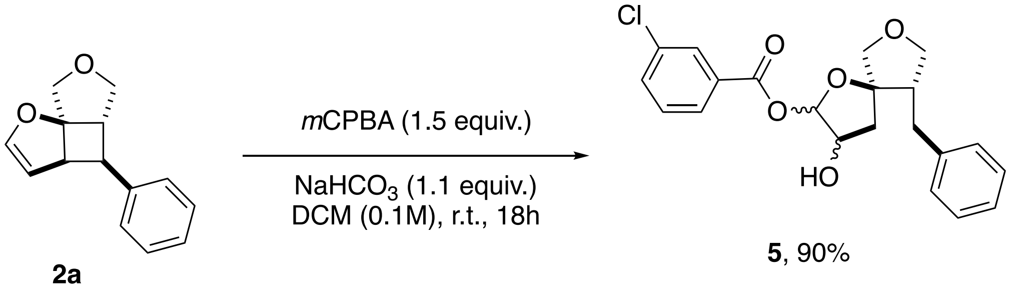

2a **5**, 90%

In a round bottom flask equipped with a magnetic stirring bar, **2a** (31.9 mg, 0.15 mmol, 1 equiv.), $NaHCO_3$ (0.17 mg, 13.4 mmol, 1.1 equiv.) and *m*CPBA (75% pure, 50.6 mg, 0.22 mmol, 1.5 equiv.) were dissolved in DCM (1.5 mL, 0.1M). Then the reaction was stirred at room temperature for 18 hours. After complete conversion as monitored by TLC, the solution was quenched with HCl, the product was extracted with EtOAc. The organic phase was washed with brine, dried over Na_2SO_4 and concentrated under reduced pressure. The crude was finally purified by chromatography on silica gel (hexane/ethyl acetate 9.5:0.5 to 8:2 gradient) to afford the desired product **5** as yellow oil in 90% (28.7 mg).

Regio and stereochemistry of the **5** were determined by 2D NMRs and on the literature. [10],[11]

^{1}H NMR (400 MHz, $CDCl_3$) δ 7.79 – 7.11 (m, 7H), 6.31 (s, 1H), 4.33 (s, 1H), 4.15 (d, *J*=9.9, 1H), 3.93 – 3.87 (m, 2H), 3.77 (d, *J* = 9.9, 1H), 3.50 (dd, *J* = 9.7, 7.2, 1H), 3.26 – 3.19 (m, 1H), 3.13 (d, *J*=9.8, 1H), 2.54 (s, 1H). **^{13}C NMR** (101 MHz, $CDCl_3$) δ 164.6, 139, 134.8, 133.6, 129.8, 128.7, 127.2, 126.5, 107.9, 93.6, 78.1, 73.2, 71.7, 54.2, 48.2, 36.9. **ESI-HRMS** calcd for $C_{21}H_{19}ClO_5$ $[M+H]^+$ 387.0994 found 387.0992.

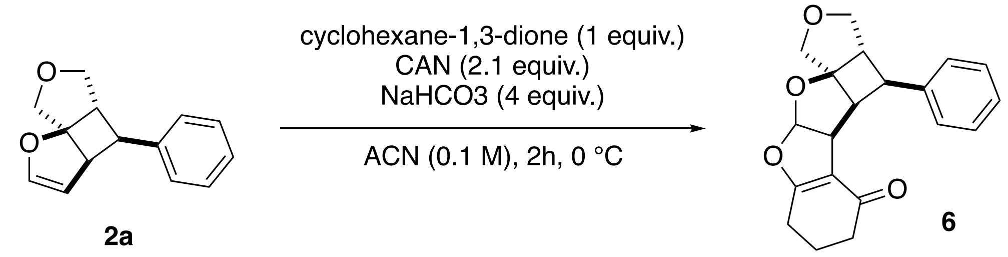

2a **6**

A solution of the cyclohexane-1,3-dione (11.2 mg, 0.1 mmol, 1 equiv.) and **2a** (25.7 mg, 0.12 mmol, 1.2 equiv) in ACN (1 mL, 0.1M) was added dropwise to a stirred mixture of CAN (115.1 mg, 0.21 mmol, 2.1 equiv.) and $NaHCO_3$ (33.6 mg, 0.4 mmol, 4 equiv.) in ACN (1 mL, 0.1M) at 0°C. The reaction mixture was stirred at 0°C for 2h, diluted with ACN and filtered. The crude was purified by chromatography on silica gel (hexane/ethyl acetate 9.5:0.5 to 8:2 gradient) to afford the desired product **6** as an orange oil in 53% (16.9 mg, 0.53 mmol).[12]

^{1}H NMR (400 MHz, $CDCl_3$) δ 7.46 – 7.10 (m, 7H dia1, 7H dia2), 6.03 (d, *J*=6.5, 1H dia1), 5.76 (d, *J*=7.1, 1H dia2), 4.28 (d, *J*=7.9, 1H dia2), 4.19 – 4.07 (m, 3H dia2), 4.02 (d, *J*=9.7, 1H dia1), 3.97 (d, *J*=3.2, 3H dia1), 3.75 (d, *J*=9.8, 1H dia1, 1H dia2), 3.49 – 3.35 (m, 2H), 3.33 (d, *J*=7.0, 1H dia2f), 3.24 (d, *J*=10.0, 1H dia1), 3.02 – 2.94 (m, 1H dia1, 1H dia2), 2.79 – 2.64 (m, 0H), 2.54 – 2.41 (m, 2H), 2.37 – 2.32 (m, 2H dia1), 2.12 – 1.95 (m, 2H dia1, 2H dia2). **^{13}C NMR** (101 MHz, $CDCl_3$) δ = 195.0, 175.0, 139.6, 128.7, 127.7, 126.5, 115.6, 113.0, 73.0, 71.7, 53.4, 48.5, 47.2, 46.6, 38.0, 36.7, 29.7, 23.8, 21.6. **ESI-HRMS** calcd for $C_{20}H_{20}O_4$ $[M+H]^+$ 325.1434 found 325.1435

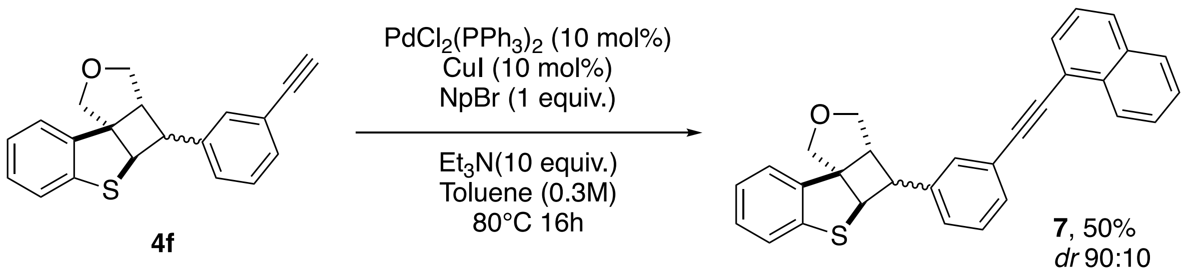

In a flame-dried Schlenk flask, under nitrogen, **4f** (45.7 mg, 0.15 mmol, 1 equiv.), CuI (2.8 mg, 0.015 mmol, 10 mol%), $PdCl_2(PPh_3)_2$ (10.5 mg, 0.015 mmol, 10 mol%) and NpBr (31.1 mg, 0.15 mmol, 1equiv.) were added. Then, sequentially, triethylamine (200 µL, 1.5 mmol, 10 equiv.) and toluene (500 µL, 0.3M) were added. The reaction mixture was heated at 80 °C for 16 hours. Upon completion of the reaction, as monitored by TLC, the mixture was filtered through celite and quenched with water. The crude was then extracted with DCM and washed with water and brine. The organic layer was dried over Na_2SO_4, concentrated under reduced pressure. The crude was purified by chromatography on silica gel (hexane/ethyl acetate 9.5:0.5 to 8:2 gradient) to afford the desired product **7** as red solid in 50% as mixture of diastereoisomers (90:10) (32.3 mg, 0.075 mmol).

^{1}H NMR (400 MHz, $CDCl_3$) δ 7.96 – 6.79 (m, 7H), 4.65 (d, J = 8.4, 1H), 4.35 (m, 1H dia1, 1H dia2), 4.21 (d, *J* = 9.7, 1H dia2), 4.02 (d, *J* = 9.8, 1H dia1), 3.95 (t, J = 8.6, 1H dia2), 3.88 (dd, J = 10.0, 3.9, 2H dia1), 3.82 – 3.77 (m, 1H dia1), 3.77 – 3.68 (m, 2H dia2), 3.64 (d, J = 9.7, 1H dia2), 3.45 – 3.40 (m, 1H dia2), 3.29 (t, J = 5.6, 1H dia1). **^{13}C NMR** (101 MHz, $CDCl_3$) δ 145.2, 140.2, 139.0, 132.5, 131.0, 129.6, 128.7, 128.3, 124.6, 124.6, 123.1, 122.1, 121.6, 81.7, 76.0, 74.1, 74.0 ,63.7, 54.8, 54.7, 51.0, 47.7. **ESI-HRMS** calcd for $C_{30}H_{22}OS$ $[M+H]^+$ 431.1464 found 431.1466

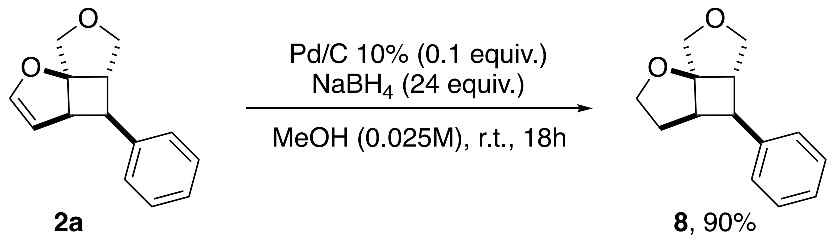

2a **8**, 90%

In a round bottom flask equipped with a magnetic stirring bar, **2a** (31.9 mg, 0.15 mmol, 1 equiv.) was dissolved in MeOH (6 mL, 0.025M). The reaction mixture was cooled to 0 °C and Pd/C 10% (15.9 mg, 0.015 mmol, 0.1 equiv.) was added under nitrogen atmosphere. After 10 minutes at 0 °C $NaBH_4$ (136 mg, 3.6 mmol, 24 equiv.) was added. The reaction was stirred at room temperature till complete conversion of the starting material which occurs after 18 hours. The solution was then quenched with water, extracted with EtOAc and the organic phase was washed with water and brine. The organic phase was dried over with Na_2SO_4, filtered and concentrated under reduced pressure. The crude was purified by chromatography on silica gel (hexane/ethyl acetate 9.5:0.5 to 8:2 gradient) to afford the desired product **8** as white solid in 90% (32.4 mg, 0.15mmol).[13]

^{1}H NMR (400 MHz, $CDCl_3$) δ 7.40 – 7.14 (m, 5H), 4.09 – 4.02 (m, 2H), 3.99 – 3.90 (m, 2H), 3.73 (d, *J*=9.5, 1H), 3.71 – 3.62 (m, 1H), 3.37 – 3.31 (m, 1H), 3.16 – 2.98 (m, 2H), 1.84 (m, 1H), 1.58 – 1.51 (m, 1H). **^{13}C NMR** (101 MHz, $CDCl_3$) δ 139.6, 128.6, 127.5, 126.2, 90.6, 73.5, 71.9, 69.2, 45.6, 45.0, 38.3, 26.6. **ESI-HRMS** calcd for $C_{14}H_{16}O_2$ $[M+H]^+$ 217.1223 found 217.1220

Mechanistic Investigation

Absorption Spectra

UV−vis absorption spectra were recorded on a Spectrophotometer UV/Vis/NIR Lambda 750 with a resolution of 1 nm. Sample solutions were prepared using degassed DCM, at a concentration of 10^{-5}M by dilution of a 100 µL of a 10^{-3}M solution of the complex in a 10 mL volumetric flask to reach a concentration of $1*10^{-5}$ M. The measures were performed with cells of optical path of 1 cm.

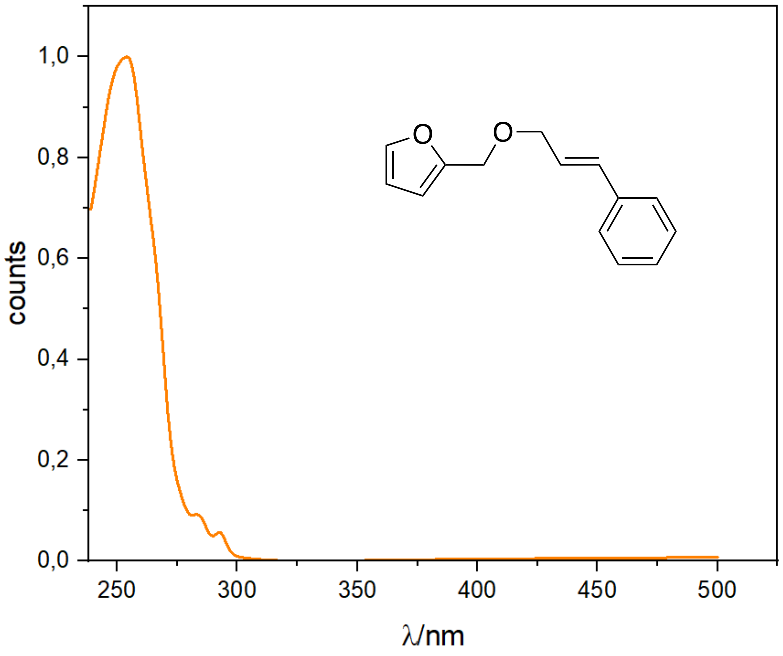

Absorption spectrum of **1a** in DCM

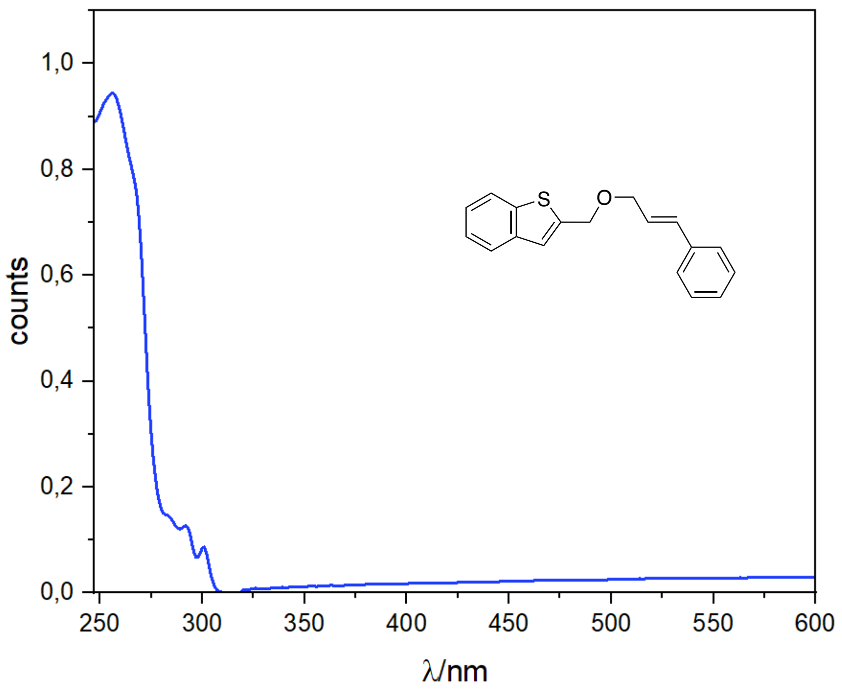

Absorption spectrum of **3c** in DCM

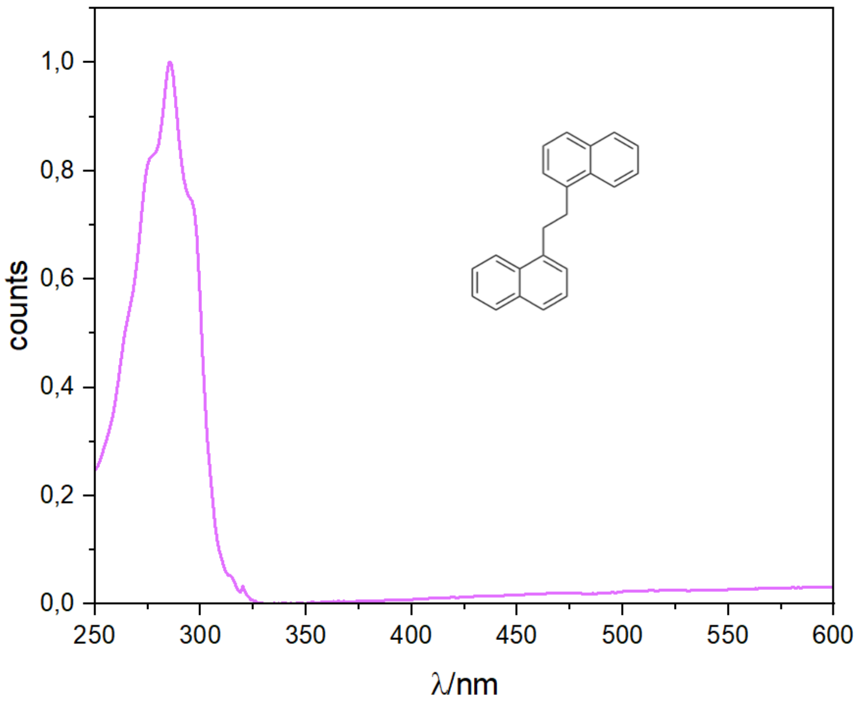

Absorption spectrum of **BiNapht8** in DCM

Emission Spectra

Phosphorescence Emission spectra were recorded on a FLS1000 Edinburgh Fluorometer (**PC8-PC11**), with a resolution of 1 nm. Samples were prepared in dry degassed DCM. The solutions were prepared at a concentration of 10^{-5} M by dilution of 100 μL of a 10^{-3}M solution of the complex in a 10 mL volumetric flask. A quartz cuvette (optical path = 1 cm) capped with a rubber septum was used for the acquisition. The samples solutions were deoxygenated by bubbling a stream of nitrogen for 10 minutes prior to the acquisition of the spectra.

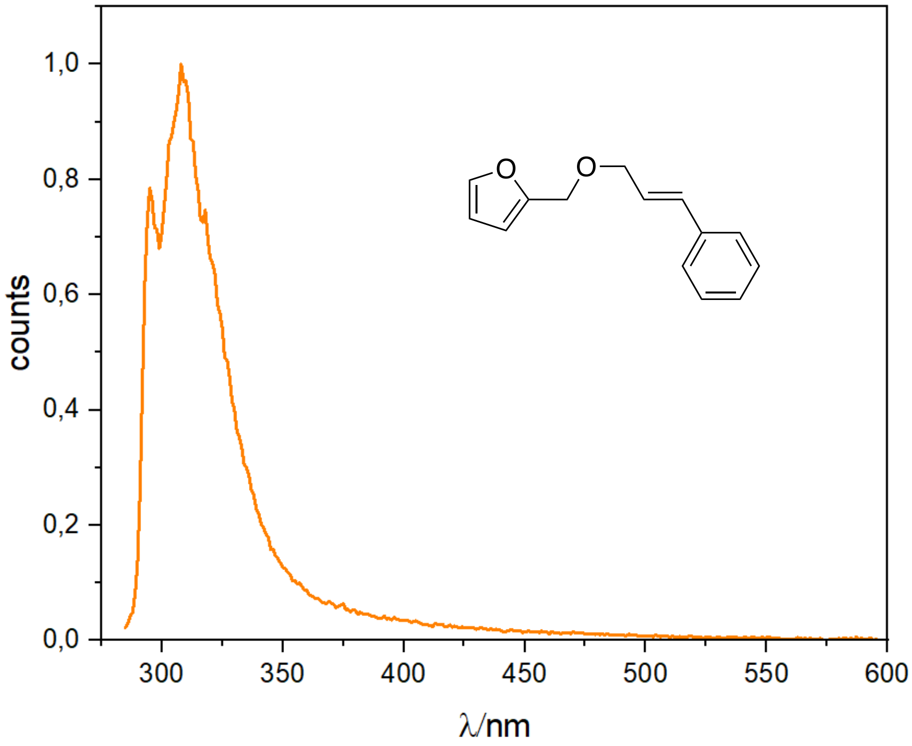

Emission spectrum of **1a** in DCM

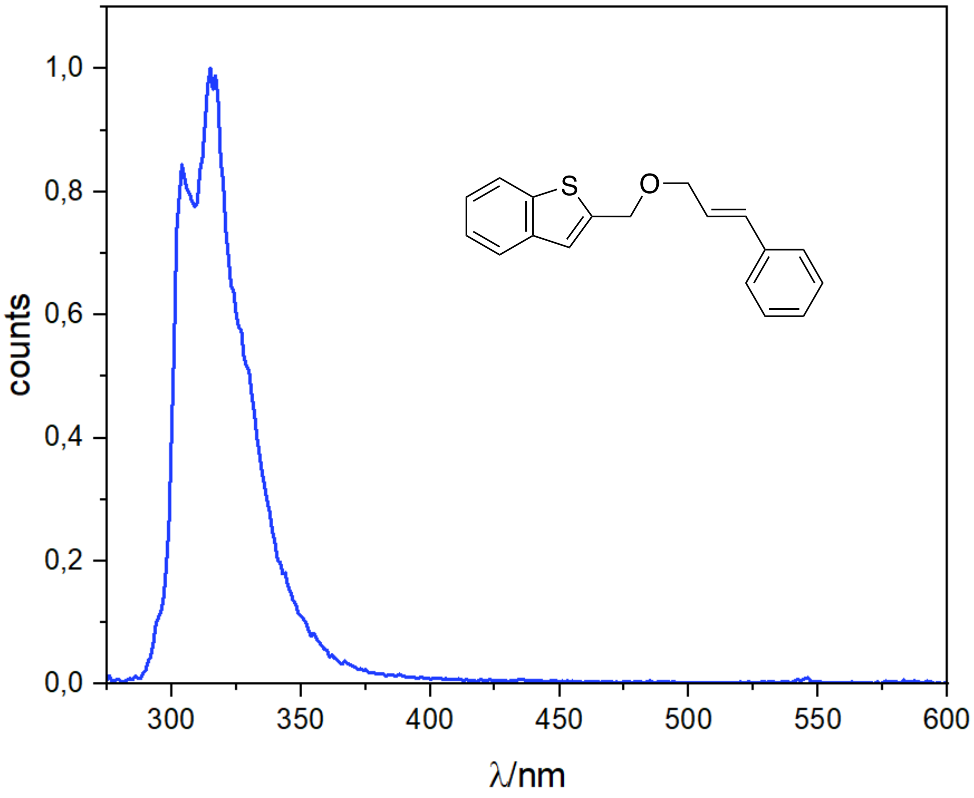

Emission spectrum of **3c** in DCM

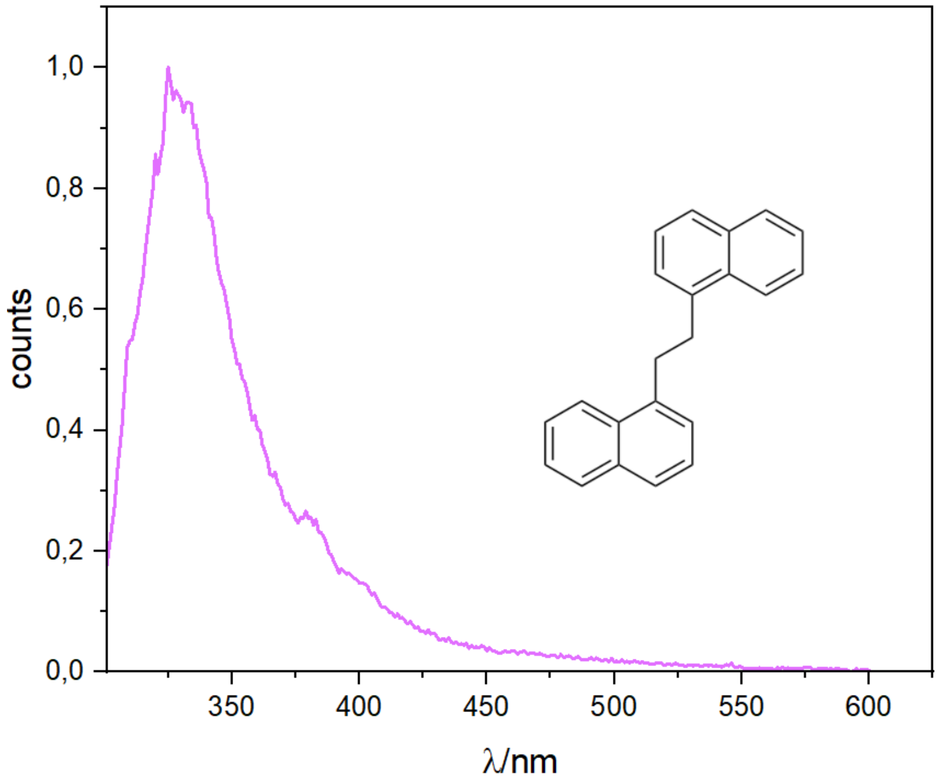

Emission spectrum of **BiNapht 8** in DCM

Cyclic Voltammetry Experiments

All the spectra were measure with IKA ElectraSyn 2.0 in dry and degassed CH_3CN 2mM in CH_3CN with $TBAPF_6$ 0.1M as electrolyte using a glassy carbon disc working electrode, a platinum plate counter electrode and Ag/AgCl as reference electrode.

Conditions: 3 Segments, Initial Voltage: 0 mV, Direction: rising, Upper Voltage: 2.5V, Lower Voltage: - 2.5V, Final Voltage: 0 mV, Sweep: 600 (mV/s), room temperature

In every case examined throughout the study, no oxidation peaks were detected that would indicate the possibility of a direct oxidation pathway. The absence of observable oxidation signals strongly suggests that the photocatalyst, under the experimental conditions applied, does not possess the necessary oxidative potential to initiate or sustain a direct electron-transfer mechanism.

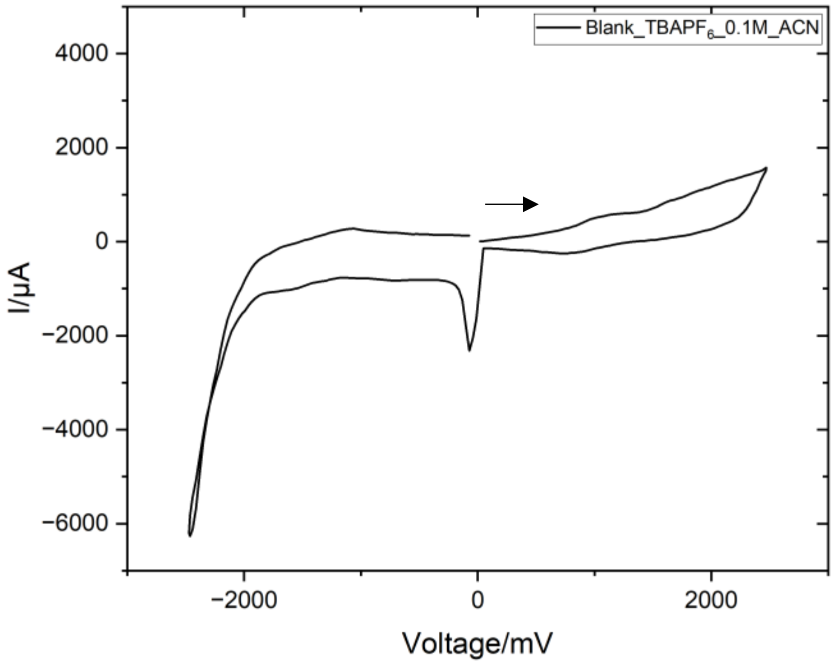

IUPAC plotting convention: $TBAPF_6$ 0.1M as electrolyte in CH_3CN, Ag/AgCl as reference electrode

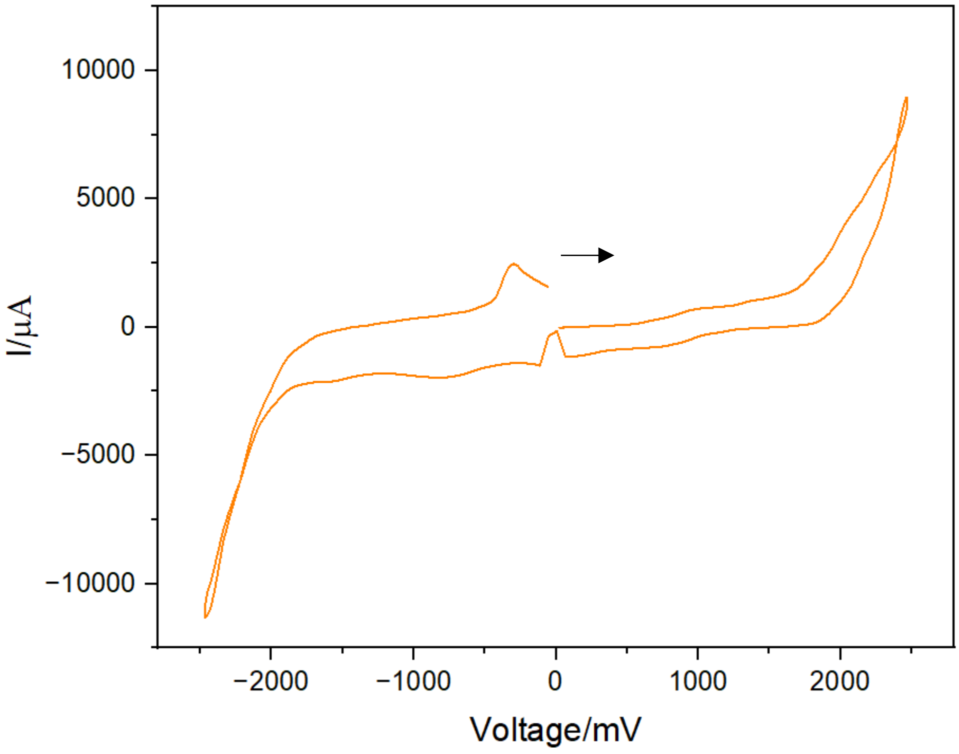

IUPAC plotting convention: **1a** (2mM) in CH_3CN with $TBAPF_6$ 0.1M as electrolyte

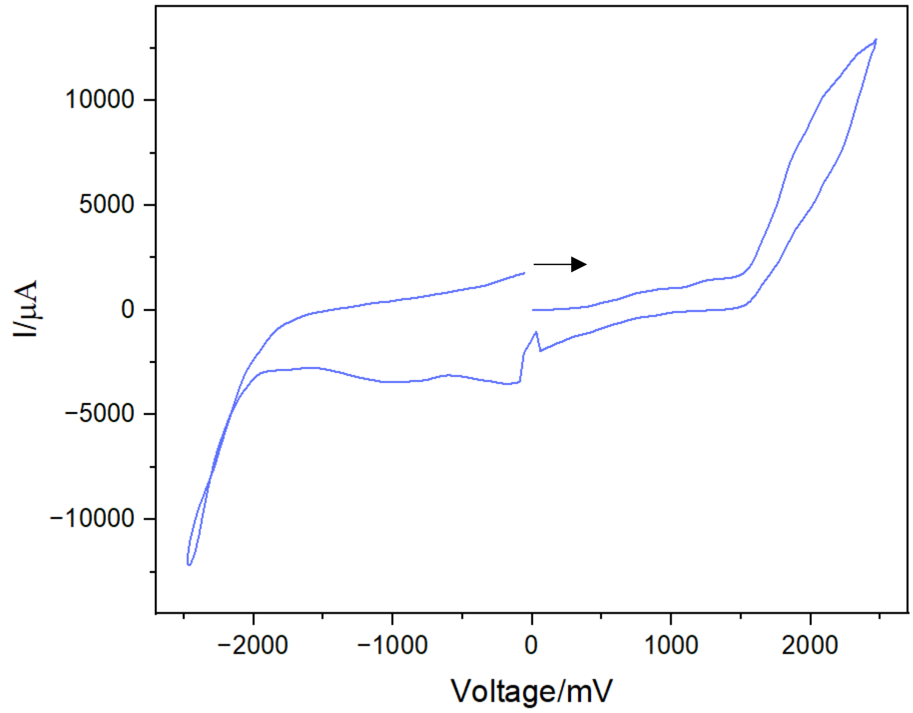

IUPAC plotting convention: **3a** (2mM) in CH_3CN with $TBAPF_6$ 0.1M as electrolyte

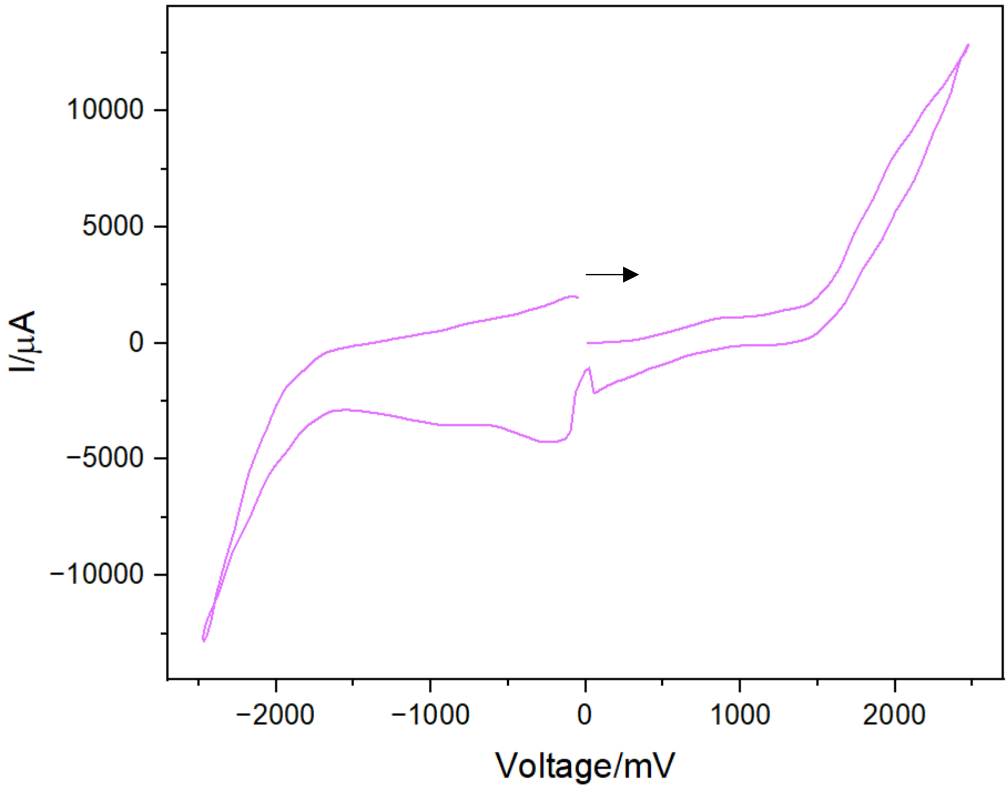

IUPAC plotting convention: **BiNapht 8** (2mM) in CH_3CN with $TBAPF_6$ 0.1M as electrolyte.

Stern-Volmer quenching studies

The measurements of fluorescence emissions were carried out with a FLS1000 Edinburgh fluorometer, equipped with automatic polarizers. Emissions have been collected by exciting the sample with a Xenon lamp at 380 nm and the luminescence was measured at 480 nm. Fluorescence spectra are corrected for the excitation intensity and detector sensitivity.

Stern–Volmer quenching studies were carried out using a 10^{-5} M solution of **PC2** in dry and degassed DMF and variable concentrations of **BiNapth8**, **1a**, **3c** and **1q** from 0 to 2 mM. 2.5 mL of **PC2** solution was transferred to a 3.5 mL quartz cuvette and degassed twice for 5 minutes (with a 30 second break in between) before collecting the first spectra. Then, after each addition of quencher, the samples were degassed for 3 minutes and spectra were quickly recorded.

Linear Stern–Volmer plots were obtained at varying concentrations of the three substrates, and KSV constants were extracted according to the equation:

$$I_0/I = 1 + K_{SV}[Q]$$

1a and **3c** clearly quench **PC2**, with high K_{sv} and without significant differences. On the other hand, **1q** does not quench **PC2**, with only 2.5 as K_{sv}. Indeed, when **1q** was tested with optimized reaction conditions, it showed no reactivity (recovery of starting material), reasonable because its triplet energy is too high (≈ 75 Kcal/mol).

BiNapht8 resulted in a significant capable quencher for **PC2** with a K_{sv} of 1950. This result is consistent with experimental observations and with the data previously reported by our group.[14] It is indeed demonstrated that the **BiNaphtil** units can quench the **PC** (Triplet Energy of **BiNapht8**= 55.1 Kcal/mol, Triplet Energy of **PC2** = 61.8 Kcal/mol) and thus result in an energy storage.[15]

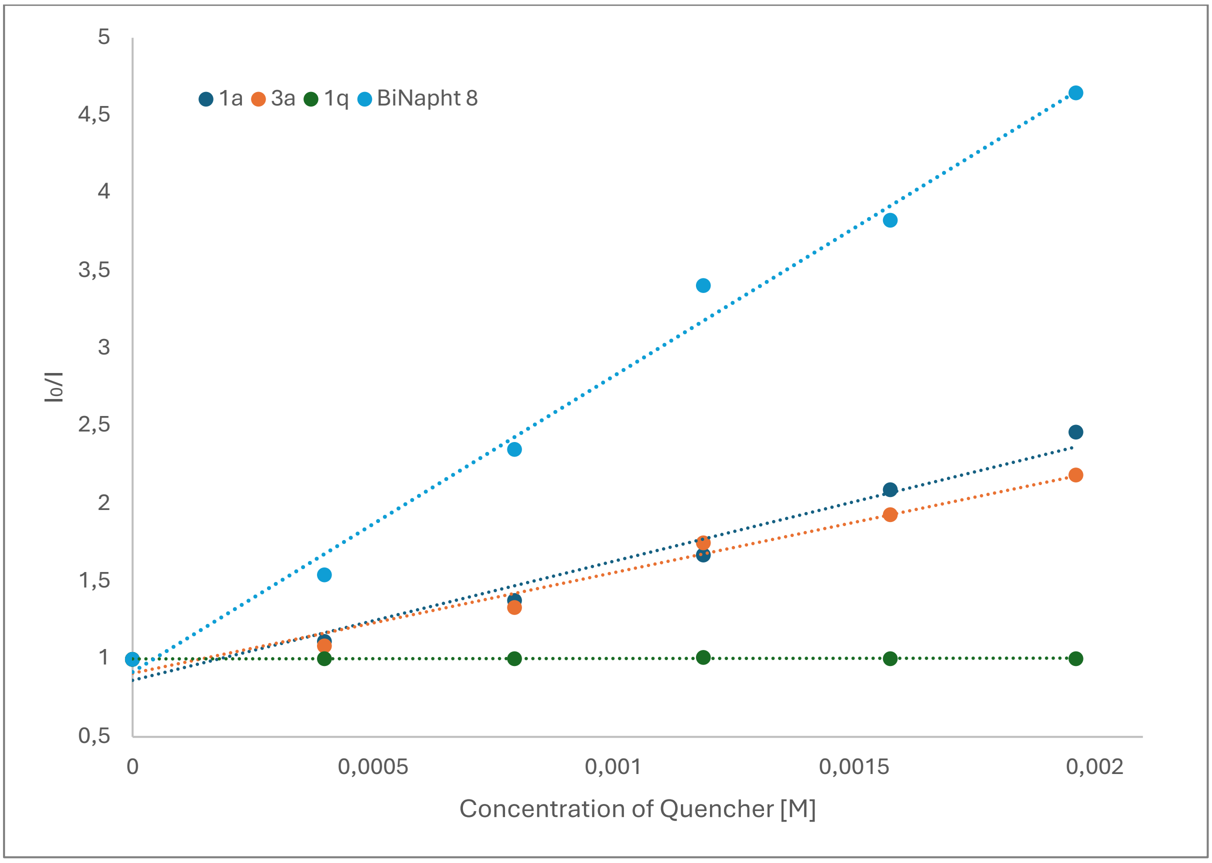

Comprehensive table of Stern – Volmer studies		
**Substrates**	**K_{sv}**	**R2**
**1a**	766.44	0.9690
**3c**	647.58	0.9753
**1q**	2.46	0.2199
**BiNapht 8**	1905.00	0.9908

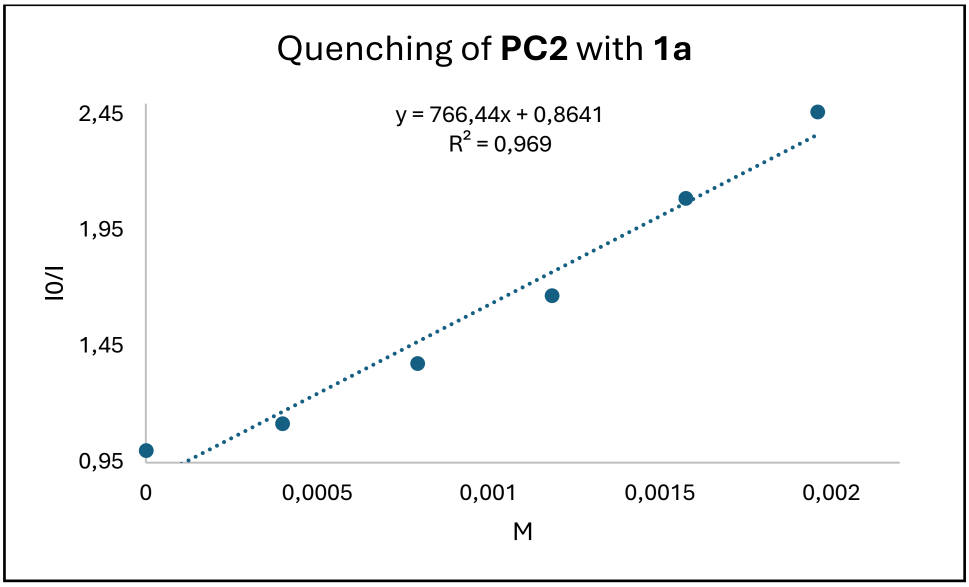

Measured emissions for the quenching of **PC2** with **1a**

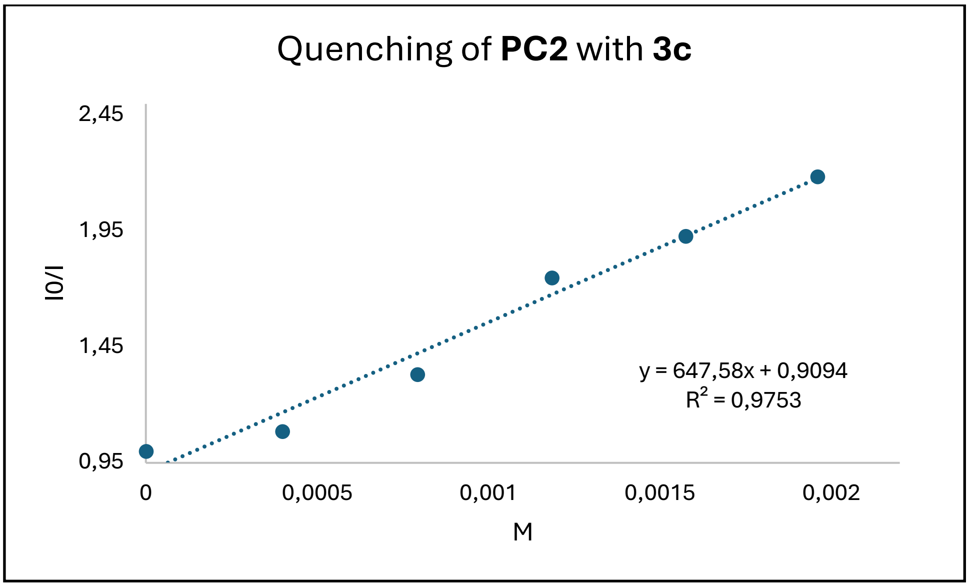

Measured emissions for the quenching of **PC2** with **3c**

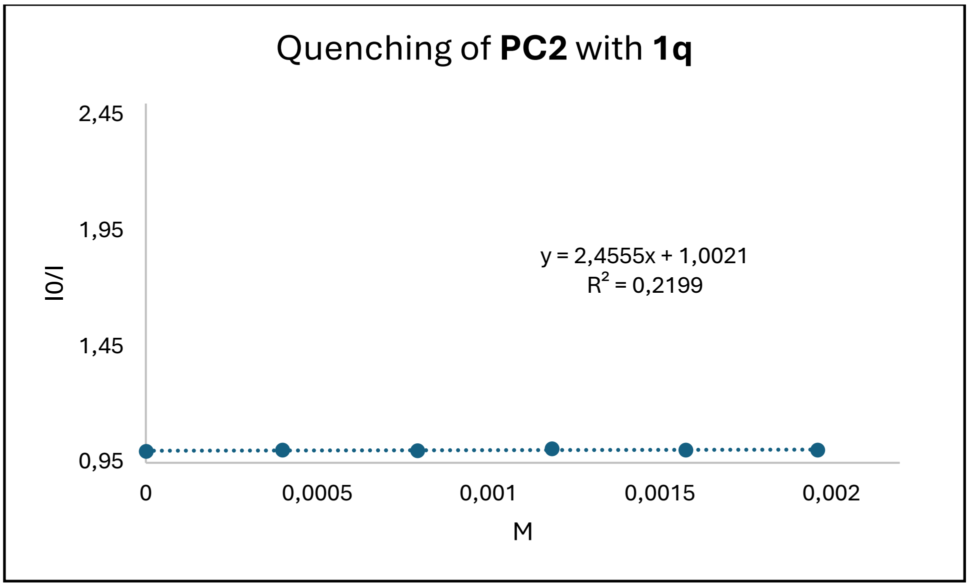

Measured emissions for the quenching of **PC2** with **1q**

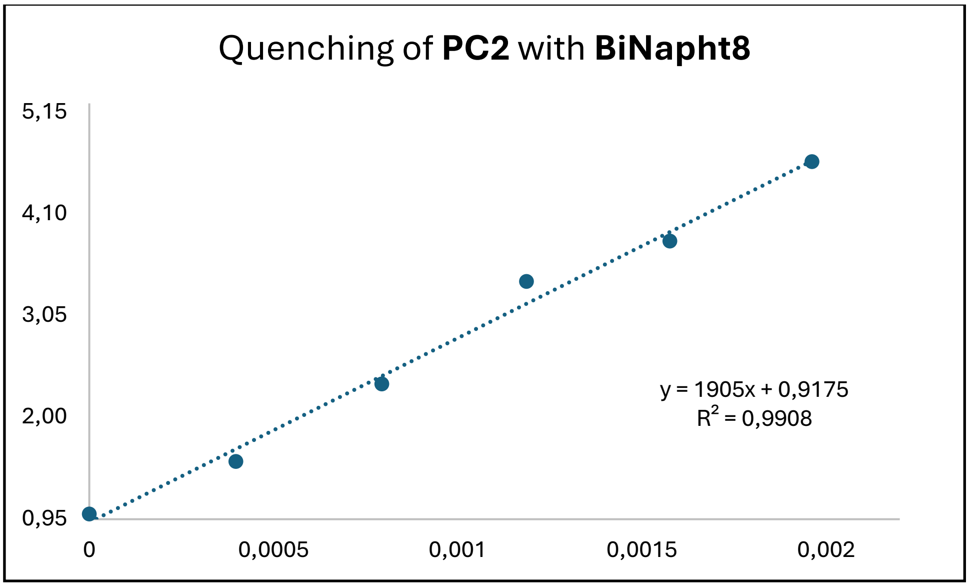

Measured emissions for the quenching of **PC2** with **BiNapht8**

Computational Data

Calculations were performed at the DFT level using Gaussian16.[16] The hybrid exchange/correlation M06 functional, as described by Zhao and Truhlar, was used for geometry optimization.[17] This broadly used functional has already proved to be reliable in related photochemical cascades. Optimization were performed without any constraint using the Def2-SVP basis set described by Weigend and Ahlrichs,[18] using DCM as implicit solvent through the CPCM method introduced by Barone and Cossi.[19] Free optimization was then performed again using the Def2-TZVP basis set in order to achieve more precise results.[20] In both cases, the model was used in combination with D3 corrections, as described by Grimme,[21] in order to take into account dispersion interactions.
All values and geometries reported hereafter are those of freely optimized solvated structures, calculated at the M06/Def2-TZVP level. All intermediates, both in their ground and higher spin states were characterized by the absence of any imaginary frequency in their Hessian matrix. All transition states were characterized by the presence of a single imaginary frequency in their Hessian matrix, which corresponds to the molecular vibration connecting the reactant with the product.

Screening of functionals for the calculation of ^{3}[1a] energy

To improve the modeling of the reaction outcome, various functionals commonly used to describe light-mediated reactions and energy transfer processes were tested at the outset of our DFT investigation.

Table S5

		**H** (Hartree/Particle)	**S** Cal/Mol-Kelvin
M06-D3 Def2-TZVP	**^{1}[1a]**	-848,51	132,32
	**^{3}[1a]**	-848,42	137,29
M06 Def2-TZVP	**^{1}[1a]**	-691,83	121,08
	**^{3}[1a]**	-691,75	126,04
B3LYP Def2-TZVP	**^{1}[1a]**	-692,36	129,49
	**^{3}[1a]**	-692,28	127,88
wB97XD Def2-TZVP	**^{1}[1a]**	-692,09	127,80
	**^{3}[1a]**	-692,01	126,91

**Functional and Base sets**	**Calculated triplet energy of 1a**
M06-D3 Def2-TZVP	55.1 Kcal/mol
M06 Def2-TZVP	47.0 Kcal/mol
B3LYP Def2TZVP	50.7 Kcal/mol
wB97XD Def2TZVP	50.6 Kcal/mol

The results showed no significant differences among the tested functionals, therefore, the M06-D3 functional was selected in combination with the Def2-TZVP basis set.

Summary of Energies

Table S6

	**H** (Hartree/Particle)	**S** Cal/Mol-Kelvin
**^{1}[1a]**	-691,83	128,24
**^{3}[1a]**	-691,754115	126,116
**^{3}[Ia]**	-691,78	118,52
**^{1}[2a]**	-691,83	110,33
**^{3}[Ia’]**	-691,78	118,77
**^{1}[2a’]**	-691,82	110,14
**^{1}[BiNapht8]**	-848,51	132,32
**^{3}[BiNapht8]**	-848,42	137,29
**^{1}[1a:BiNapht8]**	-1540,37	210,04
**^{3}[1a:BiNapht8]**	-1540,29	215,94
**^{3}[Ia:BiNapht8]**	-1540,31	202,57
**^{1}[2a:BiNapht8]**	-1540,36	196,44

Computational study on Transition States (TS)

To investigate the thermodynamically favorable formation of the new carbon–carbon bond leading to the product 2**a**, a comprehensive DFT computational study was conducted. A relaxed potential energy surface (PES) scan was performed using the Gaussian software package, employing the M06 functional with GD3 dispersion correction and the def2-SVP basis set (Opt=ModRedundant). This setup enabled a detailed and flexible exploration of molecular geometries by varying selected internal coordinates, providing insights into the energetic profile associated with structural changes or chemical transformations. The calculations were performed both for the triplet of the substrate (^{3}**1a**) and for the triplet of the bimolecular adduct with **BiNapht8** ([^{3}**1a:BiNapht8**]). The plot of these two scans are shown hereafter.

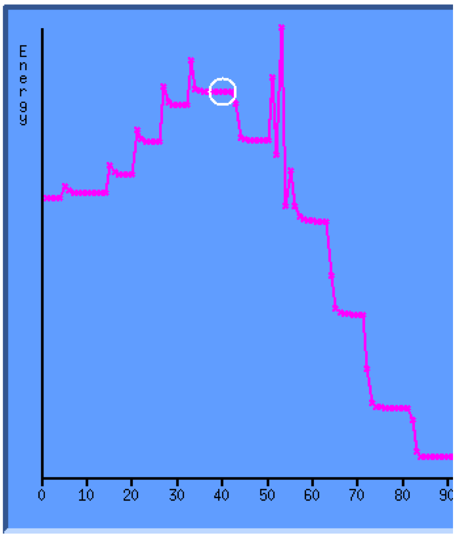

Energy profile for the relaxed PES scan for the formation of ^{3}**Ia** from ^{3}**1a**

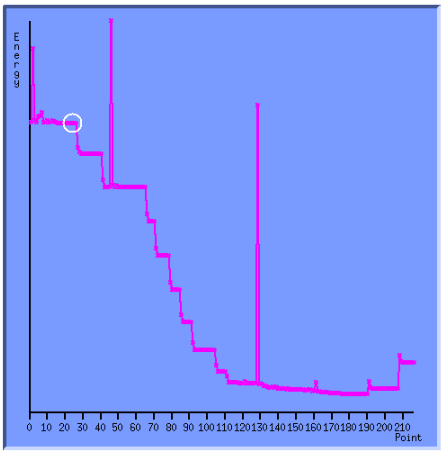

Energy profile for the relaxed PES scan for the formation of ^{3}[**Ia:BiNapht8**] from ^{3}[**1a:BiNapht8**]

These scans are used to find the approximate geometry of the transition states (TS) involved in the process, which are then freely optimized. In both cases, the point of the PES circled in white in Figure S2 was selected as approximate geometry for the transition state (TS) optimization.

Subsequent frequency analysis confirmed the nature of the two stationary points by revealing a single imaginary frequency, consistent with a first-order saddle point.

In the first case, the relaxed energy scan provided an approximate ΔE value of ca. 6 kcal/mol, which indicates that the process should be highly favorable from a kinetic point of view; the free optimization of this TS was carried out, and the evaluation of the energy of the resulting stationary point indicated that the step is essentially barrierless.
In particular, the calculated Gibbs free energy for the **TS(^{3}[1a-Ia])** is +49.52 Kcal/mol above the entry channel, while ^{3}[**1a**] lies at +49.06 Kcal/mol. Therefore, the calculated barrier is +0.46 kcal/mol in ΔG, which can be considered an essentially barrierless step.

A similar scenario emerged analyzing the second process on the bimolecular adduct; in this case, the energetic profile of the scan already showed that the cyclization should be essentially barrierless; this finding was confirmed by freely optimizing this putative TS, too. Indeed, the stationary species found, **TS(^{3}[1a-Ia]:BiNapht8)**, lied even below the parent intermediate [^{3}**1a:BiNapht8**] in ΔG.

Summary of the energy for the transition state

Table S7

	**H** (Hartree/Particle)	**S** Cal/Mol-Kelvin	**Freq**
**TS(^{3}[1a-Ia])**	-691.76	119.69	-525.86
**TS(^{3}[1a-Ia]:BiNapht8)**	-1540.30	204.73	-340.77

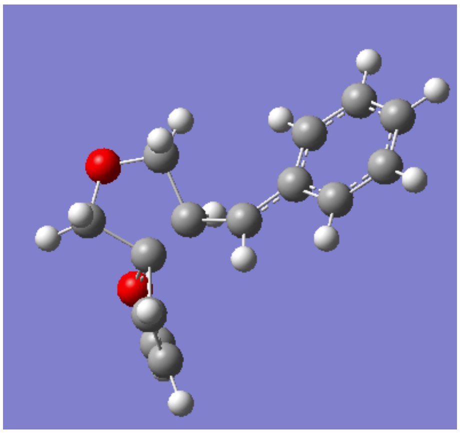

Figure S1: The structure of the transition state (**Ts(^{3}[1a]-^{3}[Ia]**))

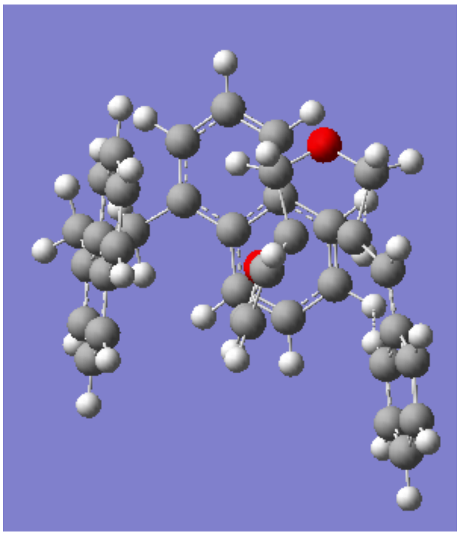

Figure S2: The structure of the transition state (**Ts(^{3}[1a:BiNapht8]-^{3}[Ia:BiNapht8]**))

Comprehensive energy profiles

The reaction coordinate has been constructed by computing and connecting the relevant stationary points along the reaction pathway, both for the system involving only substrate **1a** and for the system in the presence of **BiNapht8**.
Two alternative or parallel reaction pathways can be envisioned based on the previously reported DFT results.

As illustrated in **Figure S3**, upon irradiation of photocatalyst **PC2** with purple light, its excited state can be populated, reaching an energy level of 60.1 kcal/mol. This acts as an energy donor and transfers its energy to **BiNapht8** through a thermodynamically favorable energy transfer (EnT) process, thereby populating its triplet excited state ^{3}[BiNapht8], while simultaneously regenerating the ground state of **PC2**. The ^{3}[**BiNapht8**] is thermodynamically competent to activate substrate **1a** through a subsequent EnT. This reaction pathway is supported by two key considerations: (1) the thermodynamic favorability of the successive energy transfer steps, and (2) the statistical preference for the population of ^{3}[**1a**] via energy transfer from ^{3}[**BiNapht8**], compared to direct excitation by ^{3}[**PC2**], due to higher concentration of ^{3}[**BiNapht8**] (30 mol%) in solution if compared to ^{3}[**PC2**] (1 mol%). It is important to consider that experimental observations have demonstrated the formation of **2a** even in the absence of **BiNapht8** (Table S2, Entry 16). This suggests that a parallel mechanism may be operative in which **1a** is directly excited by **PC2** itself, without the mediation of **BiNapht8**. Therefore, a direct excitation pathway cannot be definitively excluded and must be considered a plausible alternative under the reaction conditions.

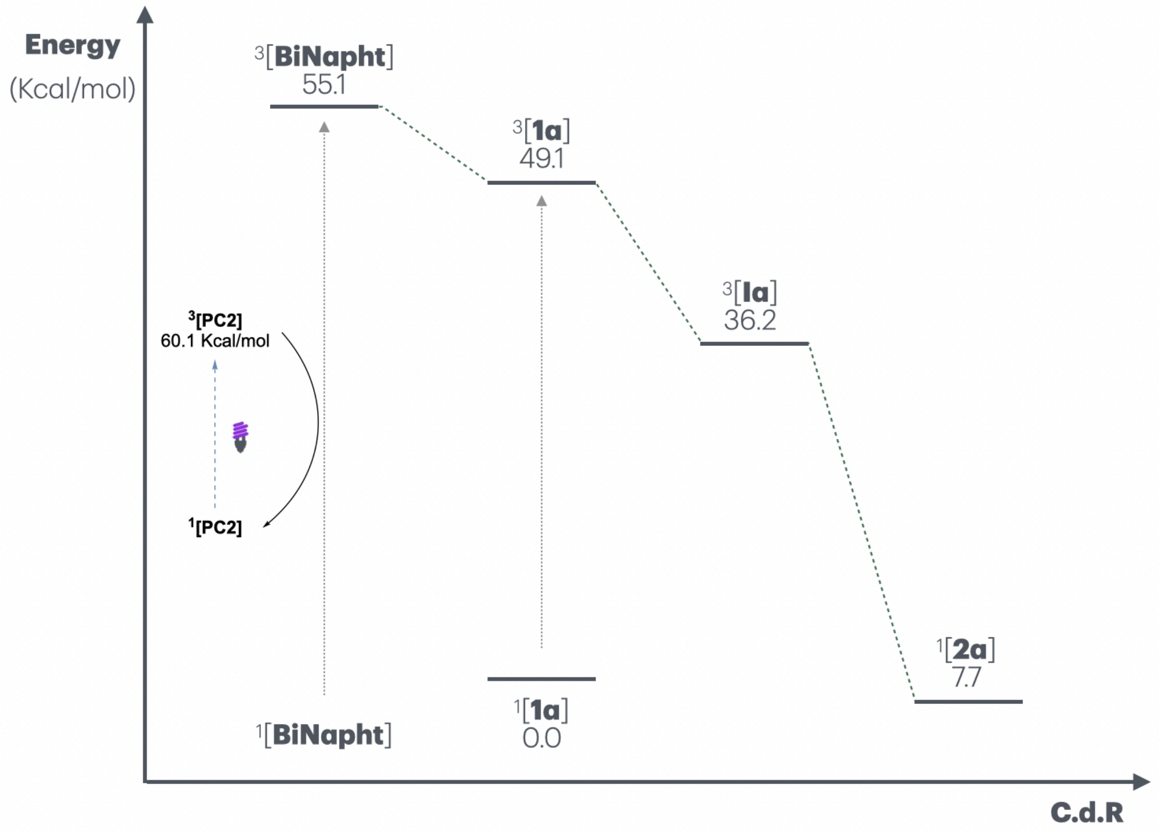

Figure S3: Relay reaction pathway

Alternative mechanistic investigations have revailed a possible complemental pathway, which is shown in **Figure S4**. In the ground state, **BiNapht8** and **1a** can reversibly associate to form the non-covalent [**BiNapht8**:**1a**] complex. The Gibbs free energy change for this association is +0.8 Kcal/mol, indicating a weakly endergonic process. This small energy cost is consistent with the predominance of weak π–π stacking and dispersion interactions between the chromophores in solution (See next section). Upon photoexcitation of the photocatalyst **PC2** (ET = 60.1 kcal/mol), both **1a** and **BiNapht8** (ET = 49.1 and 55.1 kcal/mol, respectively) can undergo an exoergonic energy transfer step (vide supra). Importantly, the calculated triplet energy of the [^{3}**1a:BiNapht8**:**1a**] complex is slightly lower (ET = 48.0 kcal/mol) than that of these two isolated species. This suggests that the sensitization of the bimolecular adduct is more favorable. This difference likely arises from enhanced stabilization through dispersion interactions in the triplet-state adduct ^{3}[**1a**:**BiNapht8**], as noted in prior literature. Following the EnT event, the reactive triplet species undergoes a *5-exo-trig* cyclization. Computational modeling reveals that this step is essentially barrierless (See previous section) for both free ^{3}[**1a]** and the complexed ^{3}[**1a**:**BiNapht8**], indicating that once the substrate is activated, the first C-C bond forming event is a rapid and favorable event. After cyclization, the reaction proceeds through intersystem crossing (ISC) and subsequent radical recombination to afford the product **2a**. This product has higher free energy relative to the starting material **1a** (ΔG = +7.7 kcal/mol), reflecting the endoergonic nature of the dearomative transformation. This energetic penalty is attributed to the loss of aromatic stabilization, a factor commonly observed in such transformations.

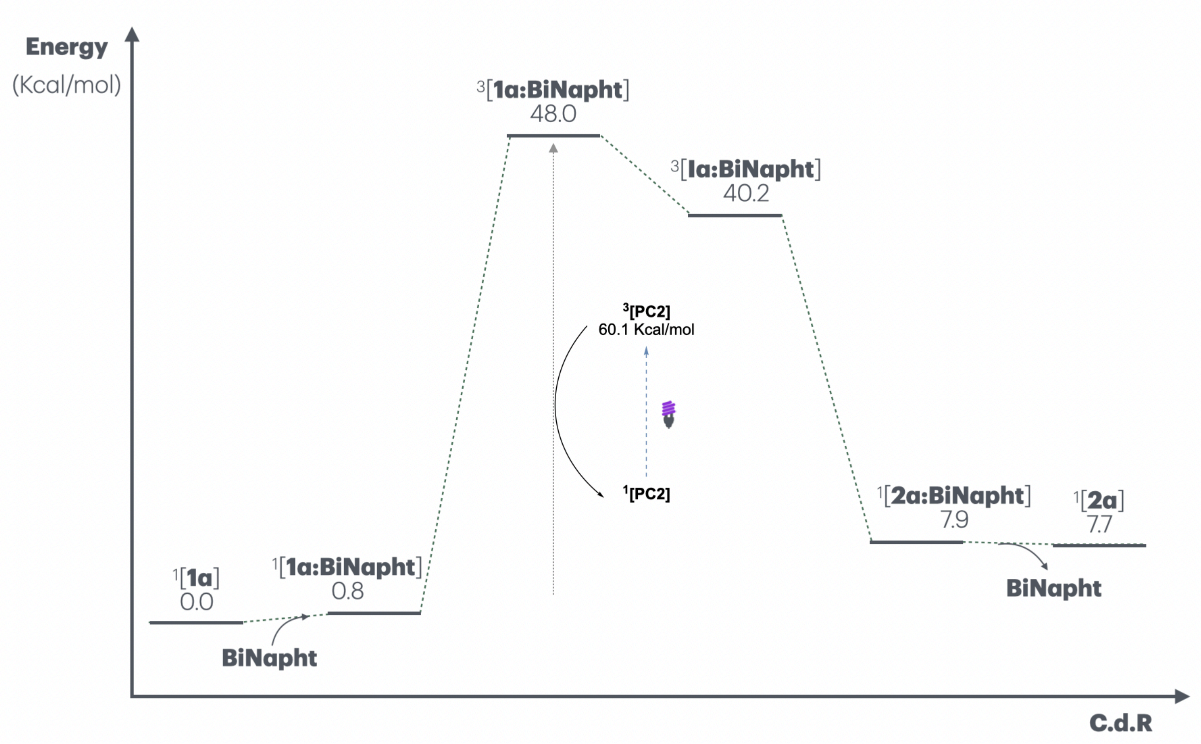

Figure S4: Complexation reaction pathway

Intermolecular Non-Covalent Interaction for [1a:BiNapht8]

The visualization and qualitative analysis of dispersion forces within intermolecular adducts were carried out through the application of the Non-Covalent Interaction (NCI) plot tool.[22] The NCI method serves as detecting and interpreting weak intermolecular interaction. We employed this tool to explore and elucidate the nature of dispersion forces in the intermolecular adducts formed by **[1a:BiNapht8]**. Our analysis was conducted for both electronic configurations, the singlet and the triplet spin states, to reveal how these weak forces behave and differ under varying spin multiplicities. This dual-state investigation allows for a more comprehensive understanding of the role and modulation of dispersion interactions in these systems.
A pronounced intermolecular interaction is observed in the case of 3[1a:BiNapht8] as supported by DFT calculations energies.

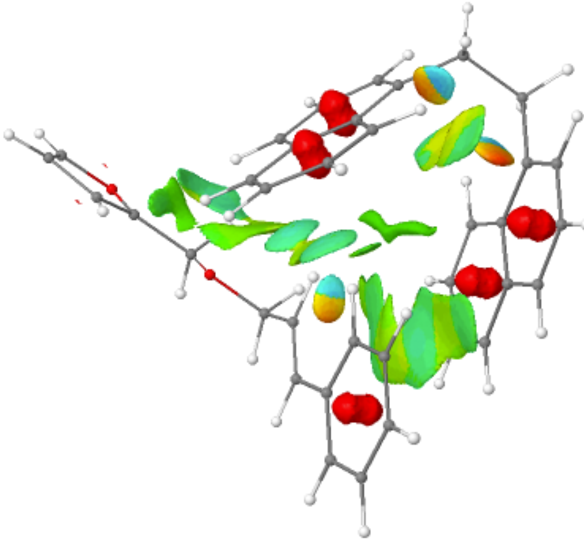

NCI visualization of ^{1}[**1a:BiNapht8**]

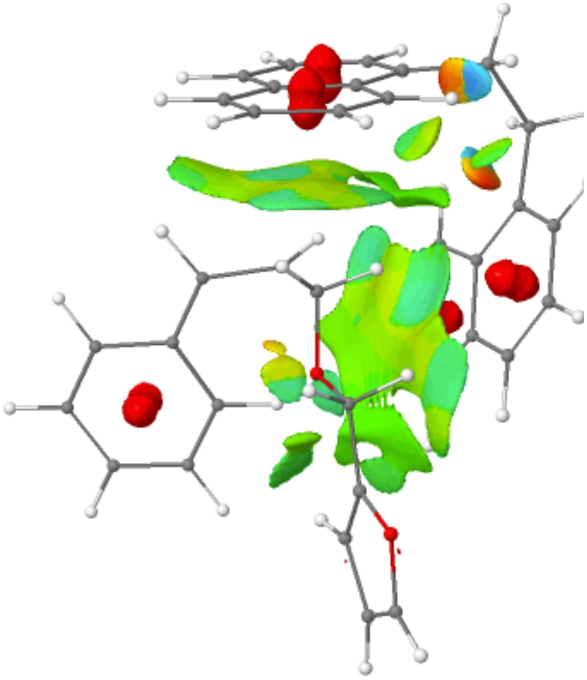

NCI visualization of ^{3}[**1a:BiNapht8**]

Conformational study of ^{3}[1a:BiNapht8] complex

For these reactions, we carried out a preliminary conformational analyses on ground-state species for 1a, BiNapht8, and their bimolecular adduct. At least three different conformers were freely optimized in each case, and the most stable one was then used as entry channel for the following steps of the computational study. Among these cases, differences remained within 3 kcal/mol, suggesting that all of the tested conformers are likely in equilibrium.
In particular, the result shown hereafter are dedicated to three different bimolecular adducts between the substrate and the cocatalyst, which were freely optimized. Differences between them remained narrow, and the most stable one was the species used as entry channel for the analysis of the reaction pathway.

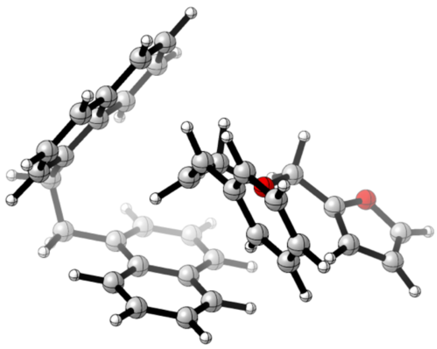

Conf. I

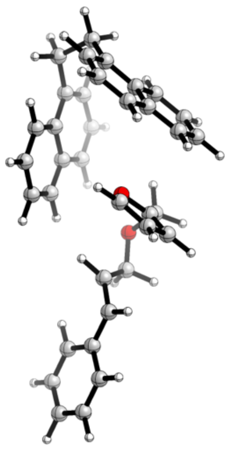

Conf. II

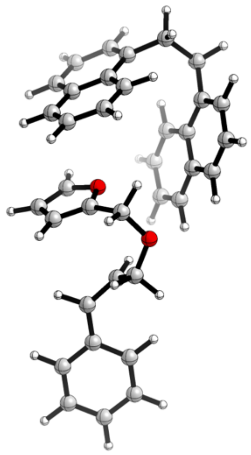

Conf. III

Table S7

	**H** (Hartree/Particle)	**S** Cal/Mol-Kelvin
**Conf. I**	-1540.286293	215.940
**Conf. II**	-1540.282071	212.302
**Conf. III**	-1540.282207	212.334

Investigation on the stereo and regio selectivity of the reaction

The transformation of compound **1a** into the products **2a**, **2a’** and **2a’’** was modeled to gain a deeper understanding of the regio- and stereoselectivity observed in the reaction. Geometry optimizations and energy calculations were performed at the M06-GD3 level using the Def2-TZVP basis set and the Self-Consistent Reaction Field (SCRF) method to account for the solvation effects of dichloromethane (DCM).

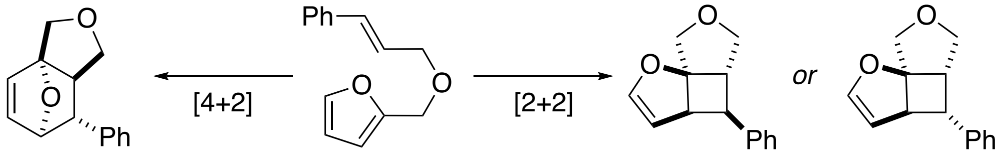

2a’, +21.6 **1a**, 0.0 **2a**, +7.70 **2a’’**, +11.20

Under the applied reaction conditions, only two products, **2a** and **2a′**, were detected. Among these, product **2a** was the only compound successfully isolated, as **2a′** rapidly decomposes, rendering it unsuitable for isolation. Notably, the product **2a″** was not observed at all during the course of the reaction. This absence is consistent with computational data, which indicates that **2a″** is considerably less stable than its peers.

Similarly, computational studies have been performed to gain a deeper understanding of the outcome using substrate **1o**. Geometry optimizations and energy calculations were performed as presented before (M06-GD3/Def2-TZVP, SCRF=DCM). Under the applied reaction conditions, only compound **2o** was isolated in moderate yield (35%), while the formation of **2o’** was not observed. This is in line with computational data, which show that **2o’** has a significantly higher associated energy (+12.45 kcal/mol) compared to **2o** (+9.04 kcal/mol), rendering its formation thermodynamically unfavorable.

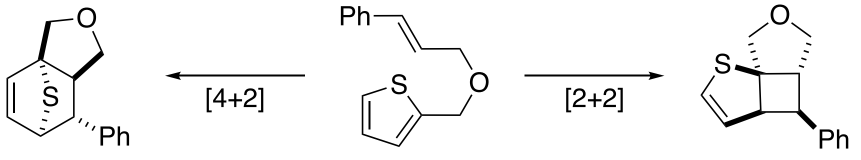

2o', 17.42 **1o**, 0.0 **2o**, 9.04

To simplify the computational process, we chose to investigate the transformation of compound **z** rather than that of **1p**. This was made to facilitate computational analysis while preserving the structure and electronic characteristics associated with the sulfonamide. The transformation of **z** into the products **z’** and **z’’** was modeled analogously. Geometry optimizations and energy calculations were performed as previously described (M06-GD3/Def2-TZVP, SCRF = DCM).

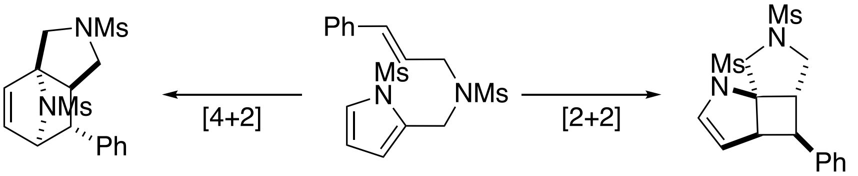

z’, 17.42 **z**, 0.0 **z’’**, 5.73

Under the applied reaction conditions, only product **z'** was observed and isolated (55% yield), while **z''** was not detected. On the contrary, computational data show that **z''** is associated with a relative lower energy (5.73 kcal/mol) if compared to **z'** (17.42 kcal/mol), rendering the formation of the desired product formation thermodynamically unfavorable. This supports the existence of a diradical intermediate along the reaction coordinate, which appears to facilitate the formation of the [4+2] product, thereby rationalizing the experimentally observed selectivity.

Cartesian Coordinates of Optimized Structures

^{1}**[1a]**

30
scf done: -692.074470

C	-1.686687	-2.043807	-0.513483
C	-1.211630	-3.275283	-0.791732
C	0.186680	-3.238829	-0.503452
C	0.445680	-1.984463	-0.072851
O	-0.684133	-1.241118	-0.072342
C	1.674024	-1.296503	0.380822
O	2.746154	-2.186144	0.289104
H	1.548674	-0.939003	1.417226
H	1.852536	-0.398129	-0.234195
C	3.956306	-1.604656	0.720843
C	5.028328	-2.626351	0.683265
H	3.825810	-1.221097	1.746973
H	4.217778	-0.743828	0.086591
C	6.178651	-2.457975	0.038925
H	4.822552	-3.540919	1.234775
H	-2.663672	-1.593238	-0.571312
H	-1.782742	-4.112853	-1.158755
H	0.905615	-4.035313	-0.602484
C	7.302799	-3.387448	-0.025502
H	6.319737	-1.529843	-0.514277
C	8.391044	-3.070712	-0.837238
C	7.341090	-4.587899	0.686572
C	8.423450	-5.438738	0.580576
C	9.496943	-5.111067	-0.236298
C	9.477081	-3.921791	-0.944473
H	8.376718	-2.138707	-1.393476
H	10.310793	-3.654330	-1.583317
H	10.345361	-5.780510	-0.315706
H	6.515239	-4.857713	1.335392
H	8.433791	-6.365981	1.141899

^{3}**[1a]**

30
scf done: -691.995104

C	0.072430	-0.345893	0.024369
C	0.484967	0.286074	1.220833
C	1.875809	0.417693	1.442132
C	2.788252	-0.047585	0.524286
C	2.357873	-0.663508	-0.648267
C	0.994506	-0.808634	-0.886734
C	-0.442348	0.771159	2.161556
C	-1.880991	0.715158	1.999006
C	-2.627202	1.728915	1.203333
O	-3.897609	2.041549	1.734118
C	-3.855171	2.958097	2.806824
C	-3.092047	2.484287	3.981373
O	-3.565758	1.393304	4.634787
C	-2.683235	1.106534	5.618203
C	-1.668877	2.000307	5.623036
C	-1.937727	2.902068	4.554101
H	-3.410111	3.909607	2.481868
H	-4.898543	3.147273	3.073132
H	-2.015825	2.640029	1.096087
H	-2.821132	1.359521	0.185881
H	-2.455379	-0.125149	2.387314
H	-0.833127	2.015102	6.303783
H	-1.343973	3.743589	4.231551
H	-0.041708	1.240924	3.062614
H	2.212479	0.898014	2.355503
H	3.849429	0.066072	0.715278
H	3.079579	-1.028512	-1.369267
H	-0.990193	-0.468507	-0.162344
H	0.655217	-1.290891	-1.796596
H	-2.916912	0.253581	6.234305

^{3}**[Ia]**

30
scf done: -692.019879

C	-0.078126	-0.394152	1.357105
C	0.229525	0.961456	1.095648
C	1.567738	1.267481	0.754782
C	2.529074	0.286753	0.680567
C	2.201376	-1.040548	0.942295
C	0.892490	-1.368056	1.280115
C	-0.728106	1.987746	1.168953
C	-2.149492	1.797230	1.527700
C	-3.062370	2.932529	1.066256
O	-4.101333	3.004429	2.015683
C	-3.449269	2.858439	3.249870
C	-2.447161	1.718981	3.069774
O	-3.102588	0.457686	3.344785
C	-2.441090	-0.145429	4.359058
C	-1.358567	0.591186	4.781675
C	-1.303507	1.736858	4.018516
H	-2.893273	3.775160	3.506170
H	-4.175838	2.648100	4.035544
H	-2.500330	3.880850	1.048534
H	-3.496855	2.768799	0.079958
H	-2.524085	0.851994	1.121936
H	-0.681745	0.299645	5.570129
H	-0.589663	2.545088	4.074660
H	-0.387892	3.005221	0.985316
H	1.825920	2.301958	0.550954
H	3.547150	0.550129	0.416974
H	2.959616	-1.812309	0.884206
H	-1.090899	-0.670192	1.630279
H	0.631674	-2.399822	1.487245
H	-2.829705	-1.097032	4.687377

[2a]

30
scf done: -692.074268

O	-1.640813	3.714465	10.149196
O	-0.369144	2.714630	7.004081
C	2.198941	5.454667	9.306572
C	1.333606	4.314386	8.878943
H	1.878513	3.708128	8.144055
C	-0.612694	3.425109	9.210849
C	-0.090298	4.588493	8.342019
H	-0.498952	5.564654	8.604982
C	2.037268	6.731174	8.783653
H	1.248517	6.920869	8.062789
C	0.735939	3.371546	9.955534
H	1.236310	2.404351	10.072793
C	-0.373349	4.134842	6.932271
H	-1.355845	4.495121	6.595046
H	0.381558	4.437654	6.203005
C	3.226056	5.240455	10.221919
H	3.367942	4.246860	10.637105
C	-1.018907	2.369205	8.208953
H	-0.723943	1.354963	8.483958
H	-2.113570	2.402651	8.095918
C	3.889667	7.542691	10.079711
H	4.542652	8.353089	10.381921
C	2.875621	7.767684	9.165122
H	2.732444	8.756785	8.745007
C	0.326203	3.990018	11.257736
H	0.985390	4.234139	12.076797
C	4.063153	6.271832	10.607216
H	4.854756	6.084555	11.323863
C	-0.994362	4.122198	11.277227
H	-1.638827	4.490536	12.063710

^{3}[**Ia’’**]

30
scf done: -692.020937

O	-0.134069	-0.062820	0.321628
C	-0.283188	-0.421833	1.716767
C	0.882872	-1.298637	1.990186
C	1.596244	-1.435691	0.818962
C	0.966088	-0.691445	-0.151456
C	-0.423548	0.841399	2.546900
O	-1.780896	1.229796	2.484956
C	-2.550735	0.199500	1.889099
C	-1.684429	-1.048149	1.911263
C	-2.034978	-2.074779	0.907698
C	-1.892595	-3.463573	1.052996
C	-2.248255	-4.314559	-0.021910
C	-2.126838	-5.680888	0.074127
C	-1.647330	-6.264257	1.243225
C	-1.290123	-5.451477	2.315073
C	-1.405349	-4.082910	2.229151
H	0.218012	1.639205	2.162247
H	-0.132909	0.629720	3.584555
H	-2.802970	0.465894	0.852997
H	-3.482553	0.079779	2.447028
H	-1.684064	-1.464501	2.925725
H	1.202327	-0.528980	-1.191654
H	2.493501	-2.015786	0.666475
H	1.087422	-1.735957	2.956138
H	-2.400750	-1.711378	-0.049886
H	-2.622922	-3.862572	-0.934830
H	-2.406567	-6.305750	-0.766477
H	-1.551415	-7.340806	1.318244
H	-1.112655	-3.471388	3.075108
H	-0.914095	-5.899340	3.228044

2a’’

30
scf done: -692.068929

O	0.611208	0.970813	0.857892
C	-0.079247	-0.176496	1.353382
C	0.747977	-1.431063	1.011723
C	1.754725	-0.834777	0.078649
C	1.603890	0.484339	0.064153
C	-0.710672	0.066121	2.703513
O	-2.068907	0.336719	2.433169
C	-2.461263	-0.555681	1.399332
C	-1.243326	-0.692295	0.496161
C	-0.489674	-2.062768	0.329016
C	-1.140941	-3.265060	0.936358
C	-2.086873	-3.955393	0.180883
C	-2.767969	-5.040140	0.701440
C	-2.512744	-5.463876	1.997088
C	-1.572946	-4.791979	2.758232
C	-0.895432	-3.701667	2.232863
H	-0.271899	0.918053	3.224432
H	-0.610607	-0.827065	3.339141
H	-3.329317	-0.125175	0.899171
H	-2.757349	-1.523963	1.827730
H	-1.352976	-0.159396	-0.448044
H	2.166432	1.235087	-0.474266
H	2.508304	-1.383857	-0.465115
H	-2.287984	-3.628180	-0.835217
H	-3.498264	-5.561018	0.092760
H	-3.042041	-6.315413	2.408162
H	-0.166792	-3.190319	2.851922
H	-1.362723	-5.114887	3.771433
H	-0.303767	-2.269286	-0.728094
H	1.202389	-1.999696	1.828530

2a’

30
scf done: -692.054244

C	-2.909038	-0.189729	0.753210
C	-2.956567	-1.508214	0.875118
C	-1.848392	0.074965	-0.288844

C	-0.447156	-0.177584	0.298191
C	-0.521361	-1.646093	0.694594
C	-1.880256	-2.008200	-0.053659
C	0.659171	-2.499001	0.314383
H	-0.690604	-1.770340	1.769366
C	1.906418	-1.960892	0.019652
C	0.533036	-3.886565	0.322302
C	1.605975	-4.706352	0.023826
C	2.839909	-4.153869	-0.282297
C	2.986258	-2.778270	-0.277110
H	2.049405	-0.885693	0.029824
H	-0.422499	-4.335275	0.575500
H	1.478836	-5.782839	0.032837
H	3.682396	-4.793397	-0.517994
H	3.947987	-2.332596	-0.504238
C	-1.682672	1.491144	-0.750442
O	-0.935002	2.073354	0.323342
H	-2.615373	2.049591	-0.853308
H	-1.128652	1.540281	-1.696557
C	-0.267579	1.067452	1.112876
H	-0.745133	0.996084	2.098751
H	0.772382	1.372848	1.253288
H	0.212281	-0.101545	-0.573212
O	-1.948788	-1.063108	-1.130754
H	-1.953894	-3.025371	-0.431847
H	-3.433326	0.575649	1.308964
H	-3.522572	-2.115865	1.566497

[1][**BiNapht8**]

40
scf done: -848.844587

C	5.555244	-2.874650	1.833309
C	4.902491	-3.282202	0.649989
C	4.458810	-4.628346	0.528333
C	4.698747	-5.506334	1.610211
C	5.333800	-5.081449	2.742923
C	5.766148	-3.749164	2.859470
C	3.791835	-5.034696	-0.661240
C	3.617402	-4.120896	-1.668567
C	4.058043	-2.792583	-1.550440
C	4.682151	-2.378733	-0.410880
C	3.195371	-6.402822	-0.794366
C	1.871240	-6.548309	-0.020558
C	0.885665	-5.471459	-0.358714
C	0.161606	-5.486358	-1.583657
C	-0.671207	-4.380741	-1.911493
C	-0.772733	-3.296781	-1.014209
C	-0.086971	-3.310816	0.164363
C	0.739089	-4.400640	0.484976
C	-1.380469	-4.388582	-3.131962
C	-1.279519	-5.437030	-3.999612
C	-0.461967	-6.534265	-3.678535
C	0.237037	-6.556267	-2.504795

H	3.888254	-7.175796	-0.450152
H	-0.388690	-7.369045	-4.365884
H	0.855920	-7.415184	-2.274119
H	1.294712	-4.387884	1.418090
H	-0.168540	-2.480502	0.856123
H	-1.829298	-5.428802	-4.933474
H	-2.011840	-3.537165	-3.365548
H	2.076240	-6.505564	1.052369
H	1.459317	-7.542424	-0.216185
H	-1.408711	-2.458730	-1.279956
H	3.015476	-6.600527	-1.854339
H	3.105204	-4.430347	-2.574961
H	3.892539	-2.100086	-2.367547
H	5.022406	-1.354207	-0.301196
H	4.379730	-6.539401	1.538747
H	5.508364	-5.776084	3.556383
H	6.268210	-3.421947	3.762415
H	5.887843	-1.844215	1.908605

[3][**BiNapht8**]

40
scf done: -848.749195

C	5.515883	-2.864208	1.848460
C	4.864619	-3.273429	0.683610
C	4.464641	-4.650755	0.551484
C	4.752119	-5.519375	1.603484
C	5.424452	-5.075006	2.781500
C	5.798438	-3.777127	2.901829
C	3.784085	-5.054466	-0.626704
C	3.544243	-4.091524	-1.679793
C	3.922881	-2.804115	-1.552665
C	4.583594	-2.374447	-0.358576
C	3.202381	-6.418134	-0.772361
C	1.861727	-6.557968	-0.020414
C	0.895185	-5.468914	-0.375091
C	0.173223	-5.486590	-1.601229
C	-0.641168	-4.371462	-1.942979
C	-0.724874	-3.274905	-1.059077
C	-0.039071	-3.284794	0.119516
C	0.767933	-4.384444	0.454969
C	-1.348997	-4.382218	-3.164046
C	-1.262654	-5.441683	-4.020022
C	-0.461659	-6.547136	-3.686031
C	0.234826	-6.567195	-2.510633
H	3.887352	-7.188154	-0.404996
H	-0.399070	-7.389895	-4.364633
H	0.841192	-7.432350	-2.270029
H	1.323559	-4.369847	1.388109
H	-0.107023	-2.444372	0.800443
H	-1.811085	-5.435532	-4.954674
H	-1.966935	-3.523936	-3.408127
H	2.058504	-6.512741	1.054103
H	1.444605	-7.548861	-0.220073

H	-1.347144	-2.430035	-1.335441
H	3.042160	-6.624075	-1.834733
H	3.036214	-4.433959	-2.575771
H	3.733365	-2.089585	-2.344378
H	4.887629	-1.338790	-0.249919
H	4.473788	-6.563958	1.530624
H	5.628132	-5.786627	3.572638
H	6.307587	-3.425830	3.791259
H	5.804310	-1.822593	1.944283

[1][1a: BiNapht8]

70
scf done: -1540.943781

C	7.285303	-3.911896	-6.380991
C	7.884259	-2.979120	-5.612419
C	7.303125	-3.098940	-4.313591
C	6.396920	-4.098296	-4.401096
O	6.372386	-4.606325	-5.653803
C	5.472524	-4.717876	-3.426426
O	5.678532	-4.119842	-2.182028
H	5.656686	-5.805931	-3.371378
H	4.427076	-4.598450	-3.757231
C	4.833502	-4.652636	-1.181846
C	5.325860	-4.224010	0.147149
H	4.848684	-5.753842	-1.248180
H	3.796042	-4.325884	-1.348199
C	4.657743	-3.410622	0.958896
H	6.293226	-4.628598	0.436627
H	7.386659	-4.200952	-7.413936
H	8.648566	-2.286567	-5.926188
H	7.523474	-2.519911	-3.431706
C	5.075219	-2.973177	2.286435
H	3.689817	-3.035131	0.627614
C	4.206852	-2.194352	3.049940
C	6.310826	-3.315056	2.839476
C	6.666918	-2.878646	4.099796
C	5.789399	-2.108216	4.850387
C	4.554642	-1.770673	4.321375
H	3.241772	-1.922799	2.633533
H	3.860701	-1.170562	4.898587
H	6.068704	-1.773283	5.842561
H	7.004762	-3.936819	2.283203
H	7.633748	-3.156138	4.505911
C	8.781201	-3.744587	-0.151810
C	8.887790	-5.148068	-0.276808
C	9.486353	-5.901808	0.772226
C	9.928447	-5.198300	1.918775
C	9.811969	-3.840092	2.009207
C	9.236725	-3.099880	0.960457
C	9.625290	-7.309968	0.614734
C	9.155626	-7.898640	-0.532802
C	8.532131	-7.159738	-1.549154
C	8.405964	-5.806600	-1.426462
C	10.250205	-8.186285	1.660496
C	9.236522	-8.901448	2.563993
C	8.426080	-7.975987	3.423154
C	7.126318	-7.527141	3.054922
C	6.425351	-6.651088	3.931087
C	7.040021	-6.216929	5.122772
C	8.294067	-6.644514	5.449363
C	8.972873	-7.531970	4.601111
C	5.124180	-6.221512	3.588428
C	4.532805	-6.623269	2.427379
C	5.225086	-7.476607	1.550079
C	6.482236	-7.914119	1.855361
H	10.948143	-7.625253	2.286045
H	4.756476	-7.791738	0.624361
H	6.995047	-8.565951	1.159835
H	9.963248	-7.880889	4.880897
H	8.765007	-6.313657	6.367777
H	3.537241	-6.278727	2.172714
H	4.608819	-5.548935	4.268180
H	9.795195	-9.577924	3.217181
H	8.595383	-9.535702	1.947328
H	6.494028	-5.538959	5.771012
H	10.844034	-8.954392	1.156505
H	9.270321	-8.972722	-0.651899
H	8.168704	-7.665179	-2.436808
H	7.941683	-5.209706	-2.204220
H	10.369026	-5.743926	2.743666
H	10.164009	-3.328313	2.897702
H	9.143695	-2.023471	1.046865
H	8.311359	-3.194657	-0.961409

[3][1a: BiNapht8]

70
scf done: -1540.860315

C	11.728185	-6.960349	-0.197890
C	11.925268	-8.346713	-0.019076
C	12.216316	-8.851612	1.281278
C	12.277016	-7.921768	2.347078
C	12.074878	-6.586434	2.142057
C	11.803818	-6.095189	0.853422
C	12.431671	-10.250103	1.445241
C	12.325046	-11.069388	0.348612
C	12.015977	-10.573636	-0.926582
C	11.828787	-9.234415	-1.108905
C	12.798528	-10.868812	2.765149
C	11.699247	-11.739123	3.378563
C	10.441602	-10.997154	3.738494
C	9.189442	-11.244904	3.106284
C	8.034947	-10.544805	3.564472
C	8.155140	-9.601643	4.604203
C	9.368497	-9.359534	5.178220
C	10.499176	-10.068760	4.748239
C	6.782450	-10.803560	2.965705

C	6.657936	-11.706339	1.950808
C	7.794229	-12.389009	1.482642
C	9.019317	-12.163439	2.042872
H	13.103338	-10.110670	3.487177
H	7.697748	-13.097931	0.668530
H	9.876867	-12.697701	1.653812
H	11.450092	-9.891170	5.242838
H	9.462726	-8.634548	5.978161
H	5.690168	-11.895172	1.501198
H	5.916033	-10.262419	3.333392
H	12.103238	-12.191566	4.290165
H	11.490065	-12.570356	2.704842
H	7.265827	-9.075125	4.935400
H	13.677171	-11.504042	2.614593
H	12.501141	-12.134417	0.472274
H	11.939094	-11.257286	-1.764320
H	11.596633	-8.829242	-2.089529
H	12.477936	-8.271156	3.351704
H	12.123203	-5.900443	2.979674
H	11.643403	-5.033702	0.702300
H	11.504016	-6.600259	-1.197639
C	8.932955	-7.734491	-5.310539
C	9.171156	-6.563253	-4.684762
C	9.035643	-6.822423	-3.286896
C	8.724114	-8.133957	-3.179005
O	8.657531	-8.706336	-4.402187
C	8.467641	-9.016882	-2.020425
O	8.567159	-8.245817	-0.862951
H	9.202594	-9.840989	-2.006997
H	7.474162	-9.489682	-2.102574
C	8.567183	-9.036661	0.312686
C	8.647318	-8.159803	1.505026
H	9.415643	-9.736618	0.288723
H	7.642124	-9.638821	0.338758
C	7.557547	-7.278408	1.879651
H	9.537390	-8.216020	2.129472
H	8.919275	-8.035492	-6.344986
H	9.413728	-5.624654	-5.156596
H	9.144466	-6.131163	-2.466175
C	7.403735	-5.951300	1.439052
H	6.804889	-7.660415	2.573288
C	6.337206	-5.151028	1.910782
C	8.301706	-5.363411	0.517394
C	8.142186	-4.059286	0.108269
C	7.087653	-3.288818	0.590647
C	6.186971	-3.848676	1.493034
H	5.634885	-5.584302	2.616274
H	5.361779	-3.254654	1.869738
H	6.966039	-2.262736	0.264197
H	9.110919	-5.972023	0.127679
H	8.843528	-3.631216	-0.600587

^{3}[Ia:BiNapht8]

70

scf done: -1540.882788

C	0.100660	0.207764	-0.155386
C	0.016070	0.185140	1.257452
C	1.223754	0.356295	1.993249
C	2.442733	0.518085	1.299349
C	2.482760	0.538501	-0.063825
C	1.293195	0.386894	-0.796950
C	1.190009	0.365635	3.401837
C	0.009857	0.203648	4.065413
C	-1.179597	0.030486	3.342326
C	-1.207033	0.022318	1.969531
C	-2.537450	-0.119627	1.283844
C	-3.191780	-1.487663	1.474013
C	-2.433056	-2.646265	0.884920
C	-2.041382	-3.778840	1.654902
C	-1.452297	-4.893751	0.990786
C	-1.197039	-4.830002	-0.391892
C	-1.528955	-3.714466	-1.102160
C	-2.164681	-2.641453	-0.461476
C	-2.197281	-3.861947	3.058791
C	-1.827379	-4.978338	3.755555
C	-1.292772	-6.091644	3.085575
C	-1.107794	-6.041801	1.734799
O	2.024205	-4.069569	-1.379170
C	2.308512	-3.037797	-0.467315
C	2.392762	-3.663935	0.929688
O	1.156622	-3.455521	1.643966
C	1.441179	-2.889753	2.836166
C	2.791577	-2.655250	2.982750
C	3.418929	-3.080414	1.835274
C	2.749931	-5.178760	-0.911084
C	2.530425	-5.199033	0.592839
C	3.565072	-5.938436	1.343821
C	3.417085	-6.437973	2.649450
C	4.483045	-7.125487	3.274821
C	4.364504	-7.613818	4.555665
C	3.182477	-7.439044	5.270274
C	2.119569	-6.766394	4.676712
C	2.226844	-6.274729	3.394966
H	3.277860	-2.572092	-0.706210
H	1.526737	-2.276434	-0.516315
H	3.824881	-5.061012	-1.133408
H	2.388589	-6.076127	-1.415947
H	1.532523	-5.609062	0.791493
H	3.248251	-2.197487	3.847298
H	4.472106	-3.050594	1.596527
H	4.532934	-6.072351	0.863678
H	5.407108	-7.262375	2.721718
H	5.197925	-8.137124	5.010723
H	3.092438	-7.823011	6.279473
H	1.387037	-5.748692	2.955291
H	1.194667	-6.621644	5.225650
H	0.598354	-2.687919	3.480979
H	-2.462403	0.105242	0.220095

H	-1.954131	-5.008050	4.831717
H	-2.609640	-3.022419	3.604531
H	-2.473763	-1.786917	-1.056280
H	-1.317100	-3.657244	-2.163556
H	-1.014886	-6.978757	3.643487
H	-0.674916	-6.886795	1.206750
H	-4.181654	-1.453549	1.005407
H	-3.379272	-1.639622	2.536709
H	-0.717721	-5.676096	-0.874585
H	-3.218808	0.632476	1.694519
H	-2.114687	-0.076304	3.885250
H	-0.017644	0.213752	5.148836
H	2.121323	0.500421	3.942508
H	-0.795130	0.078751	-0.748640
H	1.323798	0.400780	-1.880362
H	3.425425	0.667493	-0.582859
H	3.352406	0.628973	1.881329

^{1}[**2a:BiNapth8**]

70
scf done: -1540.942122

O	-0.451230	-0.361770	0.458880
O	-3.114901	-2.673023	0.830031
C	0.764386	-3.705246	2.773371
C	-0.511549	-3.075409	2.320202
H	-1.352107	-3.607419	2.779849
C	-1.188886	-1.402674	1.089529
C	-0.792379	-2.872309	0.811238
H	0.062415	-3.018760	0.150223
C	1.943746	-3.613147	2.040688
H	1.953651	-3.097521	1.086874
C	-0.696190	-1.541567	2.540931
H	-1.382274	-1.275485	3.351792
C	-2.074398	-3.481769	0.297088
H	-2.098981	-3.463510	-0.803968
H	-2.242470	-4.507730	0.628647
C	0.796726	-4.392670	3.983037
H	-0.116769	-4.481897	4.563619
C	-2.660455	-1.340767	0.760721
H	-3.246471	-0.734205	1.453871
H	-2.774134	-0.930403	-0.255109
C	3.132326	-4.875510	3.704213
H	4.047376	-5.333821	4.060988
C	3.115701	-4.191327	2.499305
H	4.018140	-4.111548	1.899703
C	0.524057	-0.678631	2.489716
H	1.235392	-0.543160	3.290133
C	1.965683	-4.973770	4.446503
H	1.963772	-5.511019	5.388161
C	0.563243	-0.058211	1.317665
H	1.270878	0.670029	0.944278
C	5.282577	-2.280341	0.099416
C	4.130345	-2.519355	-0.679477
C	4.047268	-3.705505	-1.460438
C	5.154393	-4.584507	-1.443724
C	6.257538	-4.326006	-0.678925
C	6.322649	-3.164395	0.108950
C	2.872180	-3.952357	-2.227513
C	1.851719	-3.036666	-2.184037
C	1.941623	-1.854560	-1.429220
C	3.060426	-1.600229	-0.693580
C	2.733525	-5.164216	-3.100730
C	2.554042	-6.504510	-2.372734
C	1.476000	-6.534788	-1.332063
C	0.096215	-6.600228	-1.678624
C	-0.876647	-6.666587	-0.642765
C	-0.459879	-6.645344	0.706029
C	0.864376	-6.568794	1.016630
C	1.824592	-6.516910	-0.007010
C	-2.242634	-6.758701	-0.984893
C	-2.645581	-6.768031	-2.288872
C	-1.689170	-6.699795	-3.316050
C	-0.357562	-6.624762	-3.017259
H	3.607872	-5.254357	-3.754273
H	-2.009820	-6.715711	-4.351185
H	0.363478	-6.591635	-3.825168
H	2.876463	-6.465300	0.259611
H	1.186465	-6.545668	2.051461
H	-3.698813	-6.834371	-2.535038
H	-2.971622	-6.818428	-0.182380
H	3.491623	-6.782715	-1.886455
H	2.370260	-7.272489	-3.130921
H	-1.212703	-6.693785	1.487664
H	1.879883	-4.998894	-3.762782
H	0.952235	-3.225748	-2.763685
H	1.111263	-1.157358	-1.421444
H	3.142973	-0.698659	-0.095084
H	5.135173	-5.479412	-2.053134
H	7.090708	-5.019053	-0.683990
H	7.200002	-2.970901	0.714776
H	5.320773	-1.373809	0.695182

Ts(^{3}[1a]-^{3}[Ia])

30
scf done: -691.995573

O	-0.130984	0.236437	-0.023278
C	0.064651	0.475238	1.319613
C	1.407414	0.257730	1.606884
C	2.058387	0.052582	0.374248
C	1.087187	0.058823	-0.577374
C	-1.121844	0.281193	2.195891
O	-2.098778	1.268365	1.990518
C	-1.496586	2.534271	2.162903
C	-0.259612	2.643569	1.329985
C	0.905487	3.358307	1.834220
C	0.976143	4.752665	2.017496

C	2.150136	5.343211	2.543391
C	2.240143	6.702019	2.736068
C	1.170066	7.532528	2.412056
C	0.007137	6.977436	1.887619
C	-0.095108	5.617895	1.692103
H	-0.767383	0.289191	3.238955
H	-1.595643	-0.684974	2.002726
H	-1.243753	2.695068	3.224133
H	-2.256187	3.273364	1.883507
H	-0.436165	2.685736	0.252580
H	3.113688	-0.090423	0.201227
H	1.849238	0.311121	2.590538
H	1.798784	2.785019	2.086897
H	2.985421	4.697140	2.796050
H	3.150633	7.128768	3.142612
H	1.243437	8.603003	2.565314
H	-1.004874	5.199629	1.273572
H	-0.827192	7.620176	1.628379
H	1.114246	-0.065220	-1.649075

Ts(^{3}[1a:BiNapht8]-^{3}[Ia:BiNapht8])

70
scf done: -1540.878075

C	0.014887	0.093491	0.132924
C	-0.110465	0.083032	1.542617
C	1.084694	-0.006117	2.312689
C	2.332306	-0.062866	1.653469
C	2.412862	-0.048140	0.291705
C	1.236869	0.028141	-0.475196
C	1.009242	-0.041171	3.720134
C	-0.201052	0.016073	4.347651
C	-1.377096	0.127671	3.590562
C	-1.362337	0.163799	2.218240
C	-2.672480	0.286027	1.494572
C	-3.460547	-1.025224	1.398587
C	-2.787203	-2.086874	0.576392
C	-2.161842	-3.231719	1.148376
C	-1.563847	-4.193870	0.283496
C	-1.555406	-3.975088	-1.108866
C	-2.149982	-2.865327	-1.635652
C	-2.776186	-1.939416	-0.788606
C	-2.112965	-3.480470	2.541277
C	-1.547658	-4.619666	3.041498
C	-0.993051	-5.580345	2.178701
C	-0.994175	-5.362279	0.832948
O	1.848922	-4.739277	-1.022084
C	2.332839	-3.552524	-0.438691
C	2.542397	-3.625037	1.029201
O	1.446523	-3.490833	1.835902
C	1.899695	-3.302551	3.093909
C	3.258680	-3.218596	3.114301
C	3.681629	-3.383960	1.783395
C	2.637025	-5.839861	-0.609547
C	2.618782	-5.973379	0.860546
C	3.795357	-6.349370	1.614393
C	3.831495	-6.599820	2.979355
C	5.071601	-6.878377	3.628190
C	5.142264	-7.078489	4.978963
C	3.984655	-7.024482	5.770137
C	2.758766	-6.765922	5.165773
C	2.668239	-6.555222	3.807958
H	3.297067	-3.271853	-0.891460
H	1.602595	-2.770644	-0.670453
H	3.674102	-5.727912	-0.969858
H	2.204061	-6.711215	-1.118892
H	1.639935	-6.102681	1.312166
H	3.871354	-3.068544	3.990095
H	4.690066	-3.372147	1.397290
H	4.736193	-6.403597	1.068556
H	5.970313	-6.917440	3.019678
H	6.101145	-7.281720	5.443612
H	4.045716	-7.187157	6.839804
H	1.699807	-6.358803	3.362260
H	1.858935	-6.727388	5.770915
H	1.149443	-3.227067	3.866266
H	-2.535767	0.695212	0.491580
H	-1.530243	-4.786223	4.113229
H	-2.537289	-2.759675	3.229225
H	-3.266213	-1.072051	-1.223214
H	-2.153390	-2.700068	-2.707243
H	-0.554266	-6.484844	2.586738
H	-0.549765	-6.082695	0.152807
H	-4.427080	-0.795373	0.939056
H	-3.681406	-1.378938	2.407260
H	-1.073114	-4.709062	-1.745673
H	-3.295726	1.005750	2.033979
H	-2.332862	0.195429	4.103509
H	-0.260281	-0.009486	5.429966
H	1.931675	-0.115117	4.287817
H	-0.874262	0.149200	-0.482008
H	1.299713	0.033680	-1.557802
H	3.378093	-0.101301	-0.200217
H	3.230366	-0.126961	2.259965

1o

30
scf done: -1015.053233

S	-2.460588	1.123392	-2.222862

C	-3.491830	1.896477	-1.097707
C	-3.829964	1.059796	-0.078171
C	-3.251659	-0.222860	-0.213331
C	-2.482080	-0.349286	-1.331532
C	-1.688035	-1.536041	-1.768530
O	-0.661240	-1.888361	-0.870361
H	-1.269891	-1.358444	-2.771012
H	-2.334951	-2.414465	-1.834902
C	0.357183	-0.915383	-0.780293
C	1.415241	-1.410319	0.130787
H	-0.062934	0.028462	-0.396449
H	0.771208	-0.700618	-1.778540
C	2.700768	-1.474369	-0.201190
H	1.075542	-1.715211	1.118166
C	3.812677	-1.909670	0.639268
H	2.980030	-1.176284	-1.211376
C	3.665451	-2.242013	1.987402
C	4.748459	-2.659684	2.735453
C	6.007463	-2.752456	2.158056
C	6.171496	-2.421198	0.824098
C	5.084622	-2.003467	0.076009
H	2.691619	-2.168265	2.458669
H	4.612576	-2.912702	3.780725
H	6.855331	-3.078692	2.748817
H	7.150119	-2.487272	0.362851
H	5.215255	-1.744378	-0.970017
H	-3.798722	2.919382	-1.254708
H	-4.477508	1.350177	0.738857
H	-3.391088	-1.033228	0.492351

2o

30
scf done: -1015.049891

S	-1.012676	0.237033	6.908739
O	1.054624	2.573436	4.477304
C	-0.451551	4.407160	8.249408
C	0.233555	3.632524	7.172213
H	1.031788	4.247847	6.745667
C	0.024810	1.616889	6.328927
C	-0.613834	2.998432	6.044919
H	-1.694025	3.069744	6.171620
C	-1.713173	4.059171	8.726306
H	-2.240672	3.214371	8.294937
C	0.795175	2.213552	7.527616
H	1.886695	2.160938	7.443609
C	-0.146820	3.312076	4.640771
H	-0.894205	2.984432	3.903478
H	0.075142	4.367679	4.471404
C	0.193925	5.492744	8.832775
H	1.175970	5.779078	8.468836
C	0.811774	1.315713	5.069833
H	1.767598	0.824641	5.264084
H	0.218361	0.677476	4.396011
C	-1.651285	5.853986	10.322816
H	-2.118757	6.415394	11.123356
C	-2.307312	4.775247	9.751551
H	-3.290856	4.488513	10.106090
C	0.302314	1.552660	8.763895
H	0.650584	1.847195	9.747211
C	-0.396106	6.211224	9.859239
H	0.125067	7.056090	10.294872
C	-0.573309	0.572869	8.578636
H	-1.028160	-0.031195	9.352972

2o’

30
scf done: -1015.038263

C	-2.939231	-0.160616	0.326658
C	-3.026875	-1.478227	0.220802
C	-1.684346	0.325926	-0.358890
C	-0.462472	-0.211037	0.427114
C	-0.628520	-1.729694	0.507145
C	-1.808509	-1.994622	-0.500050
C	0.608400	-2.542124	0.243494
H	-0.998333	-2.015162	1.498218
C	1.391765	-2.368192	-0.894932
C	0.978708	-3.529061	1.150604
C	2.096309	-4.318760	0.935981
C	2.865521	-4.135947	-0.200324
C	2.506974	-3.158360	-1.114854
H	1.120985	-1.610266	-1.622200
H	0.378887	-3.676587	2.043576
H	2.366388	-5.078175	1.660893
H	3.741490	-4.750453	-0.372578
H	3.102671	-3.006965	-2.007956
C	-1.415649	1.798782	-0.279311
O	-0.933463	1.974830	1.055011
H	-2.304376	2.420733	-0.408414
H	-0.657336	2.107736	-1.010528
C	-0.479544	0.730620	1.604478
H	-1.175999	0.393677	2.384167
H	0.500394	0.886187	2.061657
H	0.422741	0.019766	-0.173452
S	-1.597702	-0.737250	-1.846549
H	-1.854080	-3.011785	-0.883287
H	-3.589035	0.502626	0.884702

H	-3.760551	-2.127032	0.681086

z

44

scf done: -1828.194157

N	-0.187732	0.575286	-1.618403
C	-0.208177	1.938563	-1.345465
C	-1.234852	2.179905	-0.493001
C	-1.890835	0.944814	-0.246907
C	-1.251909	-0.032763	-0.946201
C	-1.542099	-1.494487	-0.984692
N	-1.969434	-1.967105	0.334443
H	-0.661621	-2.041959	-1.331110
H	-2.356489	-1.706277	-1.681244
C	-1.032299	-1.771356	1.459193
C	0.386318	-1.984408	1.062637
H	-1.339031	-2.450177	2.257220
H	-1.158362	-0.754791	1.842573
C	1.268738	-0.991032	1.017304
H	0.665735	-2.986420	0.740461
C	2.648440	-1.054718	0.552077
H	0.923141	0.003999	1.301143
C	3.318429	-2.257002	0.322141
C	4.599675	-2.262337	-0.191886
C	5.242710	-1.067212	-0.485332
C	4.595971	0.133591	-0.248939
C	3.313478	0.137069	0.271020
H	2.830198	-3.198320	0.549427
H	5.104970	-3.205372	-0.365862
H	6.247082	-1.075458	-0.892118
H	5.090270	1.071966	-0.471760
H	2.802413	1.078453	0.449230
H	0.527324	2.588026	-1.789111
H	-1.490500	3.141325	-0.076411
H	-2.735950	0.775103	0.403512
S	-3.000154	-3.237033	0.390543
S	0.776856	-0.051514	-2.849321
O	-3.935453	-3.081042	-0.682906
O	-3.477863	-3.346040	1.735481
C	-2.019237	-4.663205	0.049419
H	-1.539262	-4.538525	-0.921214
H	-2.678631	-5.530004	0.032182
H	-1.275617	-4.776649	0.837662
O	1.124723	-1.391566	-2.509192
O	1.795086	0.919105	-3.076973
C	-0.330271	-0.069096	-4.211978
H	-1.162185	-0.734382	-3.985550
H	0.226259	-0.440241	-5.071927
H	-0.679072	0.946914	-4.389054

z’

44

scf done: -1828.173934

C	-2.741703	1.047817	2.505847
C	-2.966576	-0.201547	2.884657
C	-2.293485	0.988598	1.060700
C	-0.873981	0.389474	1.015339
C	-1.041821	-0.989674	1.663905
C	-2.617655	-1.070961	1.701452
C	-0.272525	-2.066823	0.948900
H	-0.690119	-0.970097	2.700883
C	1.118946	-2.009088	1.008650
C	-0.860005	-3.100189	0.234108
C	-0.076182	-4.049574	-0.407141
C	1.303161	-3.980797	-0.342606
C	1.901455	-2.952490	0.371034
H	1.590925	-1.208630	1.572585
H	-1.938076	-3.168500	0.153684
H	-0.554777	-4.847686	-0.962948
H	1.912148	-4.723585	-0.844326
H	2.981773	-2.888111	0.432387
C	-2.114041	2.309415	0.361175
N	-0.766699	2.690718	0.858080
H	-2.849049	3.064045	0.642135
H	-2.122920	2.193246	-0.728164
C	-0.043590	1.550660	1.490672
H	-0.050714	1.661869	2.579233
H	0.996845	1.525113	1.163699
H	-0.651126	0.233492	-0.047027
N	-3.095249	-0.165722	0.613357
H	-3.060766	-2.061600	1.656451
H	-2.777770	1.958776	3.087745
H	-3.221858	-0.581085	3.863452
S	0.078251	3.778928	-0.015149
S	-3.319927	-0.613474	-0.946104
O	-3.741191	-1.982420	-0.915870
O	-2.217113	-0.241111	-1.789067
O	1.235904	4.133392	0.747535
O	-0.850378	4.786124	-0.430851
C	0.623012	2.915043	-1.456277
C	-4.687390	0.395347	-1.398096
H	-4.893845	0.198379	-2.449497
H	-4.424335	1.443660	-1.263470
H	-5.539299	0.129756	-0.776825
H	-0.240468	2.534646	-2.001686
H	1.168777	3.624736	-2.076585
H	1.281497	2.098876	-1.161115

z''

44

scf done: -1828.189715

N	0.621267	0.433621	7.445307
N	1.141286	2.716988	4.669555
C	-0.657536	4.328553	8.463336
C	0.214072	3.714451	7.417905
H	0.691679	4.517745	6.847348
C	0.834637	1.714299	6.757045
C	-0.363045	2.655663	6.448064
H	-1.348137	2.287244	6.729068
C	-1.690336	3.629121	9.081221
H	-1.910842	2.608660	8.786550
C	1.300695	2.688537	7.861701
H	2.325825	3.061262	7.766707
C	-0.264217	2.983711	4.975051
H	-0.913618	2.333354	4.379403
H	-0.519022	4.020750	4.748495
C	-0.410655	5.634510	8.875323
H	0.387915	6.195095	8.399063
C	1.675097	1.637978	5.492959
H	2.737548	1.789301	5.696556
H	1.551718	0.668812	5.003926
C	-2.191366	5.517970	10.478242
H	-2.788612	5.978569	11.256332
C	-2.450711	4.217512	10.077764
H	-3.252213	3.655478	10.543123
C	1.096394	1.911503	9.118366
H	1.282637	2.290438	10.112281
C	-1.166812	6.226307	9.872428
H	-0.958126	7.246514	10.173264
C	0.740403	0.665768	8.837680
H	0.570049	-0.161008	9.513643
S	-0.369923	-0.761873	6.896761
S	2.030860	3.572915	3.625326
O	1.134706	4.155185	2.672019
O	3.110790	2.737856	3.192477
O	0.035819	-1.964131	7.554515
O	-0.365493	-0.673292	5.469180
C	2.730830	4.889690	4.567717
H	1.925849	5.513851	4.954635
H	3.371147	5.473304	3.907311
H	3.319877	4.466278	5.381398
C	-1.996446	-0.387410	7.472771
H	-1.972311	-0.235599	8.551544
H	-2.616848	-1.251106	7.236075
H	-2.377411	0.492797	6.959053

Crystallographic data

Table S7. Crystal data and structure refinement for 2a.

Empirical formula	$C_{14}H_{14}O_2$
Formula weight	214.25
Temperature/K	200.00
Crystal system	monoclinic
Space group	$P2_1/c$
a/Å	8.9307(5)
b/Å	5.7413(3)
c/Å	21.6646(15)
α/°	90
β/°	101.257(2)
γ/°	90
Volume/Å^3	1089.46(11)
Z	4
ρ_{calc}g/cm^3	1.306
μ/mm^{-1}	0.086
F(000)	456.0
Crystal size/mm^3	0.2 × 0.03 × 0.03
Radiation	MoKα (λ = 0.71073 Å)
2Θ range for data collection/°	3.834 to 51.62
Index ranges	$-10 \leq h \leq 10$, $-7 \leq k \leq 7$, $-26 \leq l \leq 26$
Reflections collected	23586
Independent reflections	2067 [R_{int} = 0.0488, R_{sigma} = 0.0271]
Data/restraints/parameters	2067/0/145
Goodness-of-fit on F^2	1.032
Final R indexes [I>=2σ (I)]	R_1 = 0.0403, wR_2 = 0.0961
Final R indexes [all data]	R_1 = 0.0547, wR_2 = 0.1049
Largest diff. peak/hole / e Å^{-3}	0.24/-0.17

Crystal structure for 2a

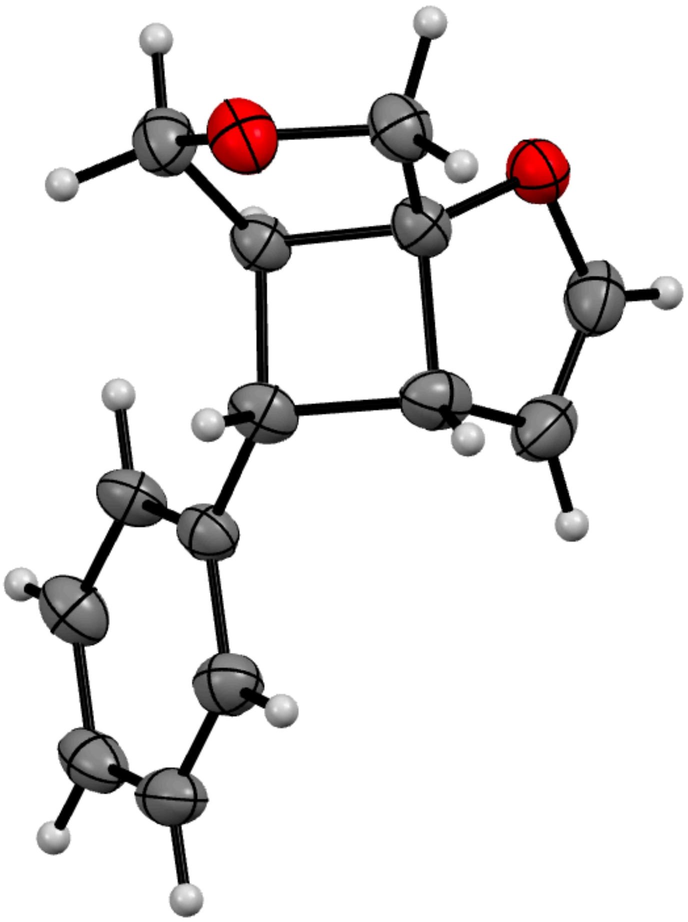

Thermal ellipsoid is drawn at 50% probability level

Table S8. Crystal data and structure refinement for **2m**.

Identification code	cu_LOT110_a
Empirical formula	$C_{24}H_{24}O_3$
Formula weight	360.43
Temperature/K	200.00
Crystal system	monoclinic
Space group	$P2_1/c$
a/Å	10.7762(3)
b/Å	7.9135(2)
c/Å	21.7832(5)
α/°	90
β/°	96.9080(10)
γ/°	90
Volume/Å^3	1844.13(8)
Z	4
ρ_{calc}g/cm^3	1.298
μ/mm^{-1}	0.669
F(000)	768.0
Crystal size/mm^3	0.4 × 0.12 × 0.04
Radiation	CuKα (λ = 1.54178)
2Θ range for data collection/°	8.176 to 140.14
Index ranges	$-13 \le h \le 13$, $-9 \le k \le 8$, $-26 \le l \le 26$
Reflections collected	19788
Independent reflections	3508 [R_{int} = 0.0662, R_{sigma} = 0.0488]
Data/restraints/parameters	3508/0/244
Goodness-of-fit on F^2	1.022
Final R indexes [I>=2σ (I)]	R_1 = 0.0490, wR_2 = 0.1233
Final R indexes [all data]	R_1 = 0.0589, wR_2 = 0.1335
Largest diff. peak/hole / e Å^{-3}	0.25/-0.26

Crystal structure for 2m

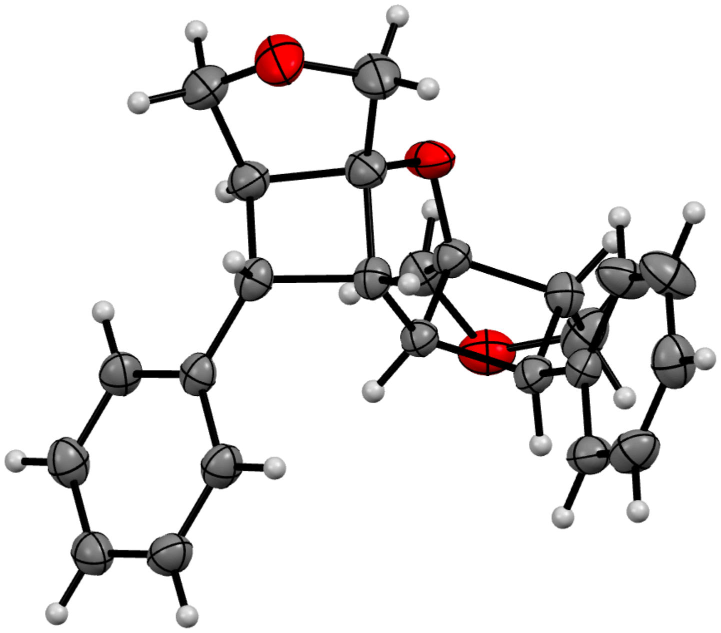

Thermal ellipsoid is drawn at 50% probability level

Table S9. Crystal data and structure refinement for 2p.

Empirical formula	$C_{28}H_{28}N_2O_4S_2$
Formula weight	520.64
Temperature/K	200.00
Crystal system	orthorhombic
Space group	Pbca
a/Å	16.110(2)
b/Å	16.1051(16)
c/Å	19.765(2)
α/°	90
β/°	90
γ/°	90
Volume/Å^3	5128.2(10)
Z	8
ρ_{calc}g/cm^3	1.349
μ/mm^{-1}	2.190
F(000)	2192.0
Crystal size/mm^3	0.4 × 0.2 × 0.06
Radiation	CuKα (λ = 1.54178 Å)
2Θ range for data collection/°	8.96 to 149.562
Index ranges	$-17 \leq h \leq 20$, $-20 \leq k \leq 20$, $-24 \leq l \leq 24$
Reflections collected	59336
Independent reflections	5259 [R_{int} = 0.0599, R_{sigma} = 0.0282]
Data/restraints/parameters	5259/0/327
Goodness-of-fit on F^2	1.020
Final R indexes [I>=2σ (I)]	R_1 = 0.0382, wR_2 = 0.1035
Final R indexes [all data]	R_1 = 0.0421, wR_2 = 0.1067
Largest diff. peak/hole / e Å^{-3}	0.27/-0.43

Crystal structure for 2p

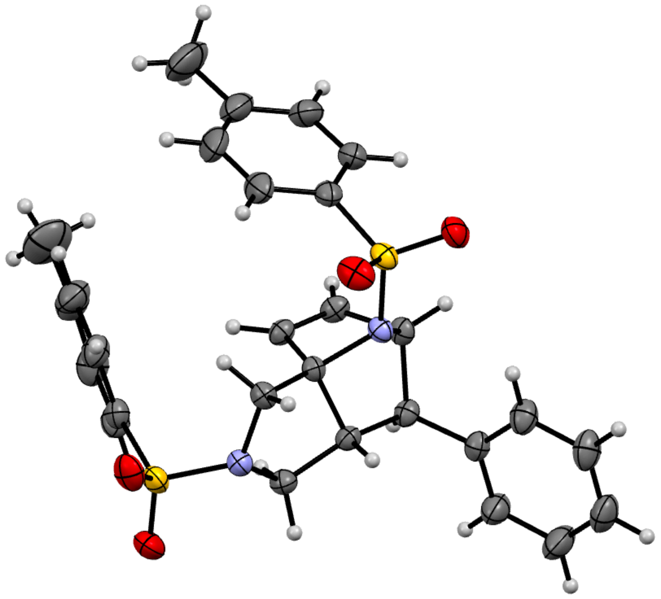

Thermal ellipsoid is drawn at 50% probability level

Table S10. Crystal data and structure refinement for 4c.

Empirical formula	$C_{18}H_{16}OS$
Formula weight	280.37
Temperature/K	200.00(10)
Crystal system	orthorhombic
Space group	$Pca2_1$
a/Å	16.5042(3)
b/Å	22.5178(4)
c/Å	7.73740(10)
α/°	90
β/°	90
γ/°	90
Volume/Å^3	2875.51(8)
Z	8
ρ_{calc}g/cm^3	1.295
μ/mm^{-1}	1.920
F(000)	1184.0
Crystal size/mm^3	0.2 × 0.07 × 0.05
Radiation	Cu Kα (λ = 1.54184 Å)
2Θ range for data collection/°	3.924 to 148.992
Index ranges	$-20 \leq h \leq 19$, $-28 \leq k \leq 28$, $-9 \leq l \leq 9$
Reflections collected	43125
Independent reflections	5815 [R_{int} = 0.0761, R_{sigma} = 0.0366]
Data/restraints/parameters	5815/1/489
Goodness-of-fit on F^2	1.038
Final R indexes [I>=2σ (I)]	R_1 = 0.0413, wR_2 = 0.0974
Final R indexes [all data]	R_1 = 0.0503, wR_2 = 0.1051
Largest diff. peak/hole / e Å^{-3}	0.18/-0.20
Flack parameter	0.011(9)

Crystal structure for 4c

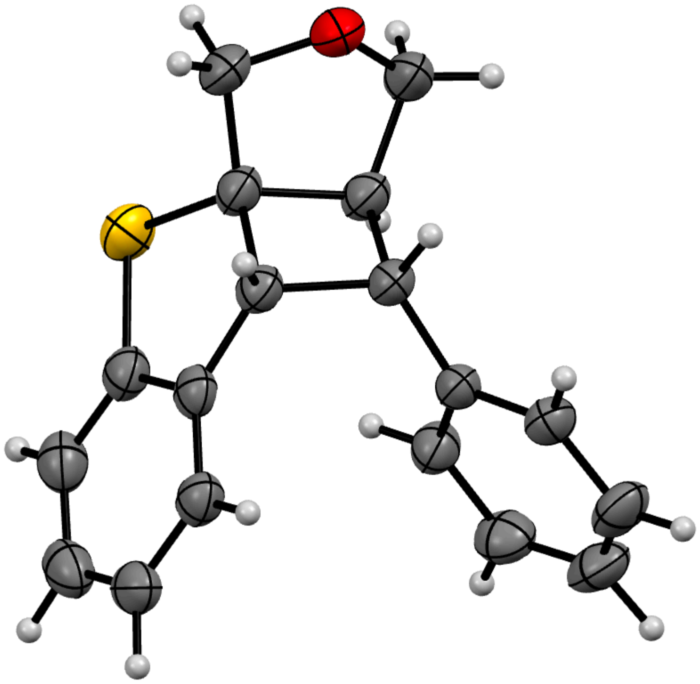

Thermal ellipsoid is drawn at 50% probability level

Table S11. Crystal data and structure refinement for 4c'.

Empirical formula	$C_{18}H_{16}OS$
Formula weight	280.37
Temperature/K	295.00(10)
Crystal system	monoclinic
Space group	Cc
a/Å	22.4167(19)
b/Å	9.7774(5)
c/Å	15.4898(12)
α/°	90
β/°	121.833(12)
γ/°	90
Volume/Å^3	2884.4(5)
Z	8
ρ_{calc}g/cm^3	1.291
μ/mm^{-1}	1.915
F(000)	1184.0
Crystal size/mm^3	0.4 × 0.2 × 0.05
Radiation	Cu Kα (λ = 1.54184 Å)
2Θ range for data collection/°	9.288 to 157.094
Index ranges	$-27 \leq h \leq 28$, $-12 \leq k \leq 12$, $-17 \leq l \leq 19$
Reflections collected	38472
Independent reflections	5797 [R_{int} = 0.0641, R_{sigma} = 0.0333]
Data/restraints/parameters	5797/2/362
Goodness-of-fit on F^2	1.107
Final R indexes [I>=2σ (I)]	R_1 = 0.0389, wR_2 = 0.0954
Final R indexes [all data]	R_1 = 0.0532, wR_2 = 0.1143
Largest diff. peak/hole / e Å^{-3}	0.18/-0.18
Flack parameter	-0.008(10)

Crystal structure for 4c'

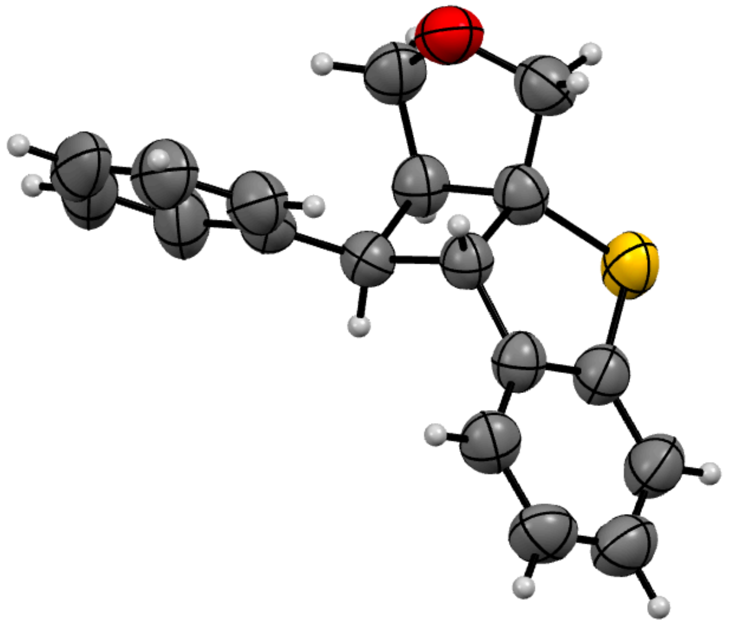

Thermal ellipsoid is drawn at 50% probability level

Copy of NMR spectra

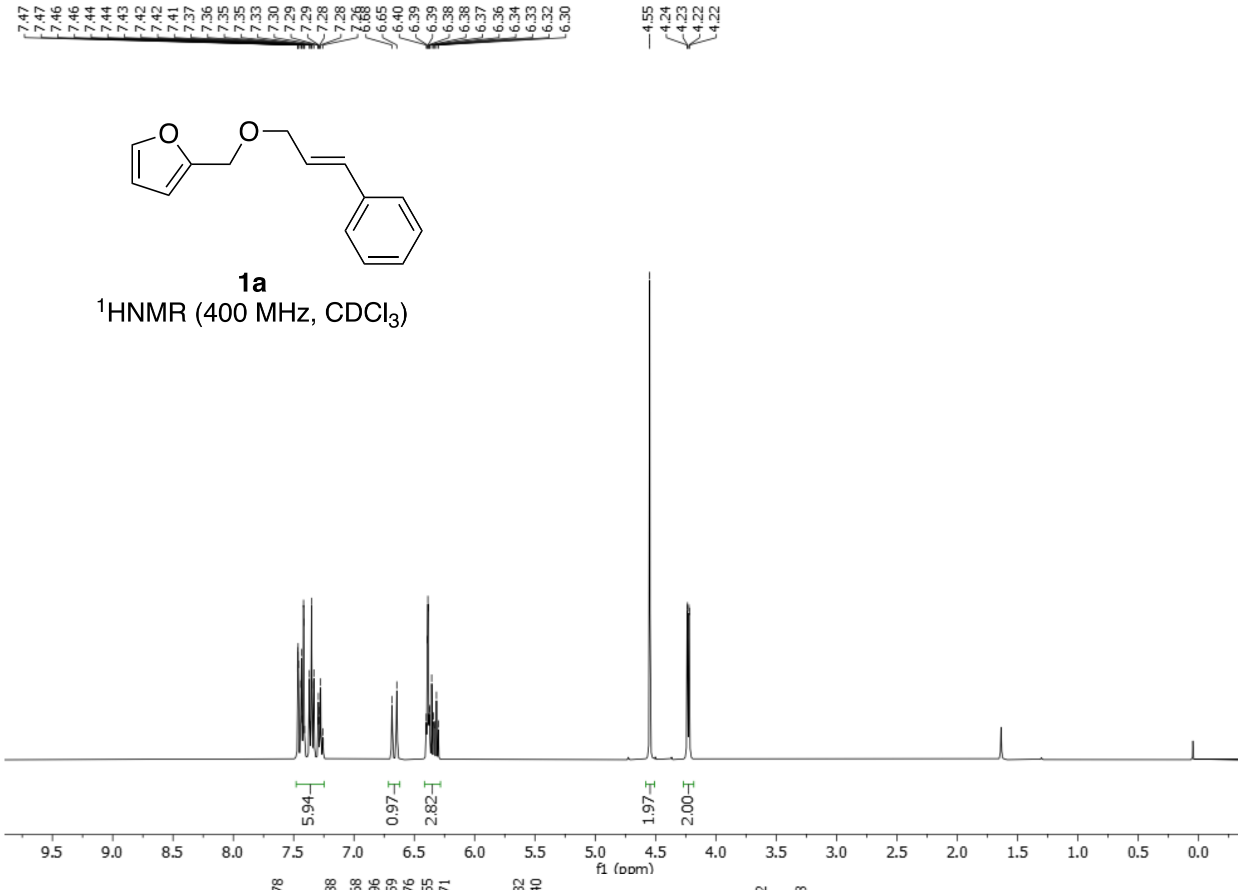

1a
^{1}HNMR (400 MHz, $CDCl_3$)

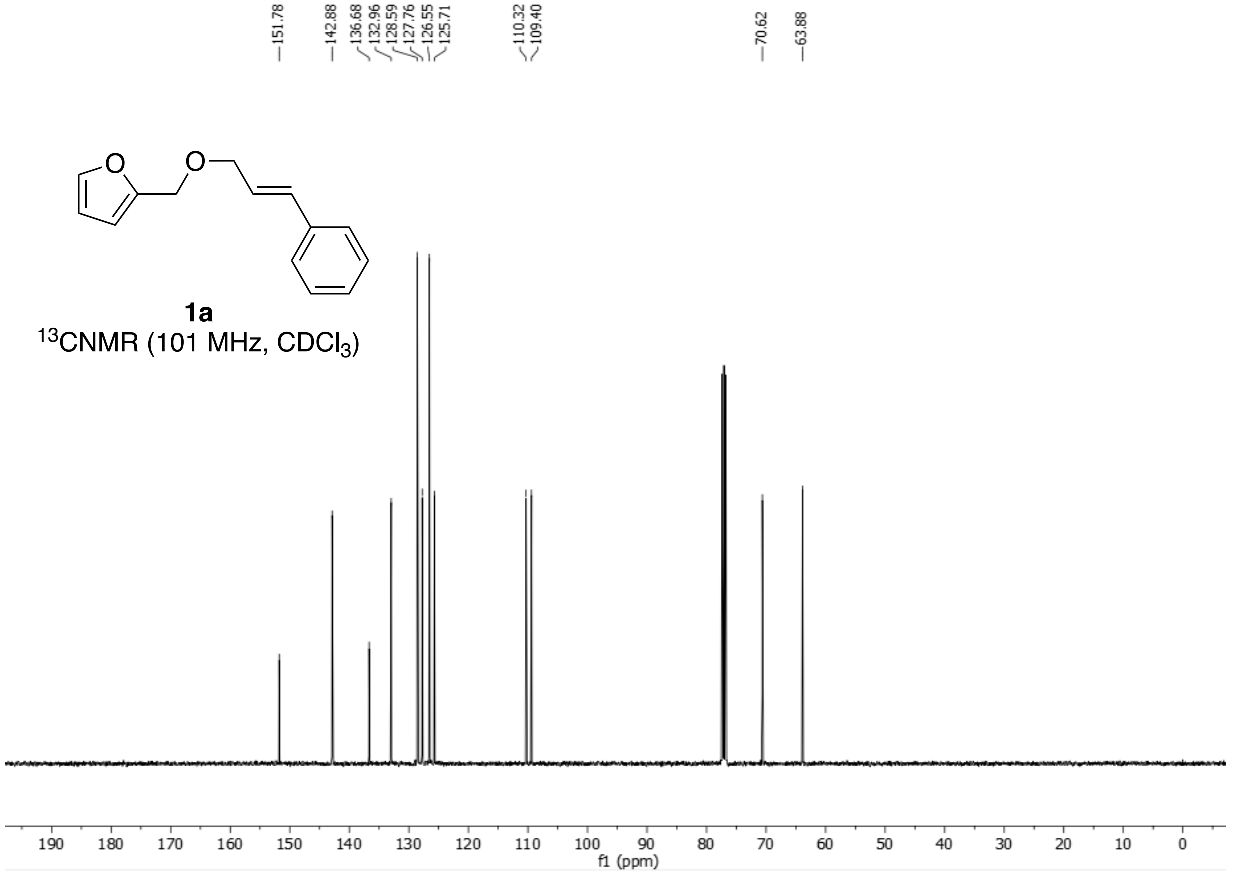

1a
^{13}CNMR (101 MHz, $CDCl_3$)

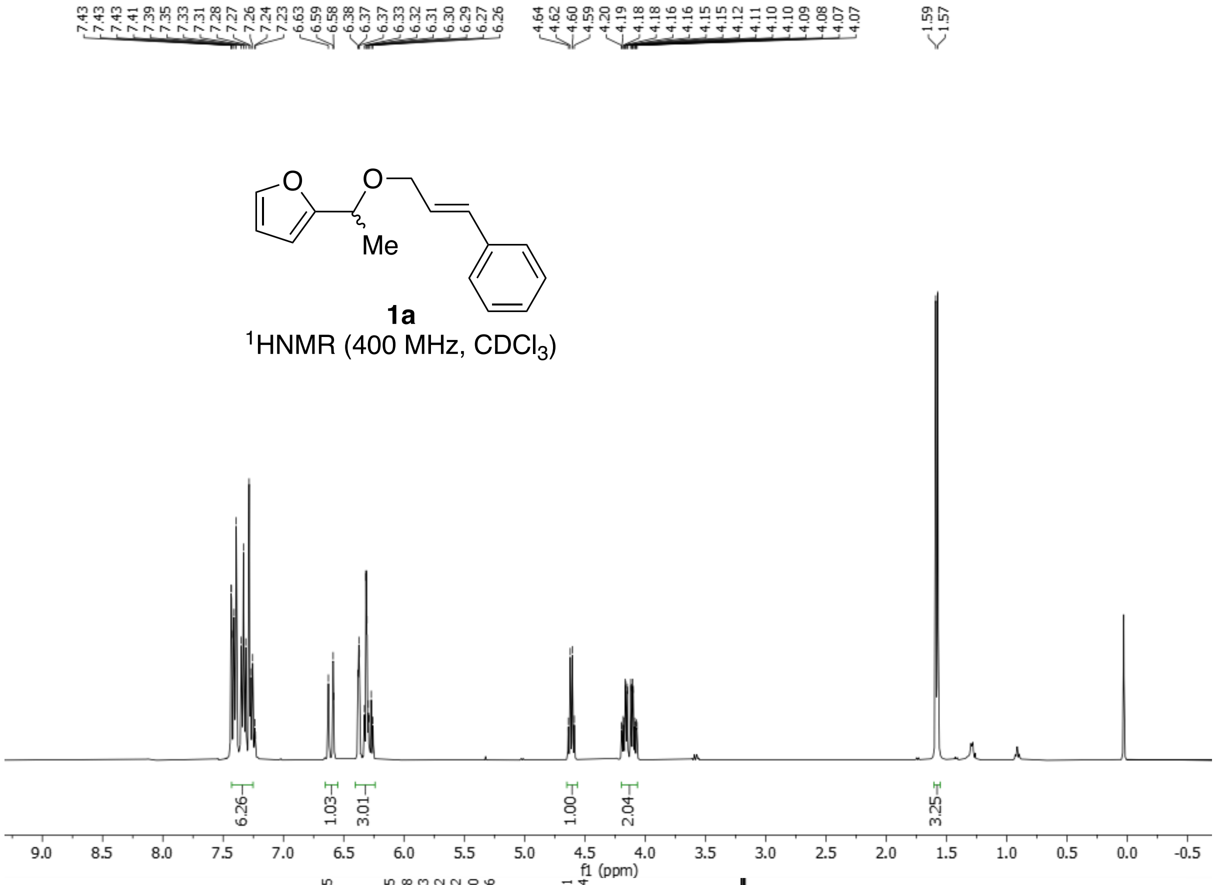
7.43 7.43 7.41 7.39 7.35 7.33 7.31 7.28 7.27 7.26 7.24 7.23 6.63 6.59 6.58 6.38 6.37 6.37 6.33 6.32 6.31 6.30 6.29 6.27 6.26
4.64 4.62 4.60 4.59 4.20 4.19 4.18 4.16 4.16 4.15 4.15 4.12 4.11 4.10 4.09 4.08 4.07 4.07
1.59 1.57
O
O
Me
1a
^{1}HNMR (400 MHz, $CDCl_3$)
6.26
1.03
3.01
1.00
2.04
3.25
9.0 8.5 8.0 7.5 7.0 6.5 6.0 5.5 5.0 4.5 4.0 3.5 3.0 2.5 2.0 1.5 1.0 0.5 0.0 -0.5
f1 (ppm)

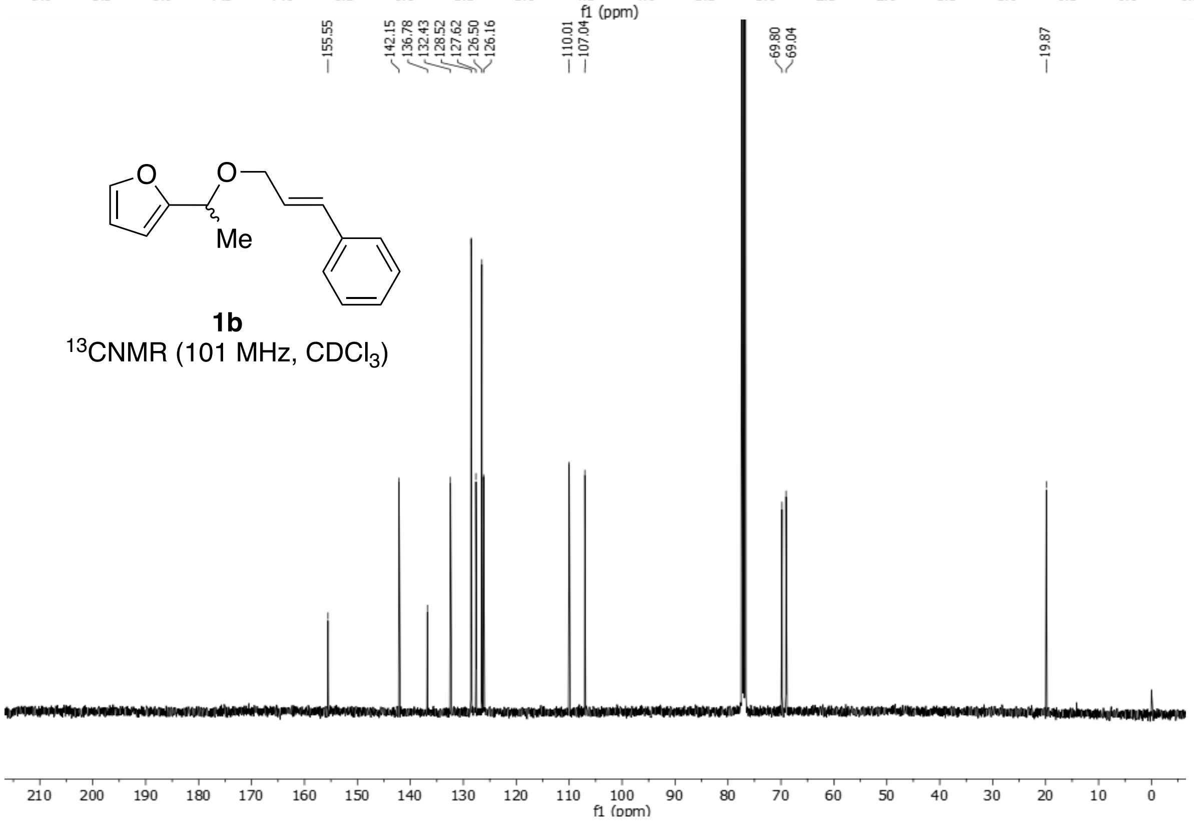
155.55
142.15 136.78 132.43 128.52 127.62 126.50 126.16
110.01 107.04
69.80 69.04
19.87
O
O
Me
1b
^{13}CNMR (101 MHz, $CDCl_3$)
210 200 190 180 170 160 150 140 130 120 110 100 90 80 70 60 50 40 30 20 10 0
f1 (ppm)

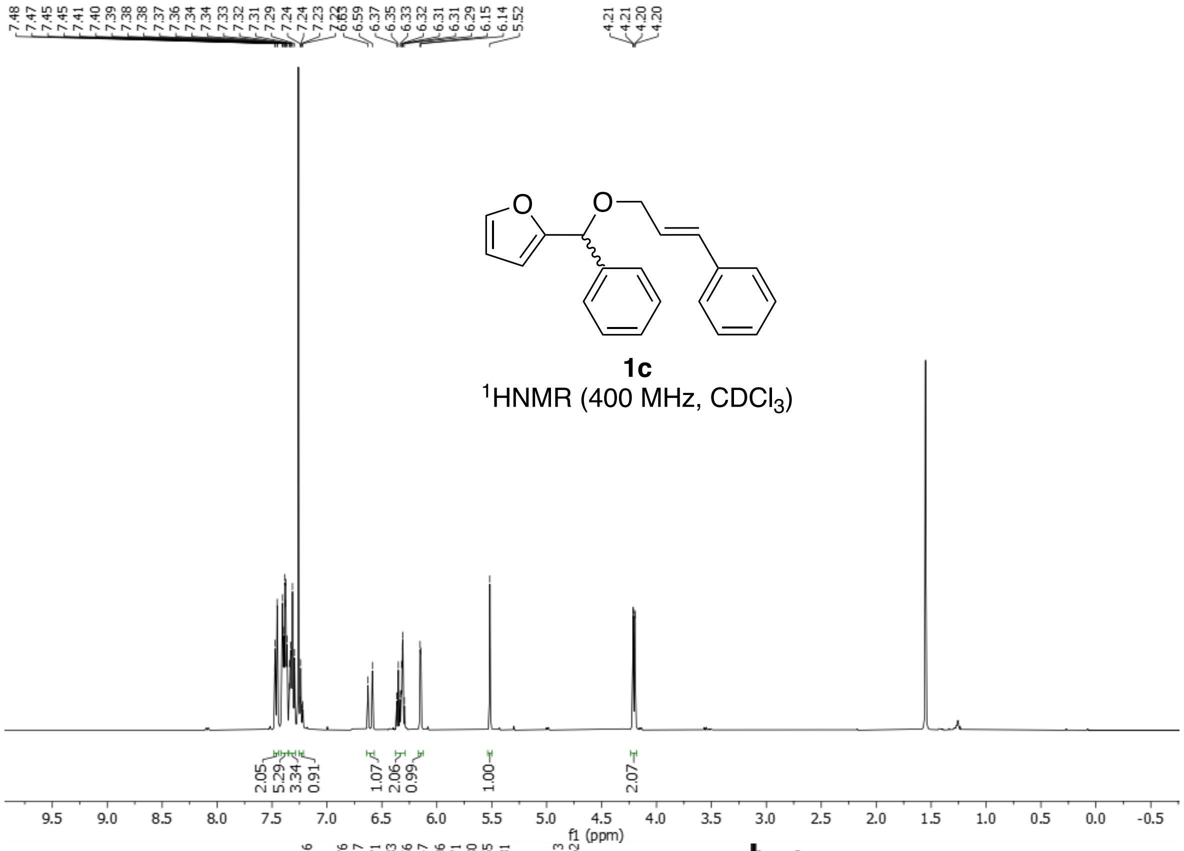

1c

^{1}HNMR (400 MHz, $CDCl_3$)

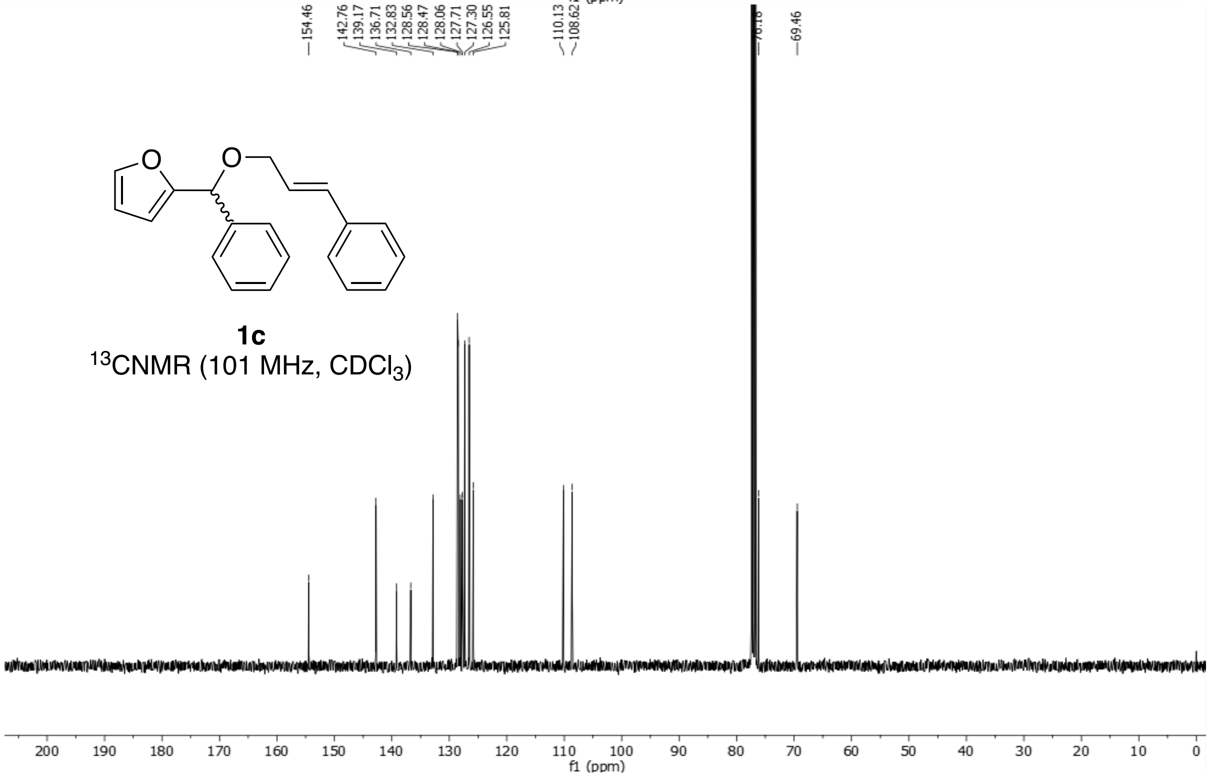

1c

^{13}CNMR (101 MHz, $CDCl_3$)

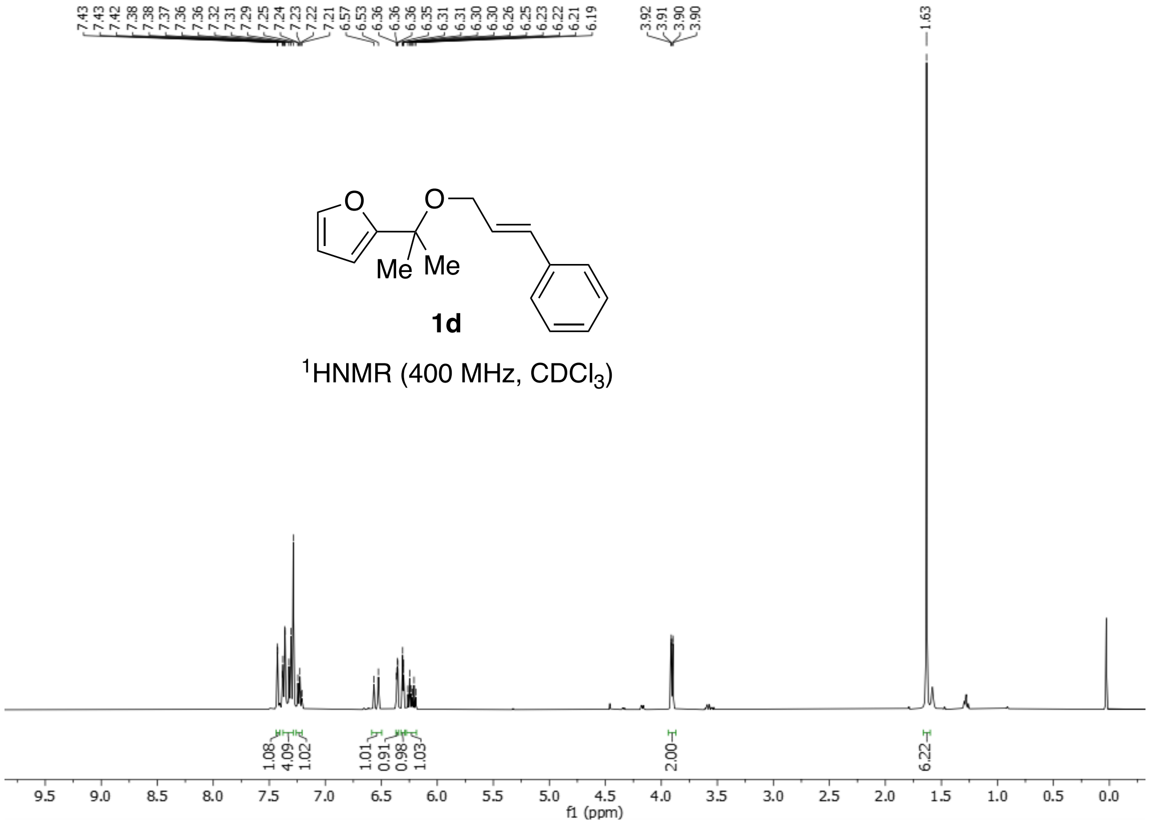
7.43
7.43
7.42
7.38
7.38
7.37
7.36
7.36
7.32
7.31
7.29
7.25
7.24
7.23
7.22
7.21
6.57
6.53
6.36
6.36
6.36
6.35
6.31
6.31
6.30
6.30
6.26
6.25
6.23
6.22
6.21
6.19
3.92
3.91
3.90
3.90
1.63
O
O
Me
Me
1d
^{1}HNMR (400 MHz, CDCl$_3$)
1.08
4.09
1.02
1.01
0.91
0.98
1.03
2.00
6.22
f1 (ppm)

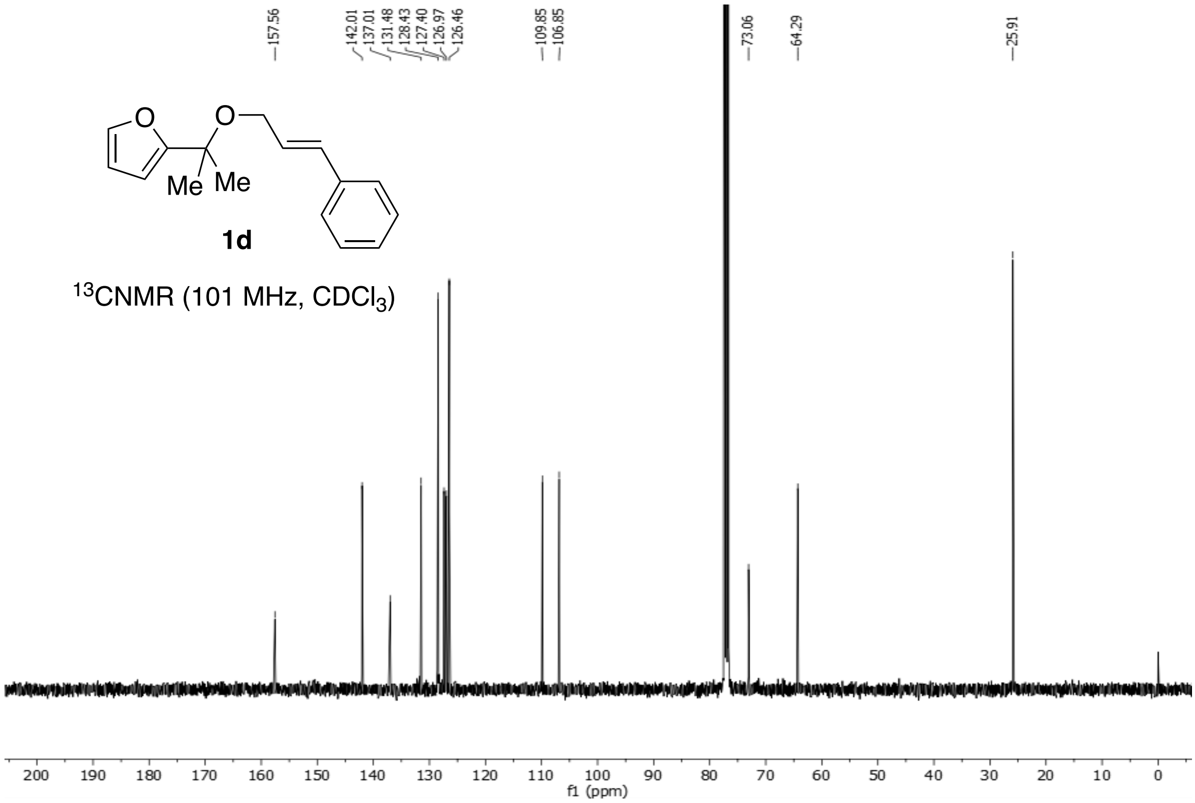
157.56
142.01
137.01
131.48
128.43
127.40
126.97
126.46
109.85
106.85
73.06
64.29
25.91
O
O
Me
Me
1d
^{13}CNMR (101 MHz, CDCl$_3$)
f1 (ppm)

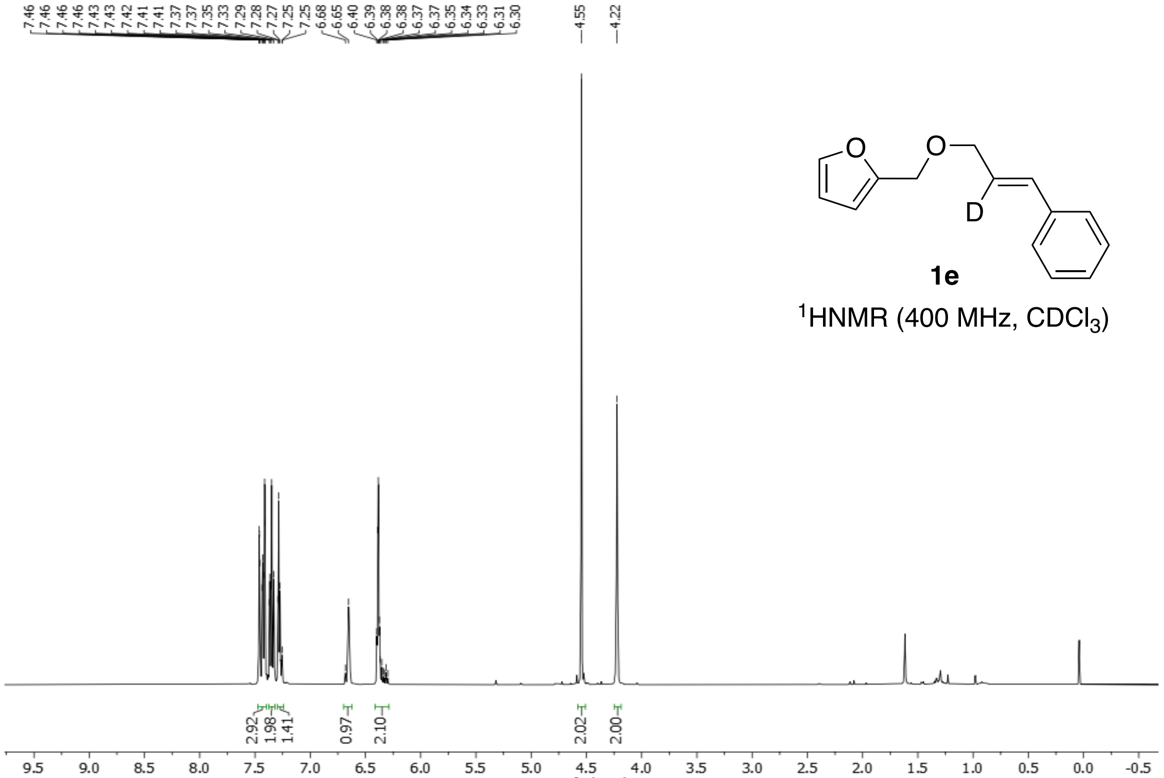
1e
1HNMR (400 MHz, CDCl3)
f1 (ppm)

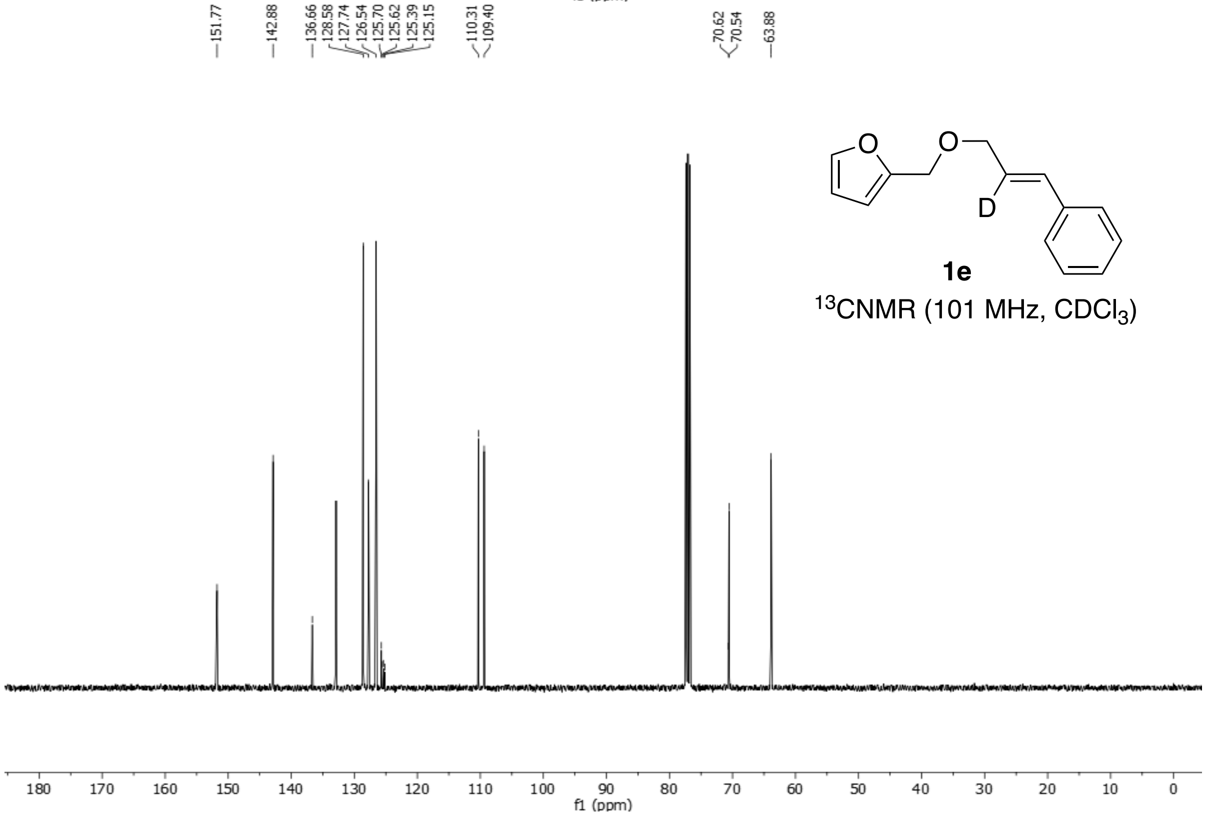
1e
13CNMR (101 MHz, CDCl3)
f1 (ppm)

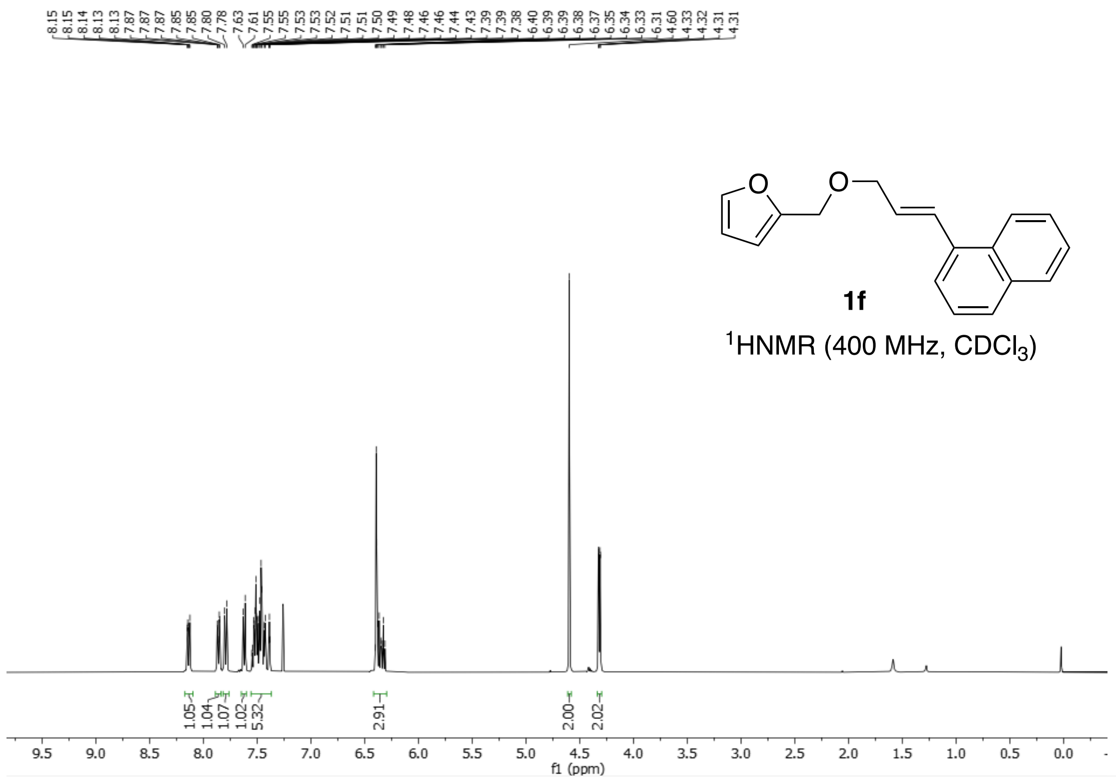
8.15
8.15
8.14
8.13
8.13
7.87
7.87
7.87
7.85
7.85
7.80
7.78
7.63
7.61
7.55
7.55
7.53
7.53
7.52
7.51
7.51
7.50
7.49
7.48
7.46
7.46
7.44
7.43
7.39
7.39
7.38
6.40
6.39
6.39
6.38
6.37
6.35
6.34
6.33
6.31
4.60
4.33
4.32
4.31
4.31
O
O
1f
¹HNMR (400 MHz, CDCl₃)
1.05
1.04
1.07
1.02
5.32
2.91
2.00
2.02
9.5
9.0
8.5
8.0
7.5
7.0
6.5
6.0
5.5
5.0
4.5
4.0
3.5
3.0
2.5
2.0
1.5
1.0
0.5
0.0
f1 (ppm)

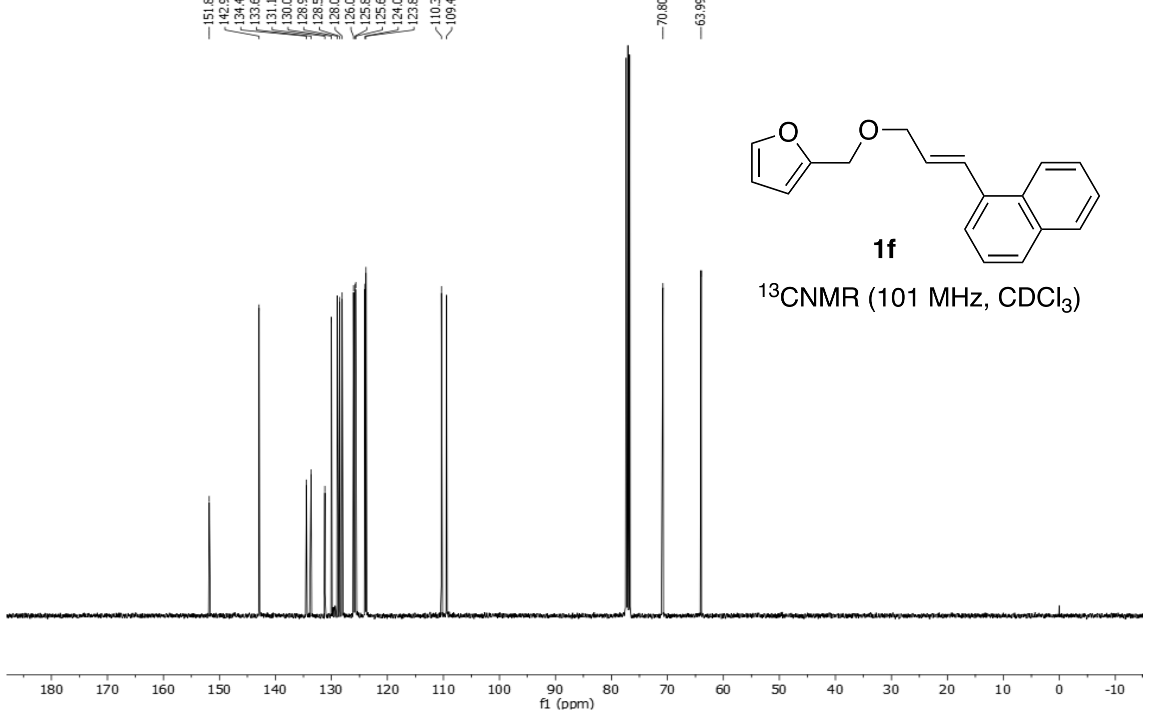
151.82
142.92
134.46
133.62
131.17
130.01
128.95
128.55
128.08
126.07
125.80
125.64
124.01
123.83
110.36
109.47
70.80
63.99
O
O
1f
¹³CNMR (101 MHz, CDCl₃)
180
170
160
150
140
130
120
110
100
90
80
70
60
50
40
30
20
10
0
-10
f1 (ppm)

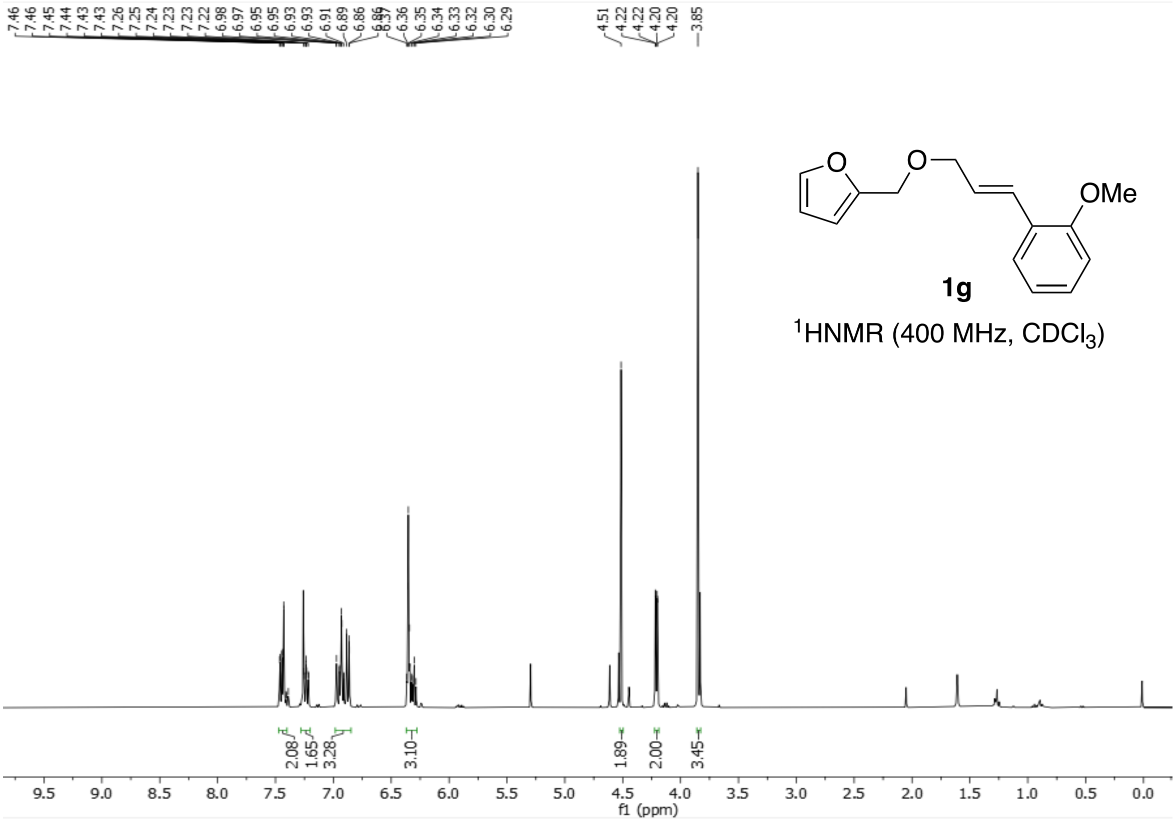

7.46
7.46
7.45
7.44
7.43
7.43
7.26
7.25
7.24
7.23
7.23
7.22
6.98
6.97
6.95
6.95
6.93
6.93
6.91
6.89
6.86
6.37
6.36
6.35
6.34
6.33
6.32
6.30
6.29
4.51
4.22
4.22
4.20
4.20
3.85
OMe
1g
1HNMR (400 MHz, CDCl3)
2.08
1.65
3.28
3.10
1.89
2.00
3.45
9.5
9.0
8.5
8.0
7.5
7.0
6.5
6.0
5.5
5.0
4.5
4.0
3.5
3.0
2.5
2.0
1.5
1.0
0.5
0.0
f1 (ppm)

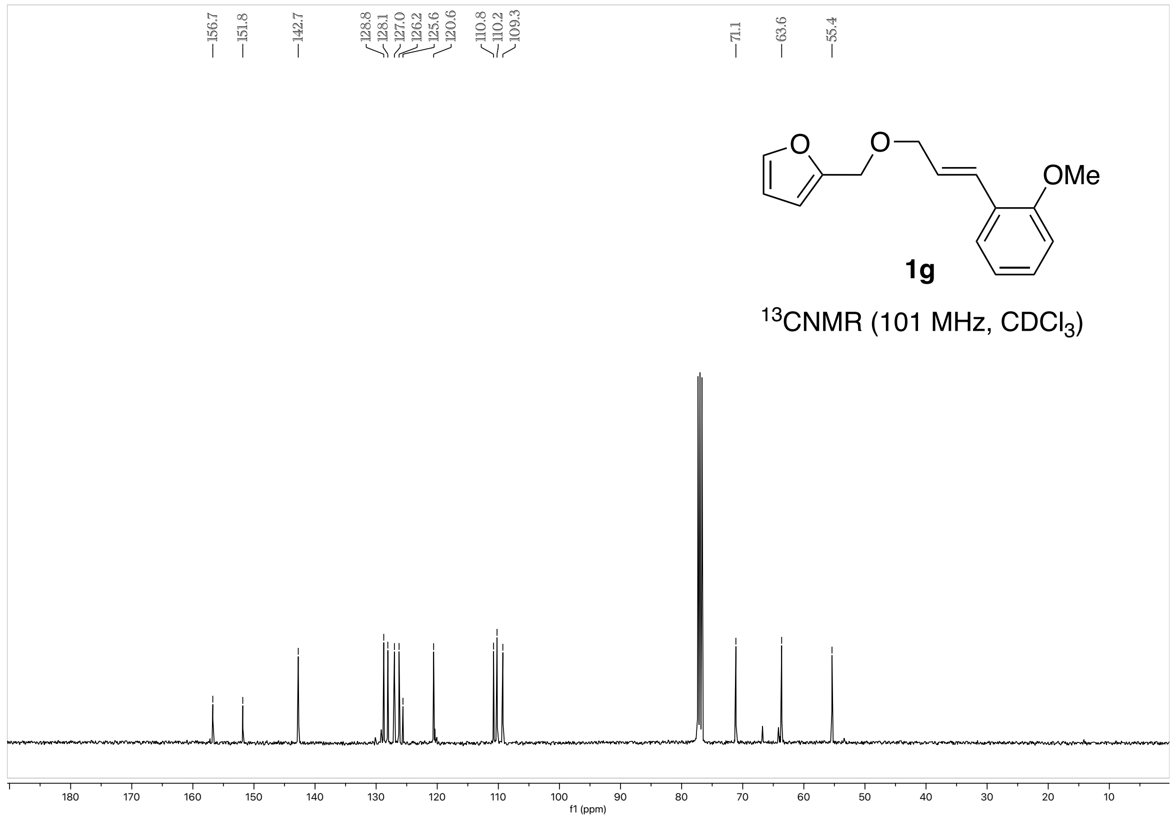

156.7
151.8
142.7
128.8
128.1
127.0
126.2
125.6
120.6
110.8
110.2
109.3
71.1
63.6
55.4
OMe
1g
13CNMR (101 MHz, CDCl3)
180
170
160
150
140
130
120
110
100
90
80
70
60
50
40
30
20
10
f1 (ppm)

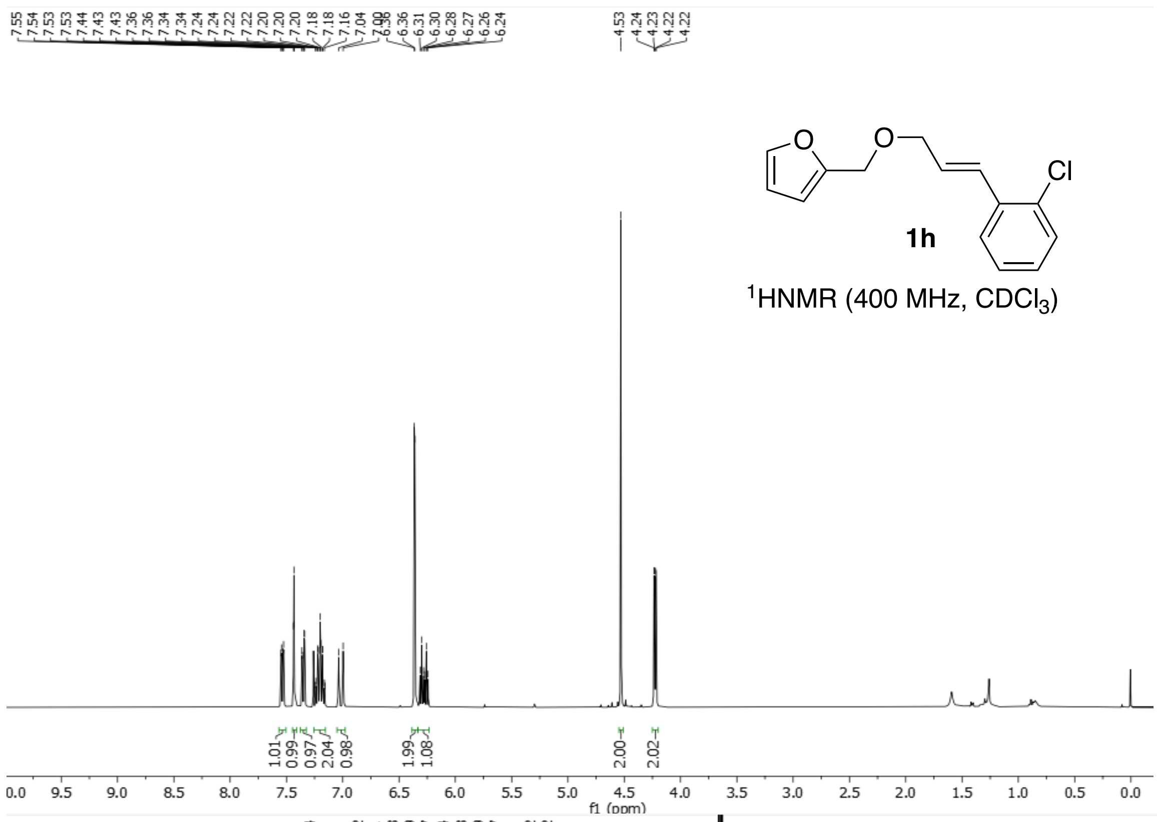

1h
^{1}HNMR (400 MHz, CDCl$_3$)

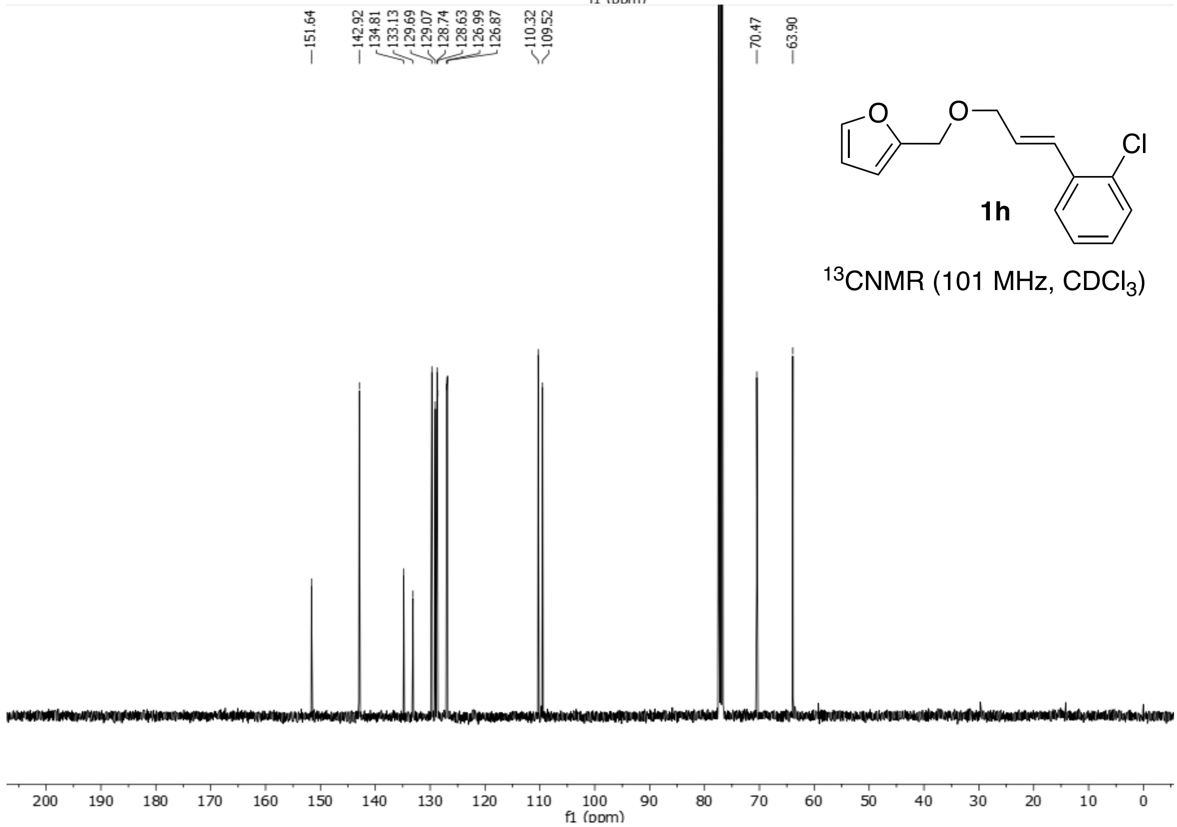

1h
^{13}CNMR (101 MHz, CDCl$_3$)

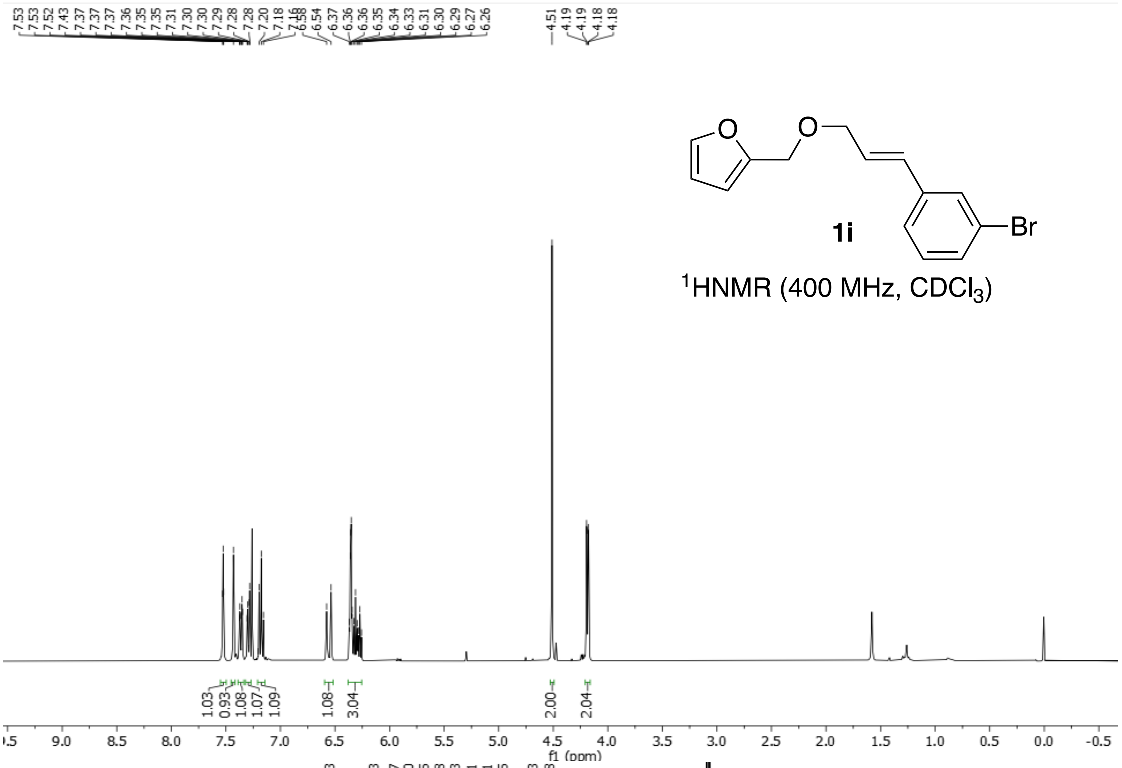
7.53 7.53 7.52 7.43 7.37 7.37 7.37 7.36 7.35 7.35 7.31 7.30 7.30 7.29 7.28 7.28 7.20 7.18 7.16 6.58 6.54 6.37 6.36 6.36 6.35 6.35 6.34 6.33 6.31 6.30 6.29 6.27 6.26
4.51 4.19 4.19 4.18 4.18
O
O
Br
1i
^{1}HNMR (400 MHz, $CDCl_3$)
1.03 0.93 1.08 1.07 1.09
1.08 3.04
2.00 2.04
f1 (ppm)

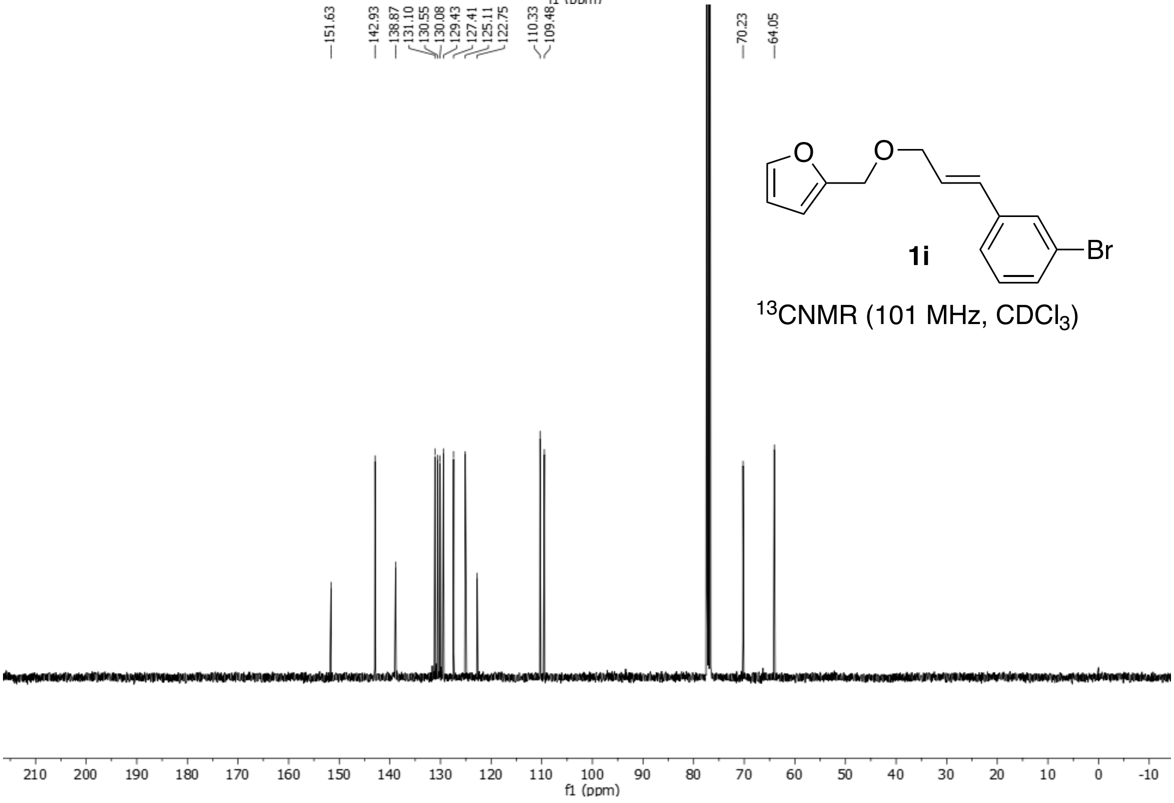
151.63 142.93 138.87 131.10 130.55 130.08 129.43 127.41 125.11 122.75 110.33 109.48 70.23 64.05
O
O
Br
1i
^{13}CNMR (101 MHz, $CDCl_3$)
f1 (ppm)

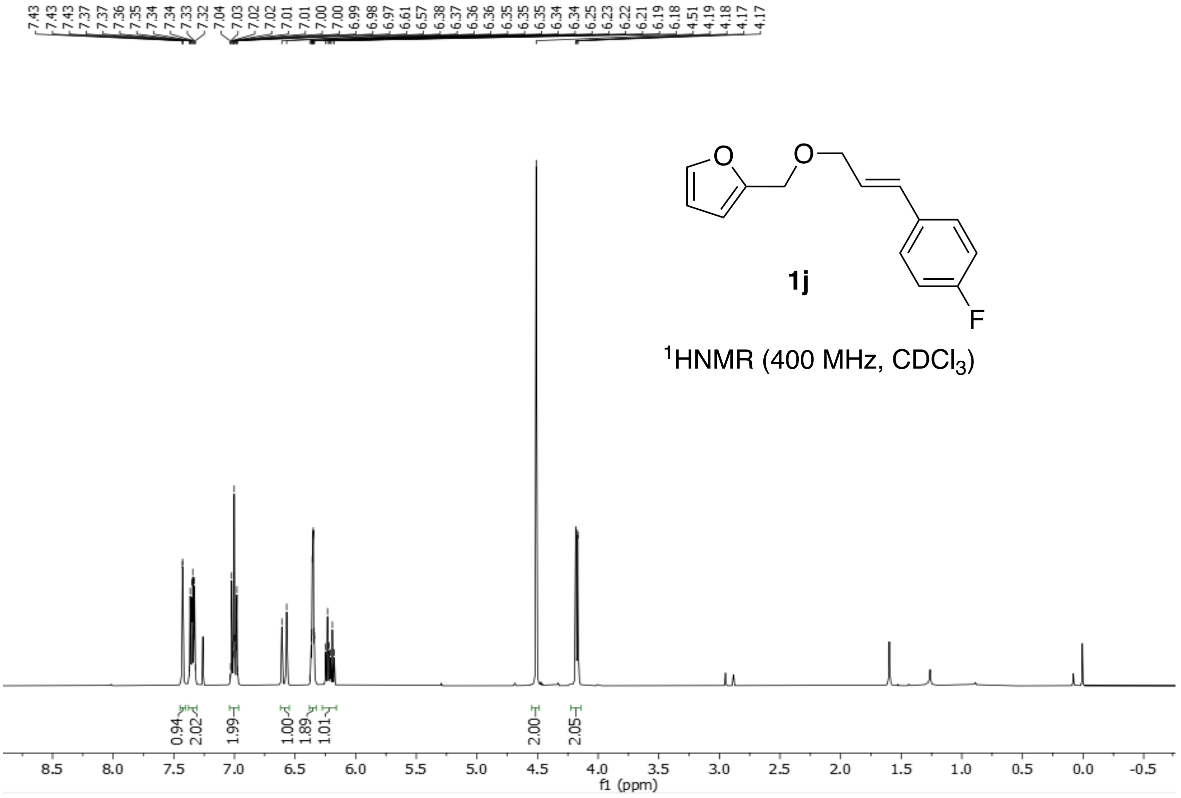
1j
^{1}HNMR (400 MHz, $CDCl_3$)
f1 (ppm)

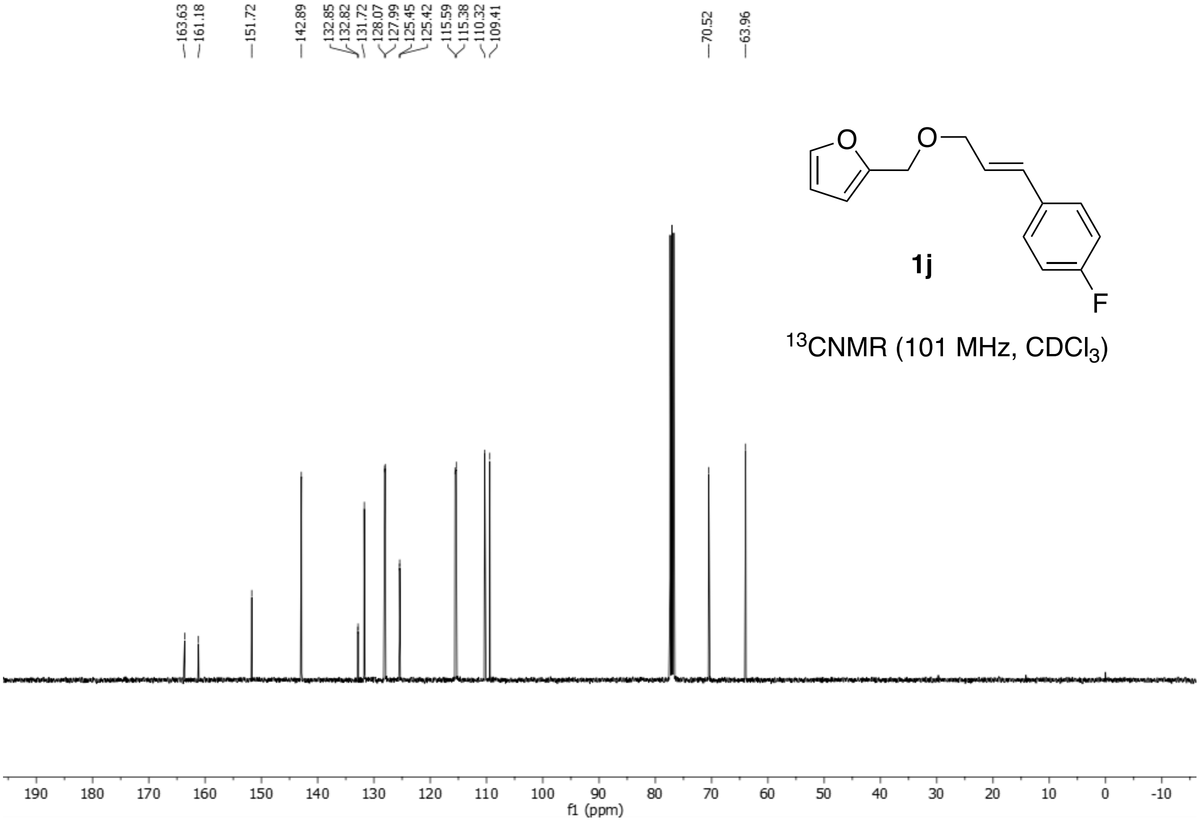
1j
^{13}CNMR (101 MHz, $CDCl_3$)
f1 (ppm)

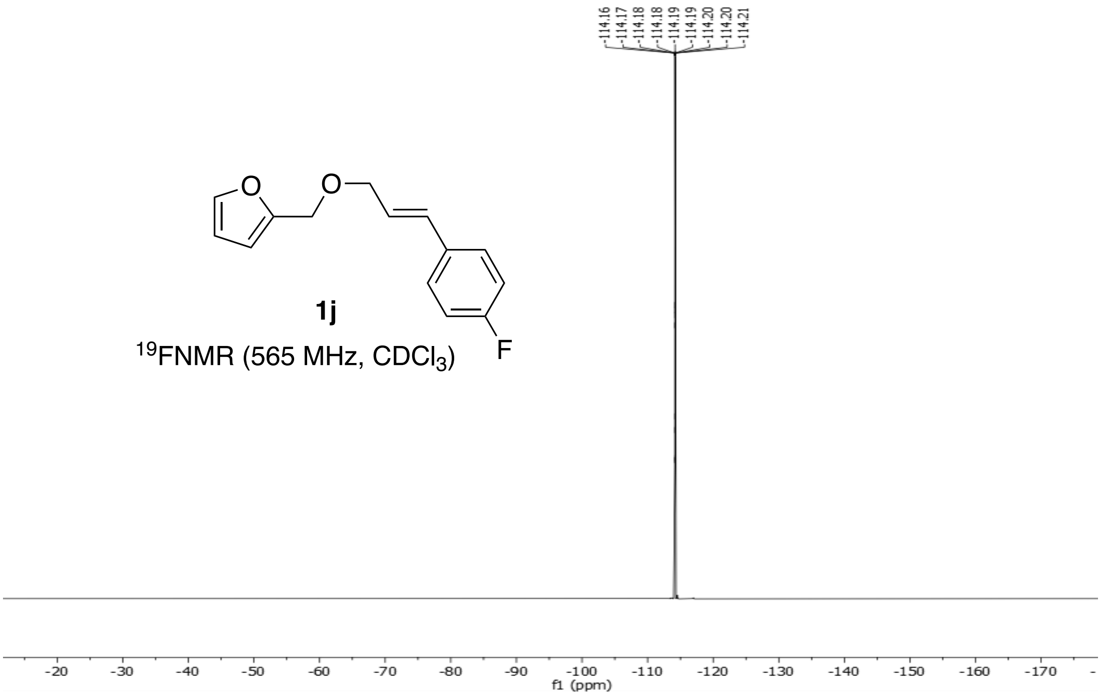
-114.16
-114.17
-114.18
-114.18
-114.19
-114.19
-114.20
-114.20
-114.21
O
O
F
1j
^{19}FNMR (565 MHz, CDCl$_3$)
-20
-30
-40
-50
-60
-70
-80
-90
-100
-110
-120
-130
-140
-150
-160
-170
f1 (ppm)

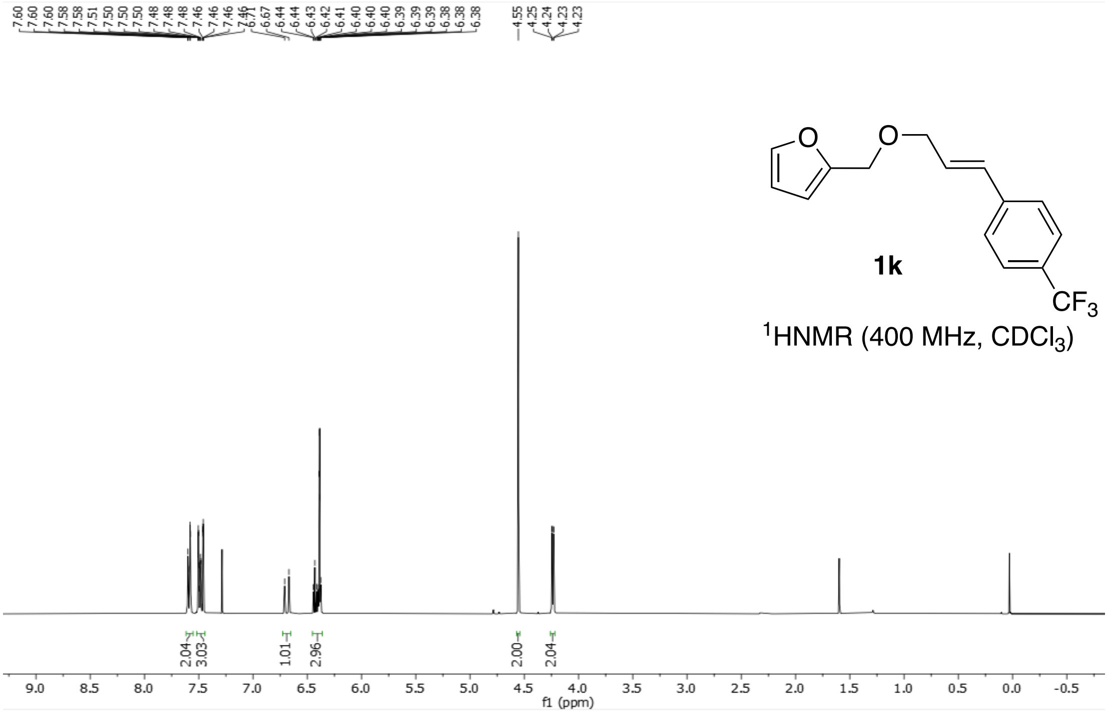
1k
^{1}HNMR (400 MHz, $CDCl_3$)
f1 (ppm)

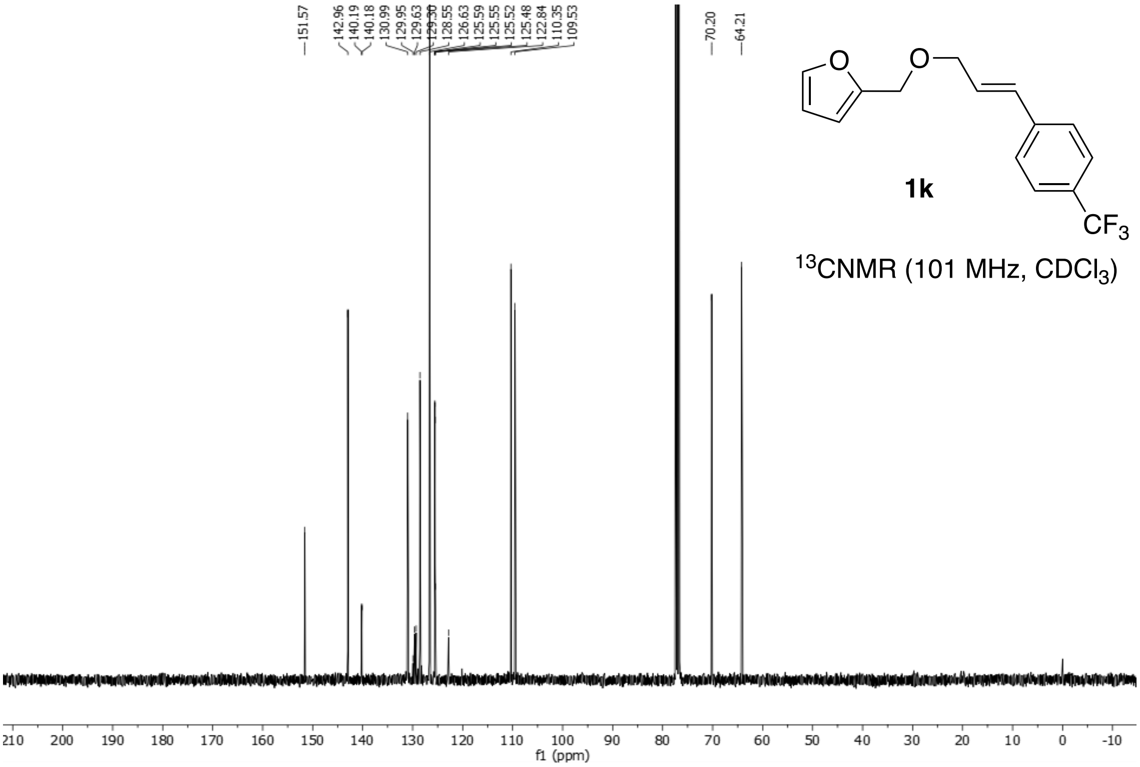
1k
^{13}CNMR (101 MHz, $CDCl_3$)
f1 (ppm)

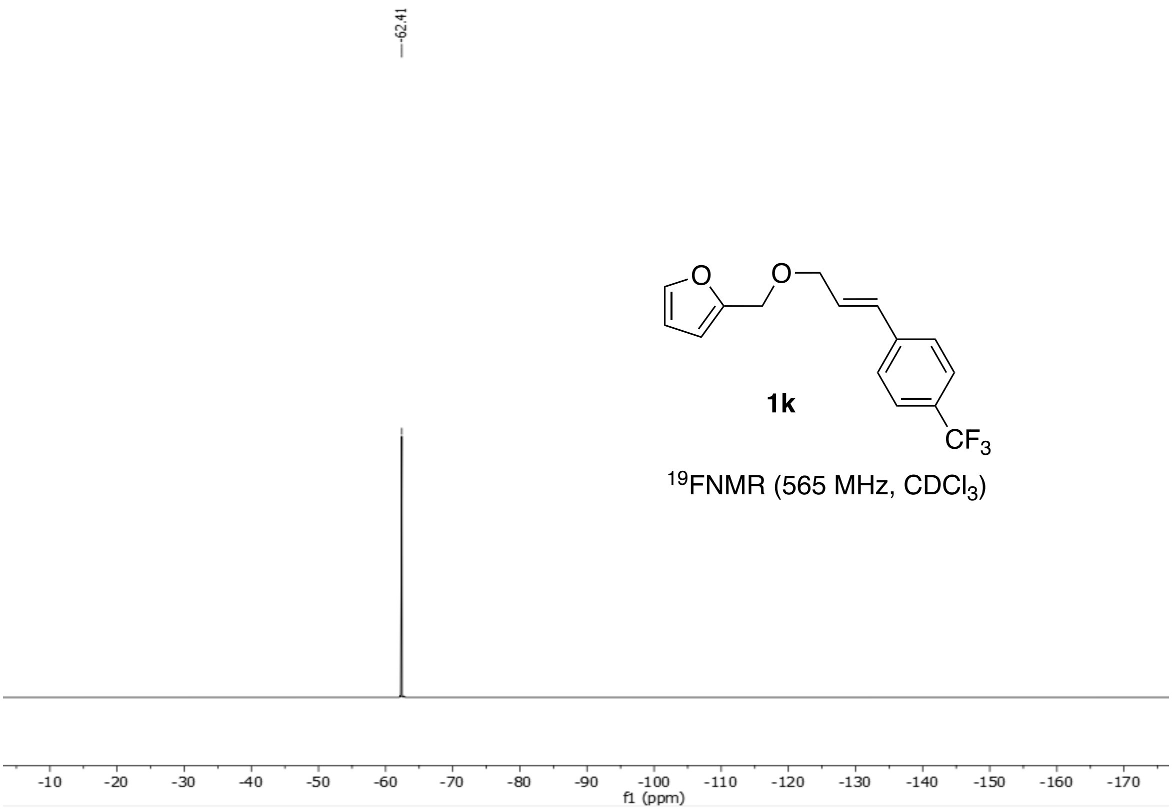

-62.41
1k
CF_3
^{19}FNMR (565 MHz, $CDCl_3$)
-10
-20
-30
-40
-50
-60
-70
-80
-90
-100
-110
-120
-130
-140
-150
-160
-170
f1 (ppm)

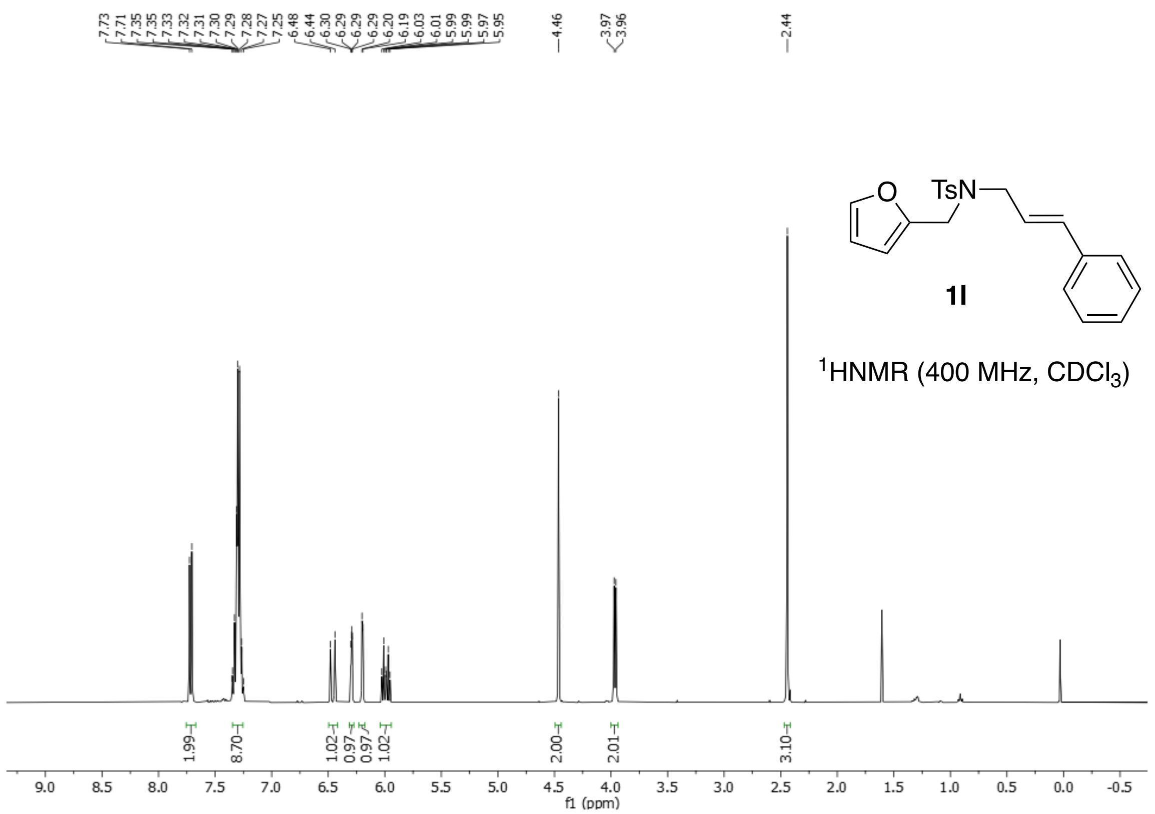
7.73 7.71 7.35 7.35 7.33 7.32 7.31 7.30 7.29 7.28 7.27 7.25 6.48 6.44 6.30 6.29 6.29 6.29 6.20 6.19 6.03 6.01 5.99 5.99 5.97 5.95
4.46
3.97 3.96
2.44
O
TsN
1l
1HNMR (400 MHz, CDCl3)
1.99 8.70 1.02 0.97 0.97 1.02 2.00 2.01 3.10
9.0 8.5 8.0 7.5 7.0 6.5 6.0 5.5 5.0 4.5 4.0 3.5 3.0 2.5 2.0 1.5 1.0 0.5 0.0 -0.5
f1 (ppm)

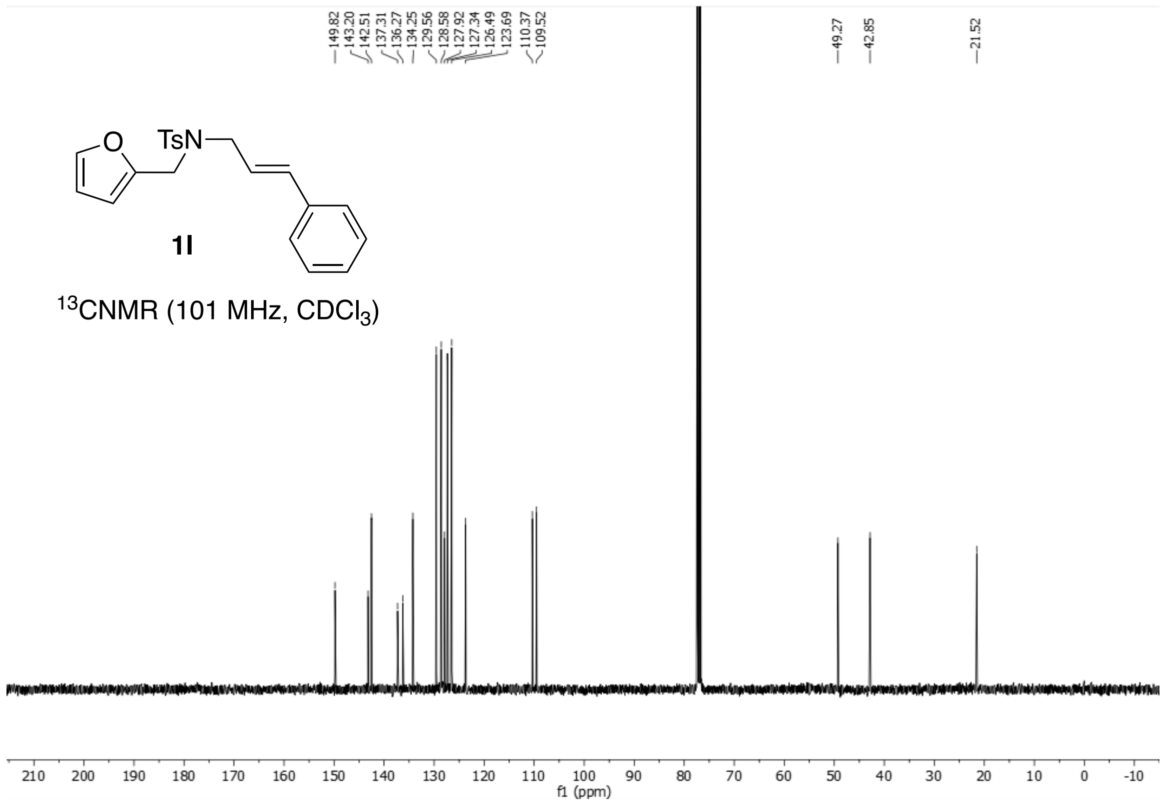
149.82 143.20 142.51 137.31 136.27 134.25 129.56 128.58 127.92 127.34 126.49 123.69 110.37 109.52
49.27
42.85
21.52
O
TsN
1l
13CNMR (101 MHz, CDCl3)
210 200 190 180 170 160 150 140 130 120 110 100 90 80 70 60 50 40 30 20 10 0 -10
f1 (ppm)

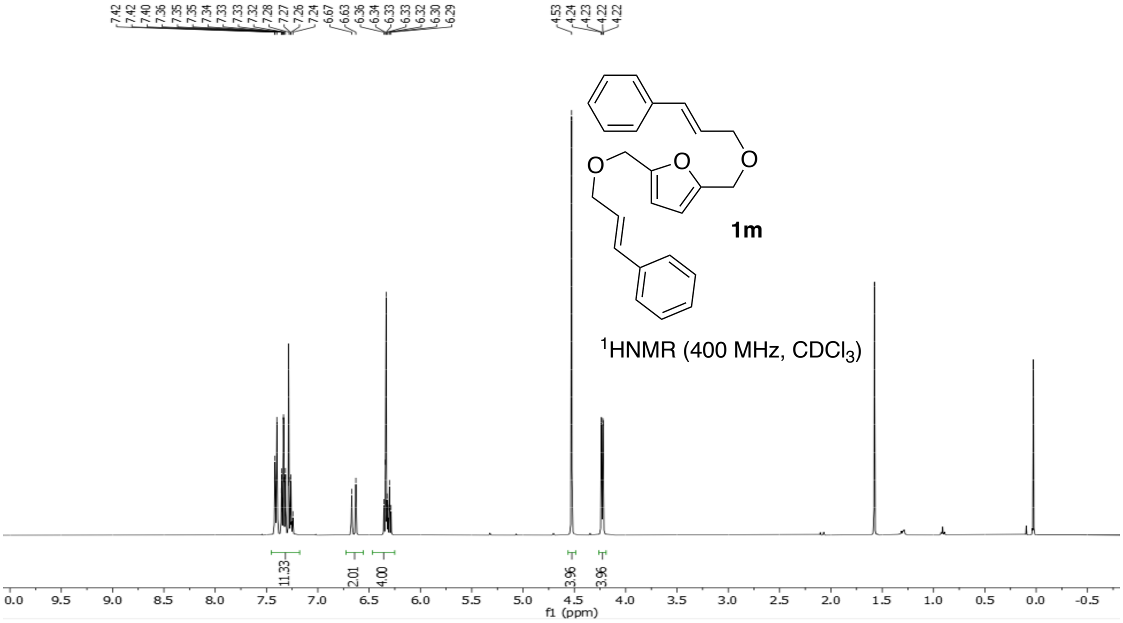
7.42 7.42 7.40 7.36 7.35 7.35 7.34 7.33 7.33 7.32 7.28 7.27 7.26 7.24 6.67 6.63 6.36 6.34 6.33 6.33 6.32 6.30 6.29
4.53 4.24 4.23 4.22 4.22
1m
1HNMR (400 MHz, CDCl3)
11.33
2.01
4.00
3.96
3.96
f1 (ppm)

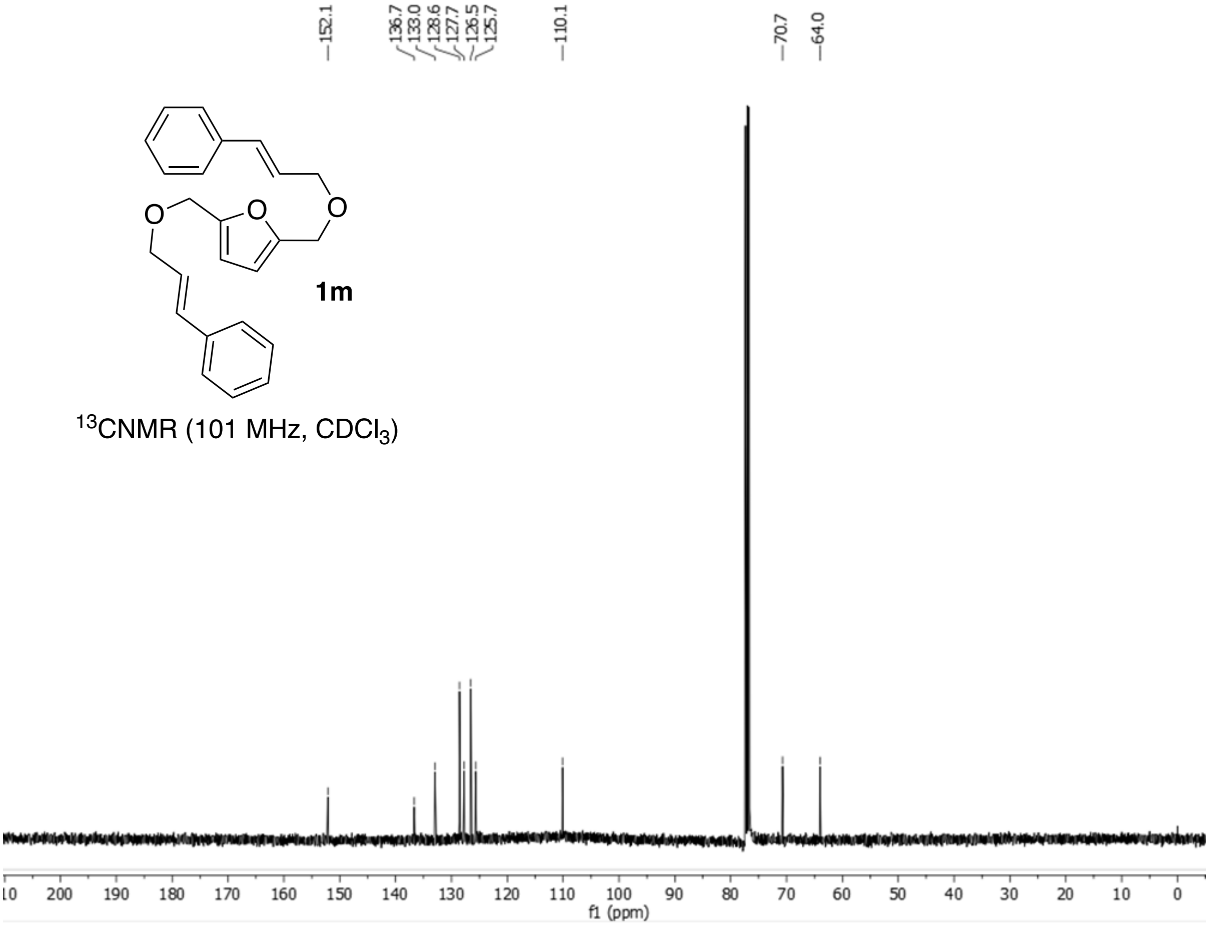
152.1
136.7
133.0
128.6
127.7
126.5
125.7
110.1
70.7
64.0
1m
13CNMR (101 MHz, CDCl3)
f1 (ppm)

a

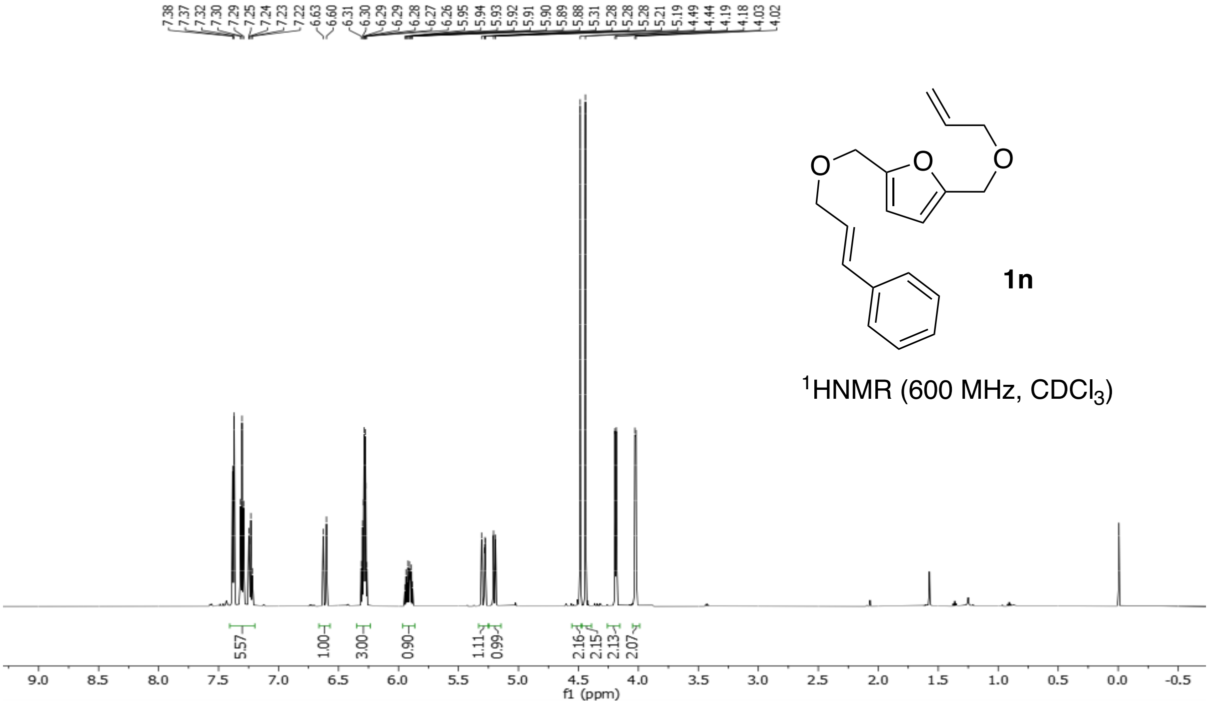
7.38
7.37
7.32
7.30
7.29
7.25
7.24
7.23
7.22
6.63
6.60
6.31
6.30
6.29
6.29
6.28
6.27
6.26
5.95
5.94
5.93
5.92
5.91
5.90
5.89
5.88
5.31
5.28
5.28
5.28
5.21
5.19
4.49
4.44
4.19
4.18
4.03
4.02
1n
^{1}HNMR (600 MHz, $CDCl_3$)
5.57
1.00
3.00
0.90
1.11
0.99
2.16
2.15
2.13
2.07
9.0
8.5
8.0
7.5
7.0
6.5
6.0
5.5
5.0
4.5
4.0
3.5
3.0
2.5
2.0
1.5
1.0
0.5
0.0
-0.5
f1 (ppm)

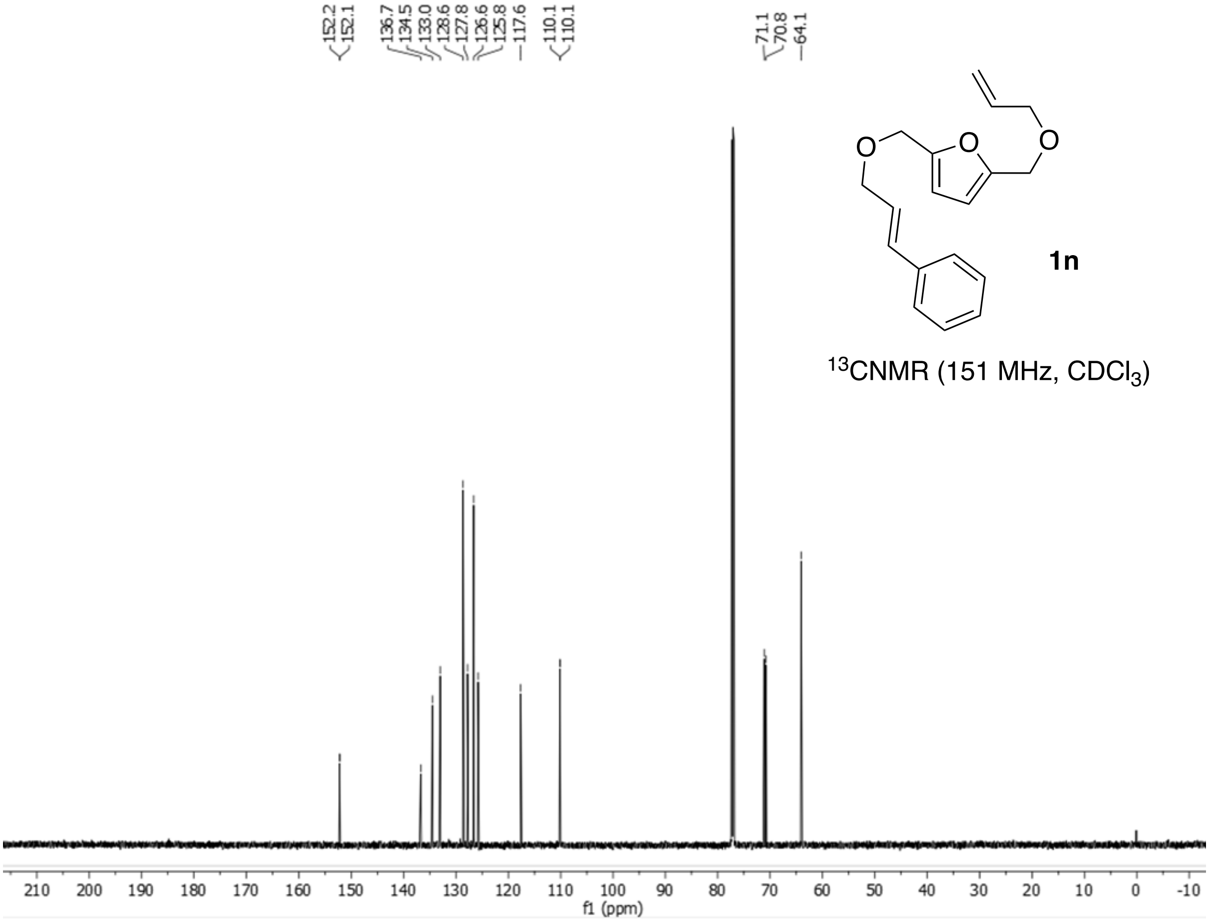
152.2
152.1
136.7
134.5
133.0
128.6
127.8
126.6
125.8
117.6
110.1
110.1
71.1
70.8
64.1
1n
^{13}CNMR (151 MHz, $CDCl_3$)
210
200
190
180
170
160
150
140
130
120
110
100
90
80
70
60
50
40
30
20
10
0
-10
f1 (ppm)

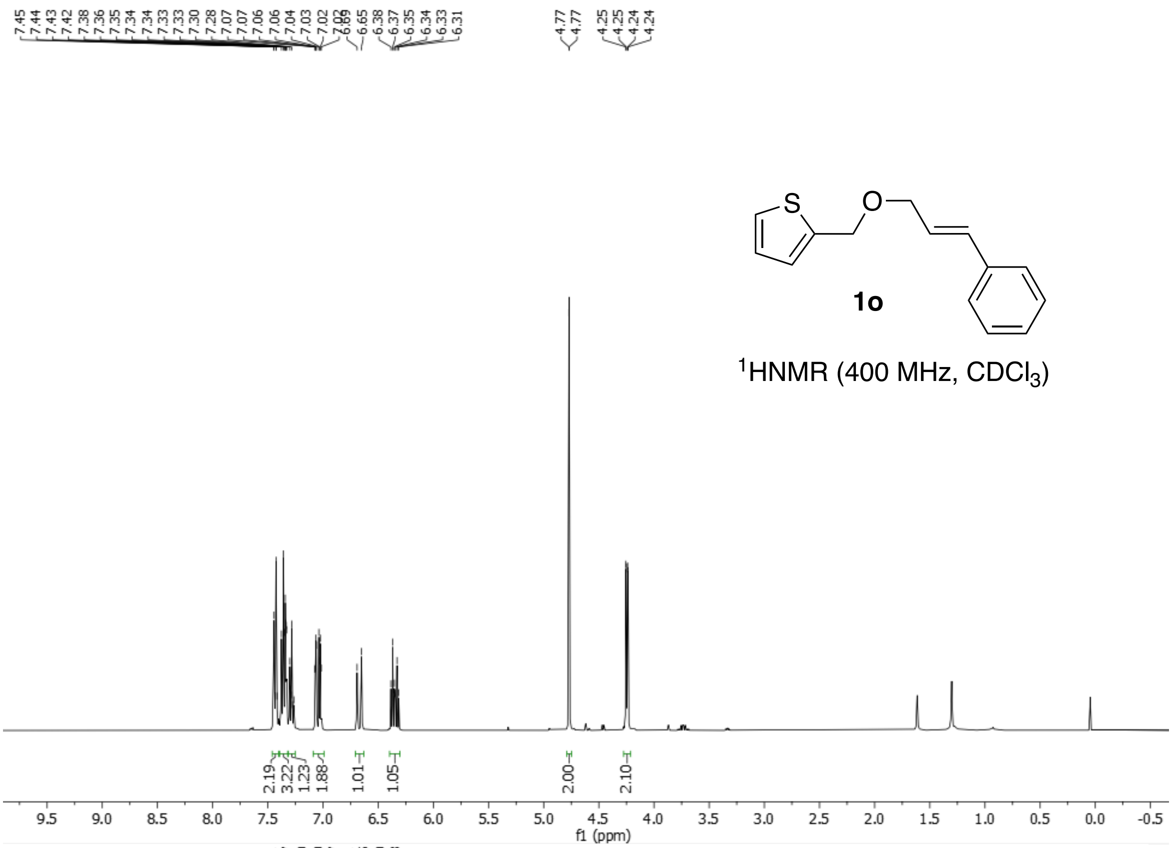
1o
^{1}HNMR (400 MHz, $CDCl_3$)
f1 (ppm)

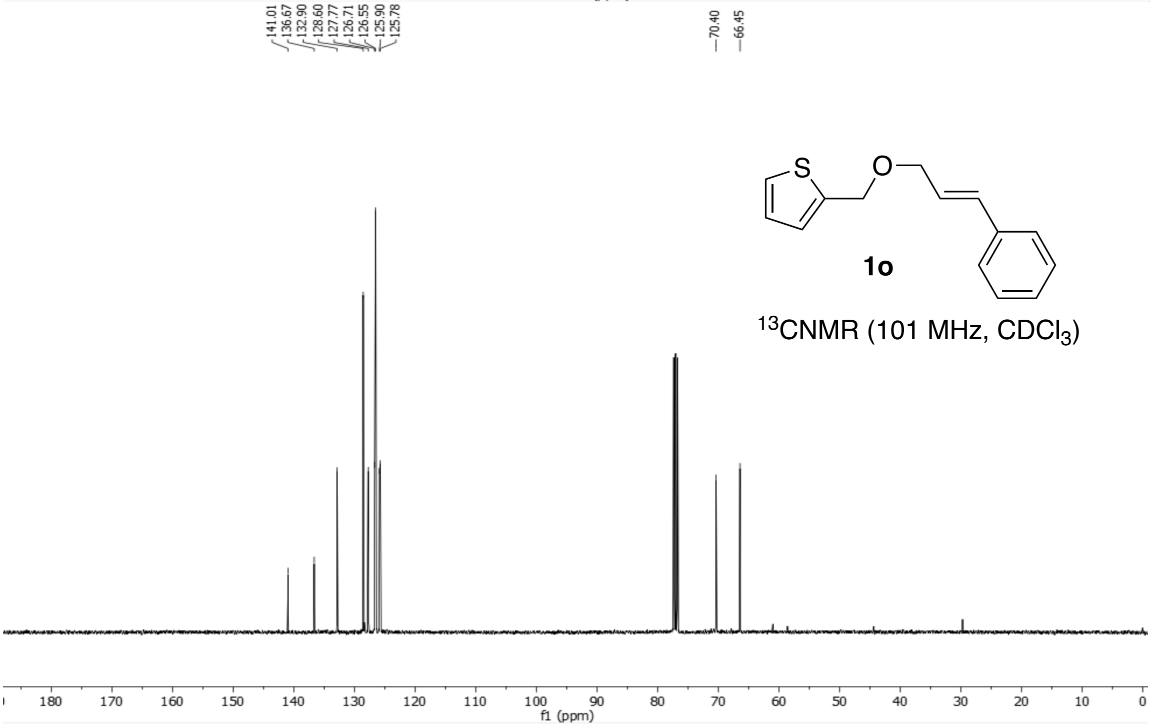
1o
^{13}CNMR (101 MHz, $CDCl_3$)
f1 (ppm)

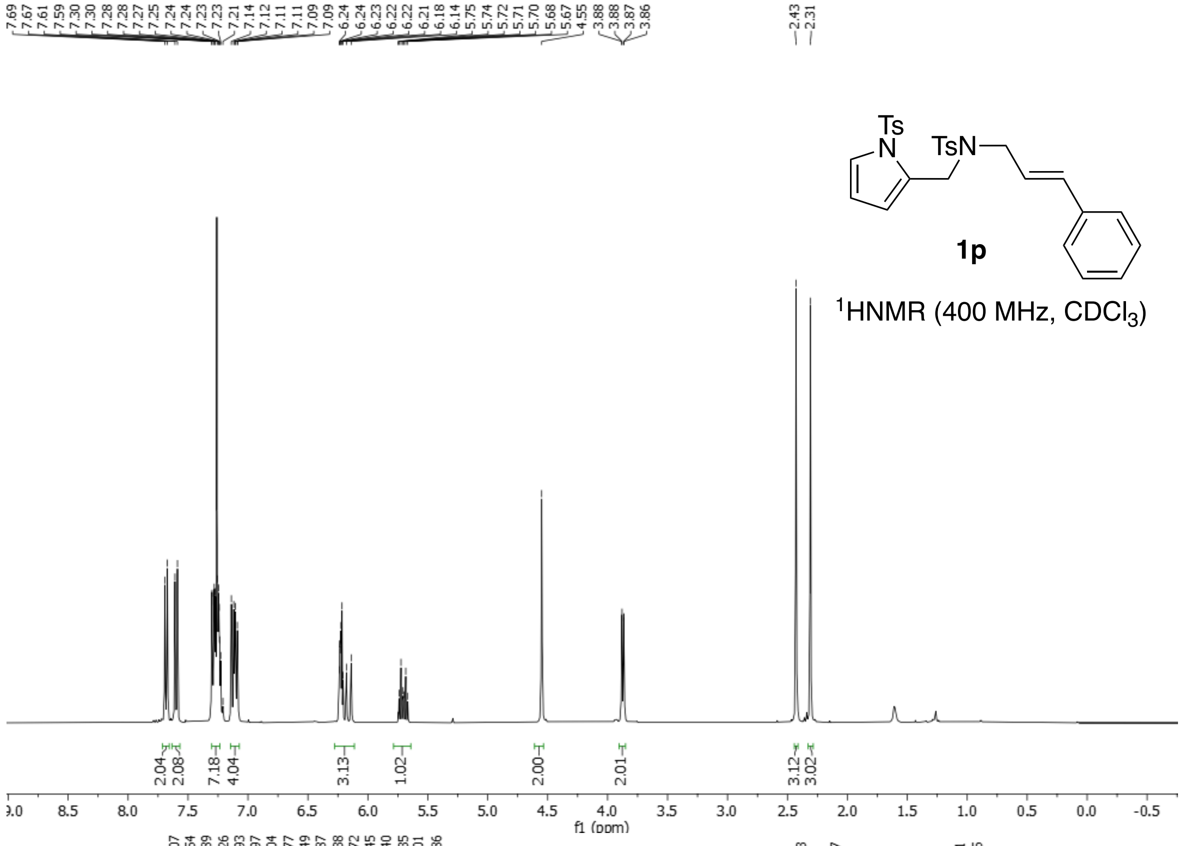

Ts
N
TsN
1p
1HNMR (400 MHz, CDCl3)
f1 (ppm)

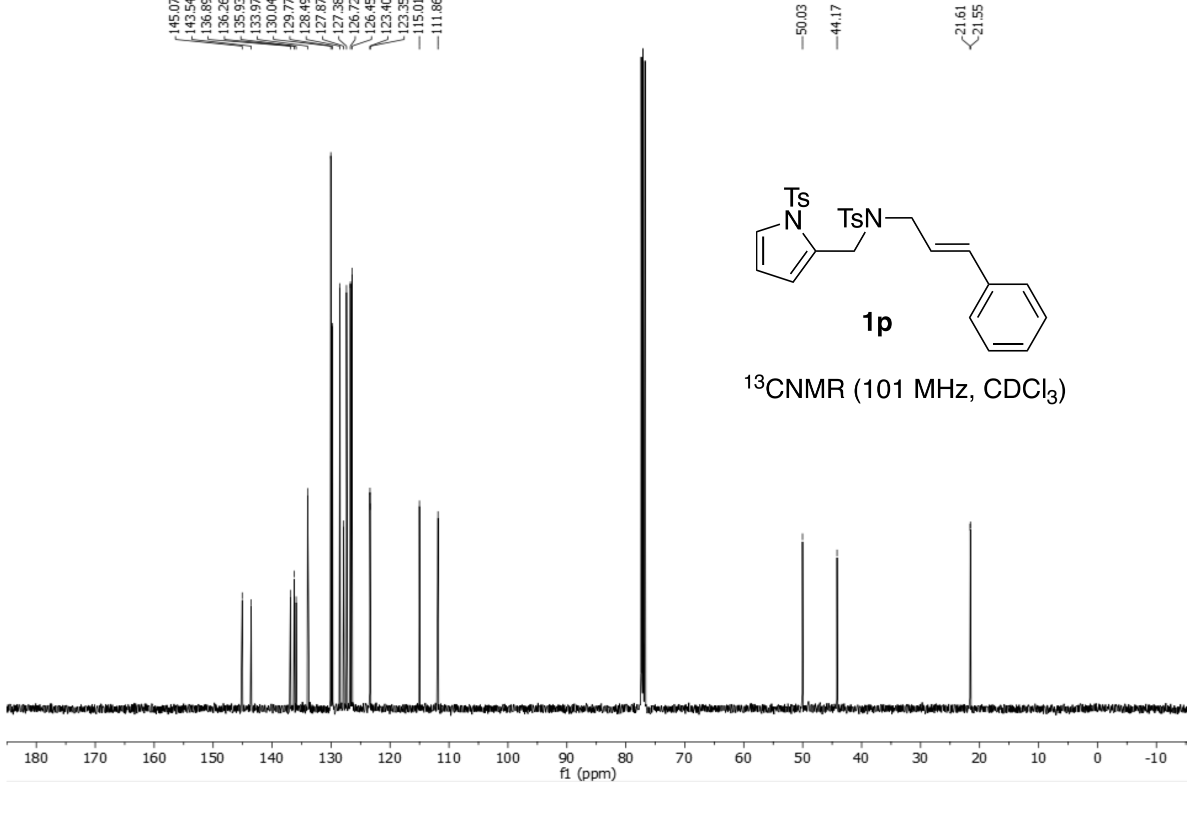

Ts
N
TsN
1p
13CNMR (101 MHz, CDCl3)
f1 (ppm)

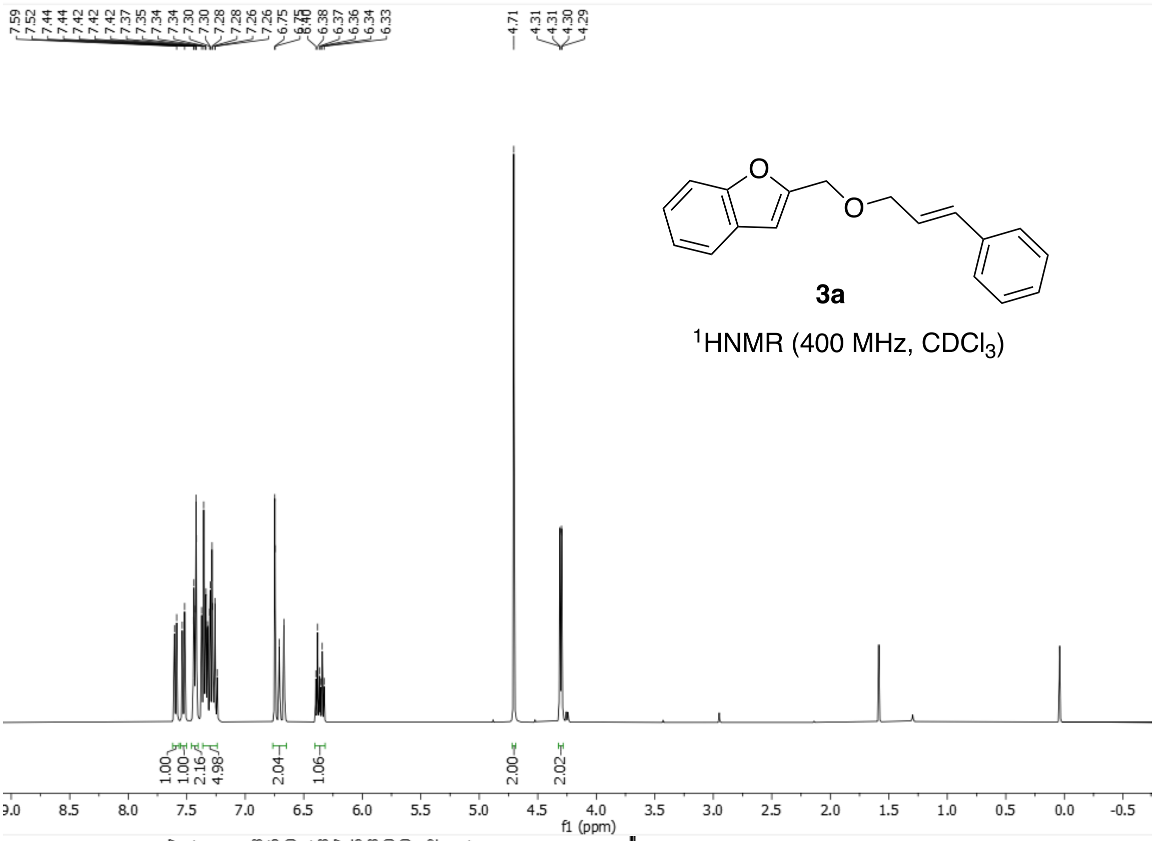
7.59
7.52
7.44
7.44
7.42
7.42
7.42
7.37
7.35
7.34
7.34
7.30
7.30
7.28
7.28
7.26
7.26
6.75
6.38
6.37
6.36
6.34
6.33
4.71
4.31
4.31
4.30
4.29
3a
¹HNMR (400 MHz, CDCl3)
1.00
1.00
2.16
4.98
2.04
1.06
2.00
2.02
f1 (ppm)

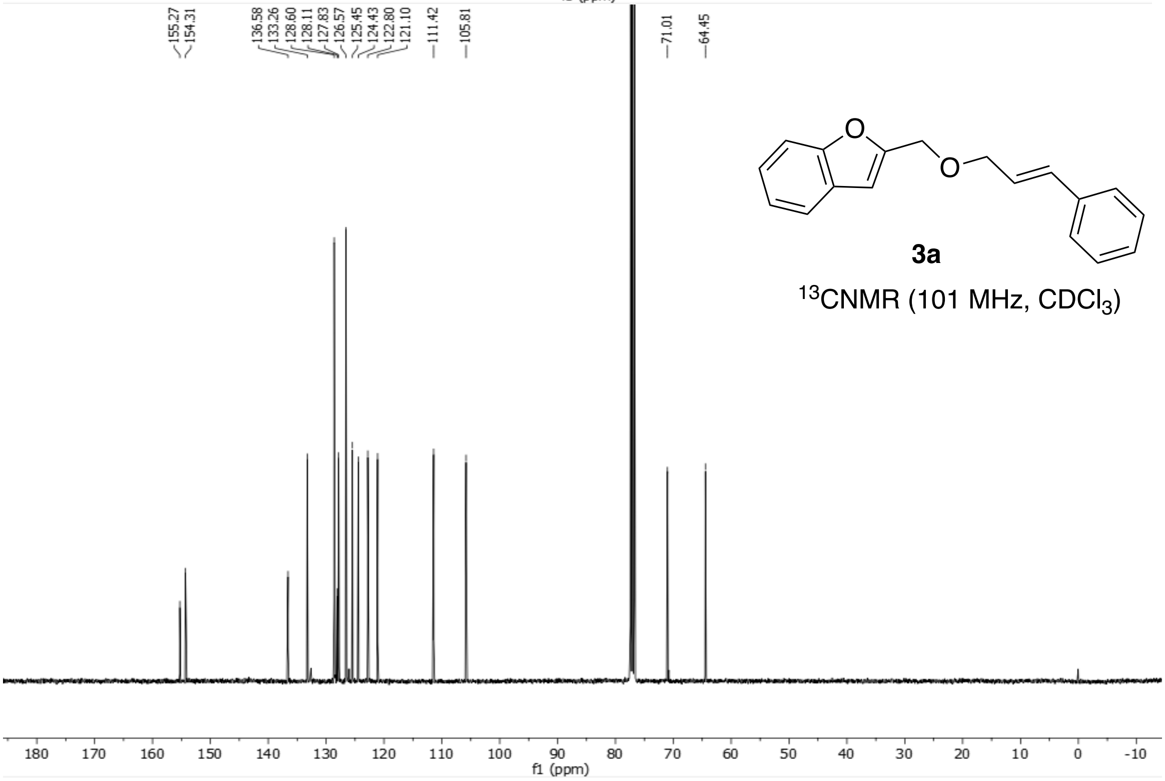
155.27
154.31
136.58
133.26
128.60
128.11
127.83
126.57
125.45
124.43
122.80
121.10
111.42
105.81
71.01
64.45
3a
¹³CNMR (101 MHz, CDCl3)
f1 (ppm)

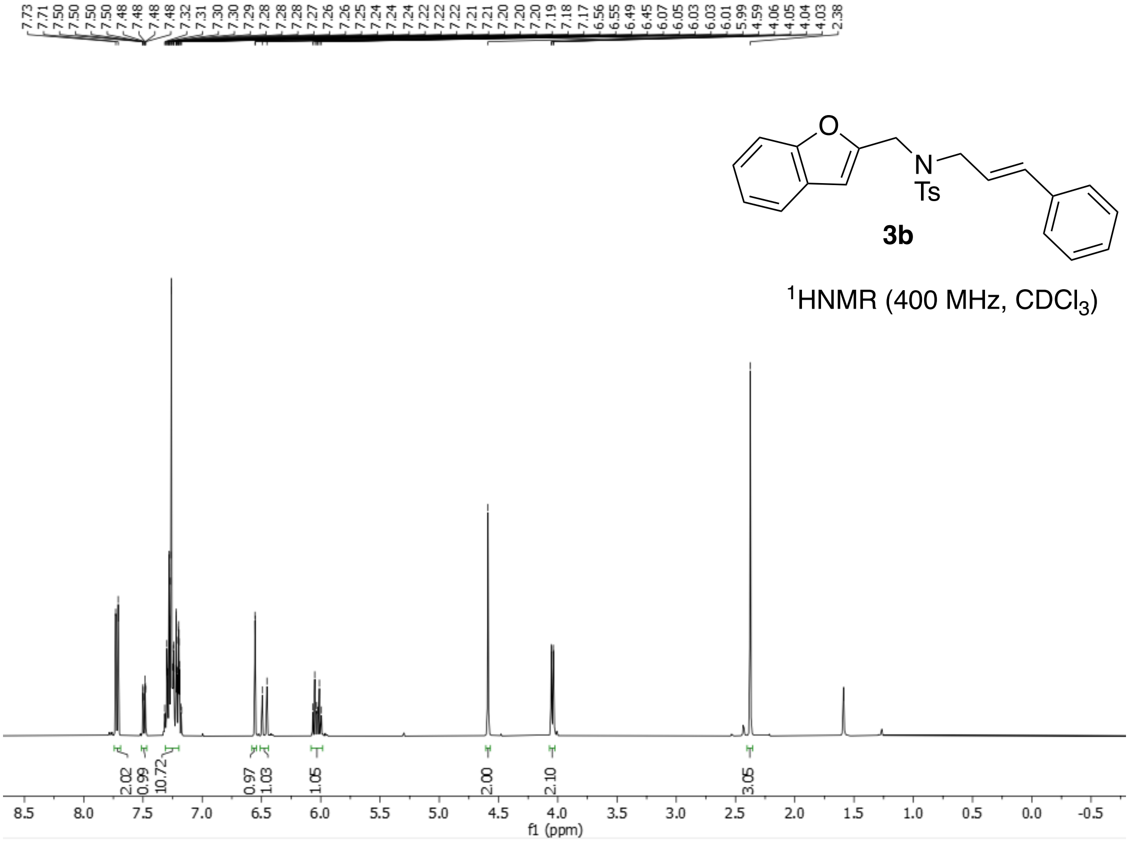
7.73 7.71 7.50 7.50 7.50 7.48 7.48 7.48 7.32 7.31 7.30 7.29 7.28 7.28 7.28 7.27 7.26 7.26 7.25 7.24 7.24 7.22 7.22 7.22 7.22 7.21 7.21 7.20 7.20 7.20 7.19 7.18 7.17 6.56 6.55 6.49 6.45 6.07 6.05 6.03 6.03 6.01 5.99 4.59 4.06 4.05 4.04 4.03 2.38
O
N
Ts
3b
^{1}HNMR (400 MHz, $CDCl_3$)
2.02 0.99 10.72 0.97 1.03 1.05 2.00 2.10 3.05
8.5 8.0 7.5 7.0 6.5 6.0 5.5 5.0 4.5 4.0 3.5 3.0 2.5 2.0 1.5 1.0 0.5 0.0 -0.5
f1 (ppm)

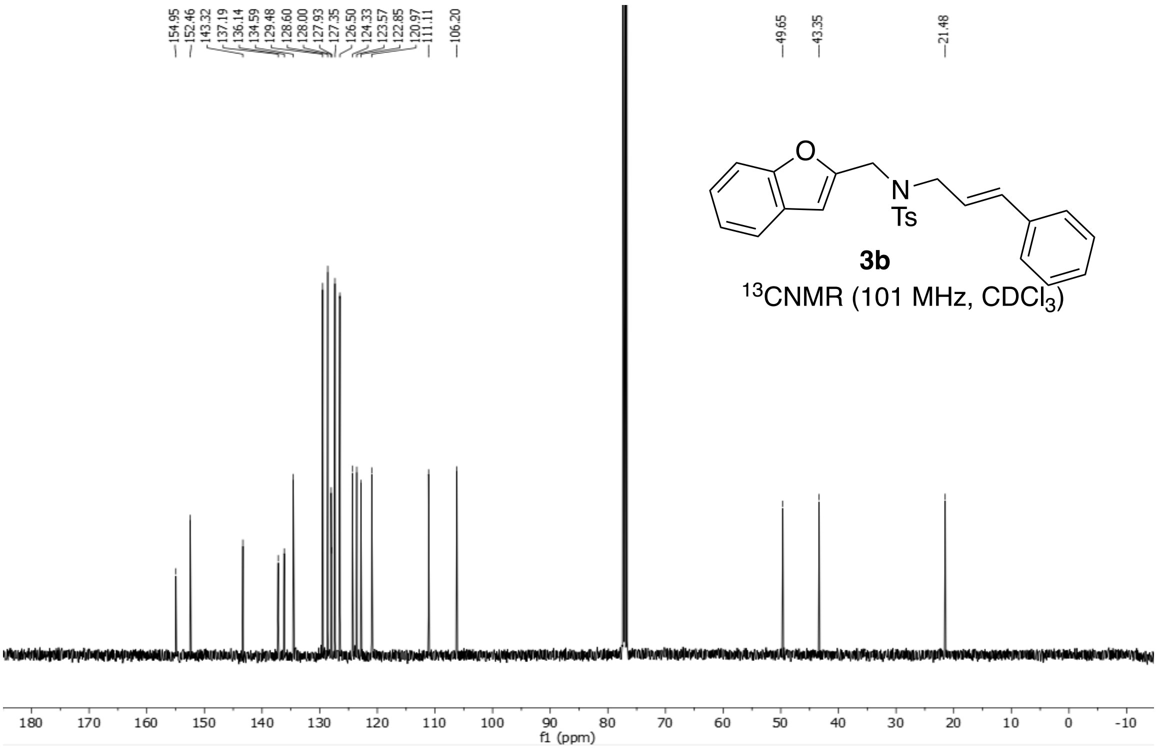
154.95 152.46 143.32 137.19 136.14 134.59 129.48 128.60 128.00 127.93 127.35 126.50 124.33 123.57 122.85 120.97 111.11 106.20
49.65 43.35 21.48
O
N
Ts
3b
^{13}CNMR (101 MHz, $CDCl_3$)
180 170 160 150 140 130 120 110 100 90 80 70 60 50 40 30 20 10 0 -10
f1 (ppm)

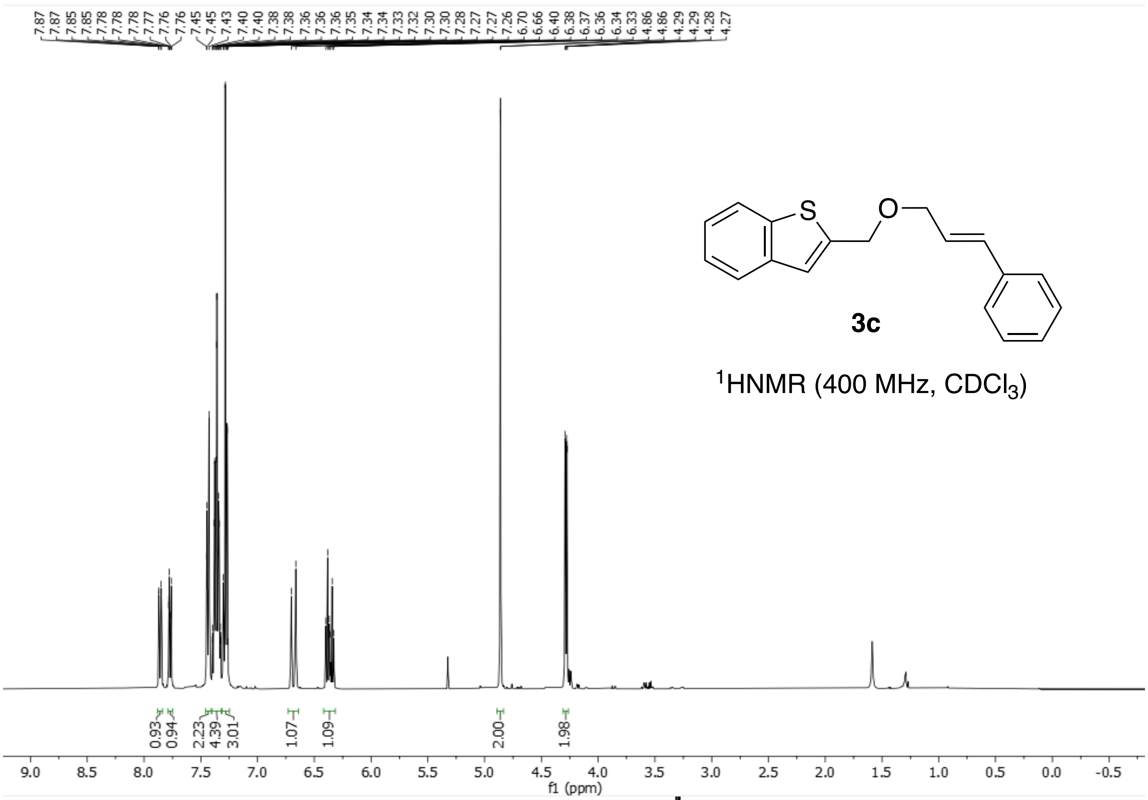
7.87 7.87 7.85 7.85 7.78 7.78 7.78 7.77 7.76 7.76 7.45 7.45 7.43 7.40 7.40 7.38 7.38 7.36 7.36 7.36 7.35 7.34 7.34 7.33 7.32 7.30 7.30 7.28 7.27 7.27 7.26 6.70 6.66 6.40 6.38 6.37 6.36 6.34 6.33 4.86 4.86 4.29 4.29 4.28 4.27
S
O
3c
1HNMR (400 MHz, CDCl3)
0.93 0.94 2.23 4.39 3.01 1.07 1.09 2.00 1.98
9.0 8.5 8.0 7.5 7.0 6.5 6.0 5.5 5.0 4.5 4.0 3.5 3.0 2.5 2.0 1.5 1.0 0.5 0.0 -0.5
f1 (ppm)

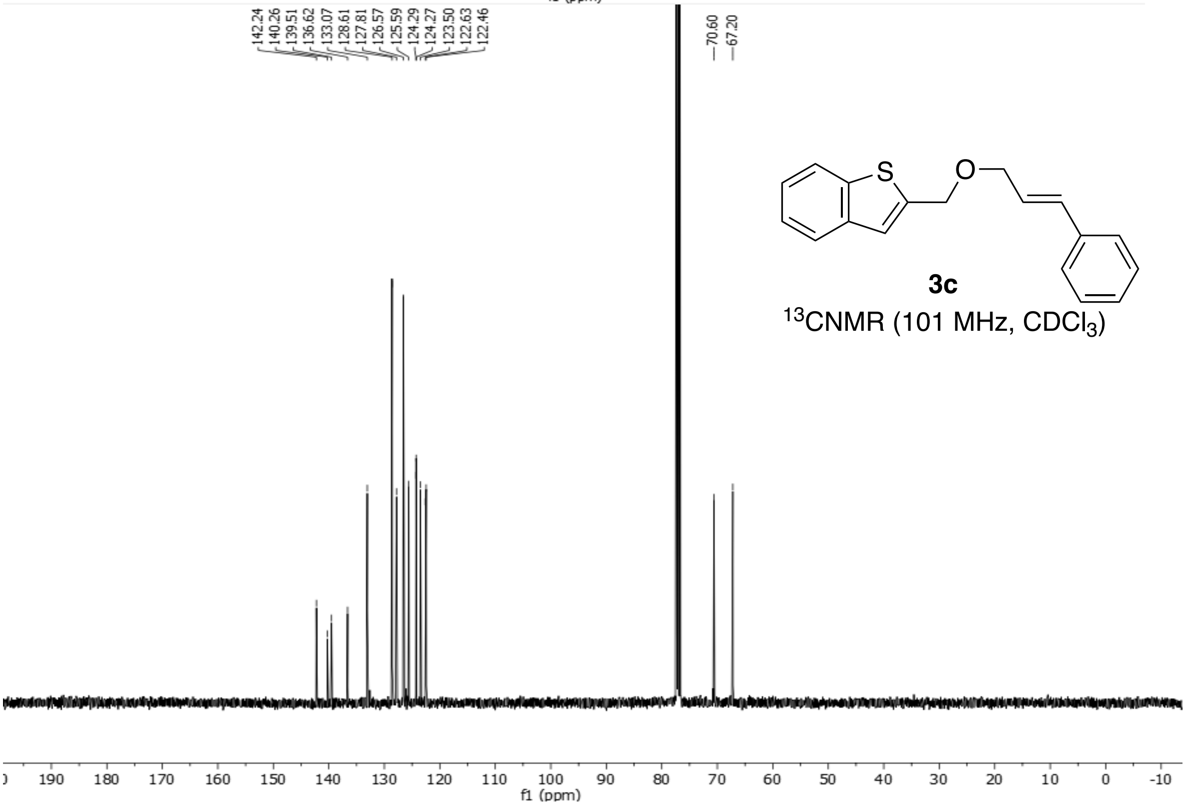
142.24 140.26 139.51 136.62 133.07 128.61 127.81 126.57 125.59 124.29 124.27 123.50 122.63 122.46
70.60 67.20
S
O
3c
13CNMR (101 MHz, CDCl3)
190 180 170 160 150 140 130 120 110 100 90 80 70 60 50 40 30 20 10 0 -10
f1 (ppm)

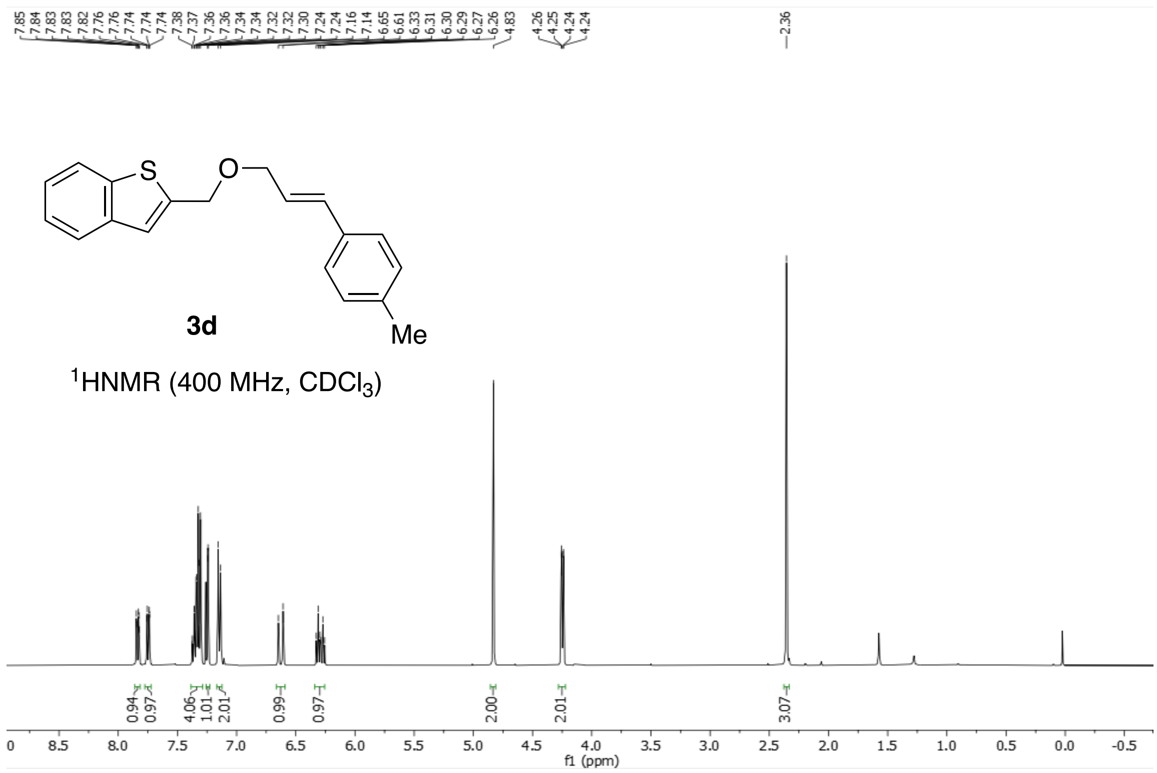
7.85
7.84
7.83
7.83
7.82
7.76
7.76
7.74
7.74
7.74
7.38
7.37
7.36
7.36
7.34
7.34
7.32
7.32
7.30
7.24
7.24
7.16
7.14
6.65
6.61
6.33
6.31
6.30
6.29
6.27
6.26
4.83
4.26
4.25
4.24
4.24
2.36
S
O
3d
Me
1HNMR (400 MHz, CDCl3)
0.94
0.97
4.06
1.01
2.01
0.99
0.97
2.00
2.01
3.07
f1 (ppm)

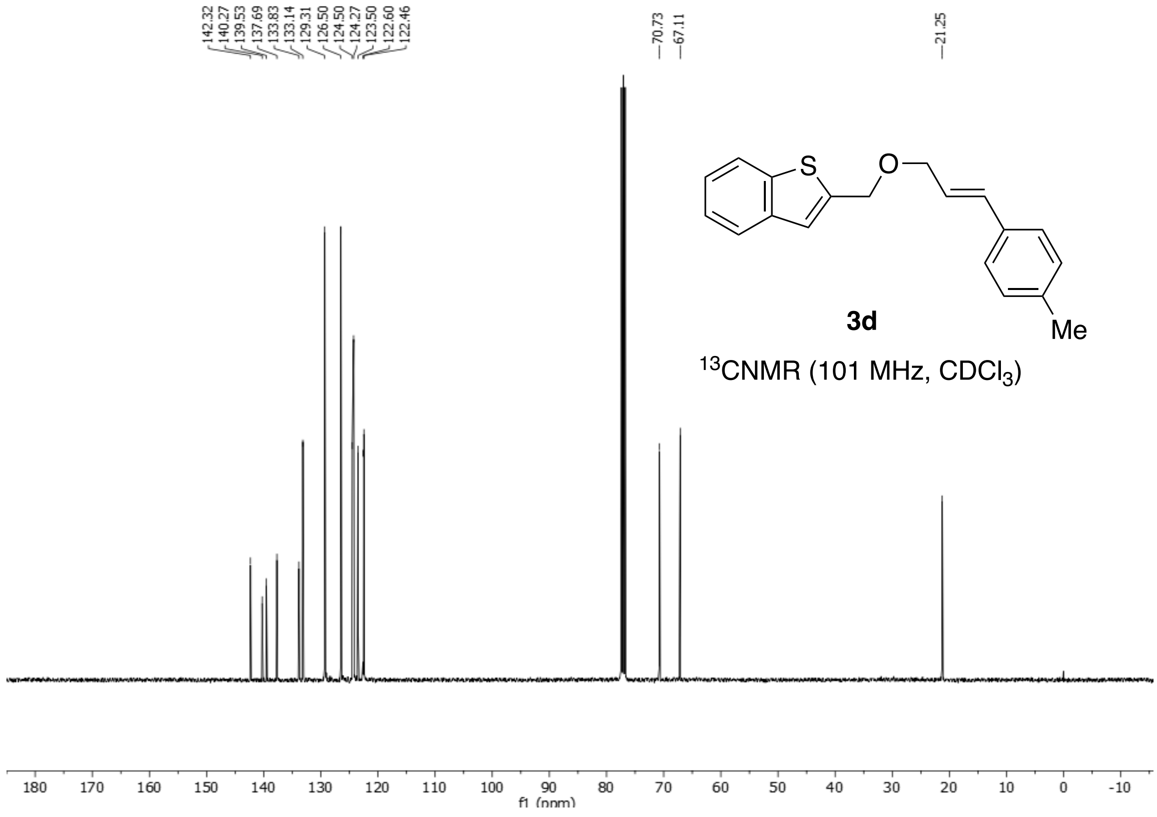
142.32
140.27
139.53
137.69
133.83
133.14
129.31
126.50
124.50
124.27
123.50
122.60
122.46
70.73
67.11
21.25
S
O
3d
Me
13CNMR (101 MHz, CDCl3)
f1 (ppm)

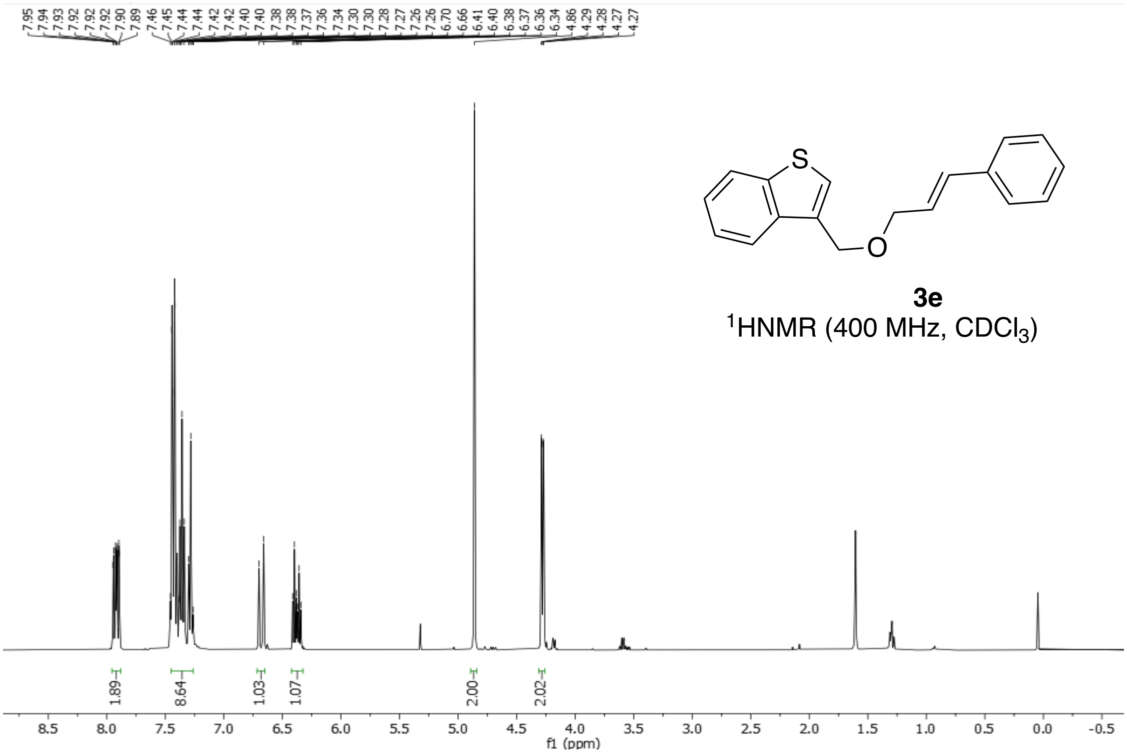

3e

^{1}HNMR (400 MHz, $CDCl_3$)

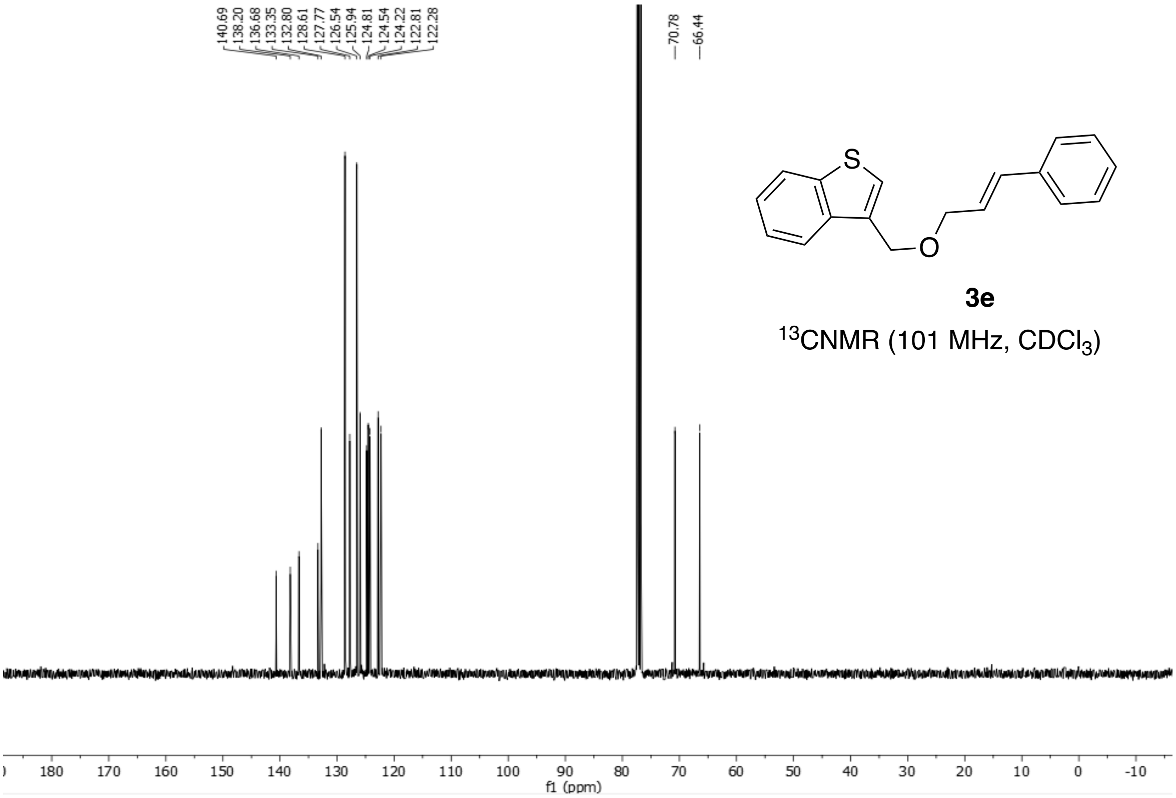

3e

^{13}CNMR (101 MHz, $CDCl_3$)

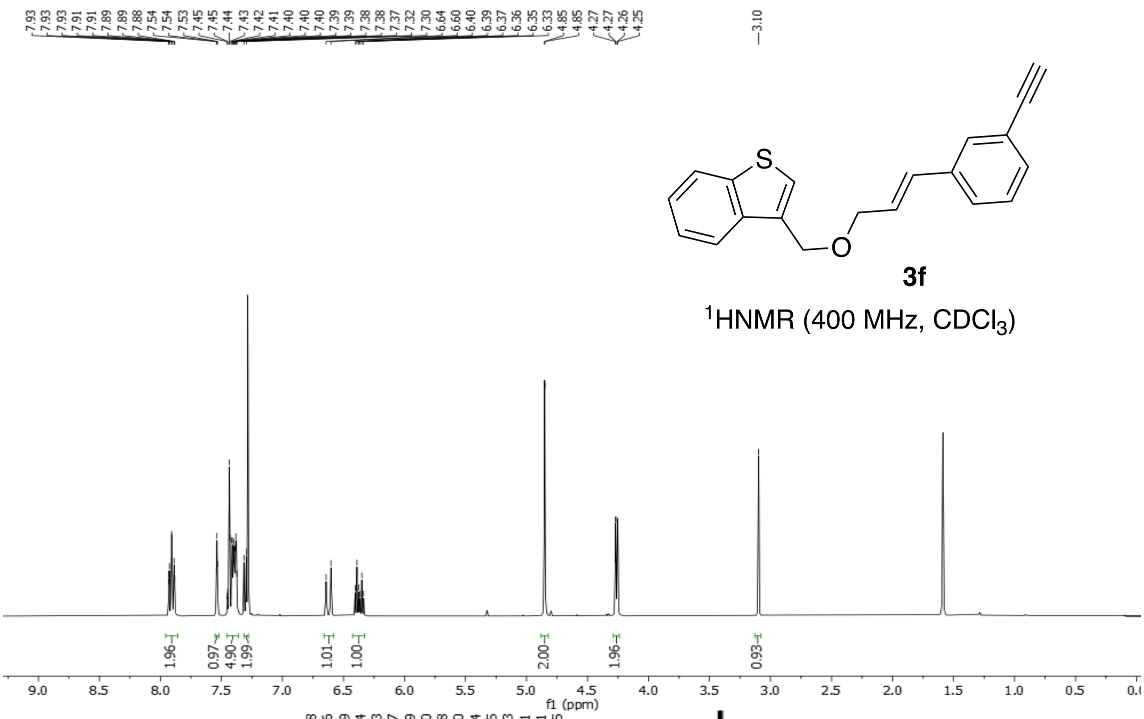

3f

^{1}HNMR (400 MHz, $CDCl_3$)

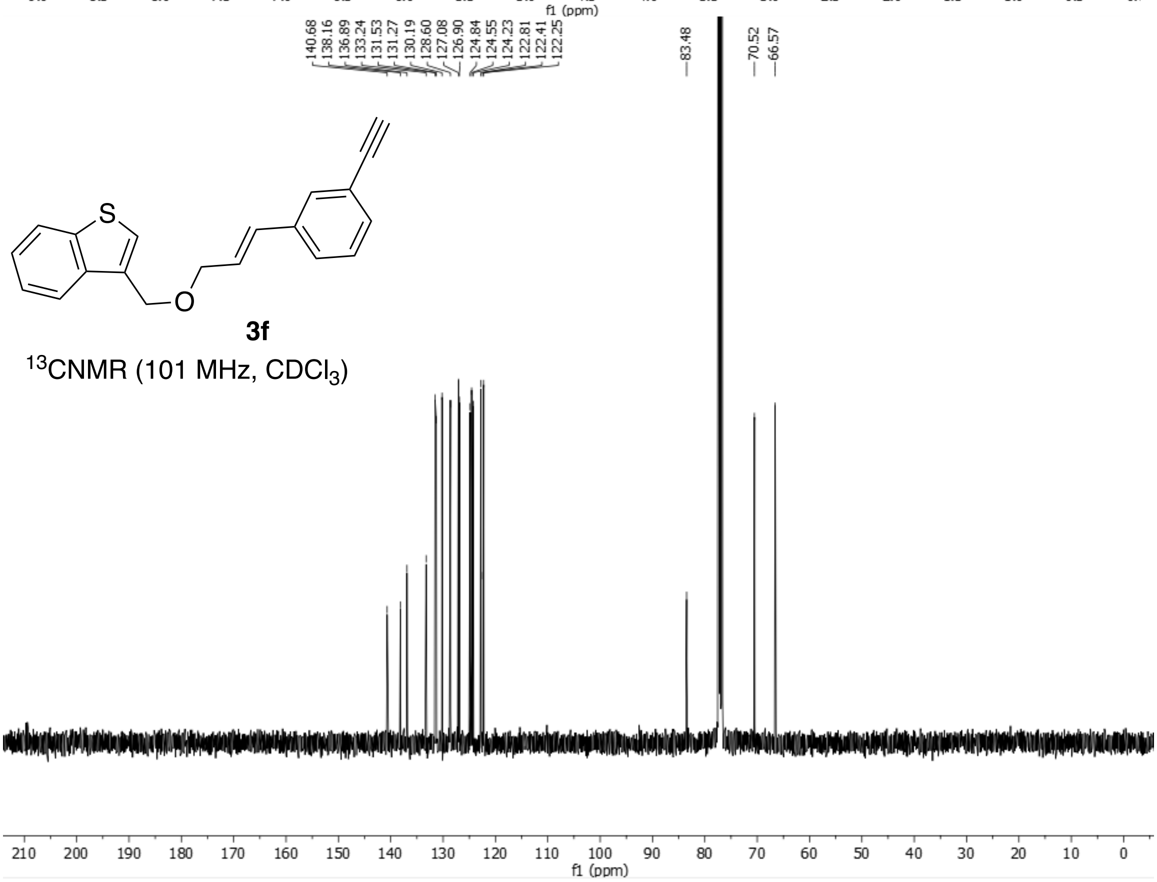

3f

^{13}CNMR (101 MHz, $CDCl_3$)

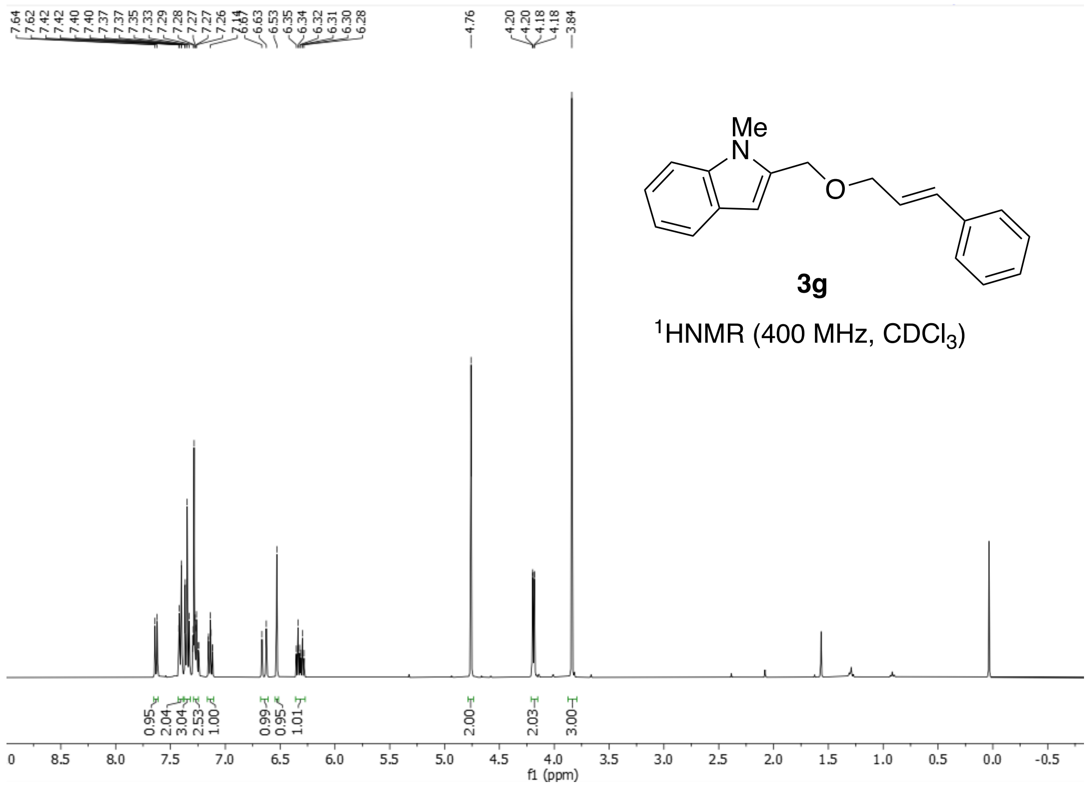

7.64
7.62
7.42
7.42
7.40
7.40
7.37
7.37
7.35
7.33
7.29
7.28
7.27
7.27
7.26
7.14
6.67
6.63
6.53
6.35
6.34
6.32
6.31
6.30
6.28
4.76
4.20
4.20
4.18
4.18
3.84
Me
N
O
3g
^{1}HNMR (400 MHz, $CDCl_3$)
0.95
2.04
3.04
2.53
1.00
0.99
0.95
1.01
2.00
2.03
3.00
f1 (ppm)

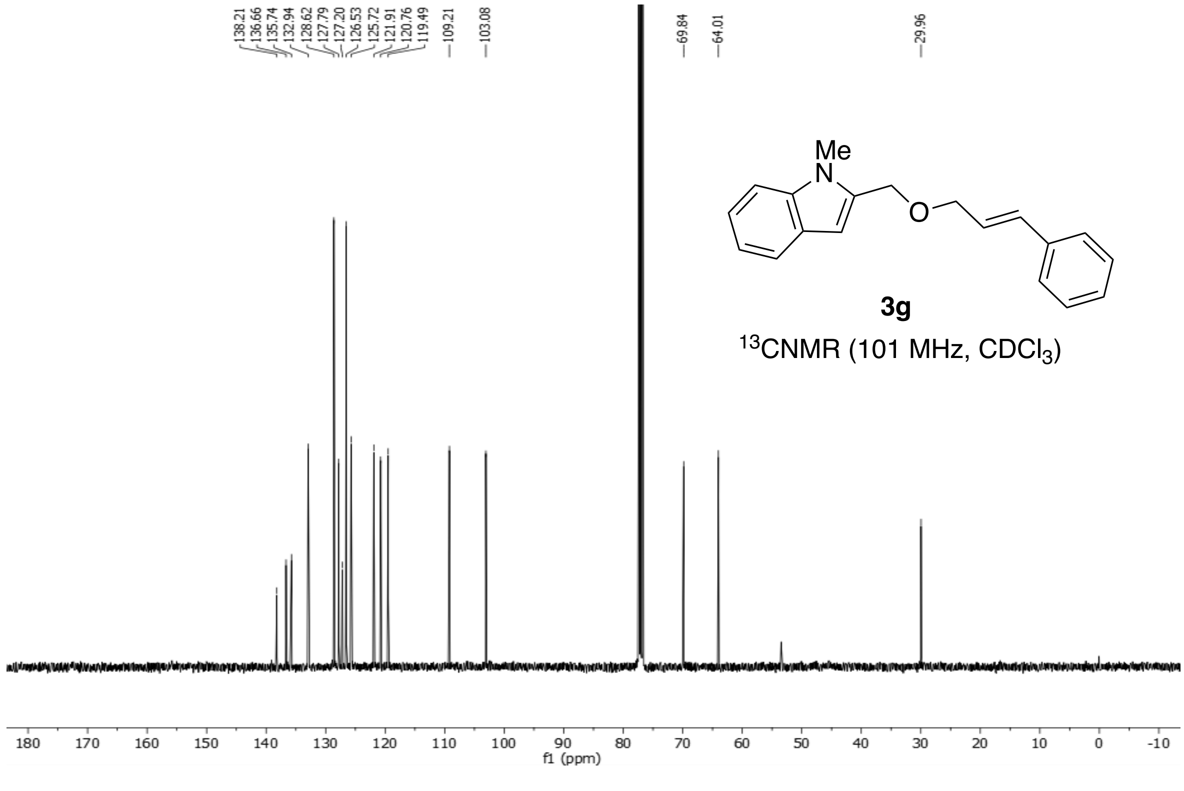

138.21
136.66
135.74
132.94
128.62
127.79
127.20
126.53
125.72
121.91
120.76
119.49
109.21
103.08
69.84
64.01
29.96
Me
N
O
3g
^{13}CNMR (101 MHz, $CDCl_3$)
f1 (ppm)

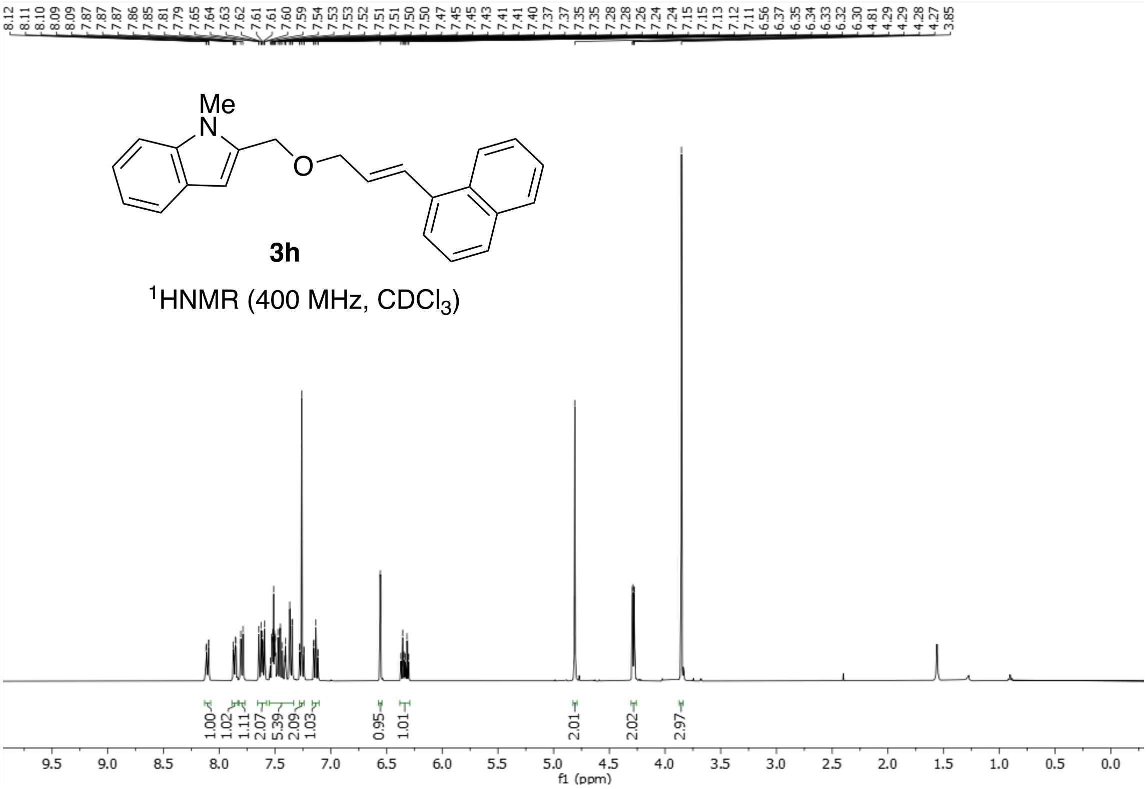
Me
N
O
3h
1HNMR (400 MHz, CDCl3)
f1 (ppm)

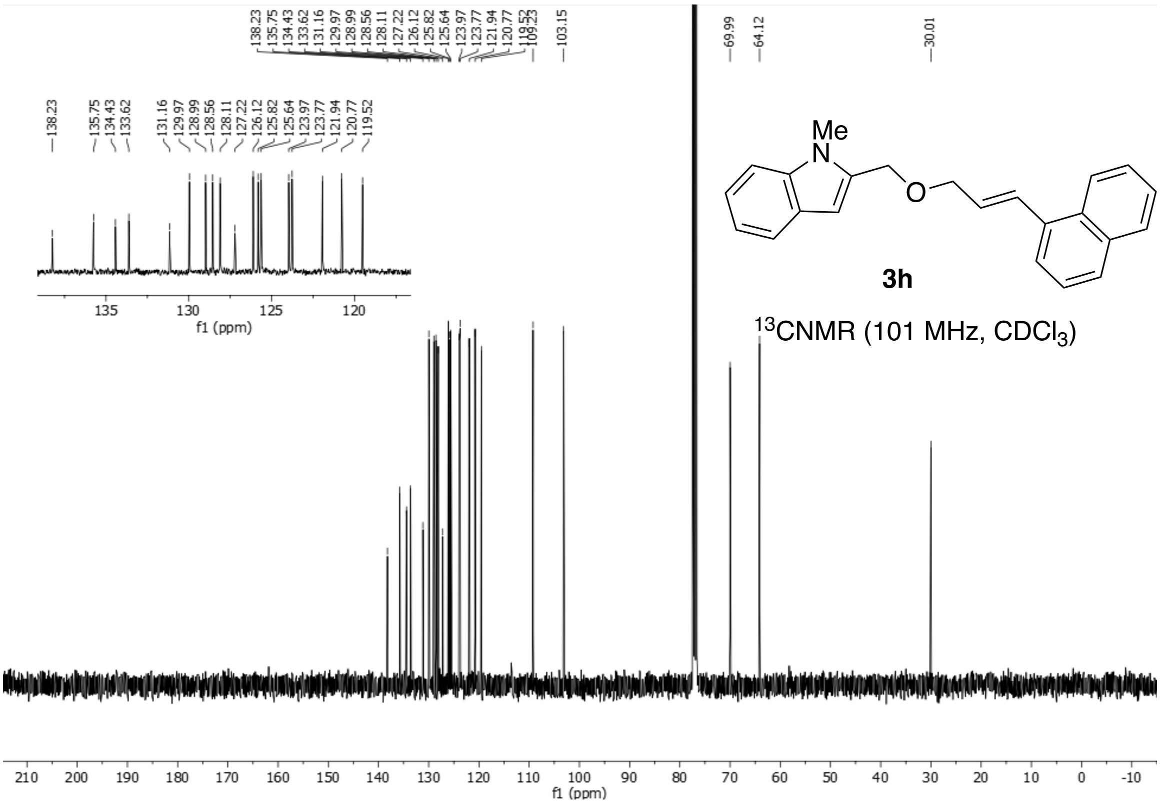
Me
N
O
3h
13CNMR (101 MHz, CDCl3)
f1 (ppm)

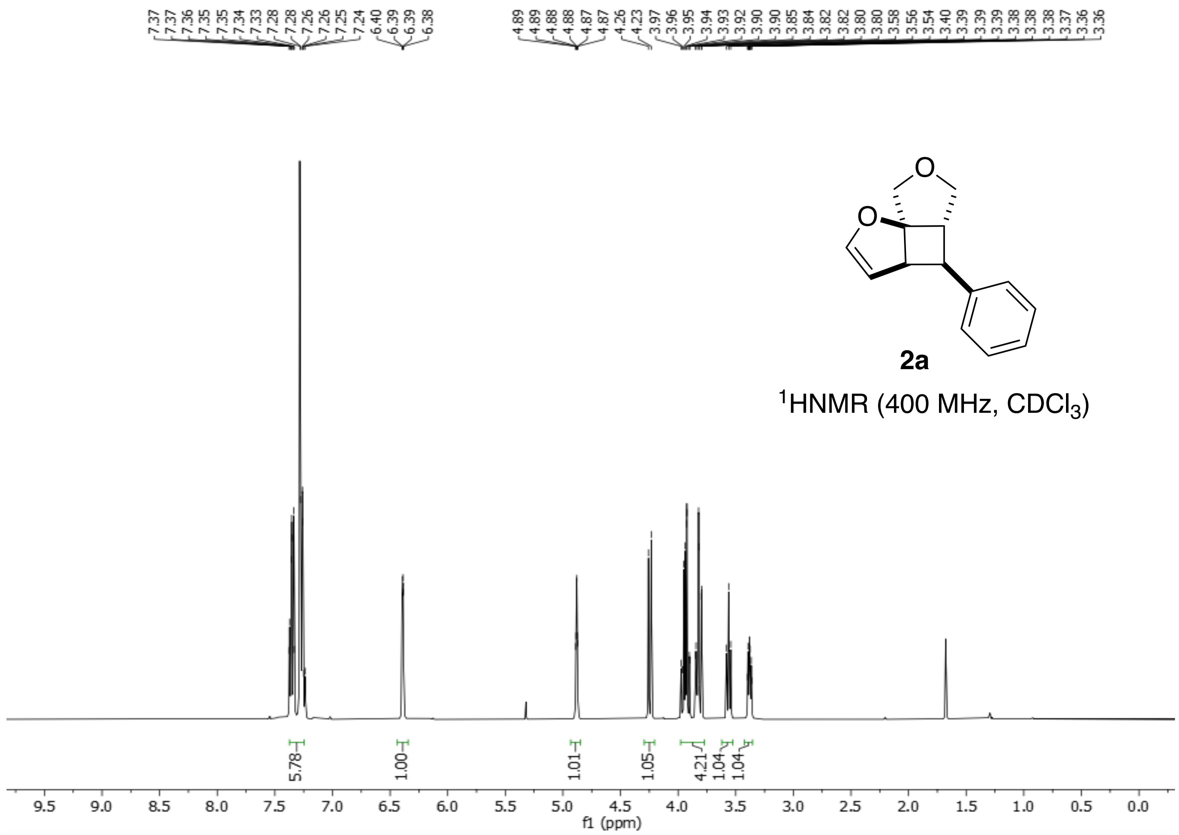

2a

^{1}HNMR (400 MHz, $CDCl_3$)

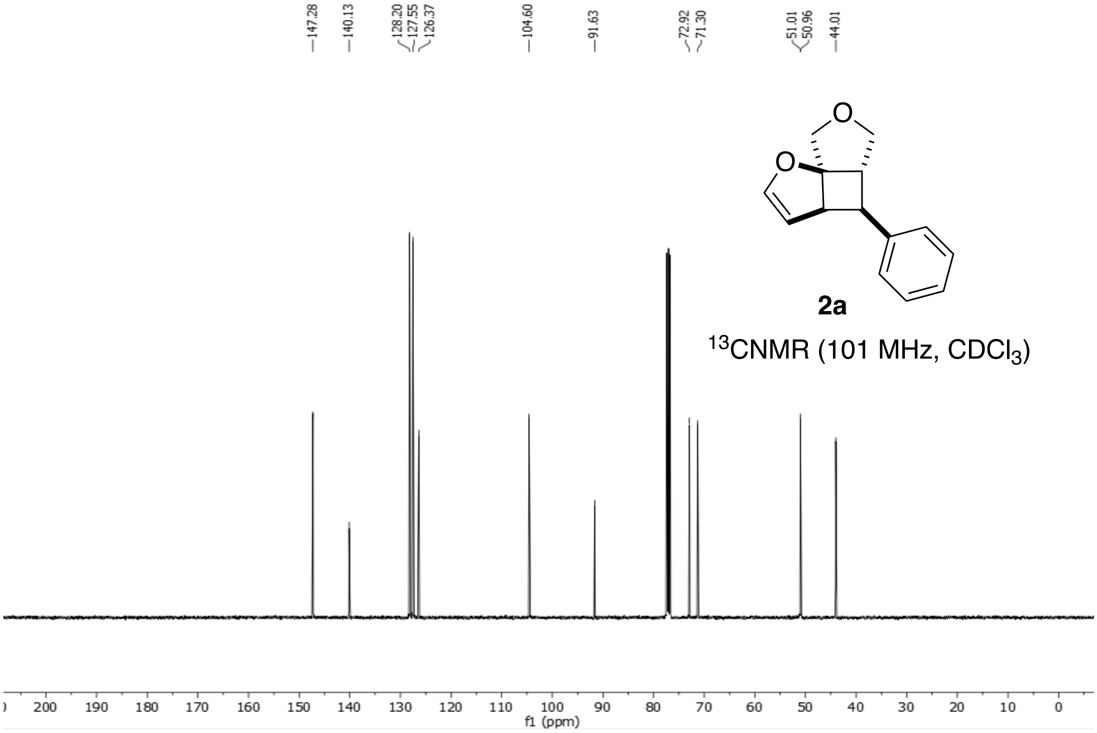

2a

^{13}CNMR (101 MHz, $CDCl_3$)

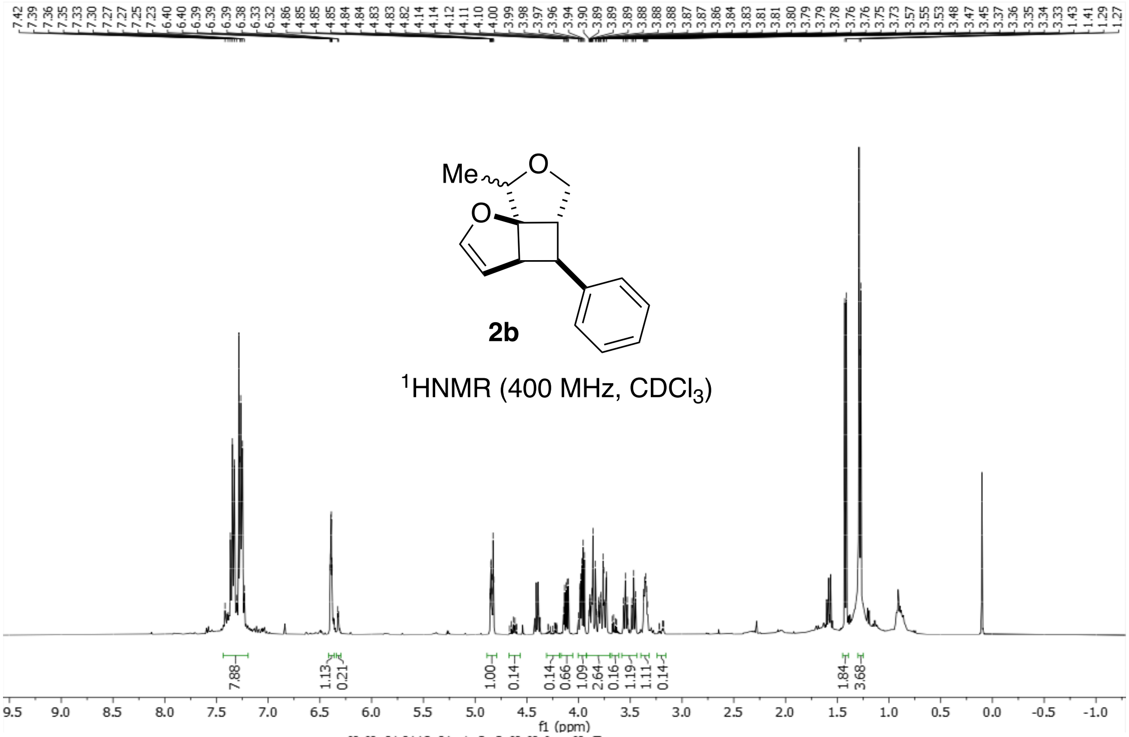
Me
O
O
2b
^{1}HNMR (400 MHz, CDCl$_3$)
7.88
1.13
0.21
1.00
0.14
0.14
0.66
1.09
2.64
0.16
1.19
1.11
0.14
1.84
3.68
f1 (ppm)

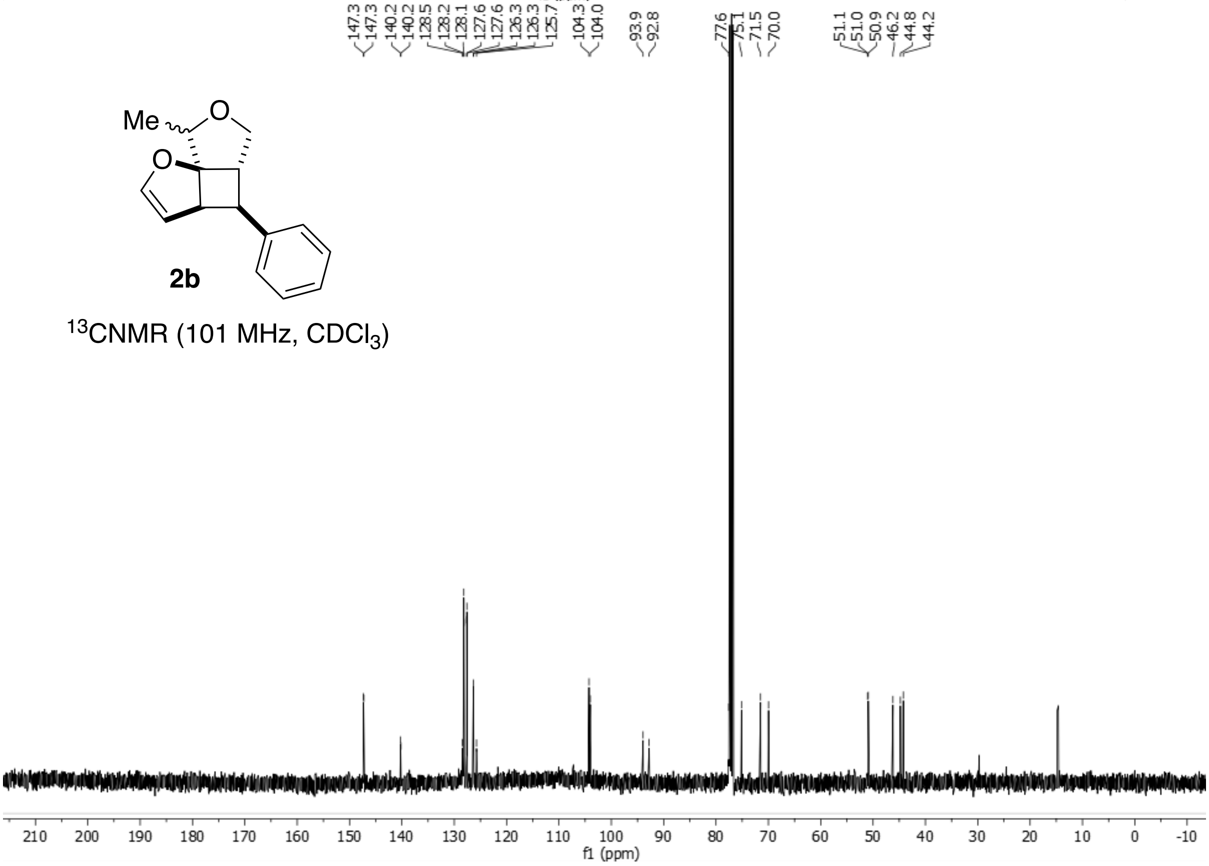
147.3
147.3
140.2
140.2
128.5
128.2
128.1
127.6
127.6
126.3
126.3
125.7
104.3
104.0
93.9
92.8
77.6
75.1
71.5
70.0
51.1
51.0
50.9
46.2
44.8
44.2
Me
O
O
2b
^{13}CNMR (101 MHz, CDCl$_3$)
f1 (ppm)

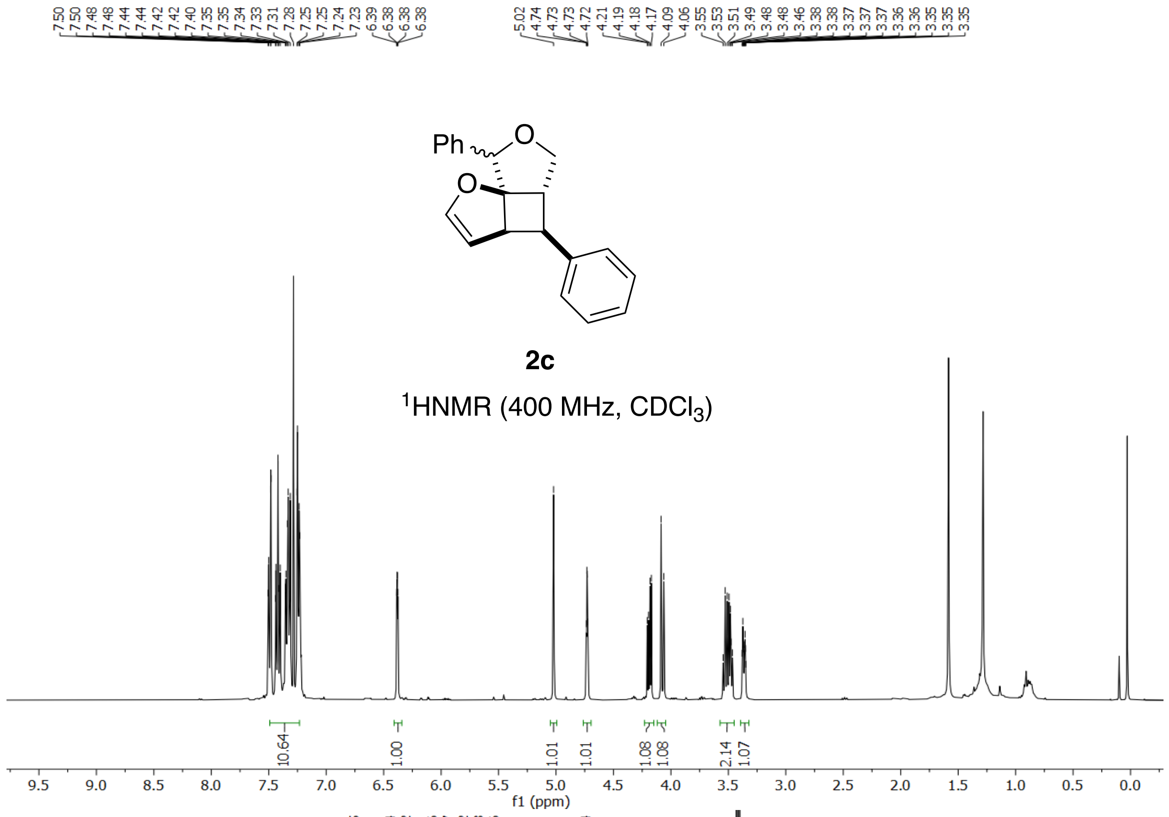
7.50 7.50 7.48 7.44 7.44 7.42 7.42 7.40 7.35 7.35 7.34 7.33 7.31 7.28 7.25 7.25 7.24 7.23 6.39 6.38 6.38 6.38
5.02 4.74 4.73 4.73 4.72 4.21 4.19 4.18 4.17 4.09 4.06 3.55 3.53 3.51 3.49 3.48 3.48 3.46 3.38 3.38 3.37 3.37 3.37 3.36 3.36 3.35 3.35 3.35
Ph
O
O
2c
1HNMR (400 MHz, CDCl3)
10.64
1.00
1.01
1.01
1.08
1.08
2.14
1.07
f1 (ppm)

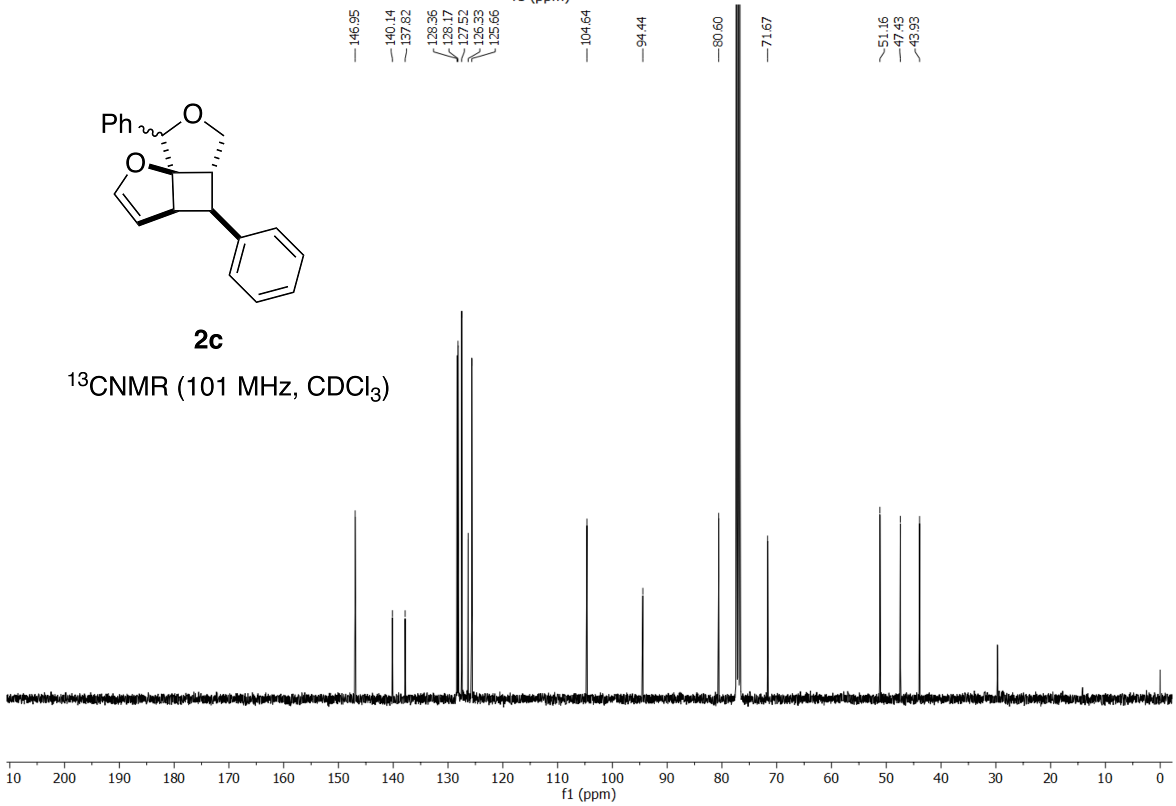
146.95
140.14
137.82
128.36
128.17
127.52
126.33
125.66
104.64
94.44
80.60
71.67
51.16
47.43
43.93
Ph
O
O
2c
13CNMR (101 MHz, CDCl3)
f1 (ppm)

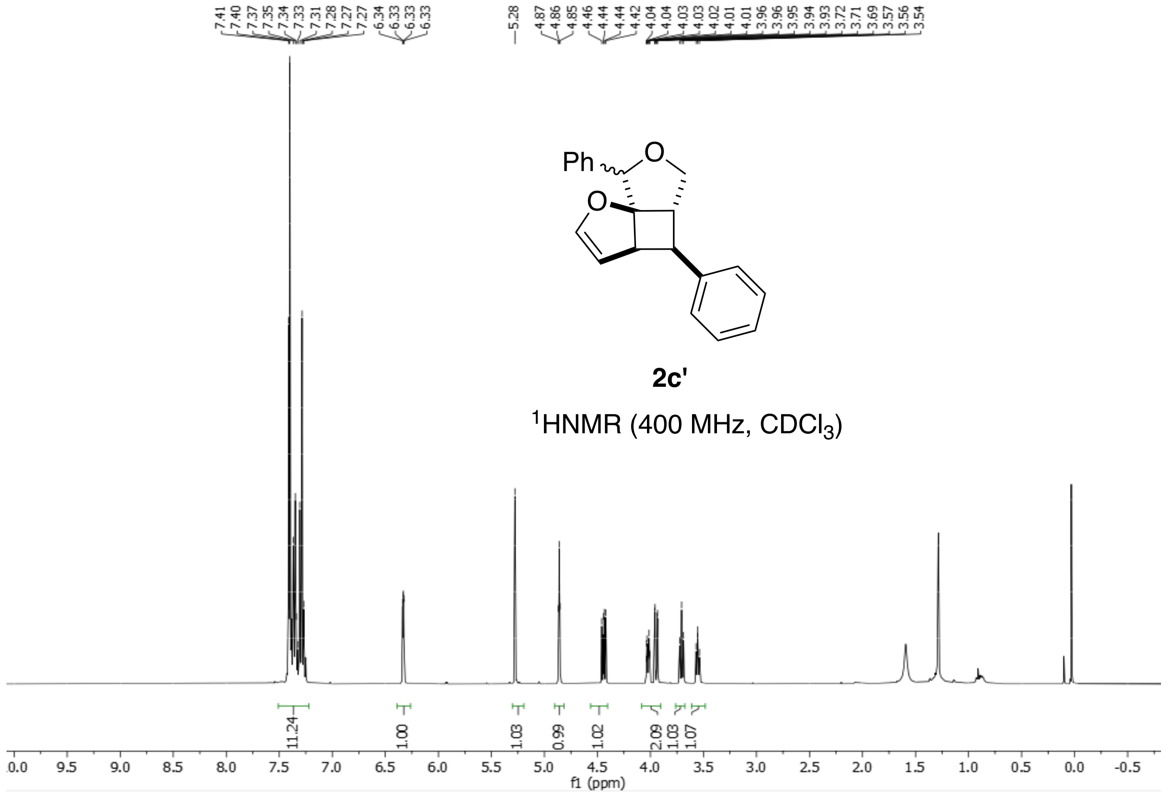
7.41 7.40 7.37 7.35 7.34 7.33 7.31 7.28 7.27 7.27 6.34 6.33 6.33 6.33
5.28 4.87 4.86 4.85 4.46 4.44 4.44 4.42 4.04 4.04 4.03 4.03 4.02 4.01 4.01 3.96 3.96 3.95 3.94 3.93 3.72 3.71 3.69 3.57 3.56 3.54
Ph
O
O
2c'
1HNMR (400 MHz, CDCl3)
11.24 1.00 1.03 0.99 1.02 2.09 1.03 1.07
f1 (ppm)

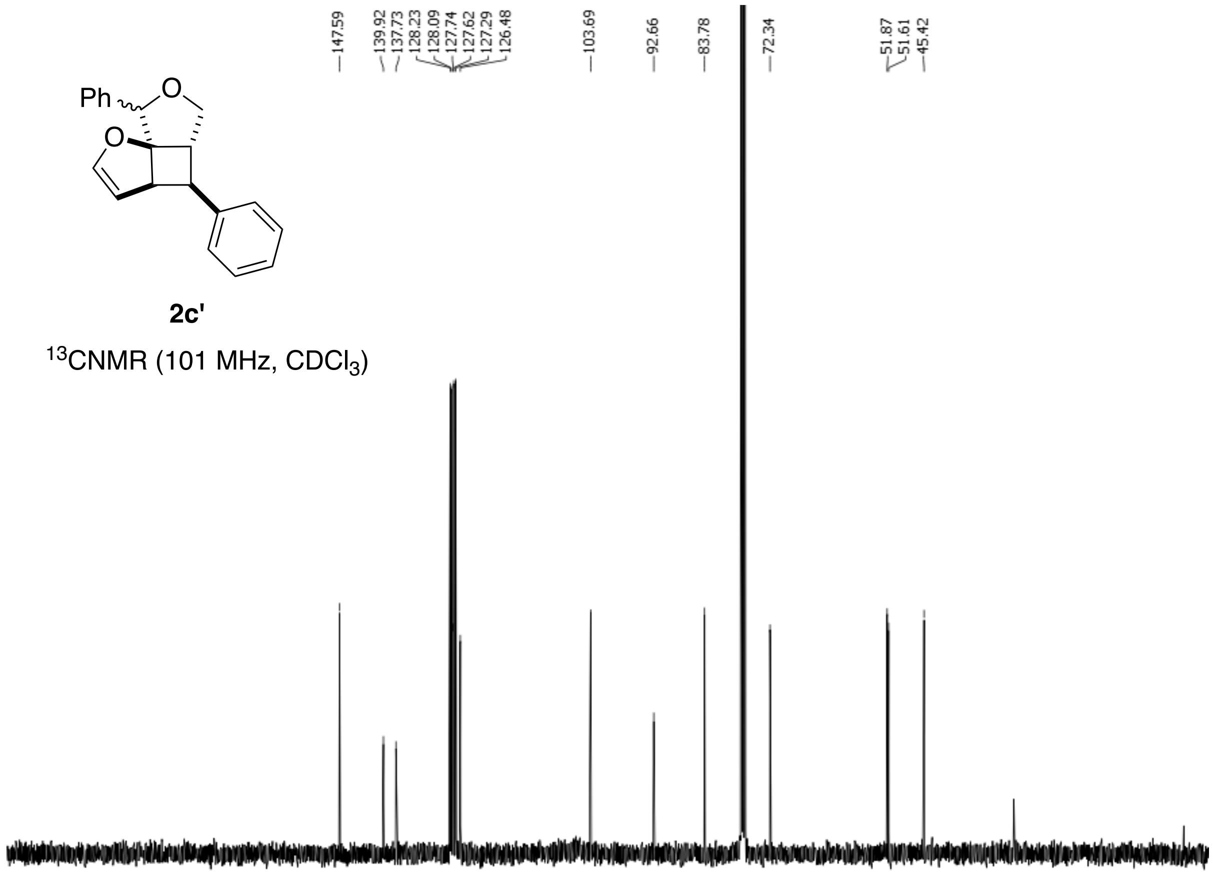
147.59 139.92 137.73 128.23 128.09 127.74 127.62 127.29 126.48 103.69 92.66 83.78 72.34 51.87 51.61 45.42
Ph
O
O
2c'
13CNMR (101 MHz, CDCl3)
f1 (ppm)

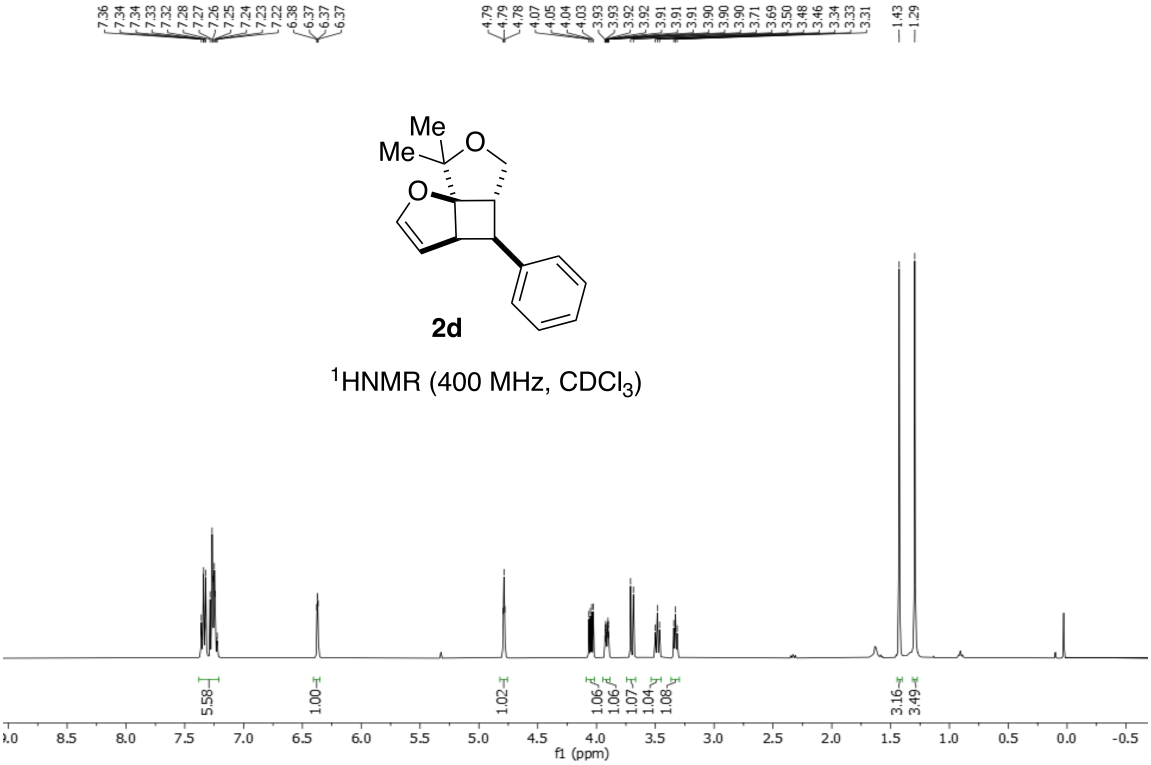

2d

^{1}HNMR (400 MHz, $CDCl_3$)

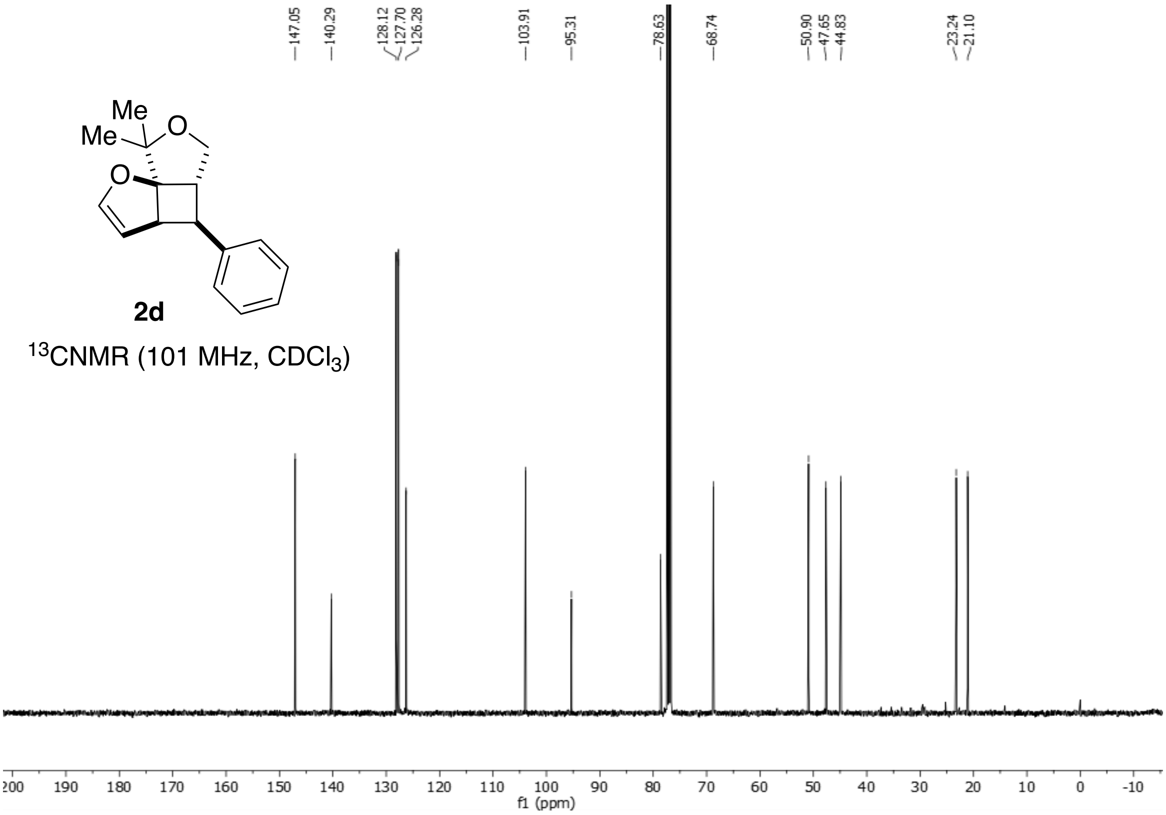

2d

^{13}CNMR (101 MHz, $CDCl_3$)

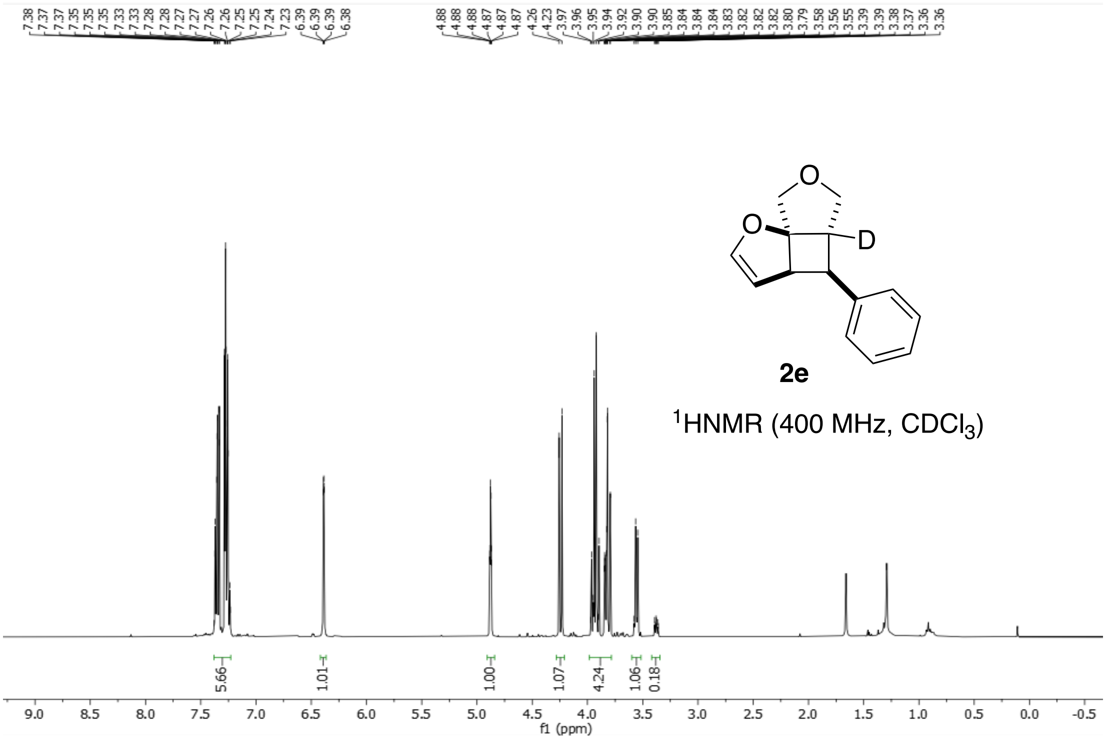

2e
^{1}HNMR (400 MHz, CDCl$_3$)
f1 (ppm)

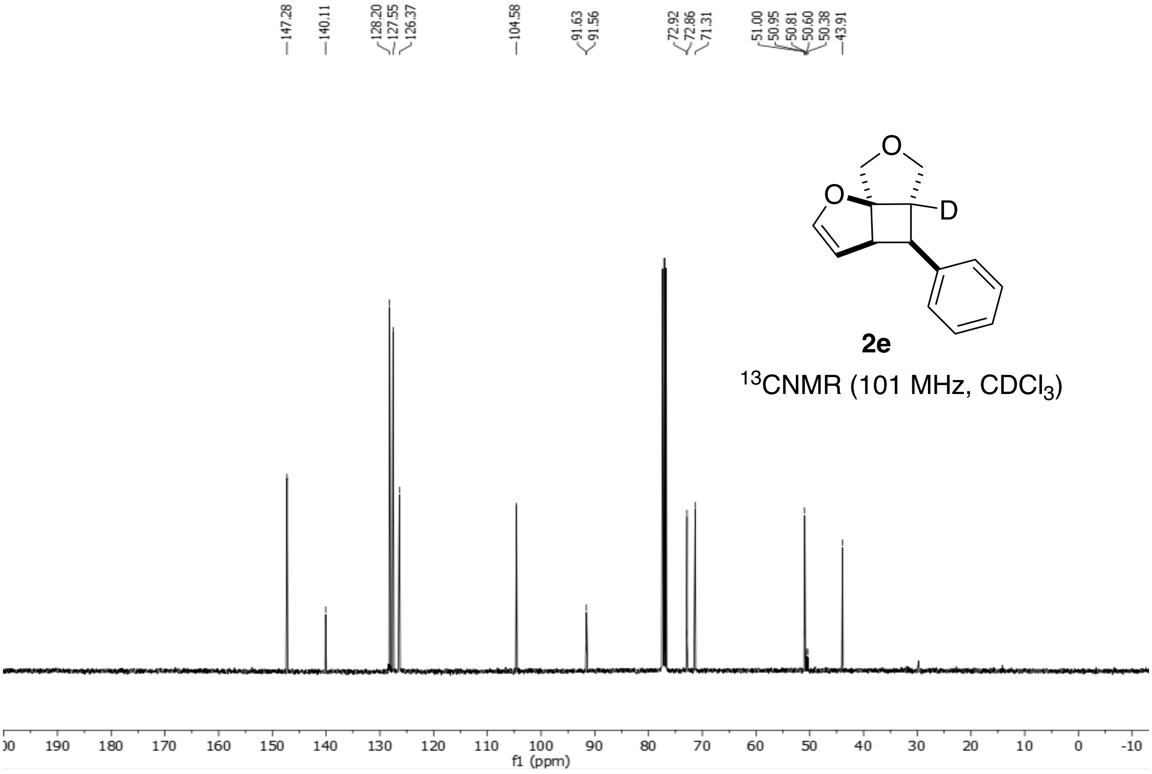

2e
^{13}CNMR (101 MHz, CDCl$_3$)
f1 (ppm)

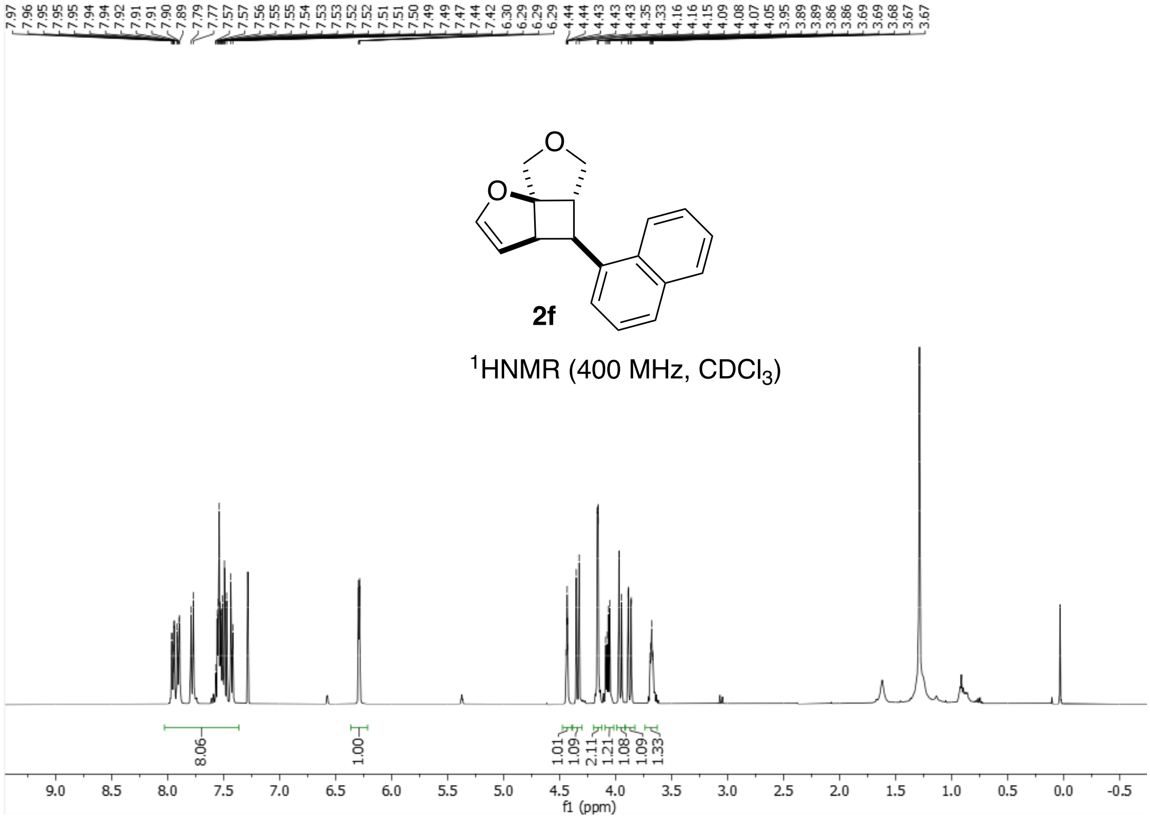
7.97 7.96 7.95 7.95 7.94 7.94 7.92 7.91 7.90 7.89 7.79 7.77 7.57 7.57 7.56 7.55 7.55 7.54 7.53 7.53 7.52 7.52 7.51 7.51 7.50 7.49 7.49 7.47 7.44 7.42 6.30 6.29 6.29 6.29 4.44 4.44 4.43 4.43 4.43 4.35 4.33 4.16 4.15 4.09 4.08 4.07 4.05 3.95 3.89 3.89 3.86 3.86 3.69 3.69 3.68 3.67 3.67
O
O
2f
1HNMR (400 MHz, CDCl3)
8.06
1.00
1.01
1.09
2.11
1.21
1.08
1.09
1.33
f1 (ppm)

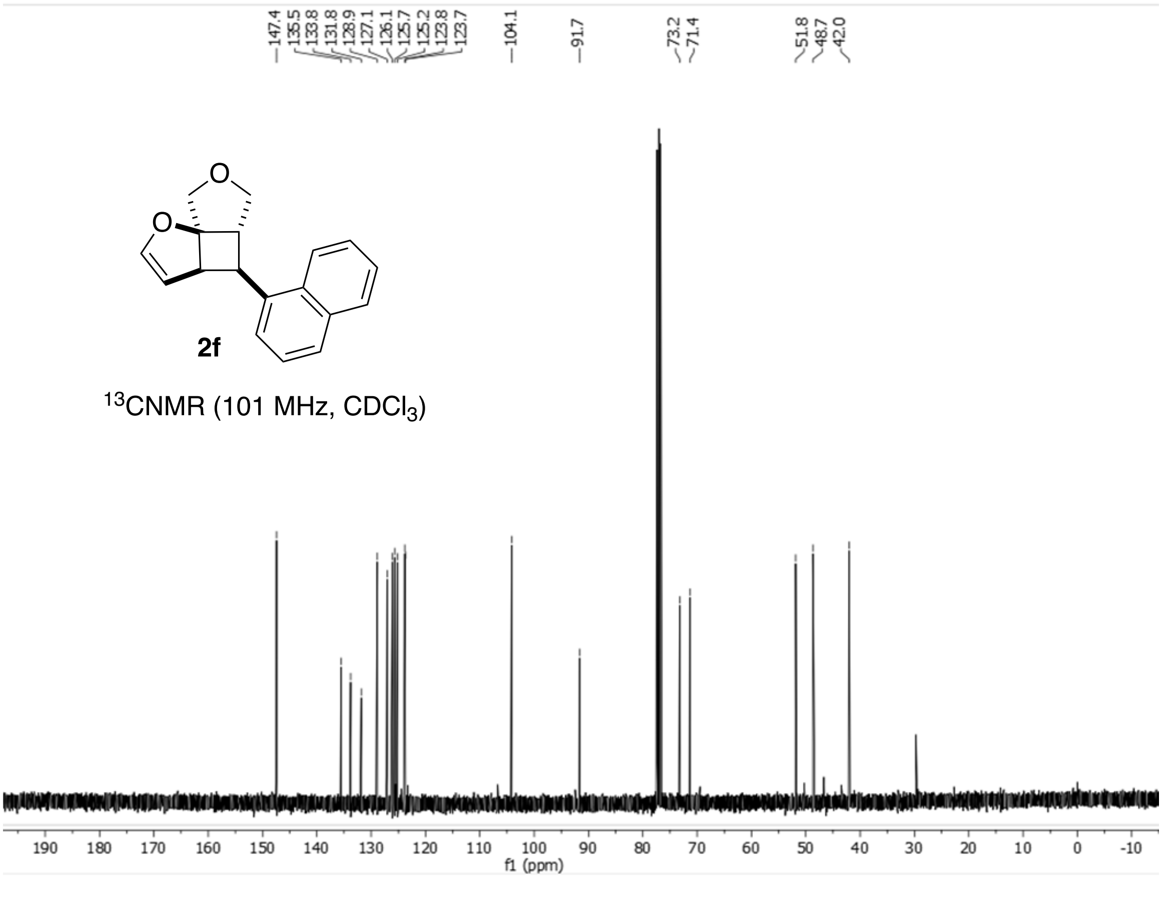
147.4 135.5 133.8 131.8 128.9 127.1 126.1 125.7 125.2 123.8 123.7 104.1 91.7 73.2 71.4 51.8 48.7 42.0
O
O
2f
13CNMR (101 MHz, CDCl3)
f1 (ppm)

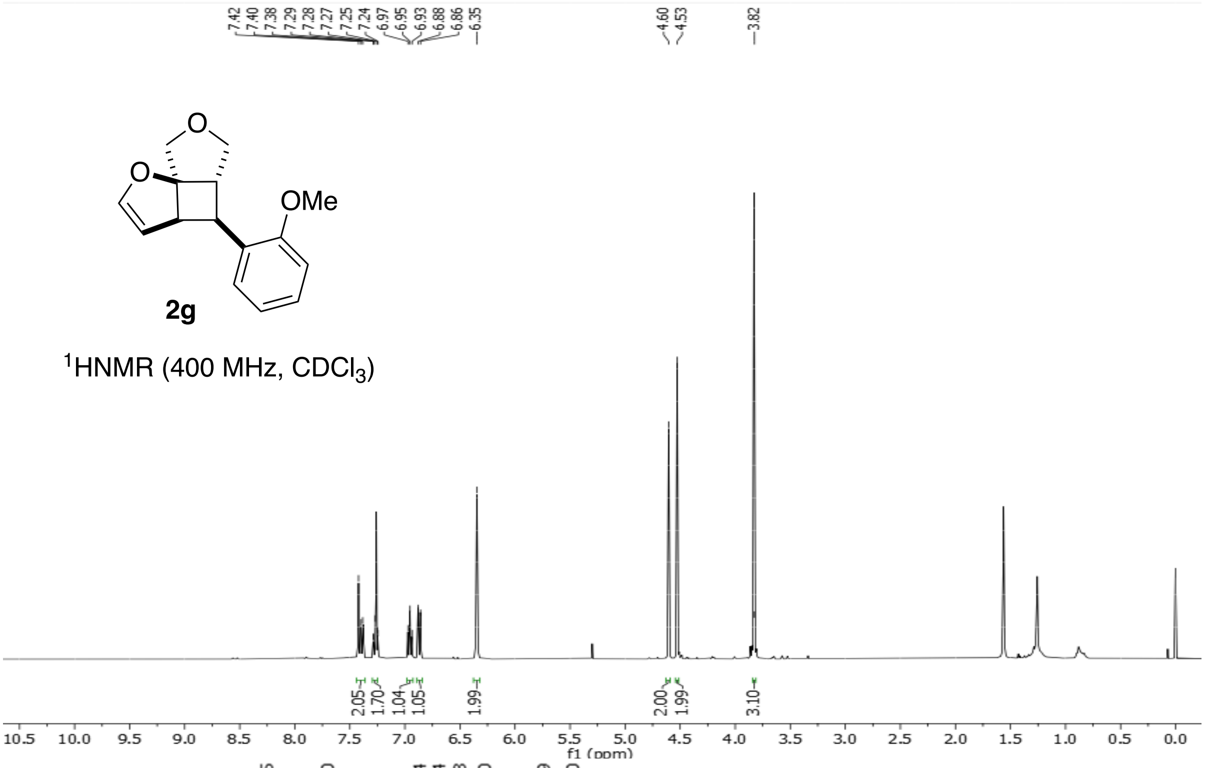

2g

^{1}HNMR (400 MHz, $CDCl_3$)

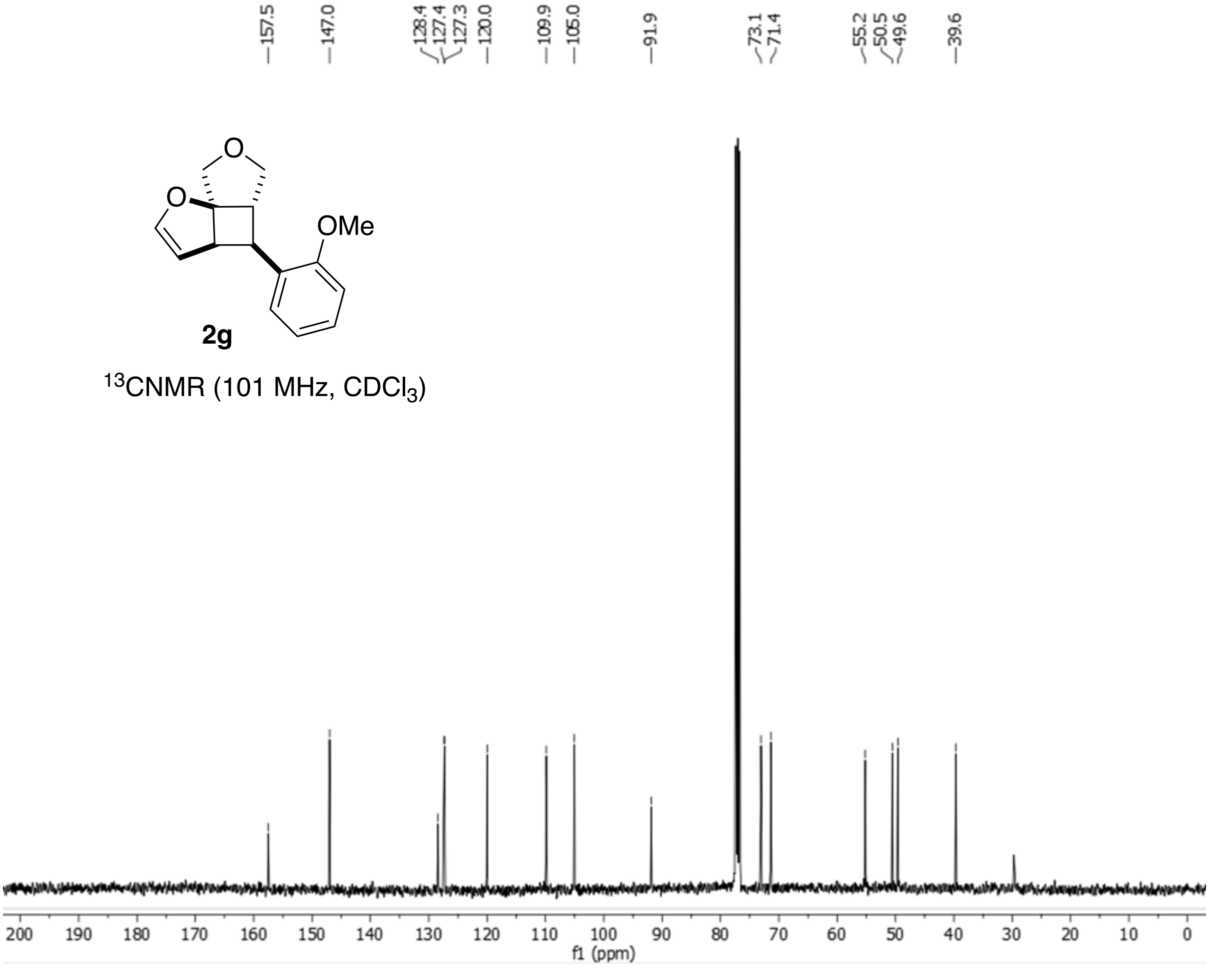

2g

^{13}CNMR (101 MHz, $CDCl_3$)

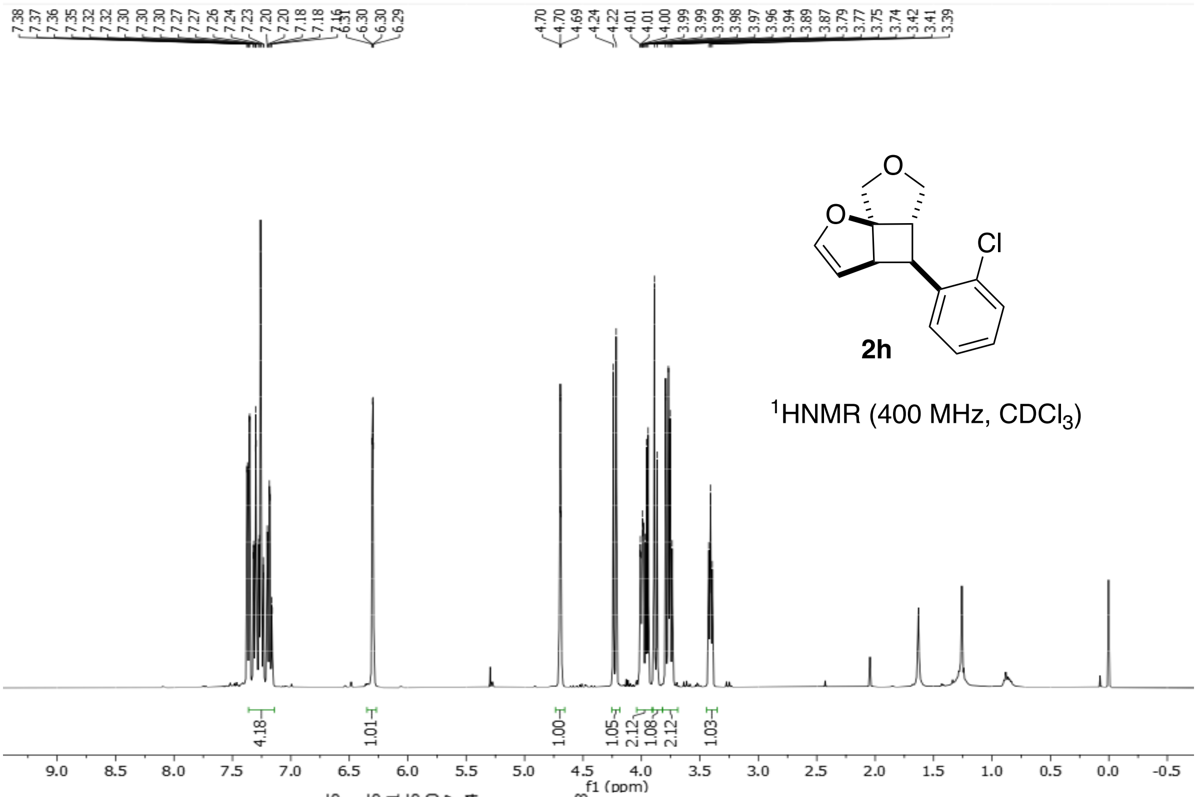

2h
^{1}HNMR (400 MHz, CDCl$_3$)
f1 (ppm)

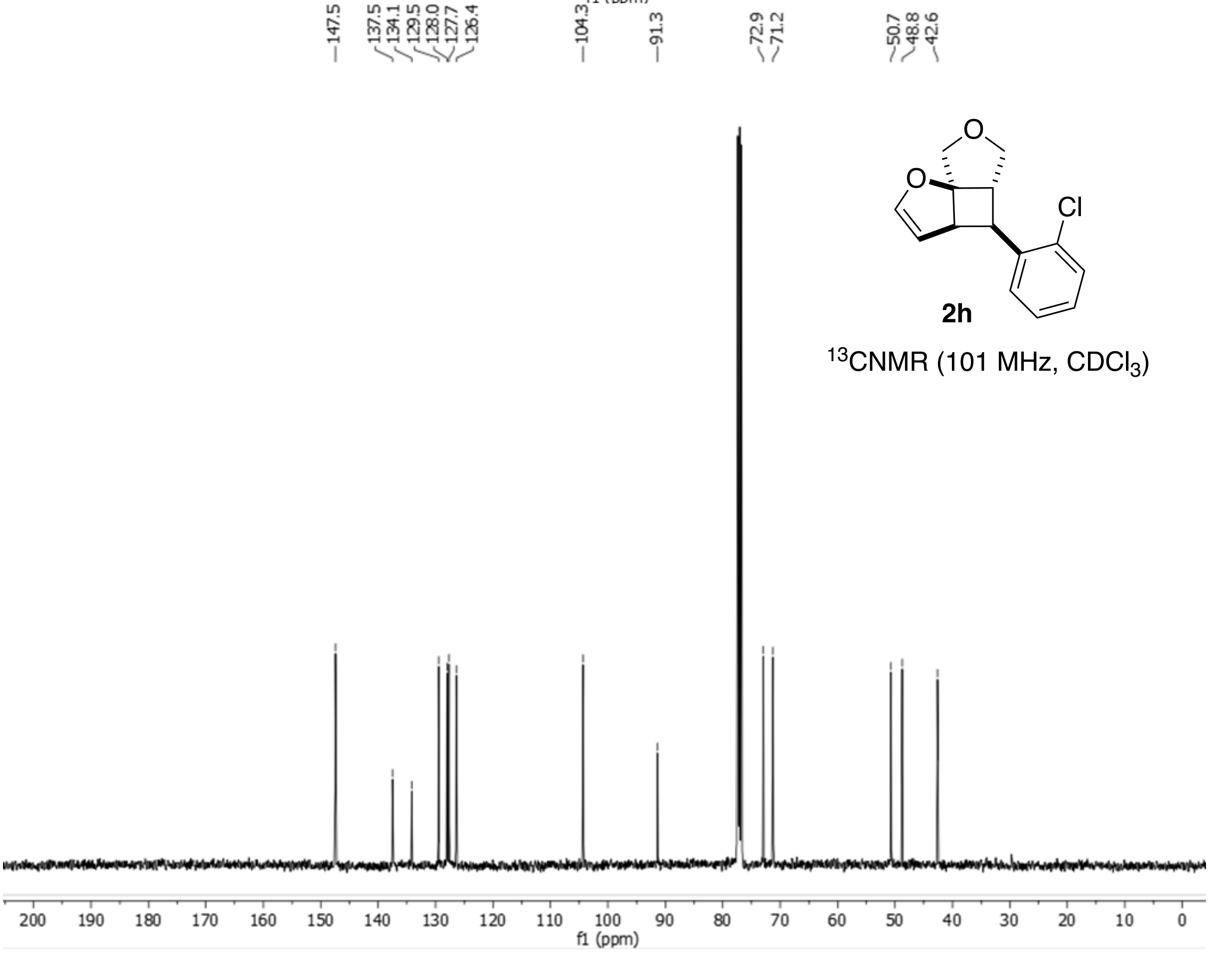

2h
^{13}CNMR (101 MHz, CDCl$_3$)
147.5
137.5
134.1
129.5
128.0
127.7
126.4
104.3
91.3
72.9
71.2
50.7
48.8
42.6
f1 (ppm)

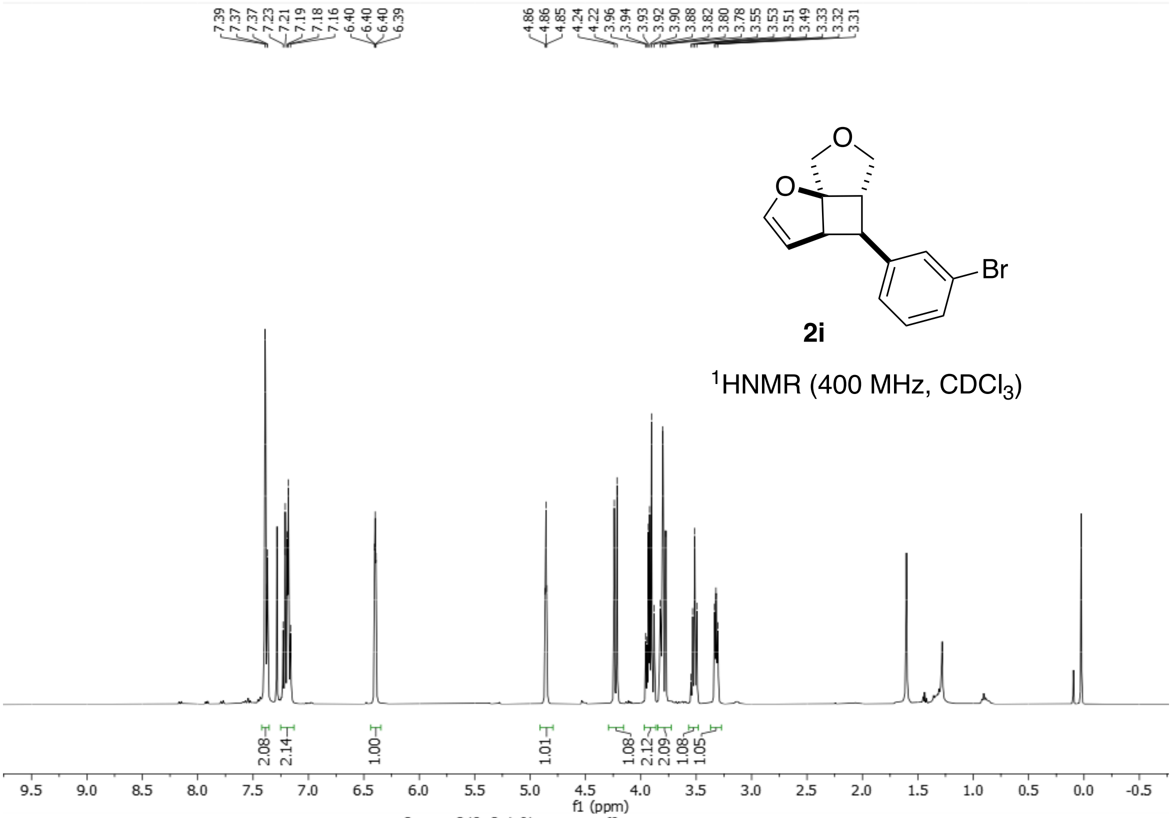

7.39 7.37 7.37 7.23 7.21 7.19 7.18 7.16 6.40 6.40 6.40 6.39
4.86 4.86 4.85 4.24 4.22 3.96 3.94 3.93 3.92 3.90 3.88 3.82 3.80 3.78 3.55 3.53 3.51 3.49 3.33 3.32 3.31
O
O
Br
2i
^{1}HNMR (400 MHz, $CDCl_3$)
2.08 2.14 1.00 1.01 1.08 2.12 2.09 1.08 1.05
f1 (ppm)

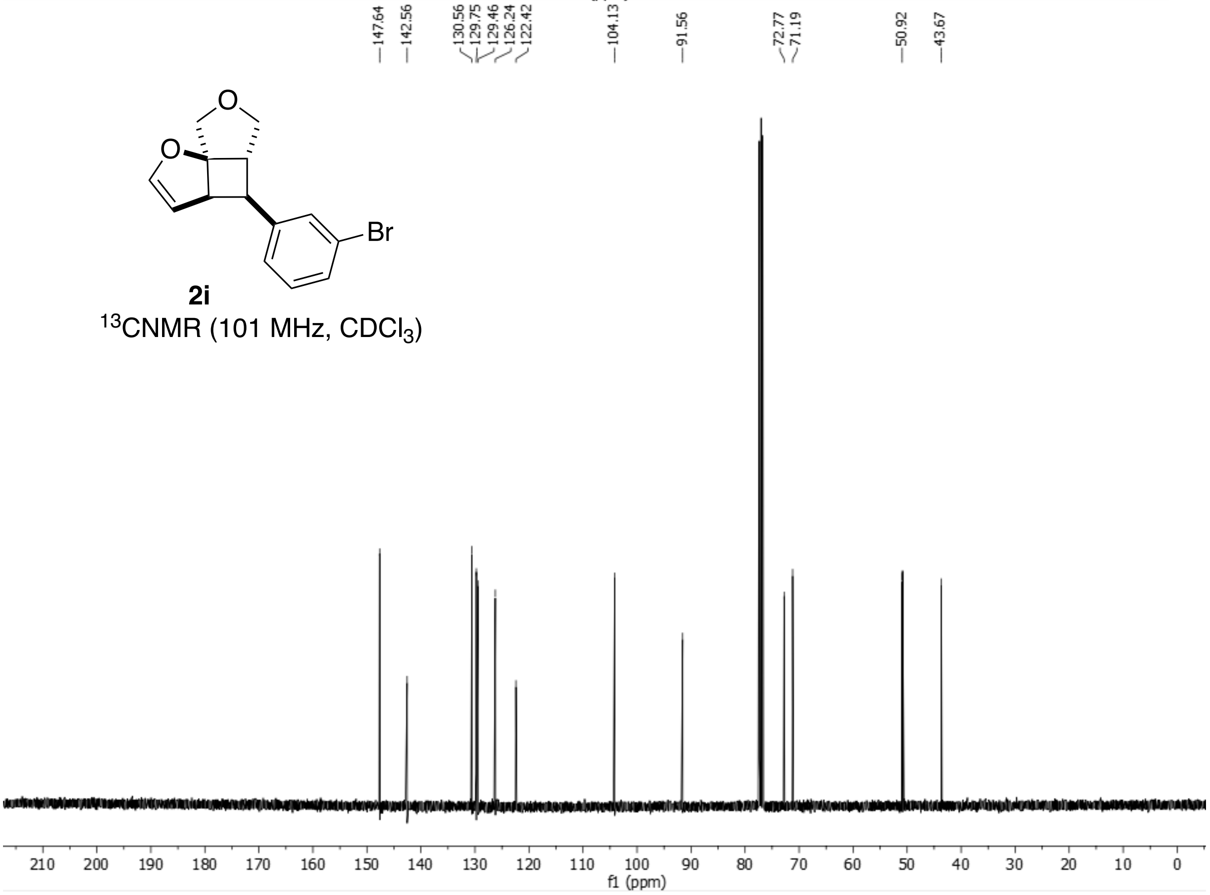

147.64 142.56 130.56 129.75 129.46 126.24 122.42 104.13 91.56 72.77 71.19 50.92 43.67
O
O
Br
2i
^{13}CNMR (101 MHz, $CDCl_3$)
f1 (ppm)

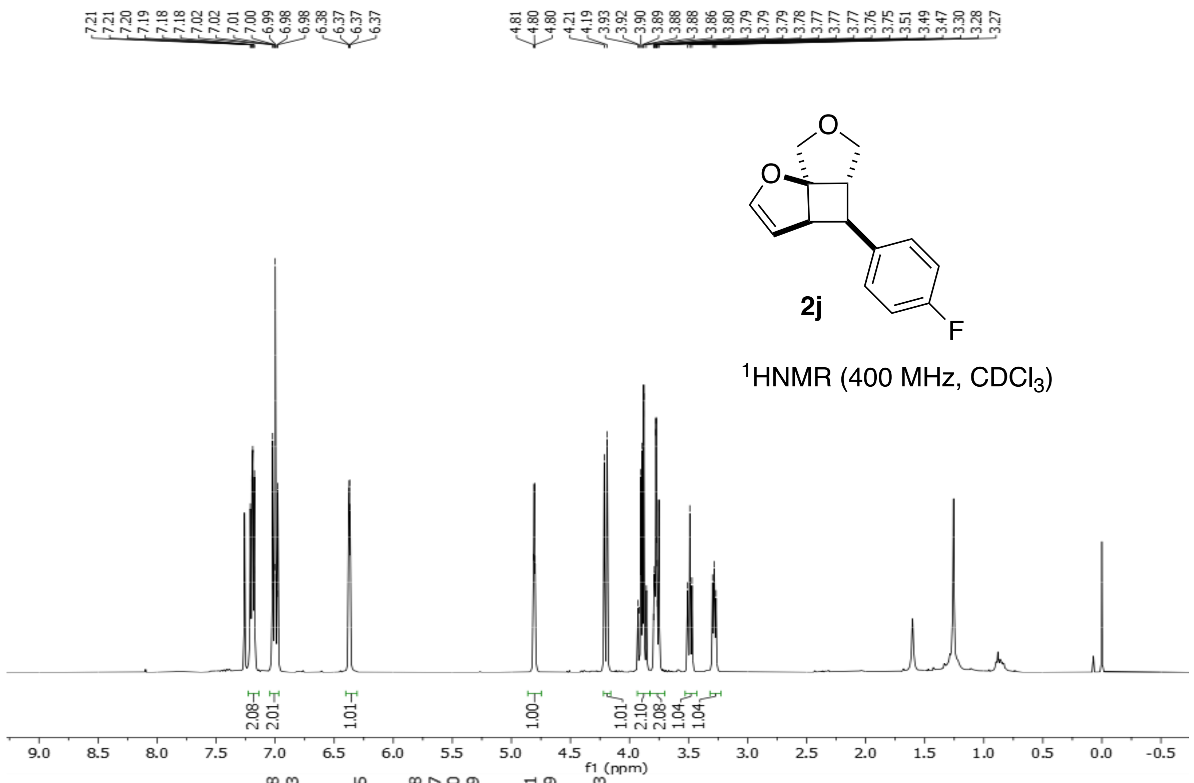

2j
F
^{1}HNMR (400 MHz, CDCl3)

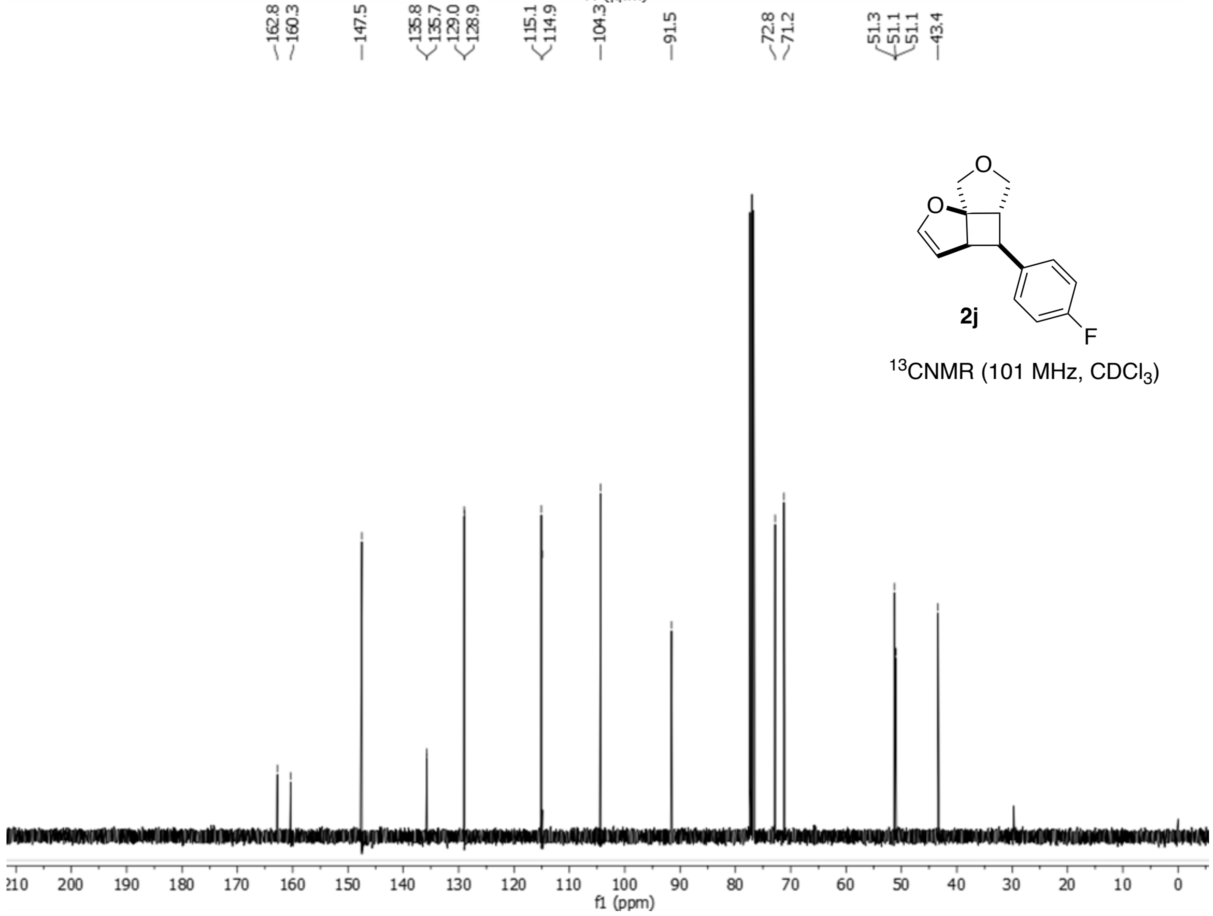

2j
F
^{13}CNMR (101 MHz, CDCl3)

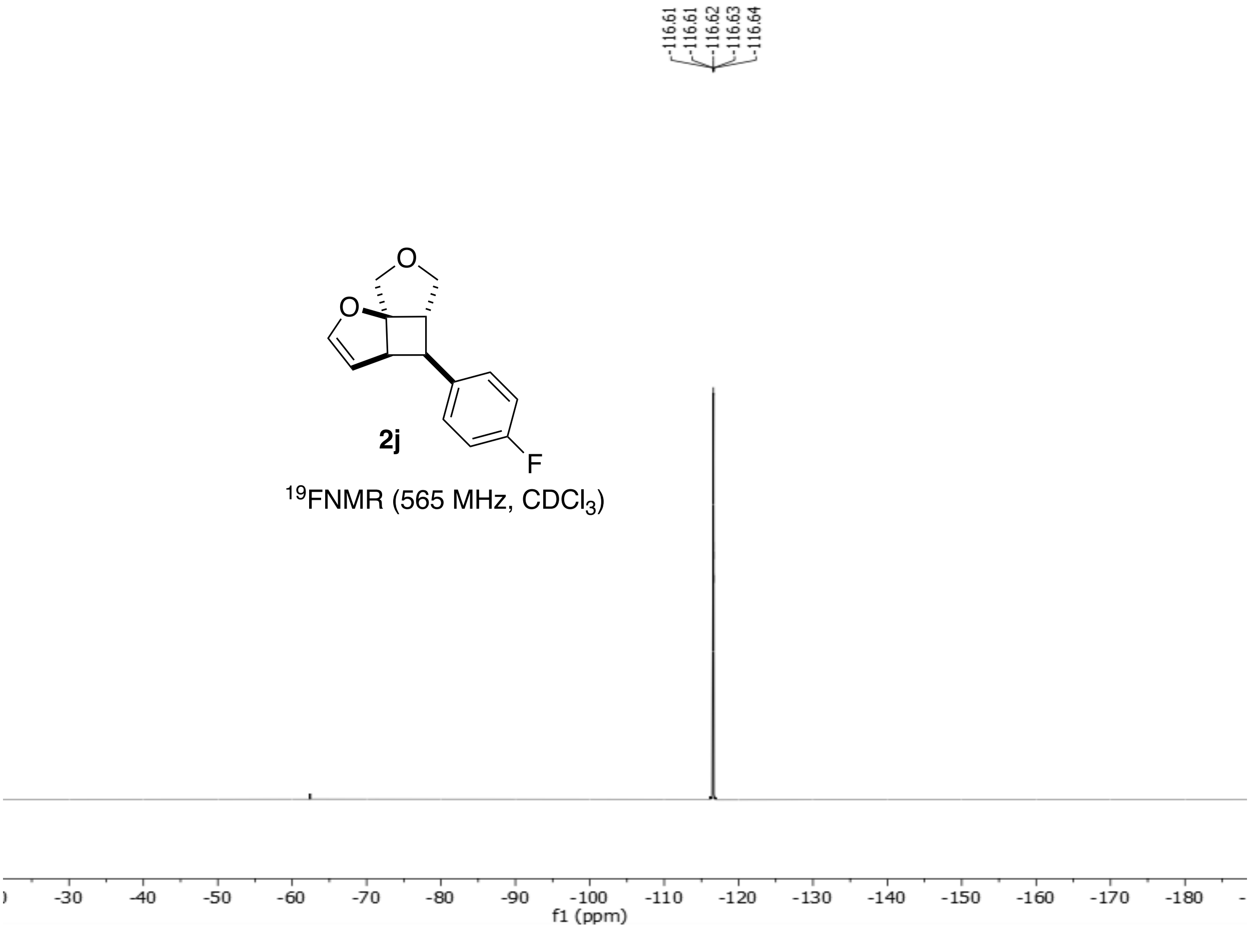
-116.61
-116.61
-116.62
-116.63
-116.64
2j
F
O
^{19}FNMR (565 MHz, $CDCl_3$)
-30 -40 -50 -60 -70 -80 -90 -100 -110 -120 -130 -140 -150 -160 -170 -180
f1 (ppm)

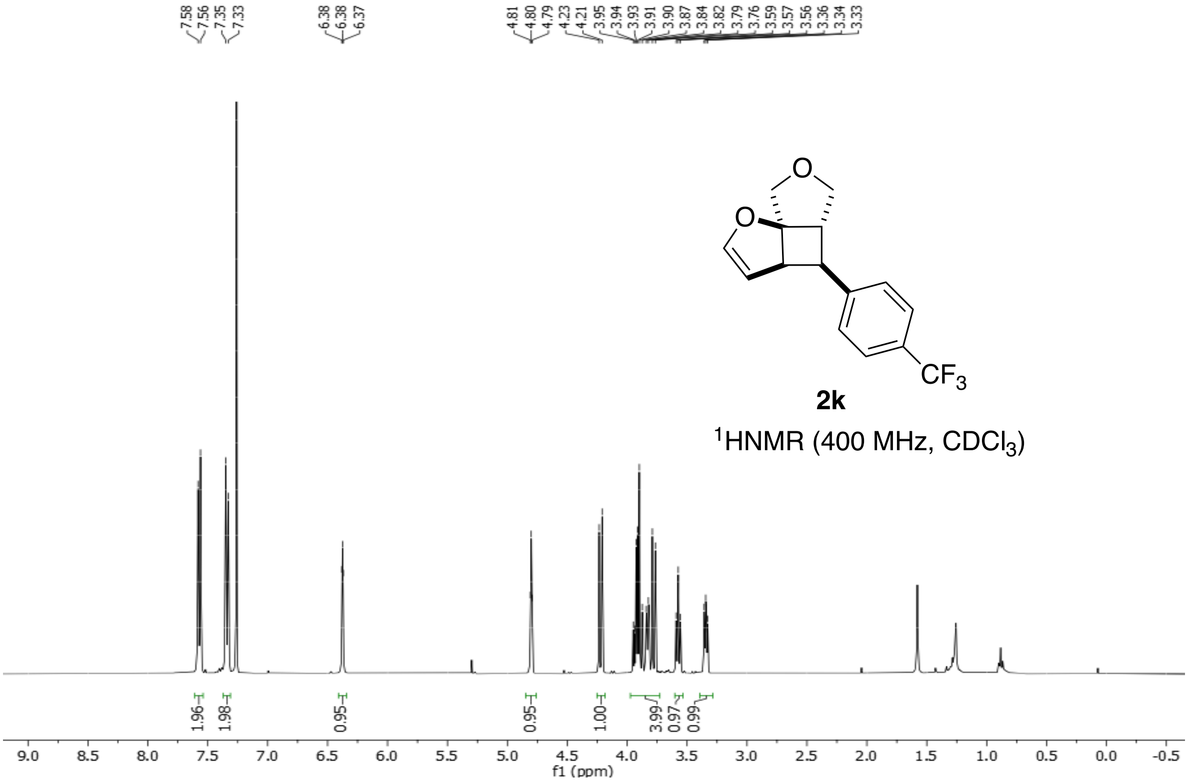
7.58
7.56
7.35
7.33
6.38
6.38
6.37
4.81
4.80
4.79
4.23
4.21
3.95
3.94
3.93
3.91
3.90
3.87
3.84
3.82
3.79
3.76
3.59
3.57
3.56
3.36
3.34
3.33
O
O
CF3
2k
1HNMR (400 MHz, CDCl3)
1.96
1.98
0.95
0.95
1.00
3.99
0.97
0.99
9.0 8.5 8.0 7.5 7.0 6.5 6.0 5.5 5.0 4.5 4.0 3.5 3.0 2.5 2.0 1.5 1.0 0.5 0.0 -0.5
f1 (ppm)

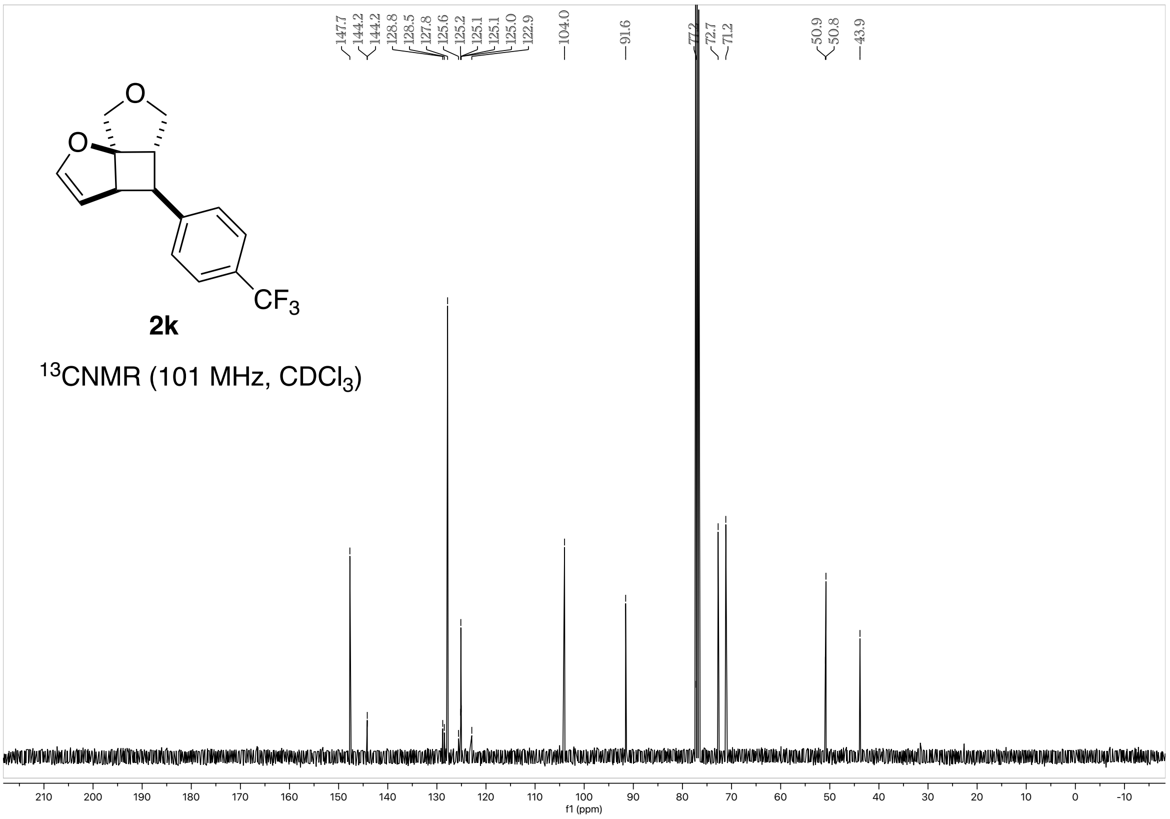
147.7
144.2
144.2
128.8
128.5
127.8
125.6
125.2
125.1
125.1
125.0
122.9
104.0
91.6
77.2
72.7
71.2
50.9
50.8
43.9
O
O
CF3
2k
13CNMR (101 MHz, CDCl3)
210 200 190 180 170 160 150 140 130 120 110 100 90 80 70 60 50 40 30 20 10 0 -10
f1 (ppm)

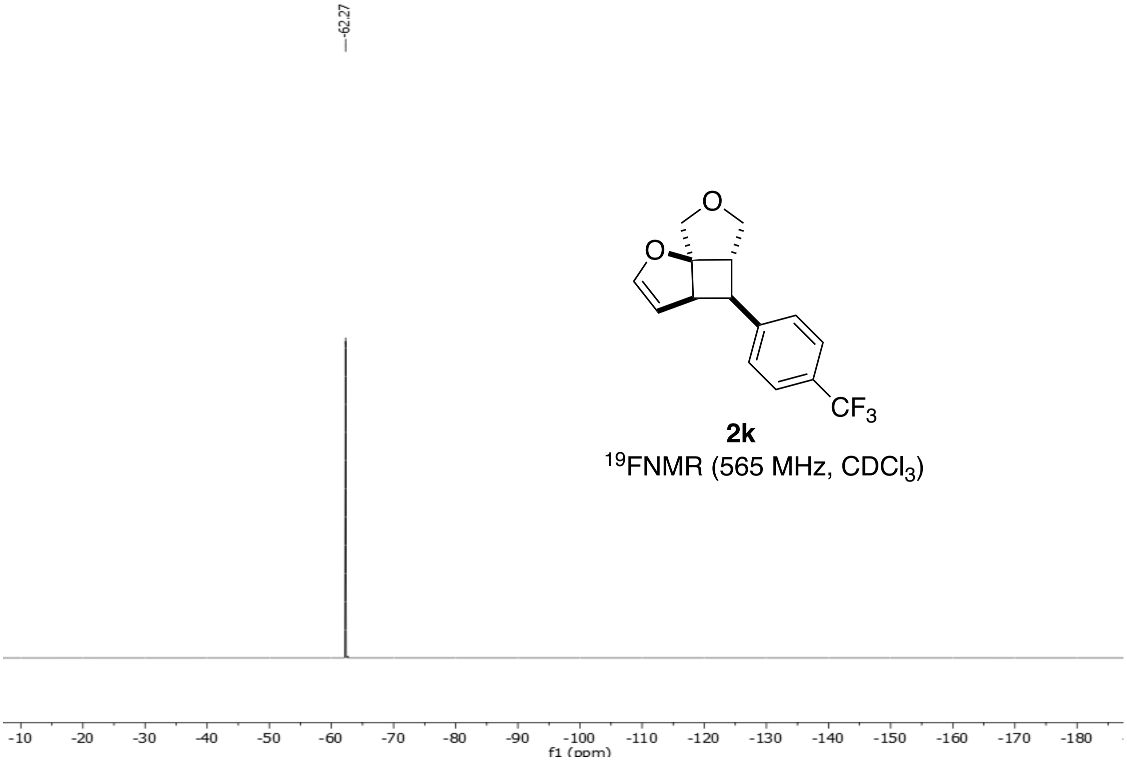

2k

^{19}FNMR (565 MHz, $CDCl_3$)

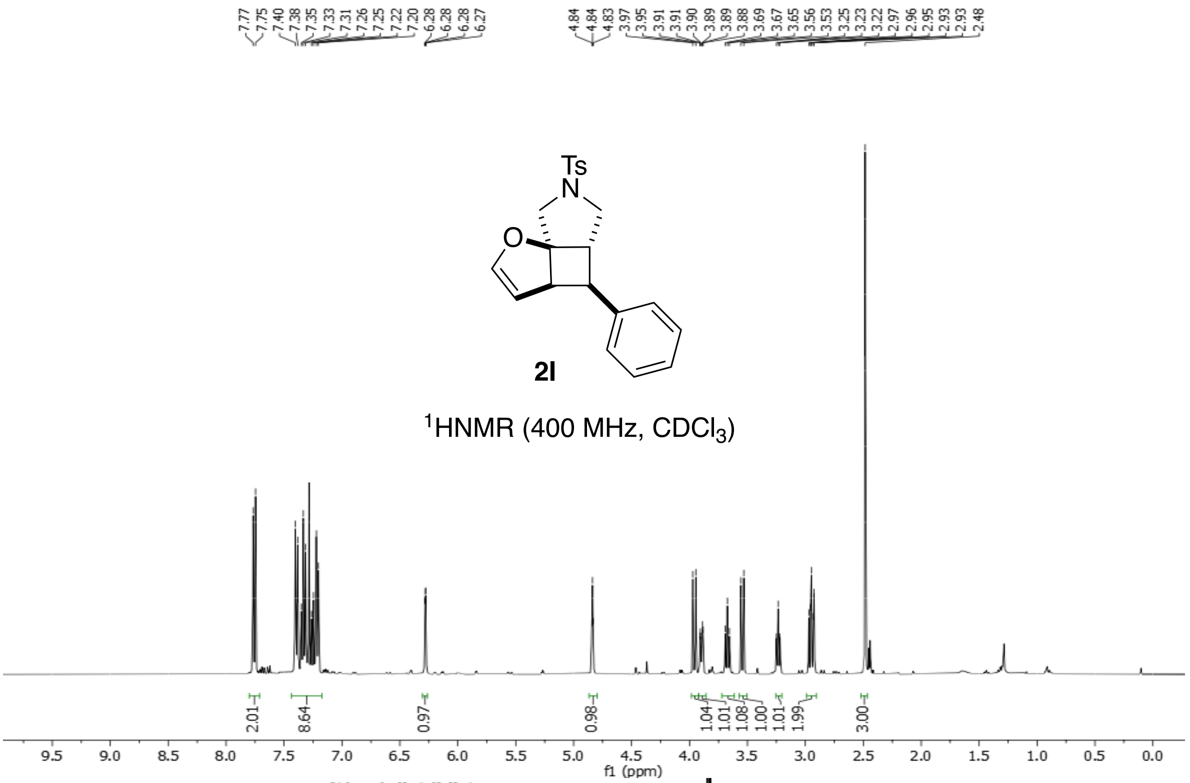
7.77
7.75
7.40
7.38
7.35
7.33
7.31
7.26
7.25
7.22
7.20
6.28
6.28
6.28
6.27
4.84
4.84
4.83
3.97
3.95
3.91
3.91
3.90
3.89
3.89
3.88
3.69
3.67
3.65
3.56
3.53
3.25
3.23
3.22
2.97
2.96
2.95
2.93
2.93
2.48
Ts
N
O
2l
^{1}HNMR (400 MHz, CDCl$_3$)
2.01
8.64
0.97
0.98
1.04
1.01
1.08
1.00
1.01
1.99
3.00
9.5
9.0
8.5
8.0
7.5
7.0
6.5
6.0
5.5
5.0
4.5
4.0
3.5
3.0
2.5
2.0
1.5
1.0
0.5
0.0
f1 (ppm)

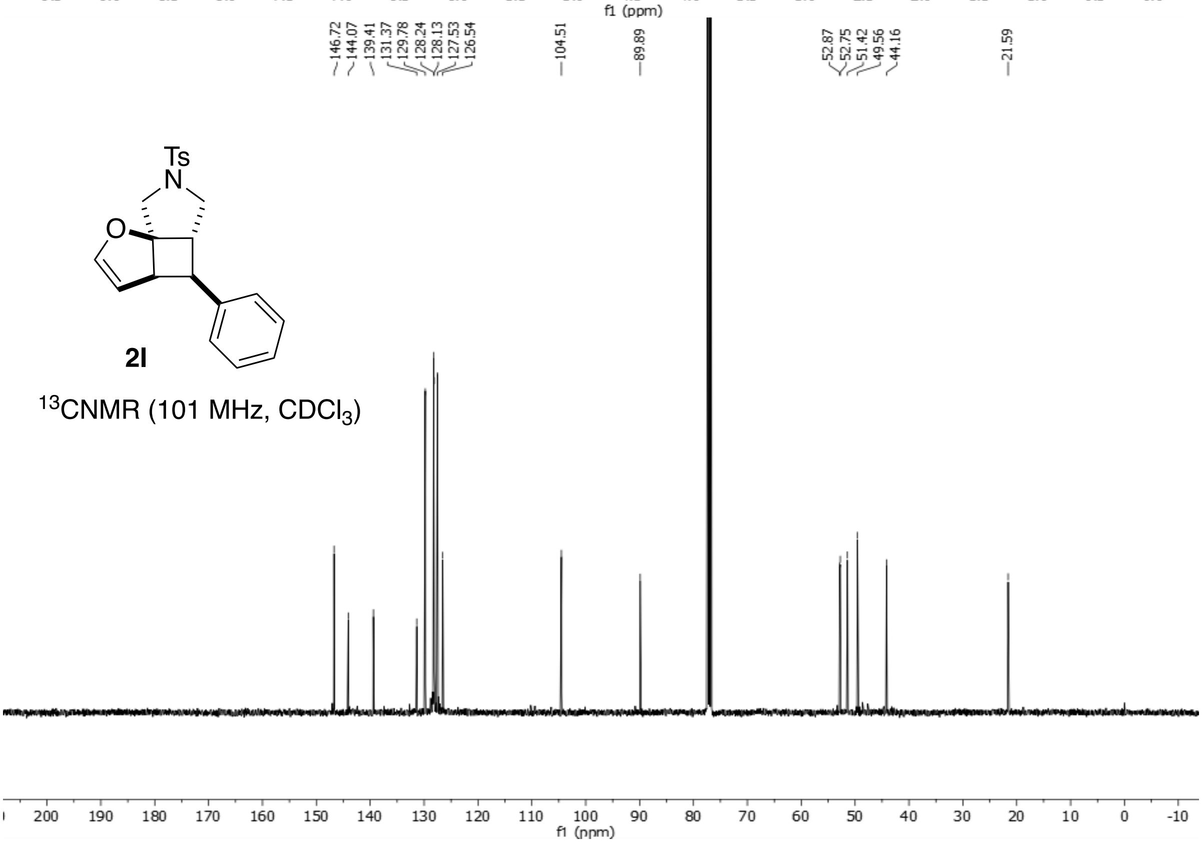
146.72
144.07
139.41
131.37
129.78
128.24
128.13
127.53
126.54
104.51
89.89
52.87
52.75
51.42
49.56
44.16
21.59
Ts
N
O
2l
^{13}CNMR (101 MHz, CDCl$_3$)
200
190
180
170
160
150
140
130
120
110
100
90
80
70
60
50
40
30
20
10
0
-10
f1 (ppm)

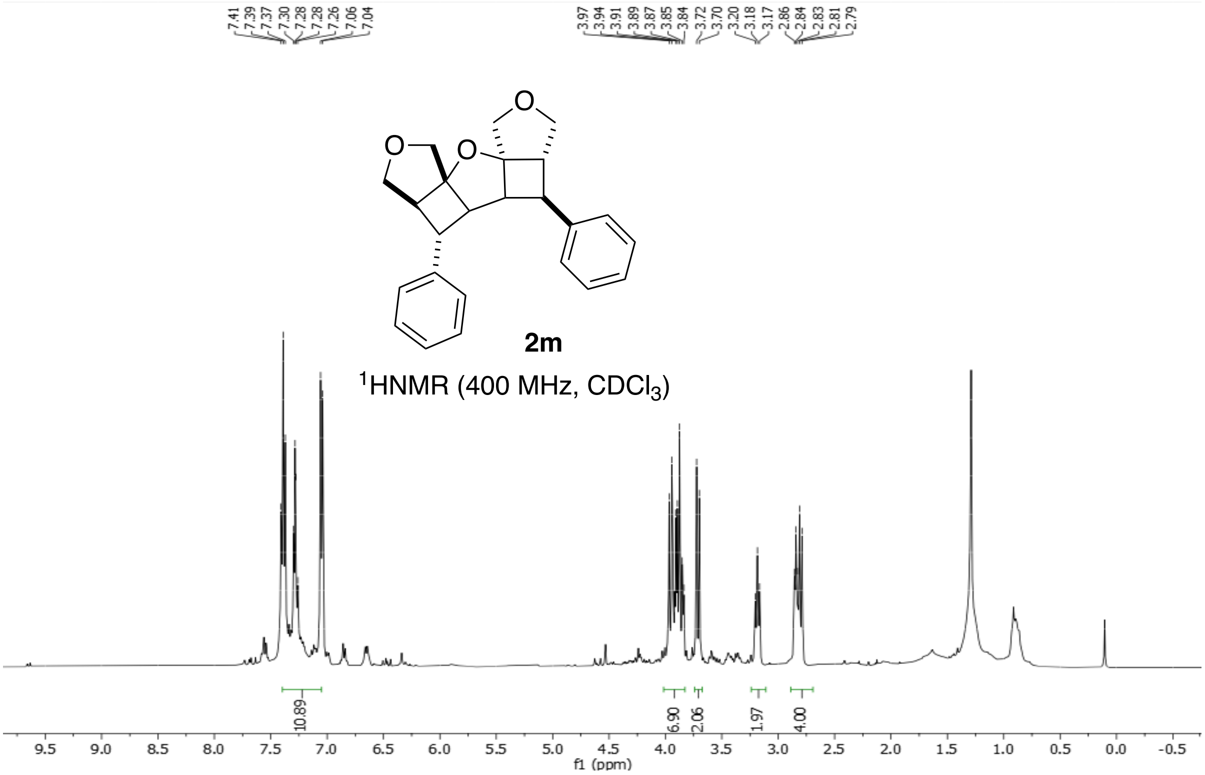
7.41
7.39
7.37
7.30
7.28
7.28
7.26
7.06
7.04
3.97
3.94
3.91
3.89
3.87
3.85
3.84
3.72
3.70
3.20
3.18
3.17
2.86
2.84
2.83
2.81
2.79
2m
^{1}HNMR (400 MHz, $CDCl_3$)
10.89
6.90
2.06
1.97
4.00
f1 (ppm)

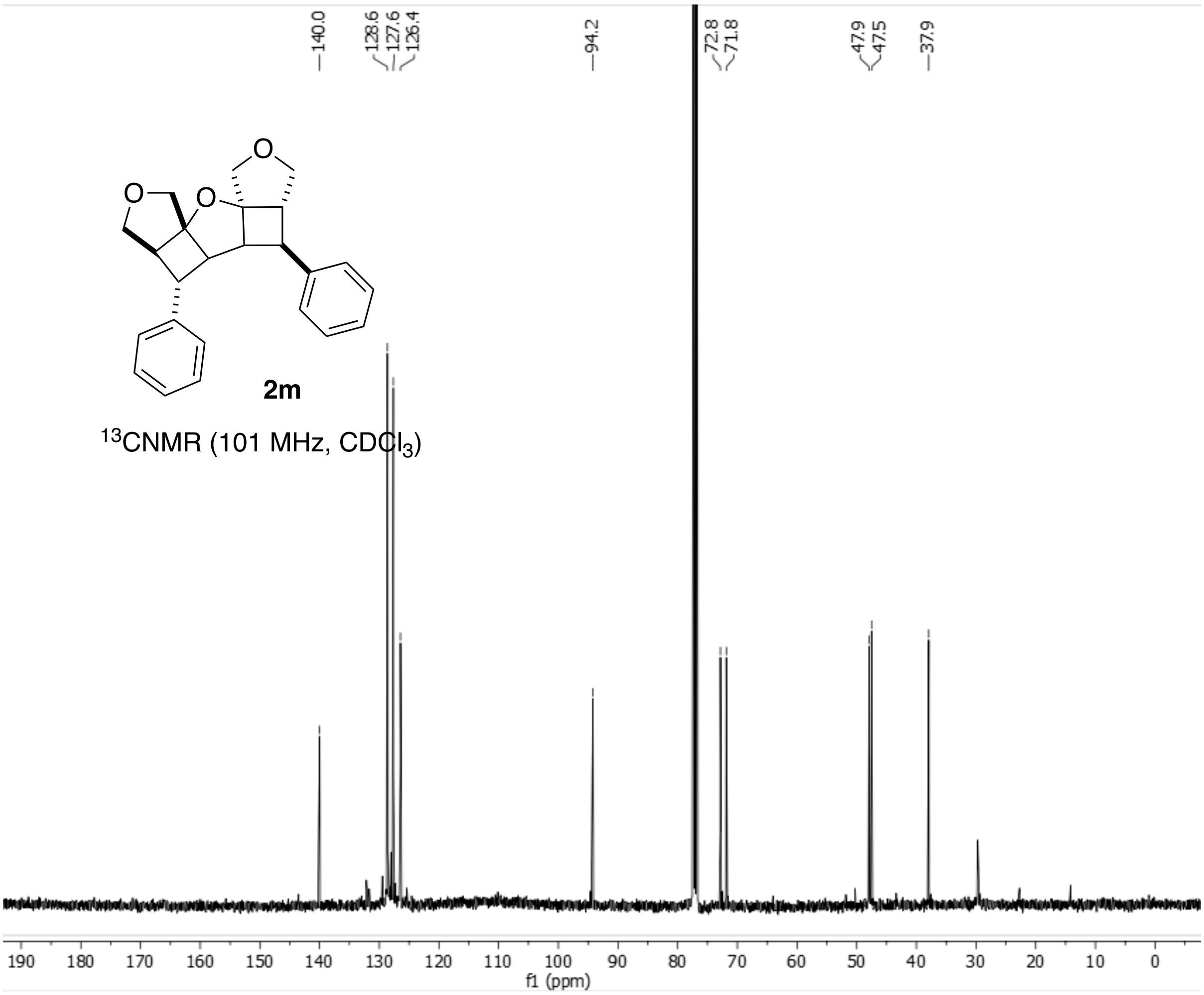
140.0
128.6
127.6
126.4
94.2
72.8
71.8
47.9
47.5
37.9
2m
^{13}CNMR (101 MHz, $CDCl_3$)
f1 (ppm)

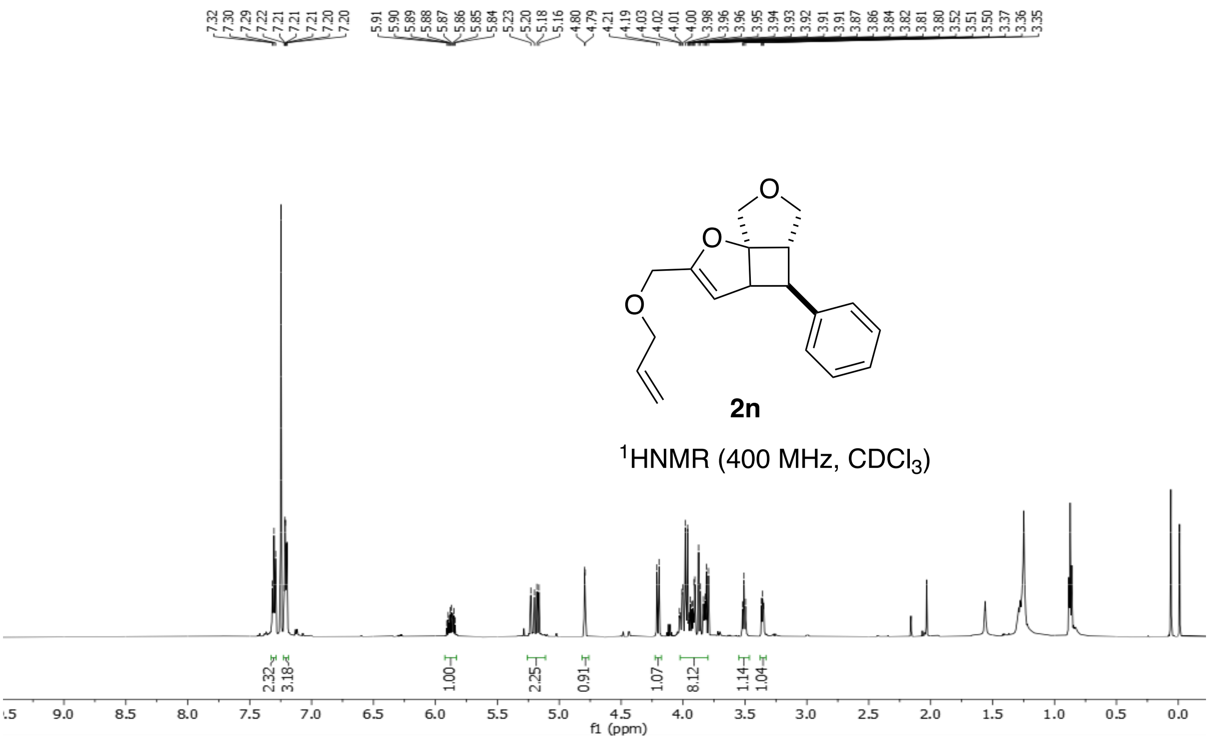
7.32 7.30 7.29 7.22 7.21 7.21 7.21 7.20 7.20
5.91 5.90 5.89 5.88 5.87 5.86 5.85 5.84 5.23 5.20 5.18 5.16 4.80 4.79 4.21 4.19 4.03 4.02 4.01 4.00 3.98 3.96 3.96 3.95 3.94 3.93 3.92 3.91 3.91 3.87 3.86 3.84 3.82 3.81 3.80 3.52 3.51 3.50 3.37 3.36 3.35
O
O
O
2n
1HNMR (400 MHz, CDCl3)
2.32 3.18 1.00 2.25 0.91 1.07 8.12 1.14 1.04
f1 (ppm)

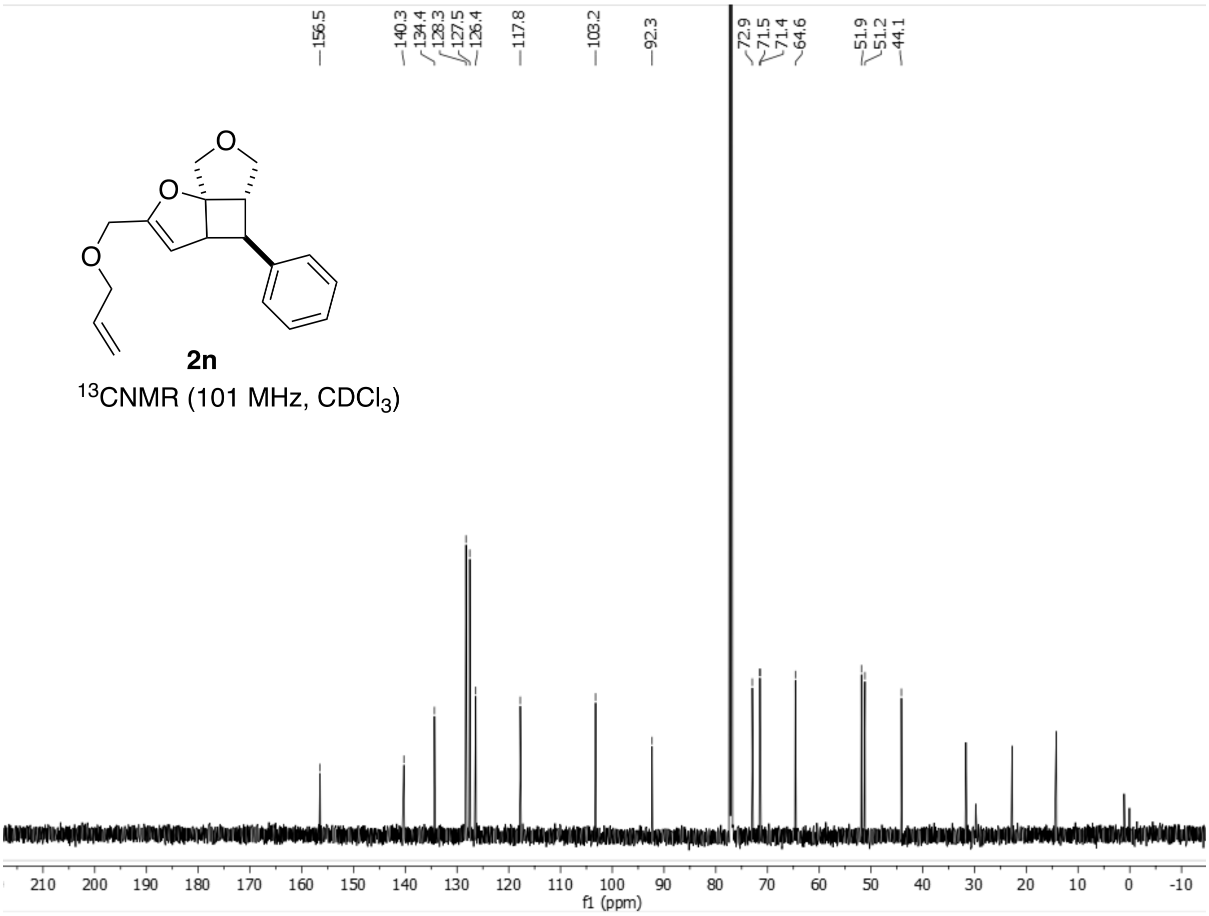
156.5
140.3 134.4 128.3 127.5 126.4
117.8
103.2
92.3
72.9 71.5 71.4 64.6
51.9 51.2 44.1
O
O
O
2n
13CNMR (101 MHz, CDCl3)
f1 (ppm)

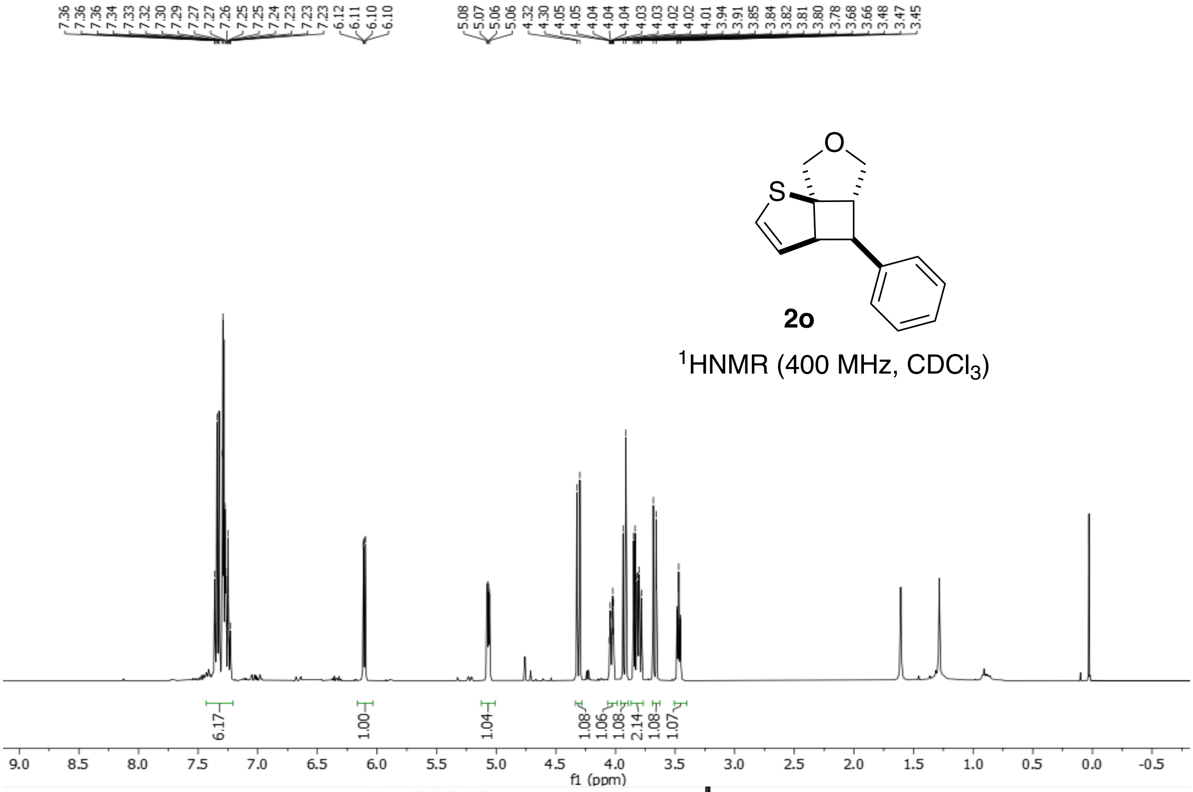
7.36 7.36 7.36 7.34 7.33 7.32 7.30 7.29 7.27 7.27 7.26 7.25 7.25 7.24 7.23 7.23 7.23 7.23 6.12 6.11 6.10 6.10
5.08 5.07 5.06 5.06 4.32 4.30 4.05 4.05 4.04 4.04 4.04 4.03 4.03 4.02 4.02 4.01 3.94 3.91 3.85 3.84 3.82 3.81 3.80 3.78 3.68 3.66 3.48 3.47 3.45
O
S
2o
1HNMR (400 MHz, CDCl3)
6.17 1.00 1.04 1.08 1.06 1.08 2.14 1.08 1.07
9.0 8.5 8.0 7.5 7.0 6.5 6.0 5.5 5.0 4.5 4.0 3.5 3.0 2.5 2.0 1.5 1.0 0.5 0.0 -0.5
f1 (ppm)

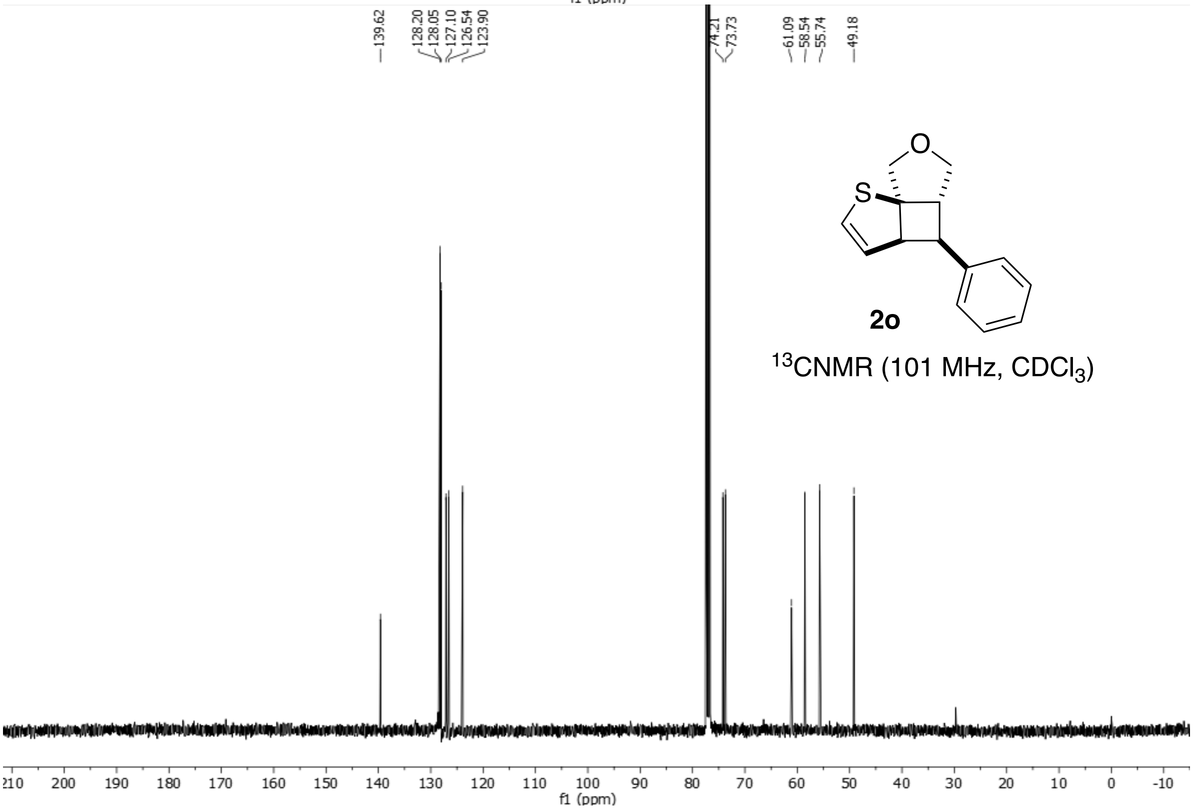
139.62
128.20 128.05 127.10 126.54 123.90
74.21 73.73
61.09 58.54 55.74
49.18
O
S
2o
13CNMR (101 MHz, CDCl3)
210 200 190 180 170 160 150 140 130 120 110 100 90 80 70 60 50 40 30 20 10 0 -10
f1 (ppm)

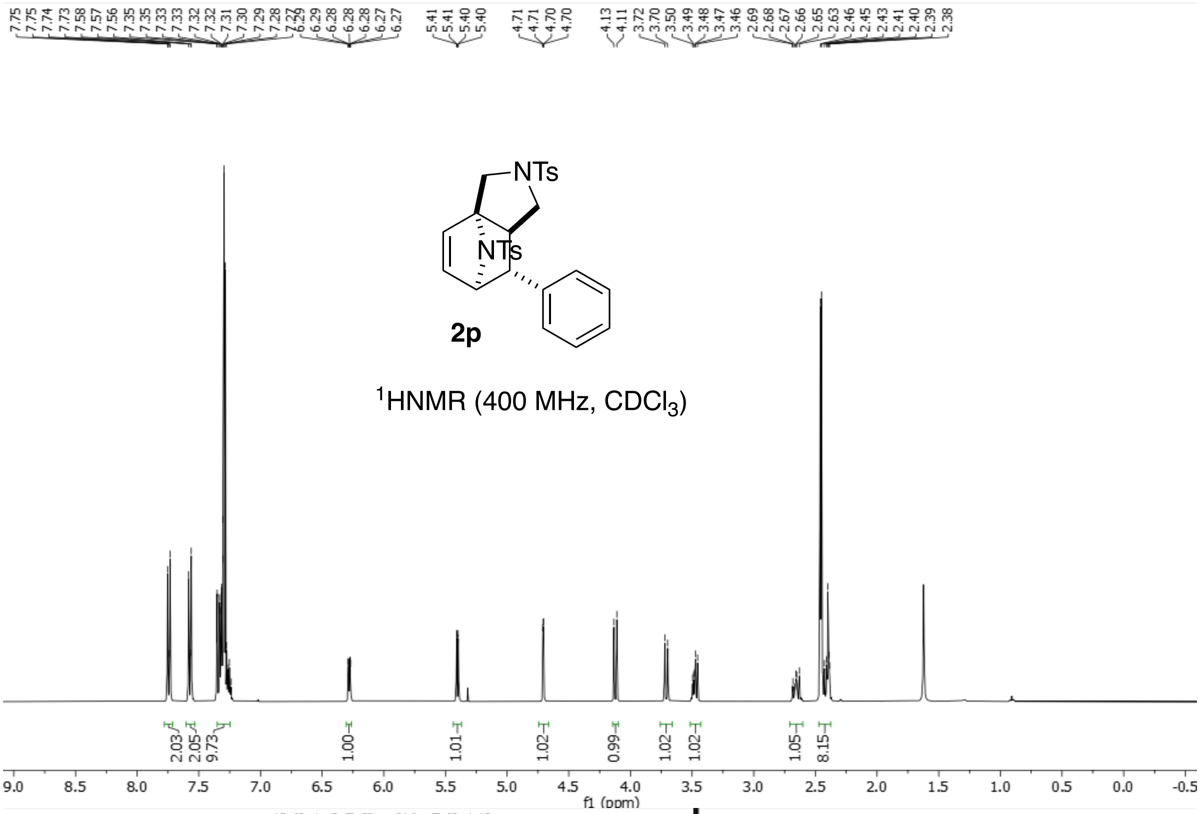
7.75 7.75 7.74 7.73 7.58 7.57 7.56 7.35 7.35 7.33 7.33 7.32 7.32 7.31 7.30 7.29 7.28 7.27 6.29 6.29 6.28 6.28 6.28 6.27 6.27 5.41 5.41 5.40 5.40 4.71 4.71 4.70 4.70 4.13 4.11 3.72 3.70 3.50 3.49 3.48 3.47 3.46 2.69 2.68 2.67 2.66 2.65 2.63 2.46 2.45 2.43 2.41 2.40 2.39 2.38
NTs
NTs
2p
^{1}HNMR (400 MHz, $CDCl_3$)
2.03 2.05 9.73 1.00 1.01 1.02 0.99 1.02 1.02 1.05 8.15
9.0 8.5 8.0 7.5 7.0 6.5 6.0 5.5 5.0 4.5 4.0 3.5 3.0 2.5 2.0 1.5 1.0 0.5 0.0 -0.5
f1 (ppm)

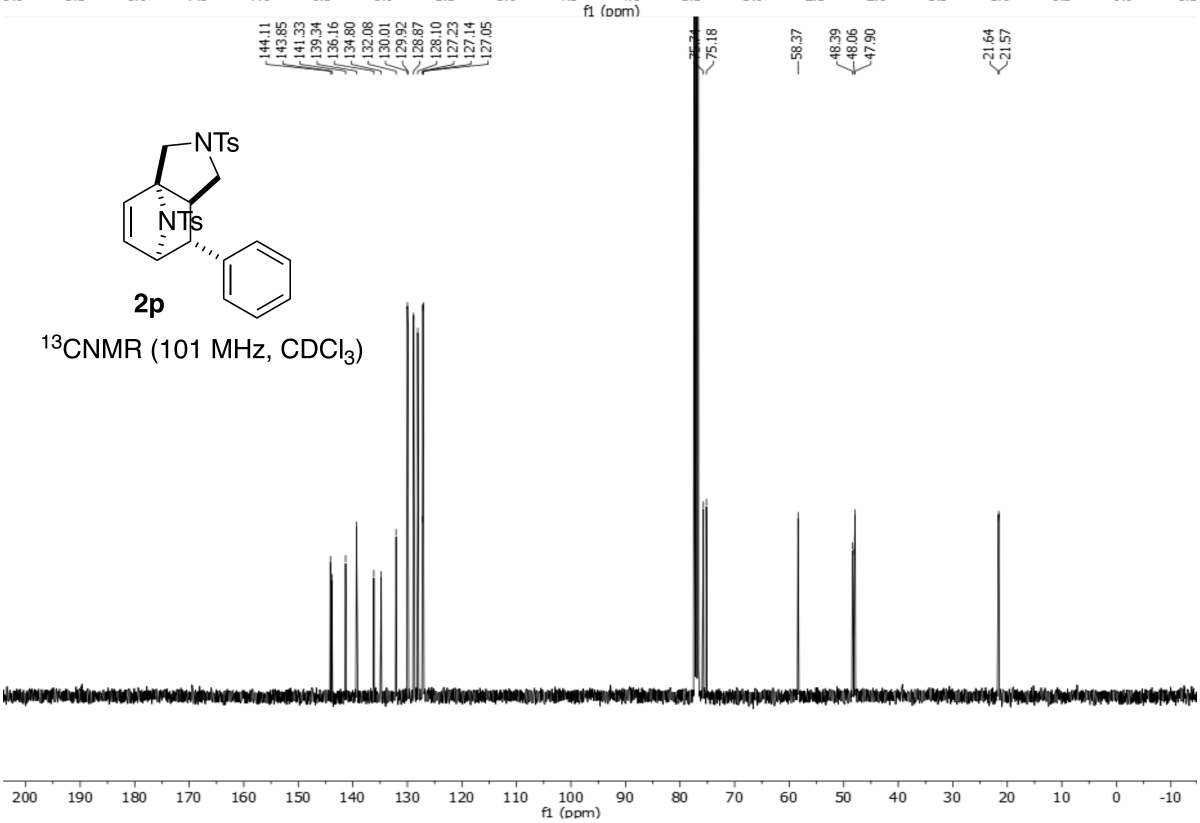
144.11 143.85 141.33 139.34 136.16 134.80 132.08 130.01 129.92 128.87 128.10 127.23 127.14 127.05
75.18
58.37
48.39 48.06 47.90
21.64 21.57
NTs
NTs
2p
^{13}CNMR (101 MHz, $CDCl_3$)
200 190 180 170 160 150 140 130 120 110 100 90 80 70 60 50 40 30 20 10 0 -10
f1 (ppm)

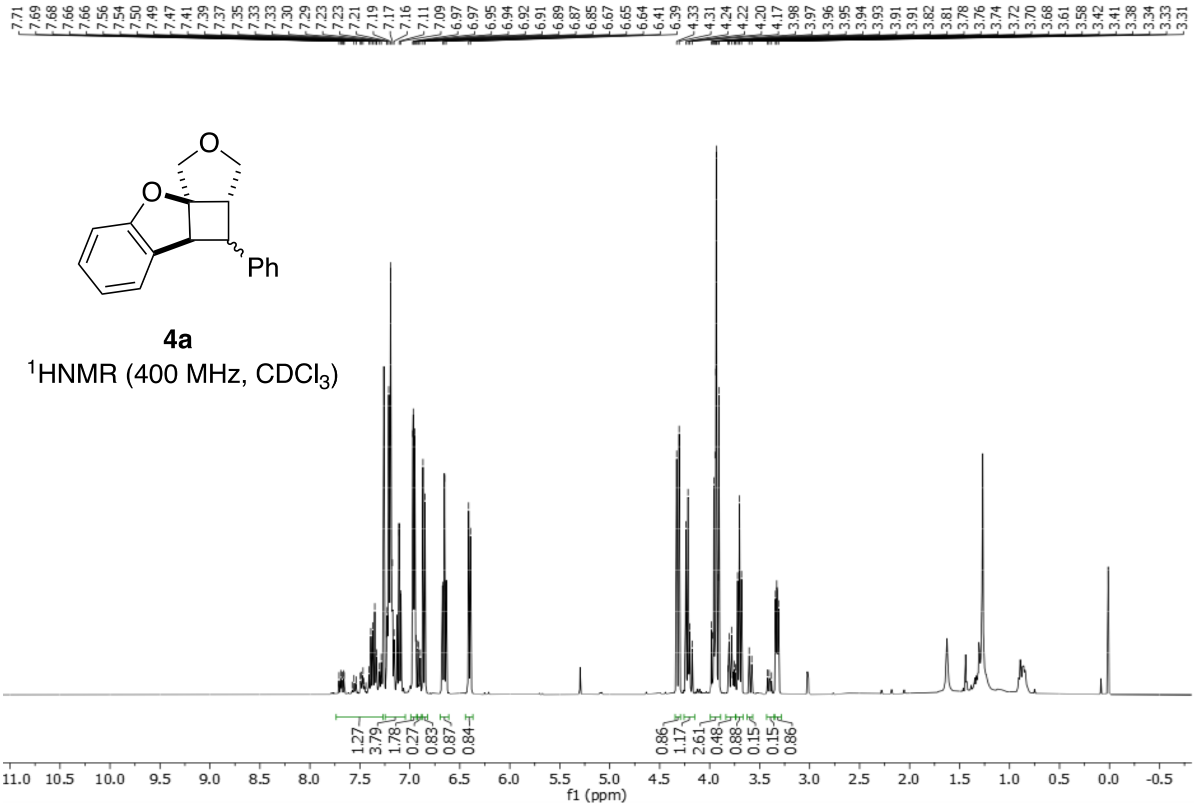
O
O
Ph
4a
1HNMR (400 MHz, CDCl3)
f1 (ppm)

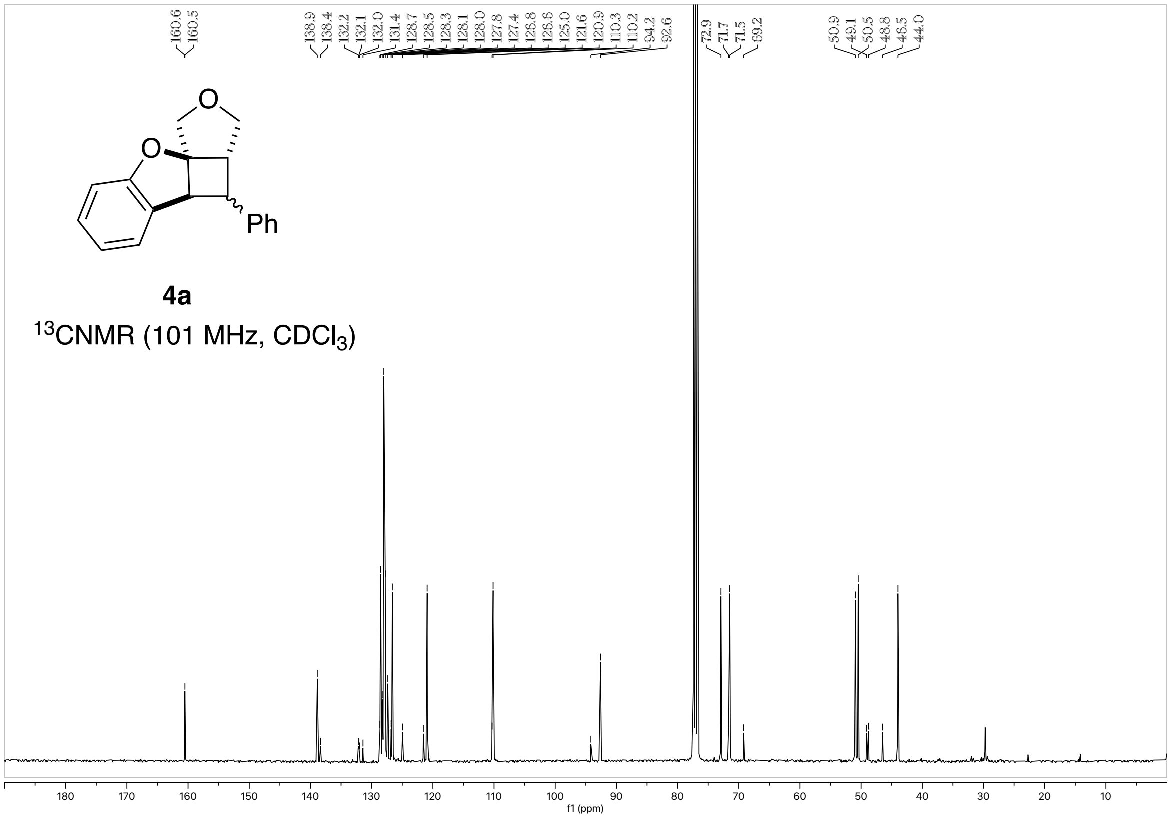
O
O
Ph
4a
13CNMR (101 MHz, CDCl3)
f1 (ppm)

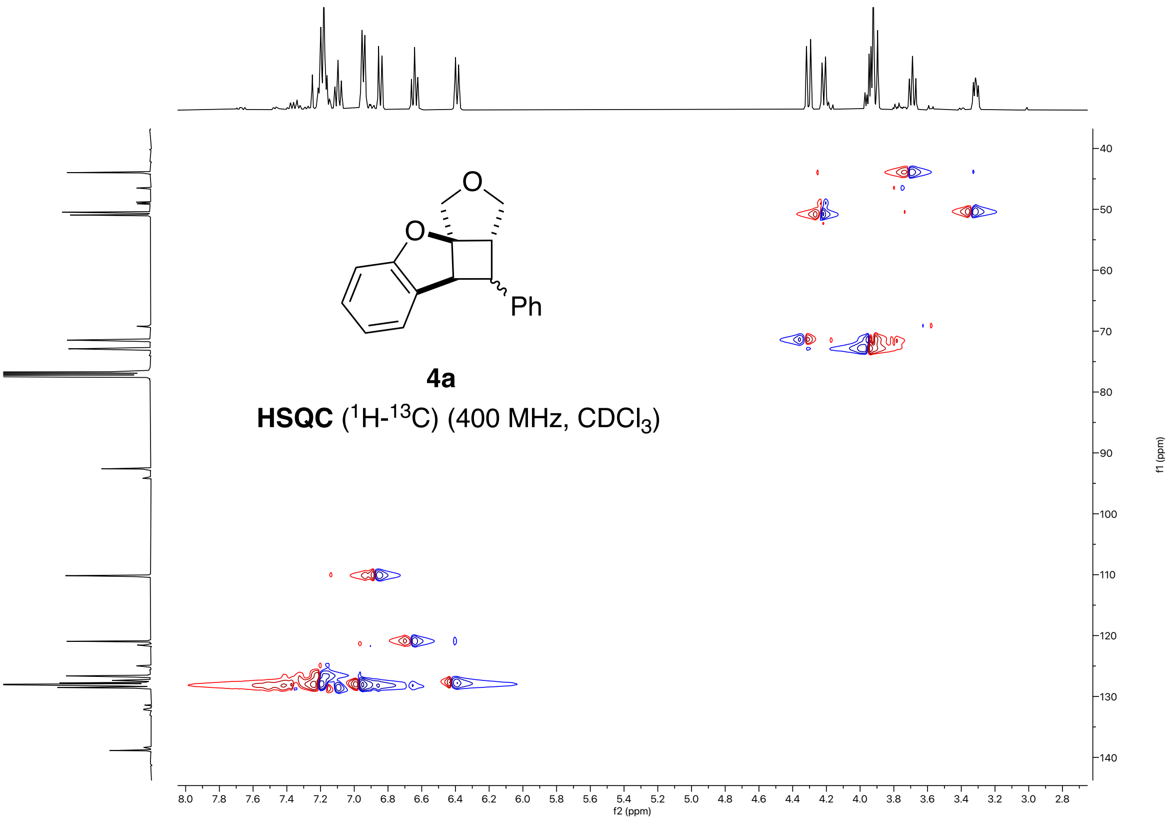
O
O
Ph
4a
HSQC (^{1}H-^{13}C) (400 MHz, $CDCl_3$)
f2 (ppm)
f1 (ppm)

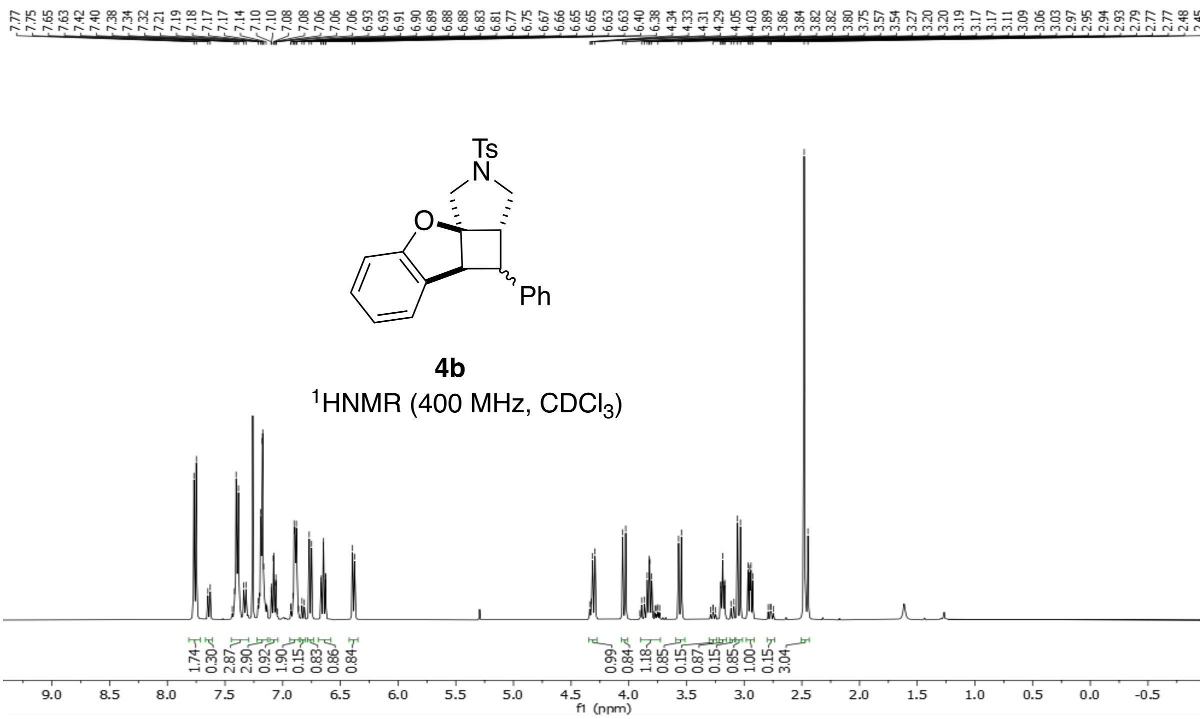
7.77 7.75 7.65 7.63 7.42 7.40 7.38 7.34 7.32 7.21 7.19 7.18 7.17 7.17 7.14 7.10 7.08 7.08 7.06 7.06 7.06 7.06 6.93 6.93 6.91 6.90 6.89 6.88 6.88 6.83 6.81 6.77 6.75 6.67 6.66 6.65 6.65 6.63 6.63 6.40 6.38 4.34 4.33 4.31 4.29 4.05 4.03 3.89 3.86 3.84 3.82 3.82 3.80 3.75 3.57 3.54 3.27 3.20 3.19 3.17 3.17 3.11 3.09 3.06 3.03 2.97 2.95 2.94 2.93 2.79 2.77 2.48
Ts
N
O
Ph
4b
^{1}HNMR (400 MHz, CDCl$_3$)
1.74 0.30 2.87 2.90 0.92 1.90 0.15 0.83 0.86 0.84
0.99 0.84 1.18 0.85 0.15 0.87 0.15 0.85 1.00 0.15 3.04
9.0 8.5 8.0 7.5 7.0 6.5 6.0 5.5 5.0 4.5 4.0 3.5 3.0 2.5 2.0 1.5 1.0 0.5 0.0 -0.5
f1 (ppm)

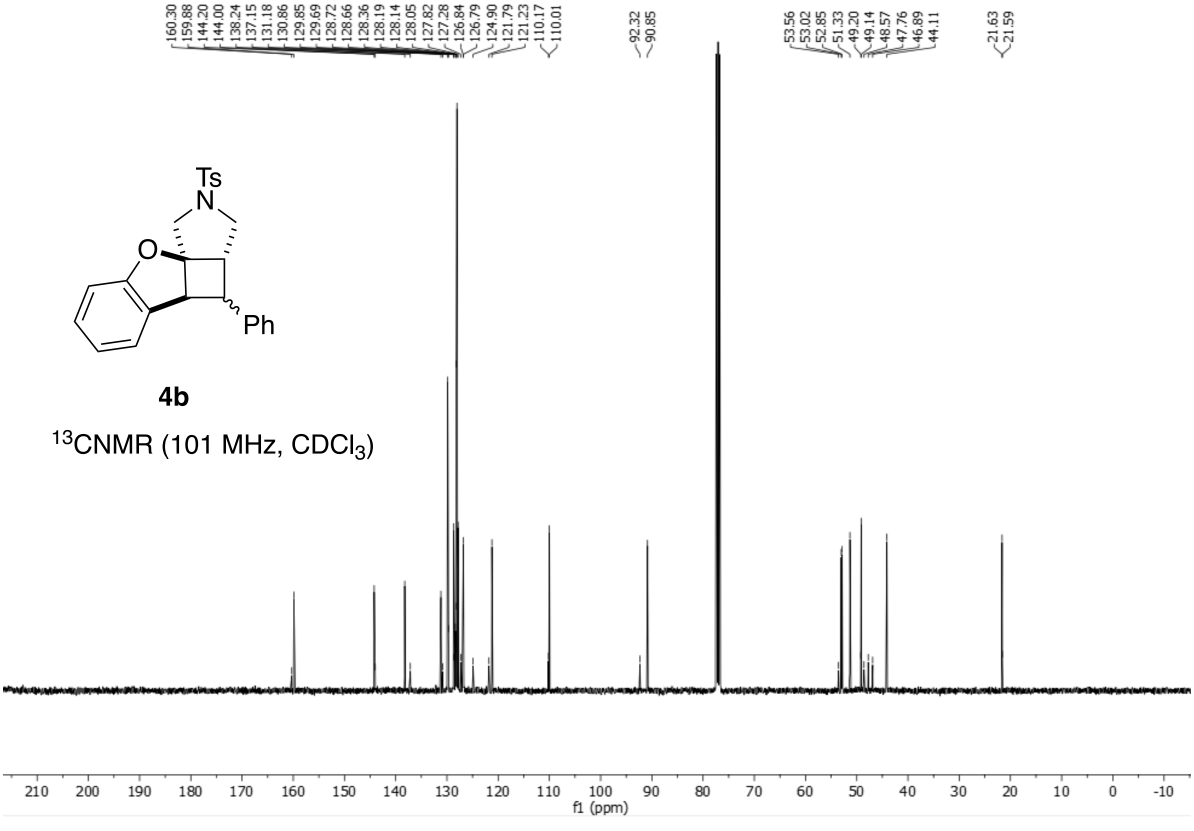
160.30 159.88 144.20 144.00 138.24 137.15 131.18 130.86 129.85 129.69 128.72 128.66 128.36 128.19 128.14 128.05 127.82 127.28 126.84 126.79 124.90 121.79 121.23 110.17 110.01
92.32 90.85
53.56 53.02 52.85 51.33 49.20 49.14 48.57 47.76 46.89 44.11
21.63 21.59
Ts
N
O
Ph
4b
^{13}CNMR (101 MHz, CDCl$_3$)
210 200 190 180 170 160 150 140 130 120 110 100 90 80 70 60 50 40 30 20 10 0 -10
f1 (ppm)

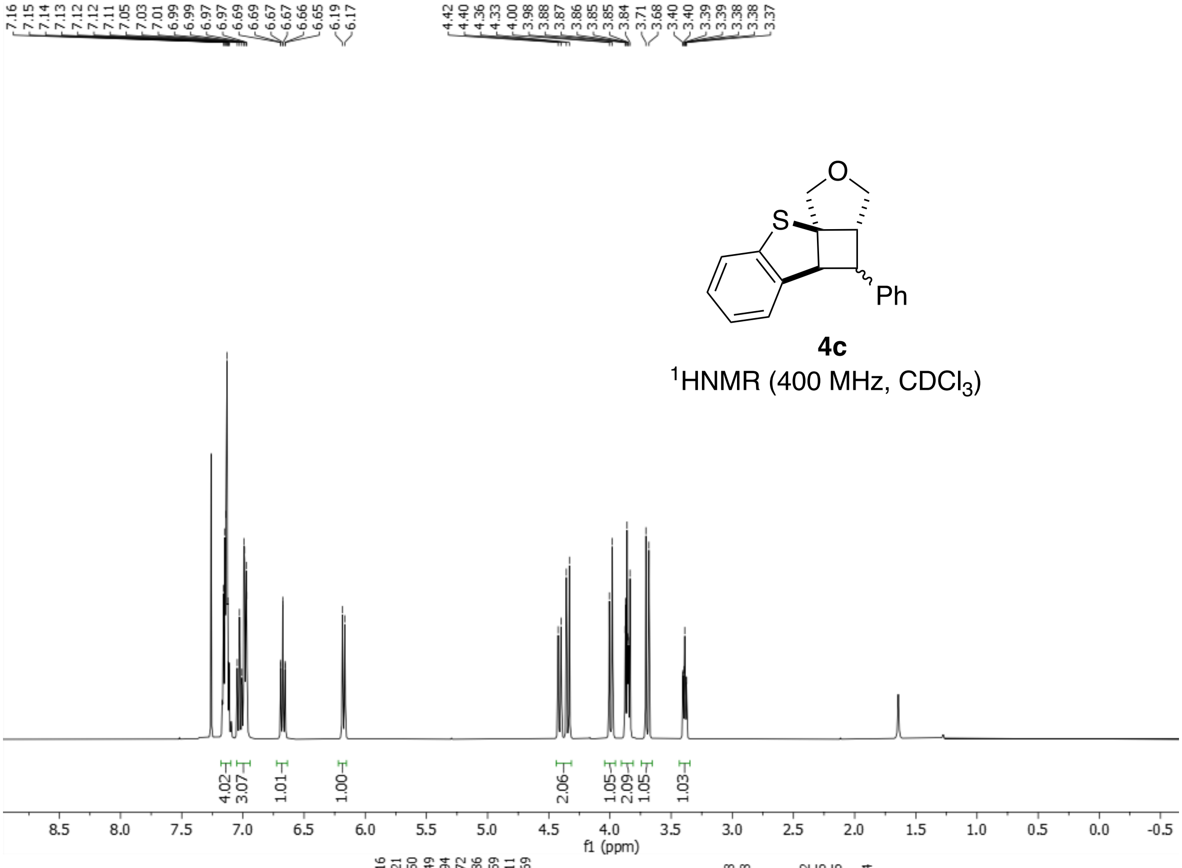

4c

^{1}HNMR (400 MHz, $CDCl_3$)

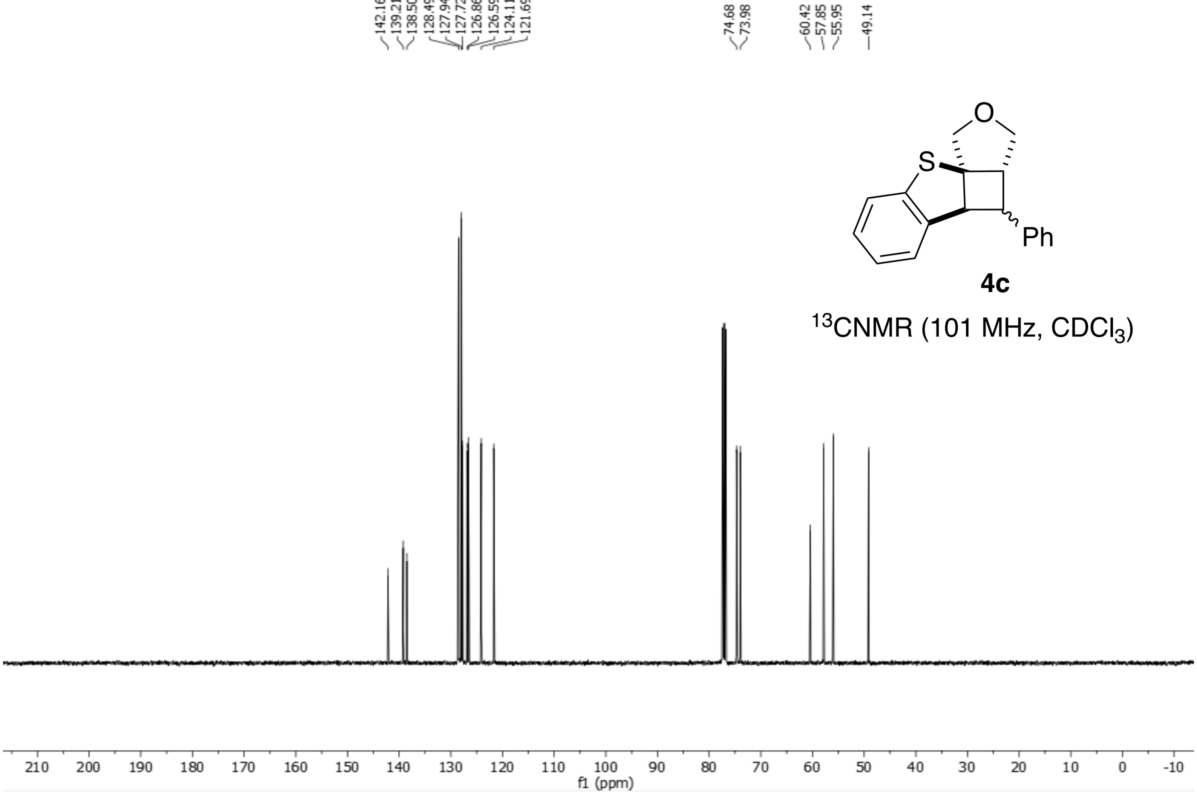

4c

^{13}CNMR (101 MHz, $CDCl_3$)

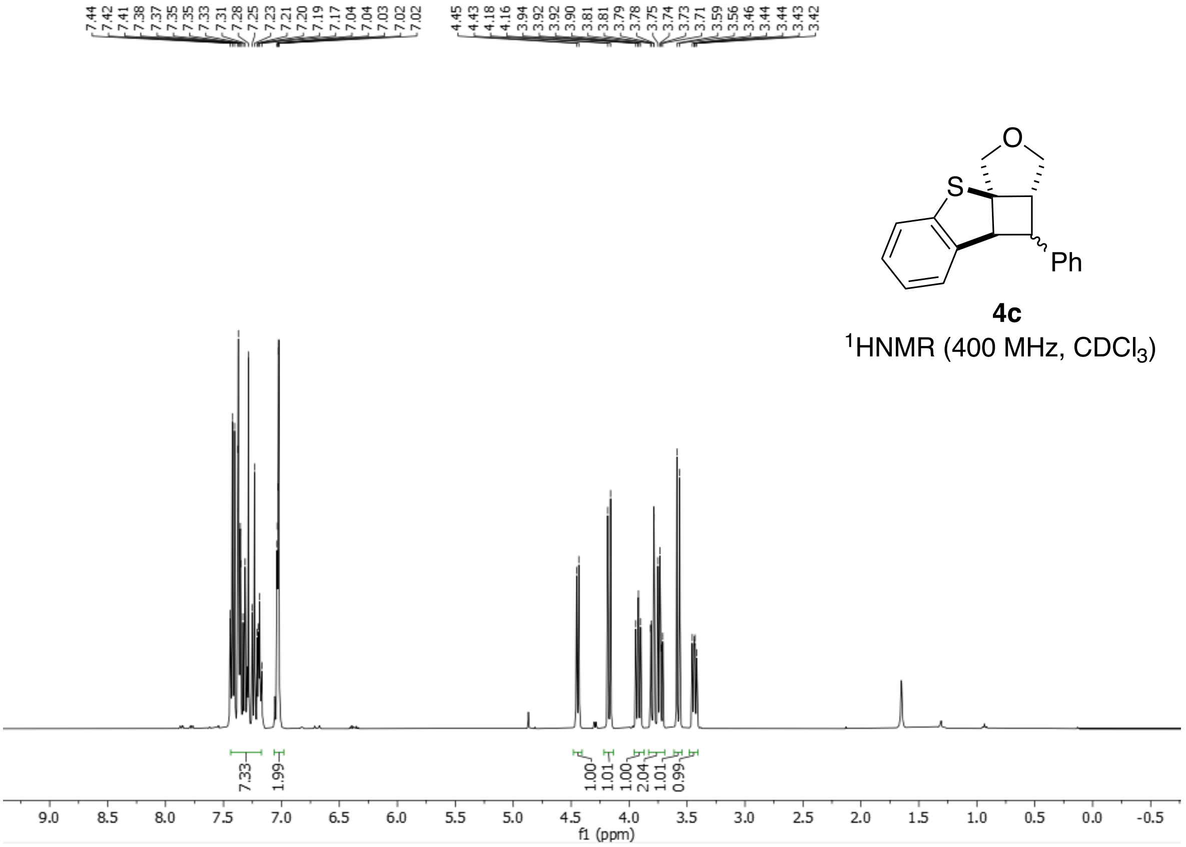
7.44 7.42 7.41 7.38 7.37 7.35 7.35 7.33 7.31 7.28 7.25 7.23 7.21 7.20 7.19 7.17 7.04 7.04 7.03 7.02 7.02
4.45 4.43 4.18 4.16 3.94 3.92 3.92 3.90 3.81 3.81 3.79 3.78 3.75 3.74 3.73 3.71 3.59 3.56 3.46 3.44 3.43 3.42
O
S
Ph
4c
^{1}HNMR (400 MHz, $CDCl_3$)
7.33 1.99 1.00 1.01 1.00 2.04 1.01 0.99
9.0 8.5 8.0 7.5 7.0 6.5 6.0 5.5 5.0 4.5 4.0 3.5 3.0 2.5 2.0 1.5 1.0 0.5 0.0 -0.5
f1 (ppm)

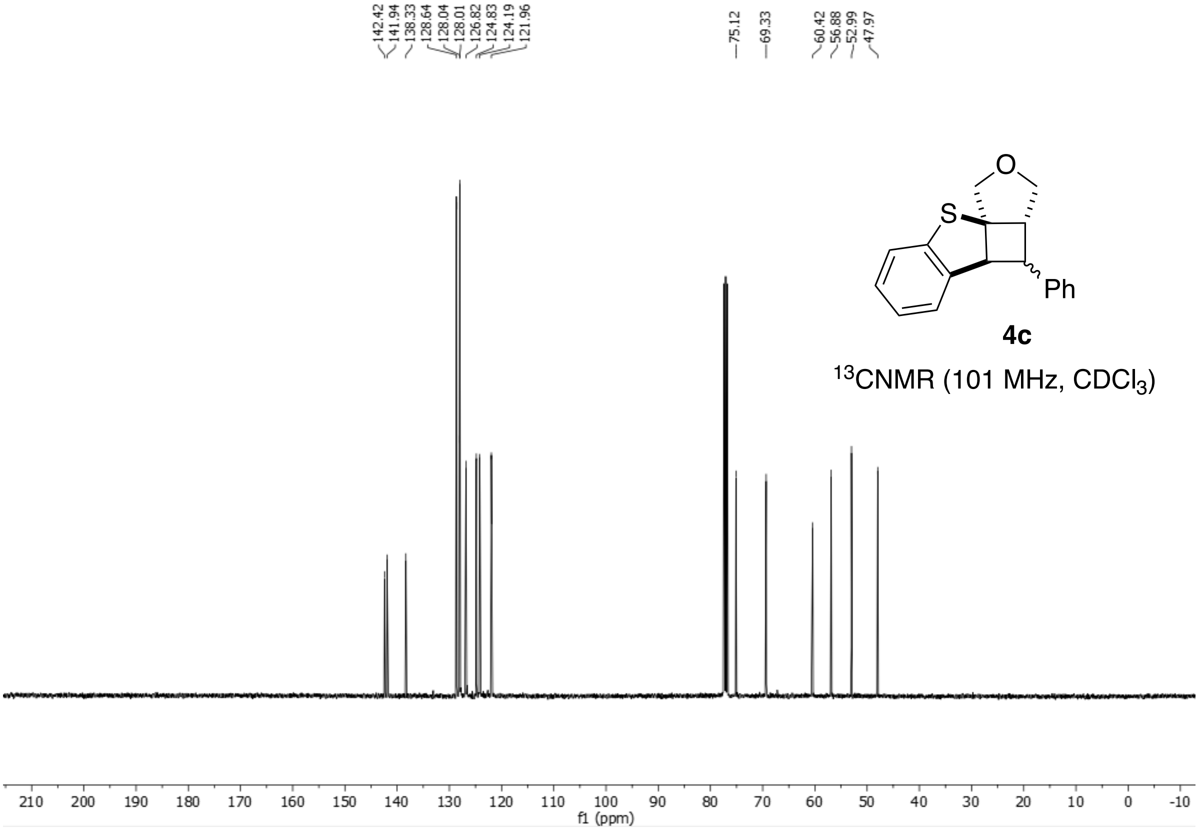
142.42 141.94 138.33 128.64 128.04 128.01 126.82 124.83 124.19 121.96
75.12
69.33
60.42 56.88 52.99 47.97
O
S
Ph
4c
^{13}CNMR (101 MHz, $CDCl_3$)
210 200 190 180 170 160 150 140 130 120 110 100 90 80 70 60 50 40 30 20 10 0 -10
f1 (ppm)

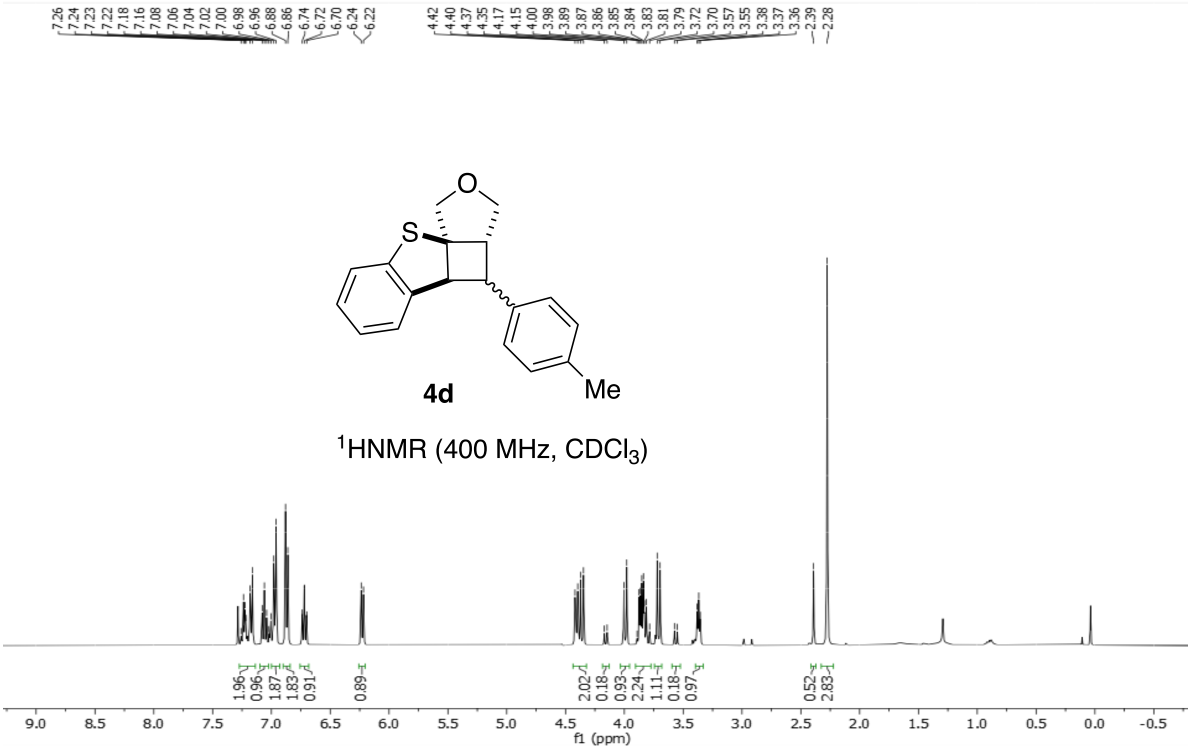

4d
^{1}HNMR (400 MHz, CDCl$_3$)
f1 (ppm)

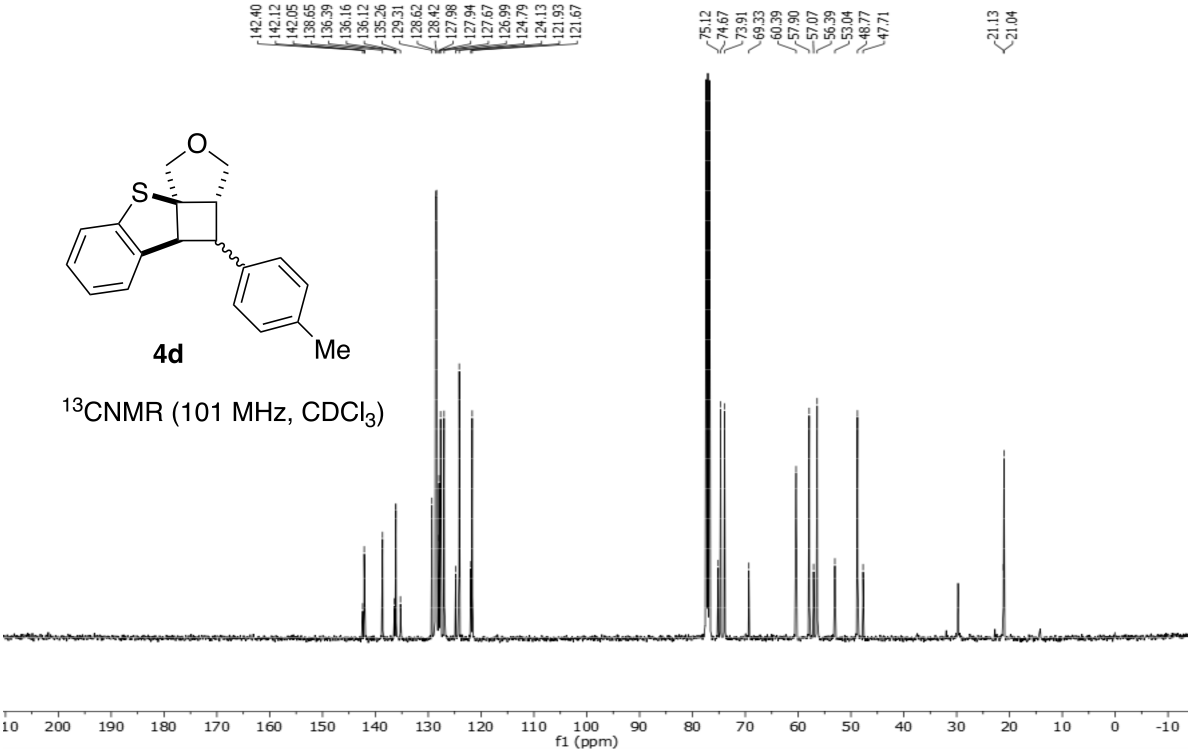

4d
^{13}CNMR (101 MHz, CDCl$_3$)
f1 (ppm)

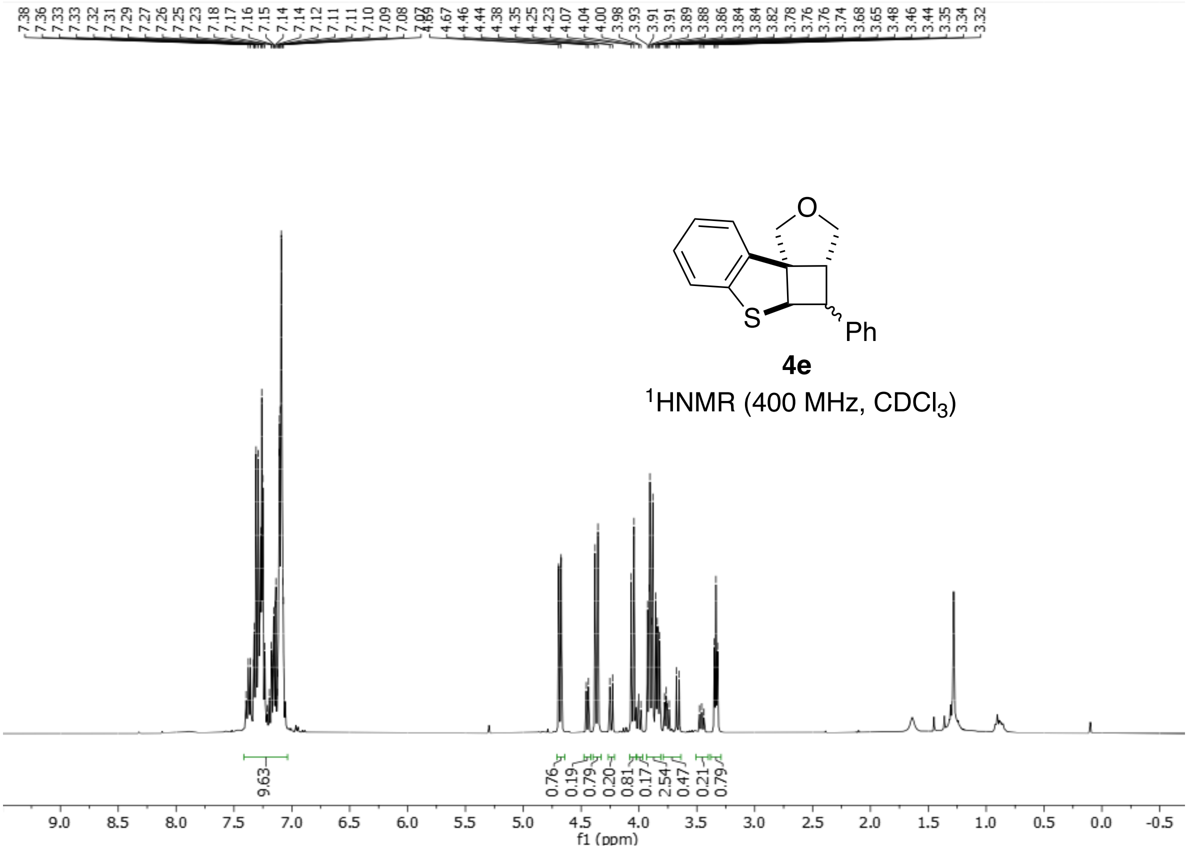
7.38 7.36 7.33 7.33 7.32 7.31 7.29 7.27 7.26 7.25 7.23 7.18 7.17 7.16 7.15 7.14 7.14 7.12 7.11 7.11 7.10 7.09 7.08 7.07 4.69 4.67 4.46 4.44 4.38 4.35 4.25 4.23 4.07 4.04 4.00 3.98 3.93 3.91 3.91 3.89 3.88 3.86 3.84 3.84 3.82 3.78 3.76 3.76 3.74 3.68 3.65 3.48 3.46 3.44 3.35 3.34 3.32
O
S
Ph
4e
1HNMR (400 MHz, CDCl3)
9.63
0.76
0.19
0.79
0.20
0.81
0.17
2.54
0.47
0.21
0.79
9.0 8.5 8.0 7.5 7.0 6.5 6.0 5.5 5.0 4.5 4.0 3.5 3.0 2.5 2.0 1.5 1.0 0.5 0.0 -0.5
f1 (ppm)

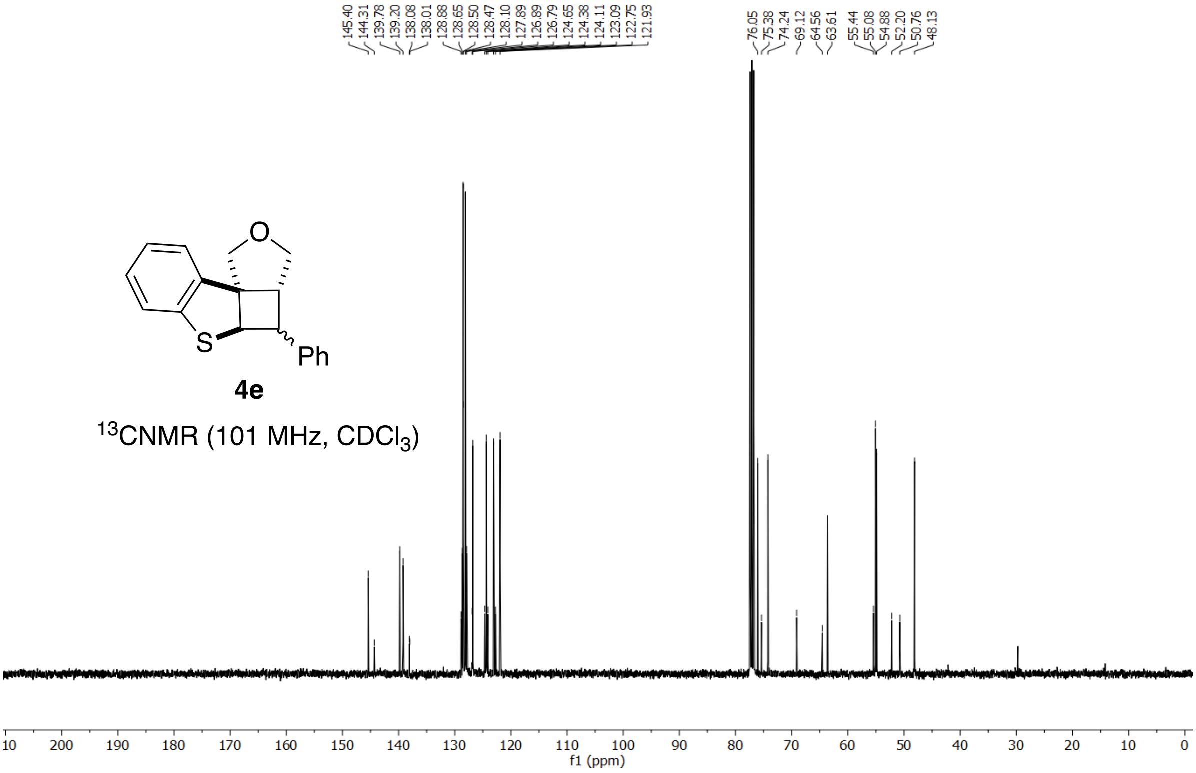
145.40 144.31 139.78 139.20 138.08 138.01 128.88 128.65 128.50 128.47 128.10 127.89 126.89 126.79 124.65 124.38 124.11 123.09 122.75 121.93
76.05 75.38 74.24 69.12 64.56 63.61 55.44 55.08 54.88 52.20 50.76 48.13
O
S
Ph
4e
13CNMR (101 MHz, CDCl3)
10 200 190 180 170 160 150 140 130 120 110 100 90 80 70 60 50 40 30 20 10 0
f1 (ppm)

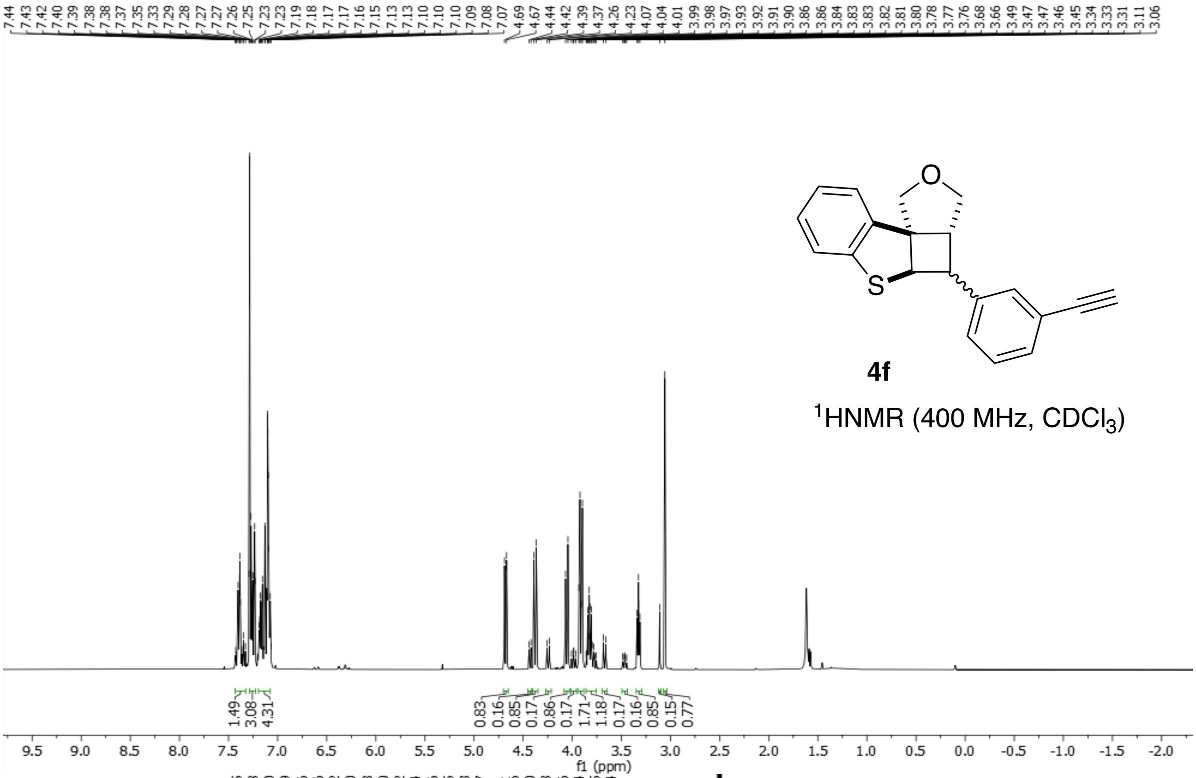

4f
^{1}HNMR (400 MHz, CDCl$_3$)
f1 (ppm)

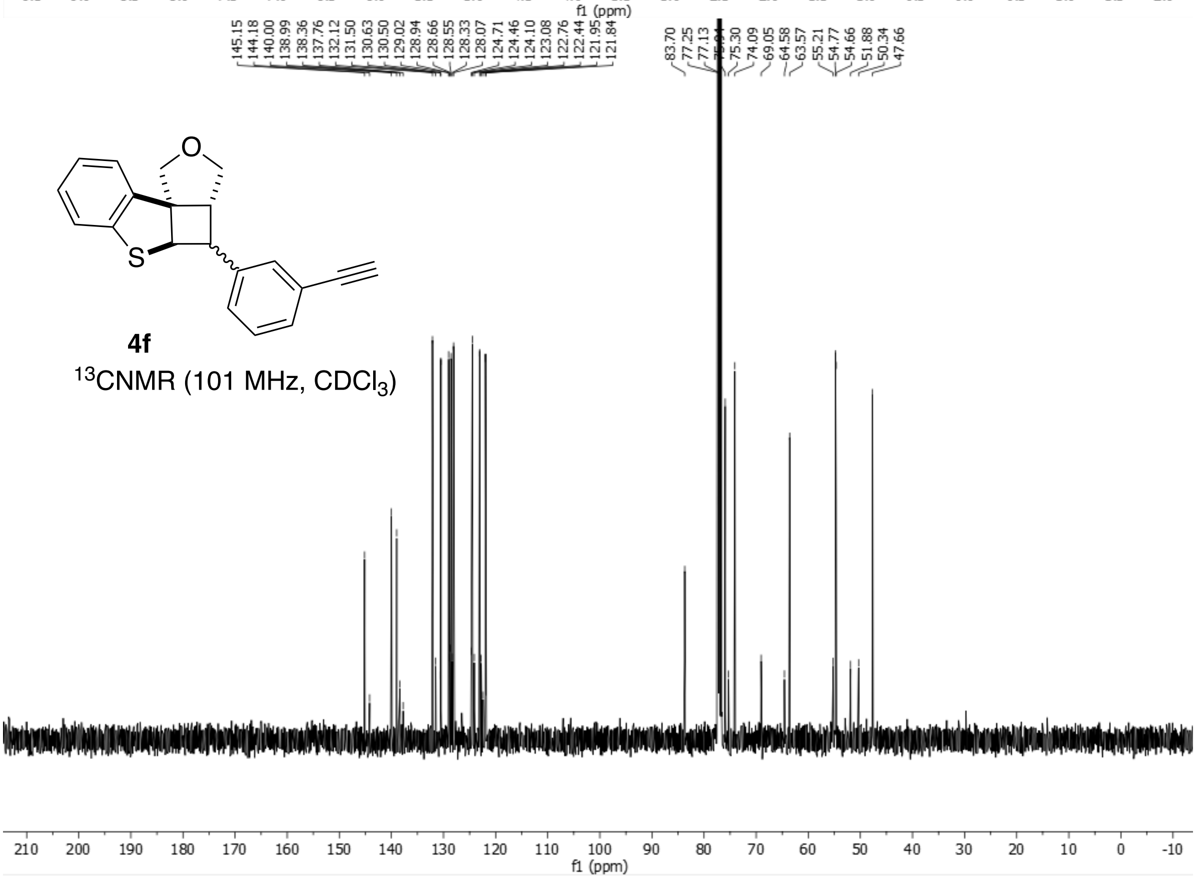

4f
^{13}CNMR (101 MHz, CDCl$_3$)
f1 (ppm)

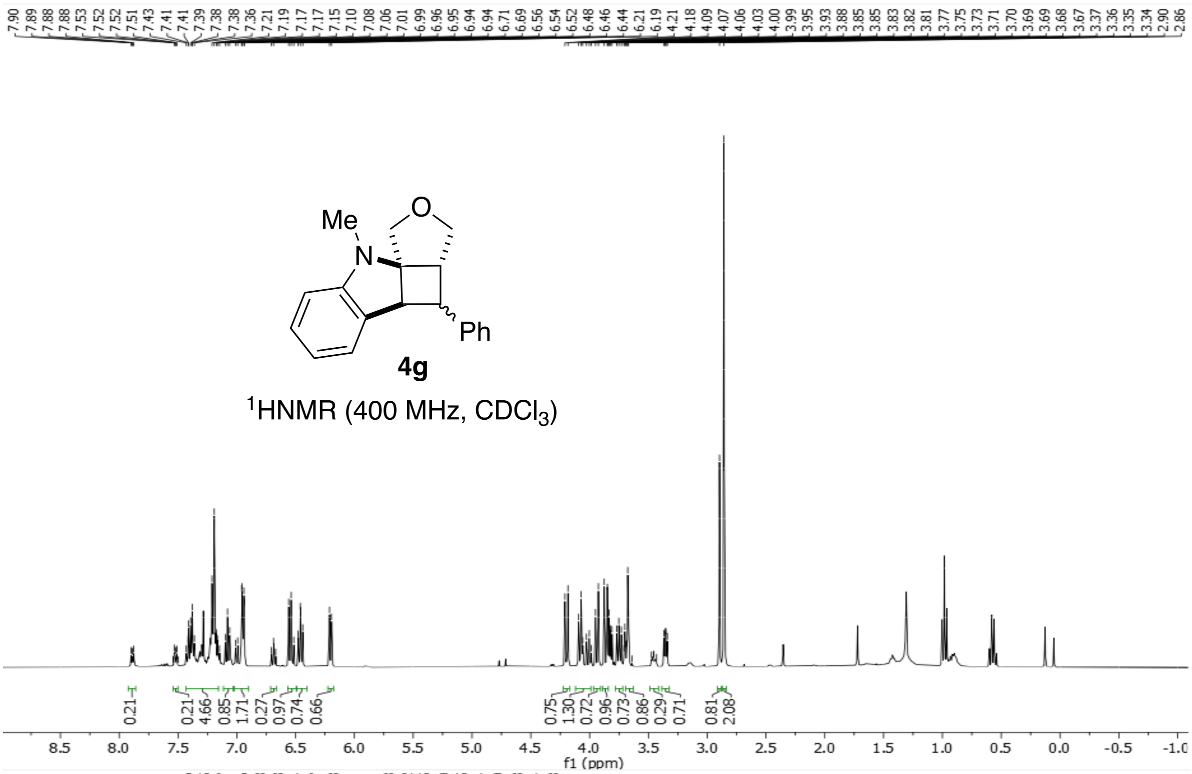
7.90 7.89 7.88 7.88 7.53 7.52 7.52 7.51 7.43 7.41 7.41 7.39 7.38 7.38 7.36 7.21 7.19 7.17 7.17 7.15 7.10 7.08 7.06 7.01 6.99 6.96 6.95 6.94 6.94 6.71 6.69 6.56 6.54 6.52 6.48 6.46 6.44 6.21 6.19 4.21 4.18 4.09 4.07 4.06 4.03 4.00 3.99 3.95 3.93 3.88 3.85 3.85 3.83 3.82 3.81 3.77 3.75 3.73 3.71 3.70 3.69 3.69 3.68 3.67 3.37 3.36 3.35 3.34 2.90 2.86
Me
N
O
Ph
4g
1HNMR (400 MHz, CDCl3)
0.21 0.21 4.66 0.85 1.71 0.27 0.97 0.74 0.66 0.75 1.30 0.72 0.96 0.73 0.86 0.29 0.71 0.81 2.08
8.5 8.0 7.5 7.0 6.5 6.0 5.5 5.0 4.5 4.0 3.5 3.0 2.5 2.0 1.5 1.0 0.5 0.0 -0.5 -1.0
f1 (ppm)

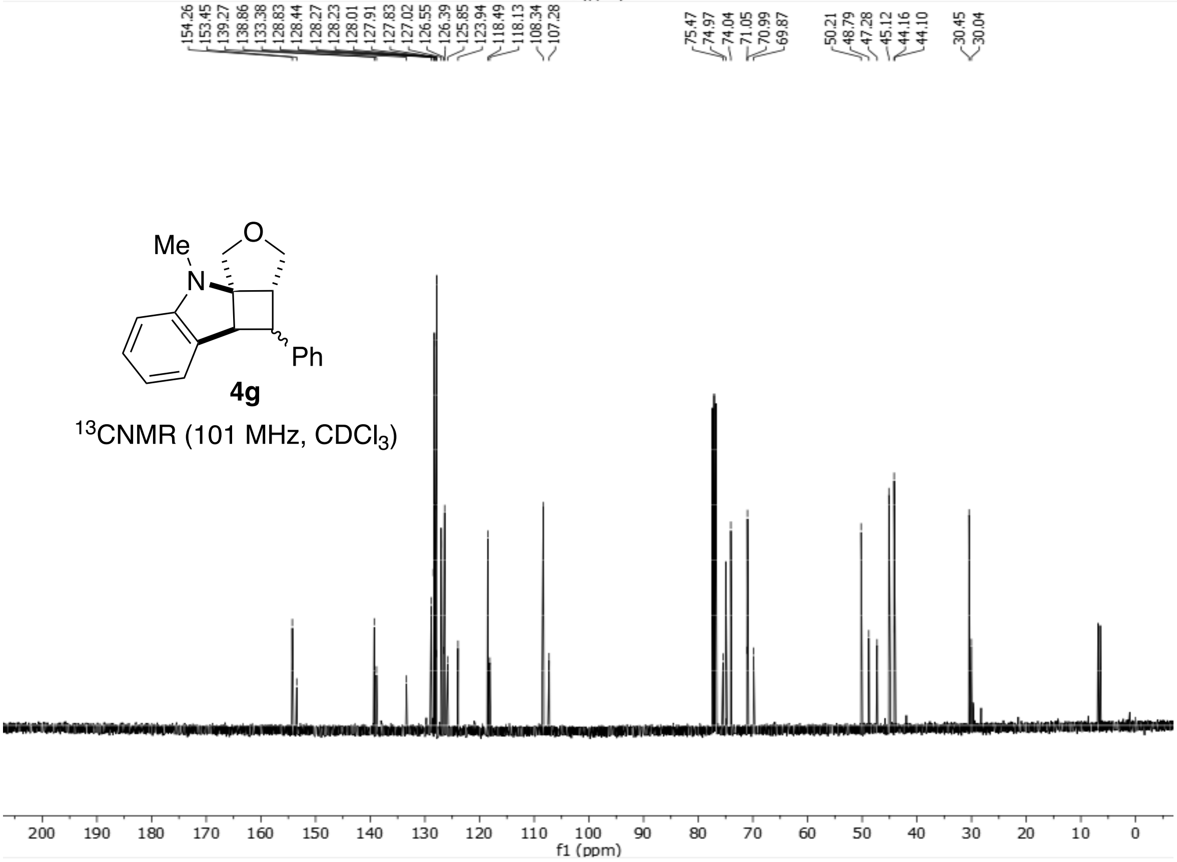
154.26 153.45 139.27 138.86 133.38 128.83 128.44 128.27 128.23 128.01 127.91 127.83 127.02 126.55 126.39 125.85 123.94 118.49 118.13 108.34 107.28 75.47 74.97 74.04 71.05 70.99 69.87 50.21 48.79 47.28 45.12 44.16 44.10 30.45 30.04
Me
N
O
Ph
4g
13CNMR (101 MHz, CDCl3)
200 190 180 170 160 150 140 130 120 110 100 90 80 70 60 50 40 30 20 10 0
f1 (ppm)

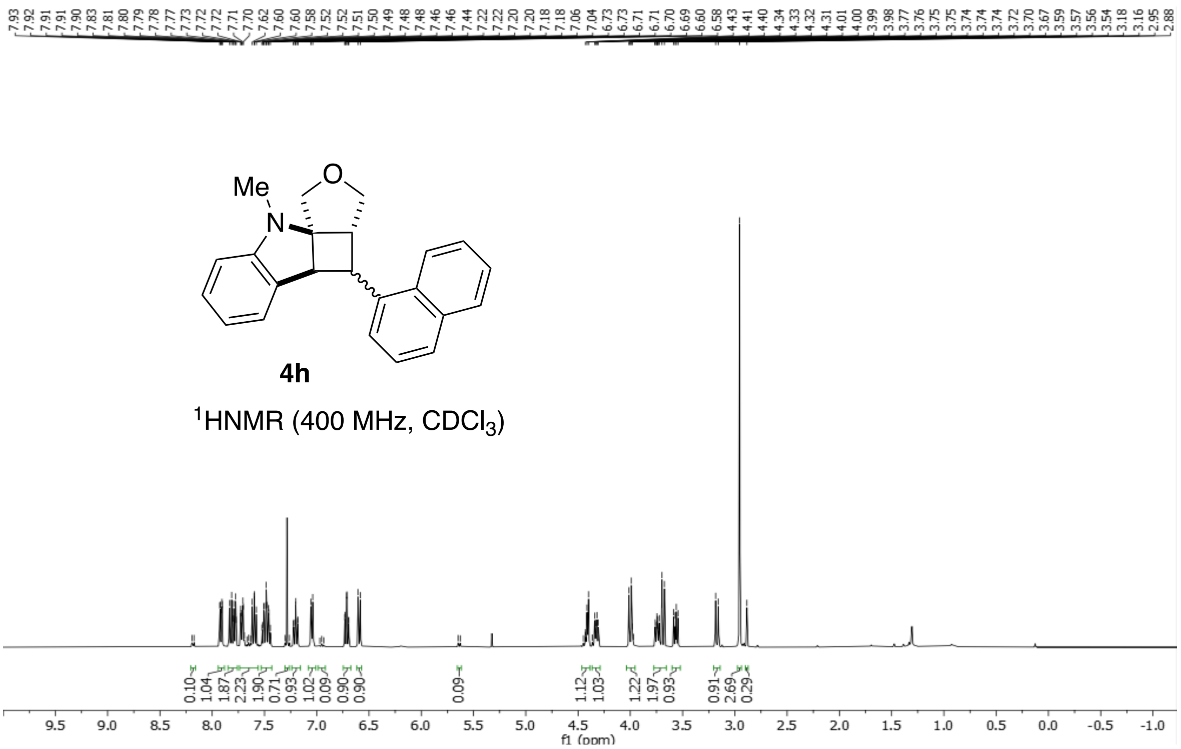
7.93 7.92 7.91 7.91 7.90 7.83 7.81 7.80 7.79 7.78 7.77 7.73 7.72 7.72 7.71 7.70 7.62 7.60 7.60 7.58 7.52 7.52 7.51 7.50 7.49 7.48 7.48 7.46 7.46 7.44 7.22 7.22 7.20 7.20 7.18 7.18 7.06 7.04 6.73 6.73 6.71 6.71 6.70 6.69 6.60 6.58 4.43 4.41 4.40 4.34 4.33 4.32 4.31 4.01 4.00 3.99 3.98 3.77 3.76 3.75 3.75 3.74 3.74 3.74 3.72 3.70 3.67 3.59 3.57 3.56 3.54 3.18 3.16 2.95 2.88
Me
N
O
4h
1HNMR (400 MHz, CDCl3)
0.10 1.04 1.87 2.23 1.90 0.71 0.93 1.02 0.09 0.90 0.90 0.09 1.12 1.03 1.22 1.97 0.93 0.91 2.69 0.29
9.5 9.0 8.5 8.0 7.5 7.0 6.5 6.0 5.5 5.0 4.5 4.0 3.5 3.0 2.5 2.0 1.5 1.0 0.5 0.0 -0.5 -1.0
f1 (ppm)

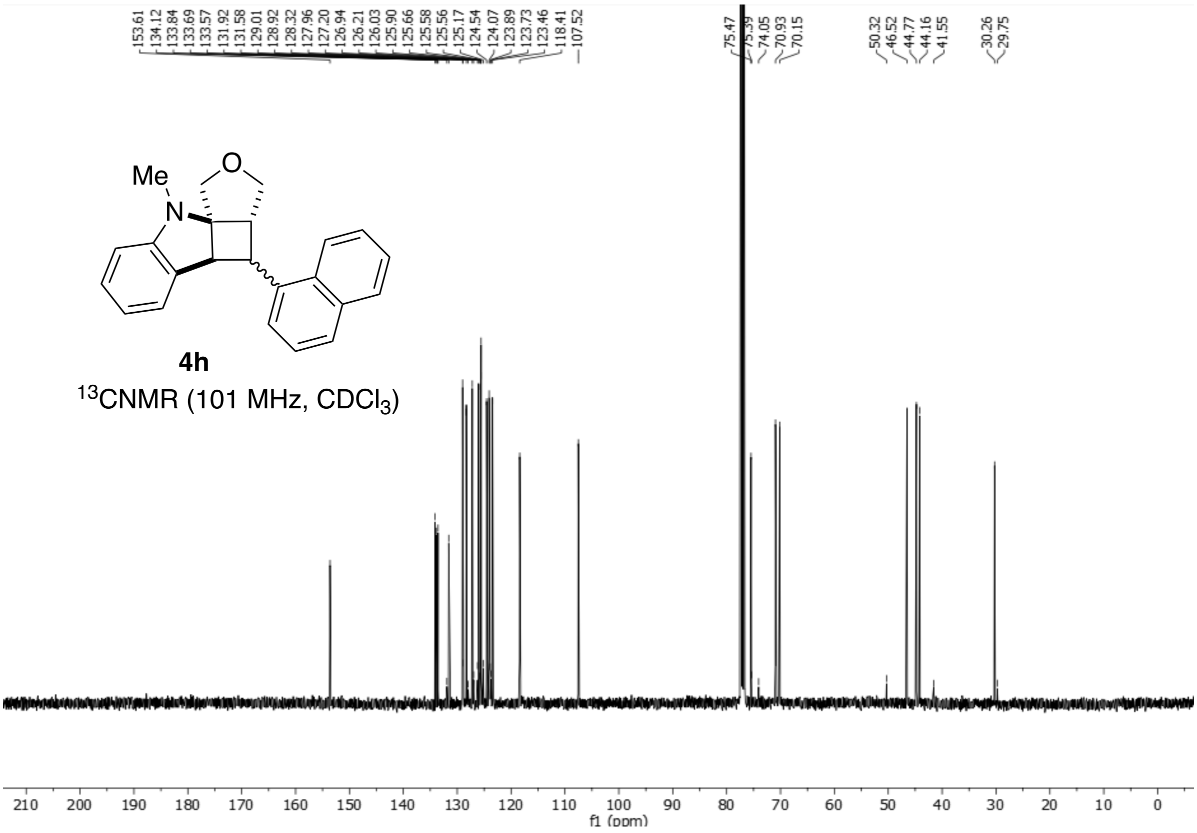
153.61 134.12 133.84 133.69 133.57 131.92 131.58 129.01 128.92 128.32 127.96 127.20 126.94 126.21 126.03 125.90 125.66 125.58 125.56 125.17 124.54 124.07 123.89 123.73 123.46 118.41 107.52
75.47 75.39 74.05 70.93 70.15
50.32 46.52 44.77 44.16 41.55
30.26 29.75
Me
N
O
4h
13CNMR (101 MHz, CDCl3)
210 200 190 180 170 160 150 140 130 120 110 100 90 80 70 60 50 40 30 20 10 0
f1 (ppm)

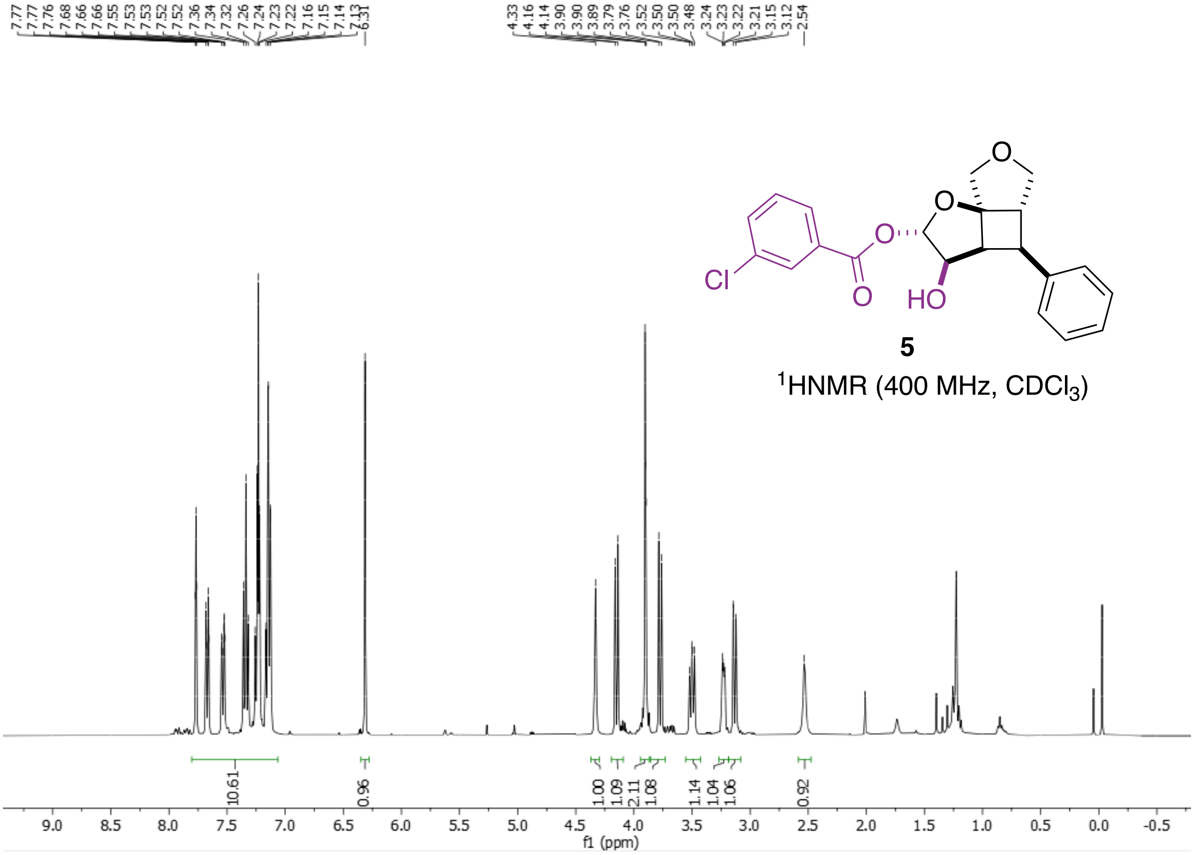
7.77 7.77 7.76 7.68 7.66 7.66 7.55 7.53 7.53 7.52 7.52 7.36 7.34 7.32 7.26 7.24 7.23 7.22 7.16 7.15 7.14 7.13 6.31
4.33 4.16 4.14 3.90 3.90 3.89 3.79 3.76 3.52 3.50 3.50 3.48 3.24 3.23 3.22 3.21 3.15 3.12 2.54
Cl
O
O
O
HO
O
5
1HNMR (400 MHz, CDCl3)
10.61 0.96 1.00 1.09 2.11 1.08 1.14 1.04 1.06 0.92
f1 (ppm)

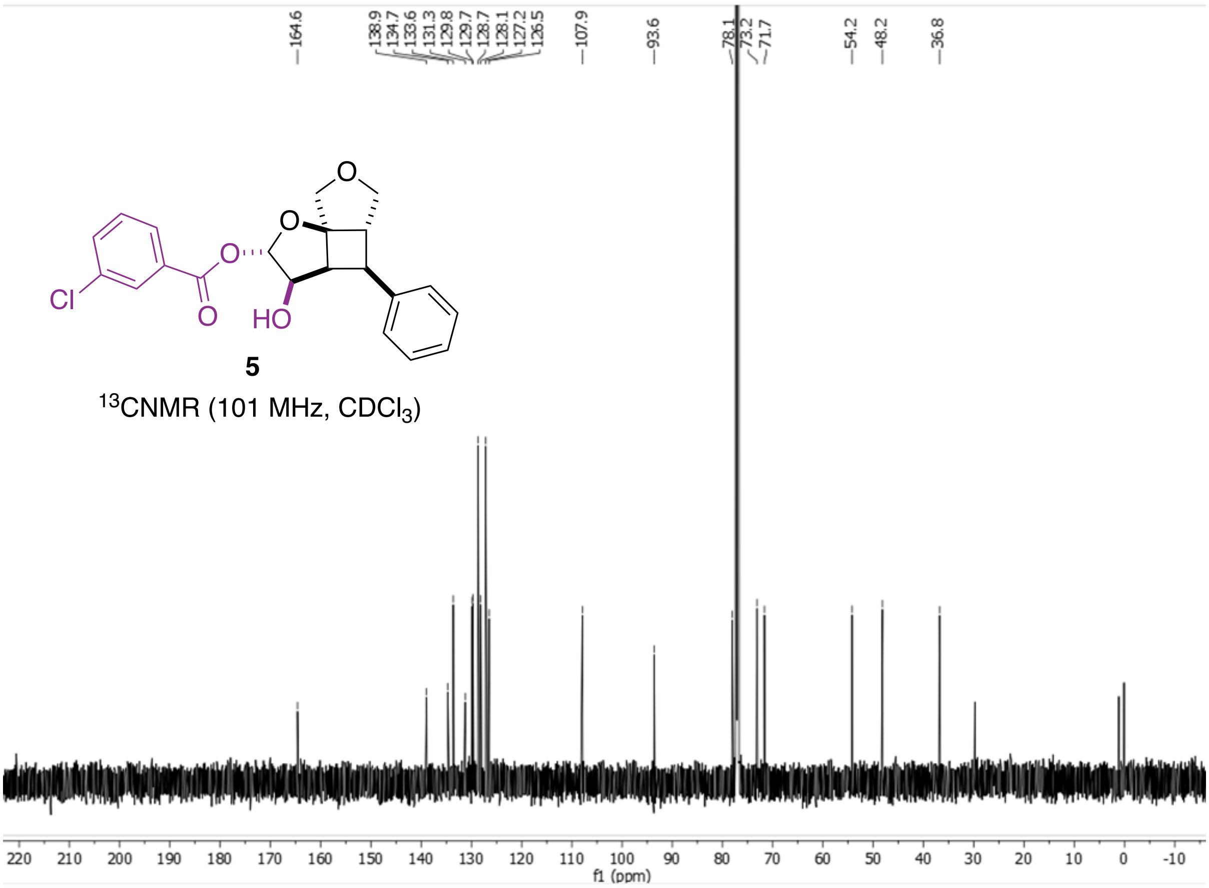
164.6
138.9 134.7 133.6 131.3 129.8 129.7 128.7 128.1 127.2 126.5
107.9
93.6
78.1 73.2 71.7
54.2
48.2
36.8
Cl
O
O
O
HO
O
5
13CNMR (101 MHz, CDCl3)
f1 (ppm)

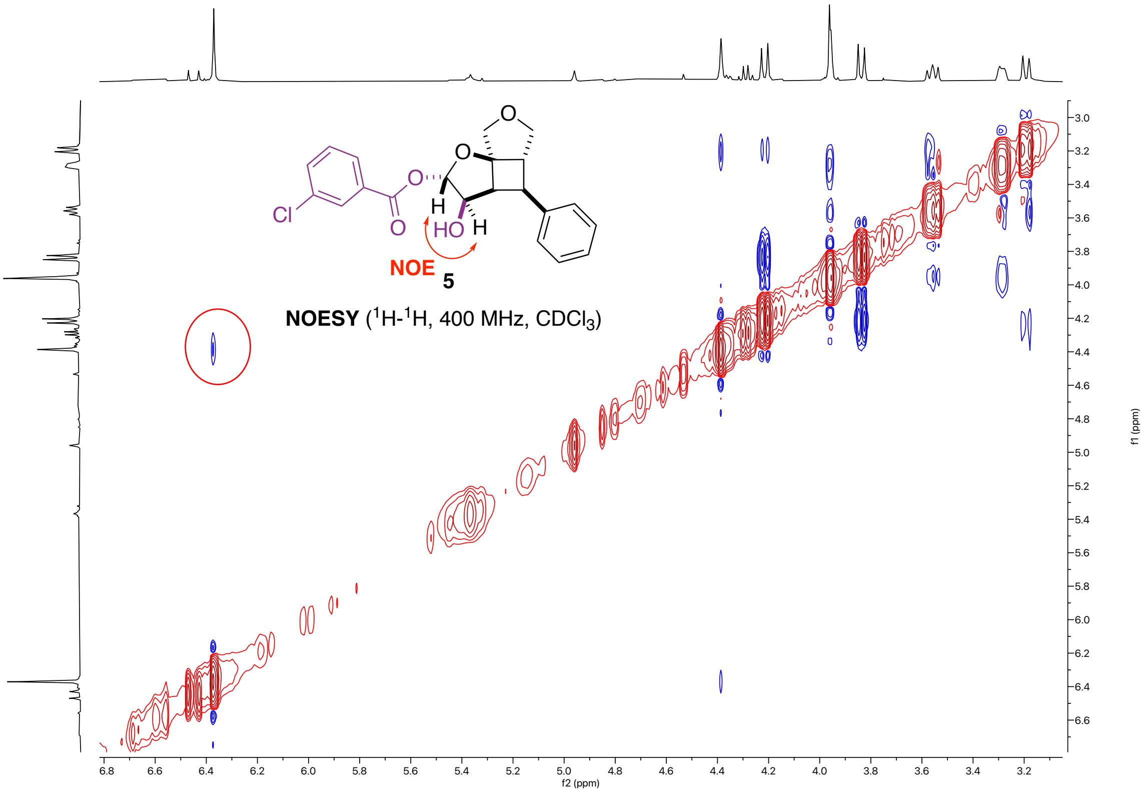

O
O
O
Cl
O
H
HO
H
NOE
5
NOESY (1H-1H, 400 MHz, CDCl3)
f2 (ppm)
f1 (ppm)

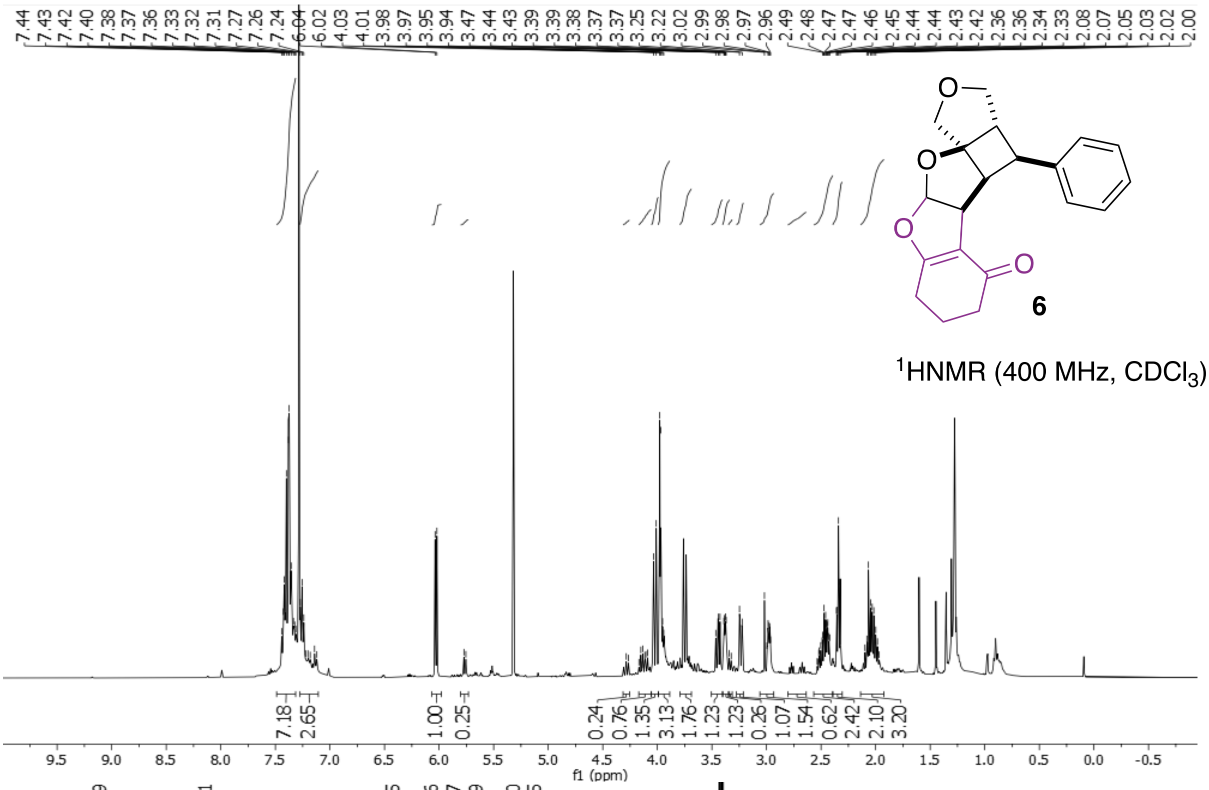

6

^{1}HNMR (400 MHz, $CDCl_3$)

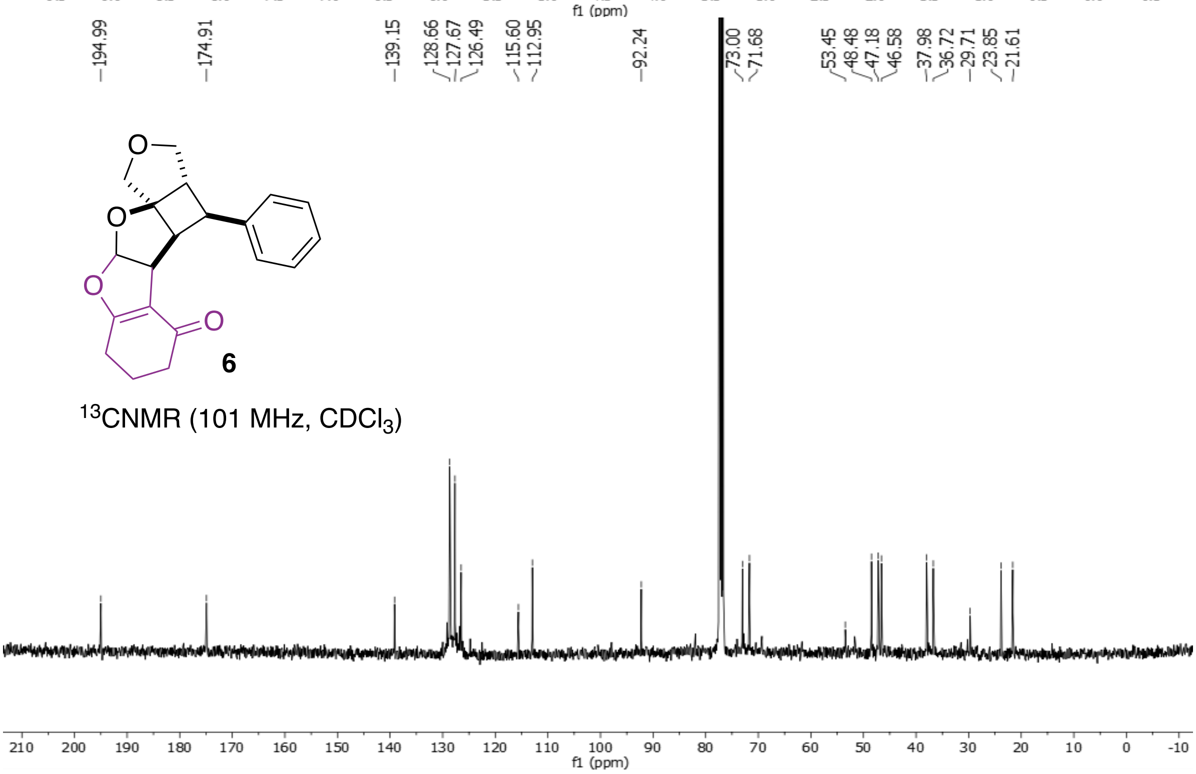

6

^{13}CNMR (101 MHz, $CDCl_3$)

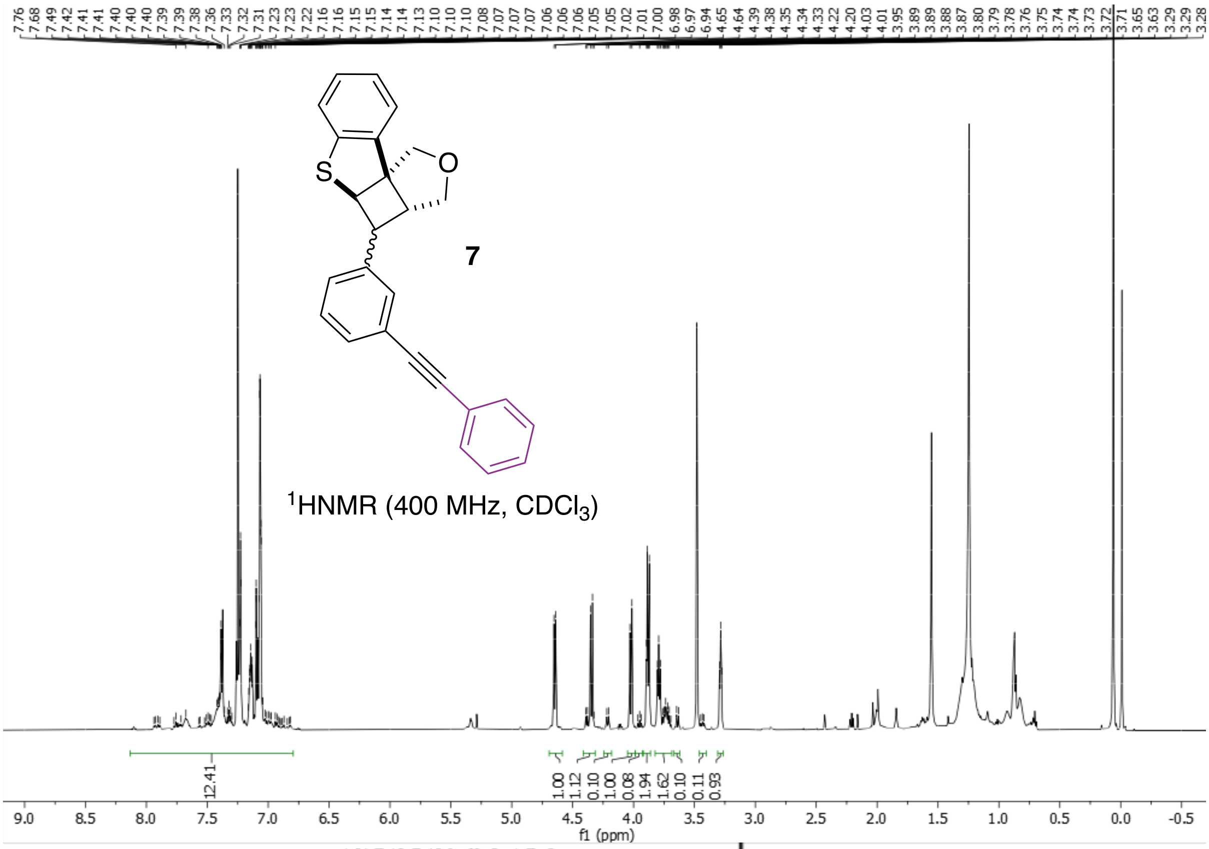
7
1HNMR (400 MHz, CDCl3)
f1 (ppm)

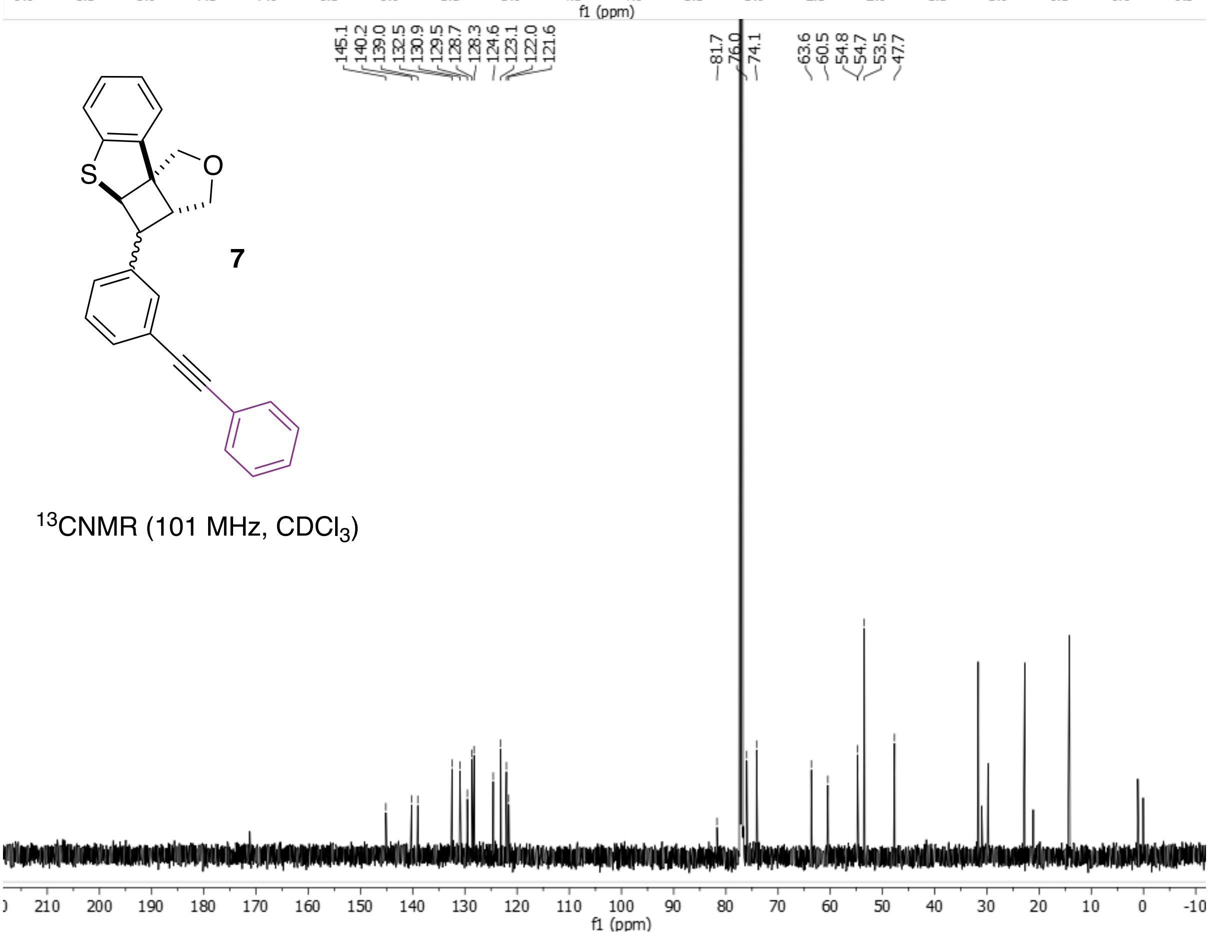
7
13CNMR (101 MHz, CDCl3)
f1 (ppm)

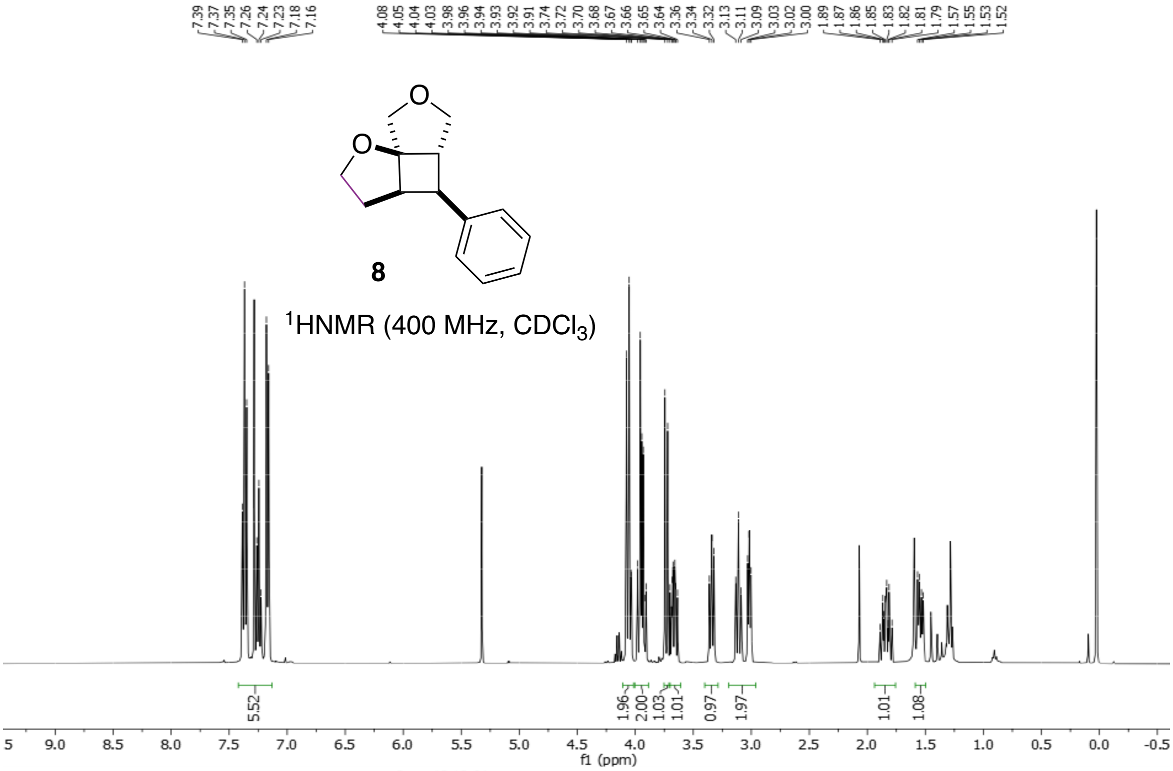
7.39 7.37 7.35 7.26 7.24 7.23 7.18 7.16
4.08 4.05 4.04 4.03 3.98 3.96 3.94 3.93 3.92 3.91 3.74 3.72 3.70 3.68 3.67 3.66 3.65 3.64 3.36 3.34 3.32 3.13 3.11 3.09 3.03 3.02 3.00 1.89 1.87 1.86 1.85 1.83 1.82 1.81 1.79 1.57 1.55 1.53 1.52
O
O
8
1HNMR (400 MHz, CDCl3)
5.52
1.96
2.00
1.03
1.01
0.97
1.97
1.01
1.08
f1 (ppm)

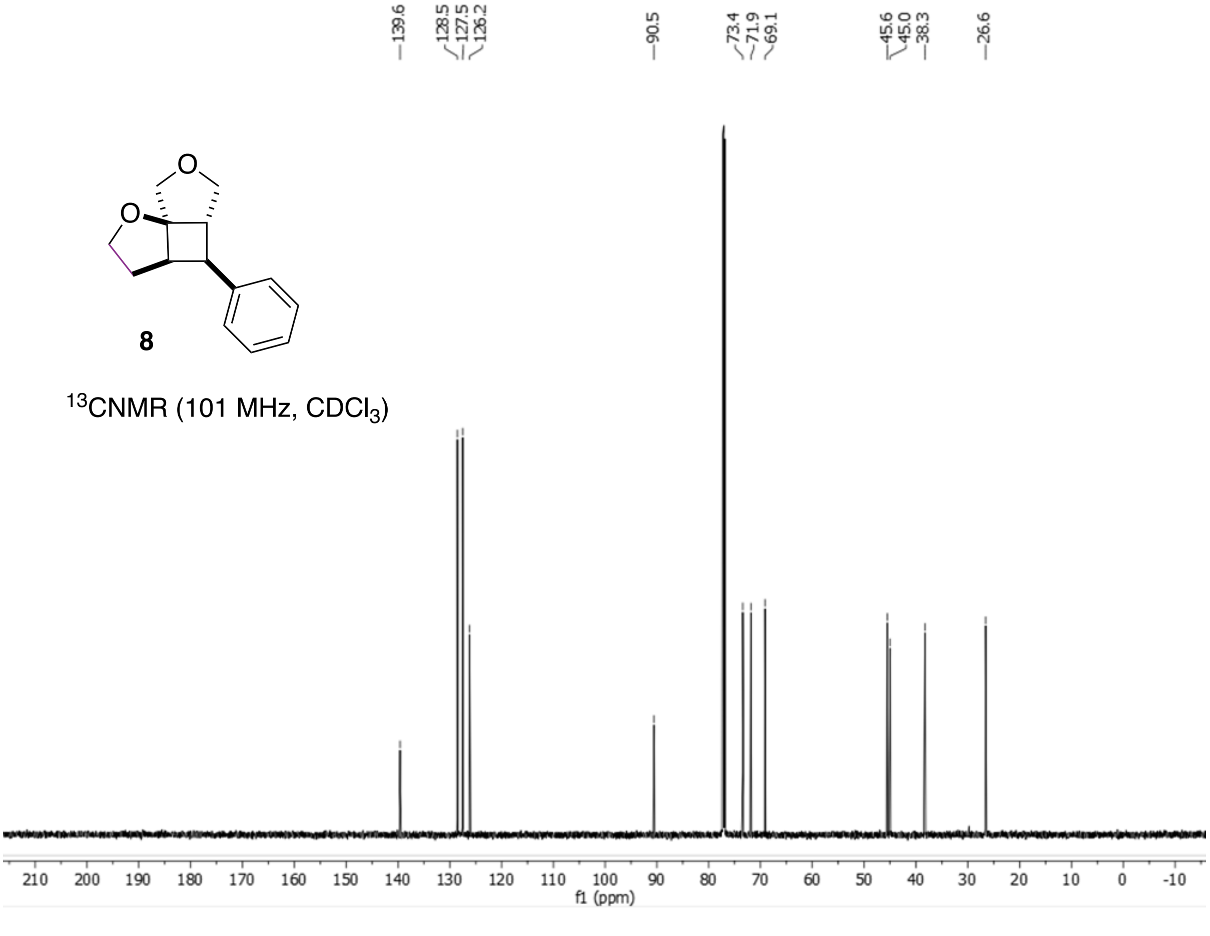
139.6
128.5
127.5
126.2
90.5
73.4
71.9
69.1
45.6
45.0
38.3
26.6
O
O
8
13CNMR (101 MHz, CDCl3)
f1 (ppm)

References

[1] Templ, J.; Schnürch, M. Allylation of C-, N-, and O-Nucleophiles via a Mechanochemically-Driven Tsuji–Trost Reaction Suitable for Late-Stage Modification of Bioactive Molecules. *Angew. Chem.* **2024**, *136* (1), e202314637

[2] Chiminelli, M.; Scarica, G.; Serafino, A.; Marchiò, L.; Viscardi, R.; Maestri, G. Visible-Light-Promoted Tandem Skeletal Rearrangement/Dearomatization of Heteroaryl Enallenes. *Molecules* **2024**, 29 (3), 595

[3] Moore, P. W.; Mirzayans, P. M.; Williams, C. M. NMO·TPB: A Selectivity Variation on the Ley–Griffith TPAP Oxidation. *Chem. – Eur. J.* **2015**, *21* (9), 3567–3571

[4] Plutschack, M. B.; Seeberger, P. H.; Gilmore, K. Visible-Light-Mediated Achmatowicz Rearrangement. *Org. Lett.* **2017**, *19* (1), 30–33

[5] Lanzi, M.; Santacroce, V.; Balestri, D.; Marchiò, L.; Bigi, F.; Maggi, R.; Malacria, M.; Maestri, G. Visible-Light-Promoted Polycyclizations of Dienynes. *Angew. Chem. Int. Ed.* **2019**, *58* (20), 6703–6707

[6] Teegardin, K.; Day, J. I.; Chan, J.; Weaver, J. Advances in Photocatalysis: A Microreview of Visible Light Mediated Ruthenium and Iridium Catalyzed Organic Transformations. *Org. Process Res. Dev.* **2016**, *20* (7), 1156–1163

[7] Ruggeri, D.; Hoch, M.; Spataro, D.; Marchiò, L.; Protti, S.; Cauzzi, D.; Tegoni, M.; Lanzi, M.; Maestri, G. Tuning the Efficiency of Iridium(III) Complexes for Energy Transfer (EnT) Catalysis through Ligand Design. *Chem. – Eur. J.* e202403309

[8] Dutta, S.; Erchinger, J. E.; Strieth-Kalthoff, F.; Kleinmans, R.; Glorius, F. Energy Transfer Photocatalysis: Exciting Modes of Reactivity. *Chem. Soc. Rev.* **2024**, *53* (3), 1068–1089

[9] Strieth-Kalthoff, F.; Glorius, F. Triplet Energy Transfer Photocatalysis: Unlocking the Next Level. *Chem* **2020**, *6* (8), 1888–1903

[10] Attolino, E.; Catelani, G.; D'Andrea, F.; Landi, M. A New and Efficient Entry to D-Xylo-Hexos-4-Ulose and Some Derivatives Thereof through Epoxidation of the 3,4-Hexeno Derivative of Diacetone-d-Glucose. *Carbohydrate Research* **2006**, *341* (*15*), 2498–2506

[11] Schreiber, S. L.; Satake, K. Studies of the Furan-Carbonyl Photocycloaddition Reaction: The Determination of the Absolute Stereostructure of Asteltoxin. *Tetrahedron Letters* **1986**, *27* (*23*), 2575–2578.

[12] Roy, S. C.; Mandal, P. K. Synthesis of Fused Acetals by Ceric Ammonium Nitrate Mediated Cycloaddition of 1,3-Dicarbonyl Compounds to Cyclic Enol Ethers. *Tetrahedron* **1996**, *52* (38), 12495–12498

[13] Schreiber, S. L.; Satake, K. Studies of the Furan-Carbonyl Photocycloaddition Reaction: The Determination of the Absolute Stereostructure of Asteltoxin. *Tetrahedron Lett.* **1986**, *27* (23), 2575–2578

[14] Sparascio, S.; Scarica, G.; Cerveri, A.; Russo, G.; Spataro, D.; Marchiò, L.; Lanzi, M.; Maestri, G. Synthesis of Vinyl- Azetidines and Beta-Lactams from Allenamides via Energy-Transfer Relay. *ChemRxiv* February 17, **2025**

[15] Strieth-Kalthoff, F.; James, M. J.; Teders, M.; Pitzer, L.; Glorius, F. Energy Transfer Catalysis Mediated by Visible Light: Principles, Applications, Directions. *Chem. Soc. Rev.* **2018**, *47* (19), 7190–7202.

[16] Gaussian 16, Revision B.01, Frisch, M. J.; Trucks, G. W.; Schlegel, H. B.; Scuseria, G. E.; Robb, M. A.; Cheeseman, J. R.; Scalmani, G.; Barone, V.; Petersson, G. A.; Nakatsuji, H.; Li, X.; Caricato, M.; Marenich, A. V.; Bloino, J.; Janesko, B. G.; Gomperts, R.; Mennucci, B.; Hratchian, H. P.; Ortiz, J. V.; Izmaylov, A. F.; Sonnenberg, J. L.; Williams-Young, D.; Ding, F.; Lipparini, F.; Egidi, F.; Goings, J.; Peng, B.; Petrone, A.; Henderson, T.; Ranasinghe, D.; Zakrzewski, V. G.; Gao, J.; Rega, N.; Zheng, G.; Liang, W.; Hada, M.; Ehara, M.; Toyota, K.; Fukuda, R.; Hasegawa, J.; Ishida, M.; Nakajima, T.; Honda, Y.; Kitao, O.; Nakai, H.; Vreven, T.; Throssell, K.; Montgomery, J. A., Jr.; Peralta, J. E.; Ogliaro, F.; Bearpark, M. J.; Heyd, J. J.; Brothers, E. N.; Kudin, K. N.; Staroverov, V. N.; Keith, T. A.; Kobayashi, R.; Normand, J.; Raghavachari, K.; Rendell, A. P.; Burant, J. C.; Iyengar, S. S.; Tomasi, J.; Cossi, M.; Millam, J. M.; Klene, M.; Adamo, C.; Cammi, R.; Ochterski, J. W.; Martin, R. L.; Morokuma, K.; Farkas, O.; Foresman, J. B.; Fox, D. J. Gaussian, Inc., Wallingford CT, 2016

[17] Zhao, Y.; Truhlar, D. G. The M06 Suite of Density Functionals for Main Group Thermochemistry, Thermochemical Kinetics, Noncovalent Interactions, Excited States, and Transition Elements: Two New Functionals and Systematic Testing of Four M06-Class Functionals and 12 Other Functionals. Theor. *Chem. Acc*. **2008**, *120*, 215

[18] Weigend, F.; Ahlrichs, R. Balanced Basis Sets of Split Valence, Triple Zeta Valence and Quadruple Zeta Valence Quality for H to Rn: Design and Assessment of Accuracy. *Phys. Chem. Chem. Phys.* **2005**, *7*, 3297

[19] Barone, V.; Cossi, M. Quantum Calculation of Molecular Energies and Energy Gradients in Solution by a Conductor Solvent Model. *J. Phys. Chem. A* **1998**, *102*, 1995

[20] Cossi, M.; Rega, N.; Scalmani, G.; Barone, V.Energies, Structures, and Electronic Properties of Molecules in Solution with the C-PCM Solvation Model. *J. Comput. Chem.* **2003**, *24*, 669

[21] Grimme, S.; Antony, J.; Ehrlich S.; Krieg, H. A consistent and accurate ab initio parameterization of density functional dispersion correction (DFT-D) for the 94 elements HPu. *J. Chem. Phys.* **2010**, *132*, 154104

[22] Humphrey, W., Dalke, A. and Schulten, K., "VMD - Visual Molecular Dynamics", J. Molec. Graphics, 1996, vol. 14, pp. 33-38.
